# Peptidic boronic acid *Plasmodium falciparum* SUB1 inhibitors with improved selectivity over human proteasome

**DOI:** 10.1021/acs.jmedchem.4c01005

**Published:** 2024-07-25

**Authors:** Chrislaine Withers-Martinez, Elina Lidumniece, Fiona Hackett, Christine R. Collins, Zahie Taha, Michael J. Blackman, Aigars Jirgensons

**Affiliations:** a Malaria Biochemistry Laboratory, The Francis Crick Institute, London NW1 1AT, United Kingdom; b Latvian Institute of Organic Synthesis, Riga LV-1006, Latvia; c Faculty of Infectious and Tropical Diseases, London School of Hygiene & Tropical Medicine, London WC1E 7HT, United Kingdom

## Abstract

*Plasmodium falciparum* subtilisin-like serine protease 1 (PfSUB1) is essential for egress of invasive merozoite forms of the parasite, rendering PfSUB1 an attractive antimalarial target. Here we report studies aimed to improve drug-like properties of peptidic boronic acid PfSUB1 inhibitors including increased lipophilicity and selectivity over human proteasome (H20S). Structure-activity relationship investigations revealed that lipophilic P_3_ amino acid side chains as well as *N*-capping groups were well tolerated in retaining PfSUB1 inhibitory potency. At the P_1_ position, replacing the methyl group with a carboxyethyl substituent led to boralactone PfSUB1 inhibitors with remarkably improved selectivity over H20S. Combining lipophilic end-capping groups with the boralactone reduced the selectivity over H20S. However, compound **4c** still showed >60-fold selectivity *versus* H20S and low nanomolar PfSUB1 inhibitory potency. Importantly this compound inhibited growth of a genetically modified *P. falciparum* line expressing reduced levels of PfSUB1 13-fold fold more efficiently compared to a wild-type parasite line.

## Introduction

Malaria is a global health challenge impacting on the lives of around half of the earth’s population.^1^ The vector born disease is caused by obligate intracellular parasites of the genus *Plasmodium*, with *P. falciparum* being the most dangerous species. Extensive efforts to control and eradicate malaria, particularly the availability of antimalarial drugs, have resulted in considerable reductions in mortality due to the disease over recent decades.^2^ However widespread resistance of the parasite to many currently used drugs, including artemisinin-based combinations (ACTs), is of great concern.^3–5^ There is a widely accepted need to strengthen the antimalarial drug pipeline through the identification of new classes of antimalarial drugs with new modes of action.^6–9^

All the clinical manifestations of malaria are caused by cycles of asexual parasite proliferation within red blood cells (RBCs). Specialised developmental forms called merozoites invade the RBC and rapidly transform within a parasitophorous vacuole (PV) into feeding forms called trophozoites. Over a period of around 48 hours in the case of *P. falciparum*, the intracellular parasite undergoes nuclear division and segmentation to form 16 or more daughter merozoites. These are released from the cell in a lytic process called egress to allow the merozoites to invade fresh RBCs and repeat the cycle. Egress is regulated by a parasite enzyme pathway, with a central role for a calcium-dependent serine protease called subtilisin-like serine protease 1 (SUB1).

**Figure 1:**
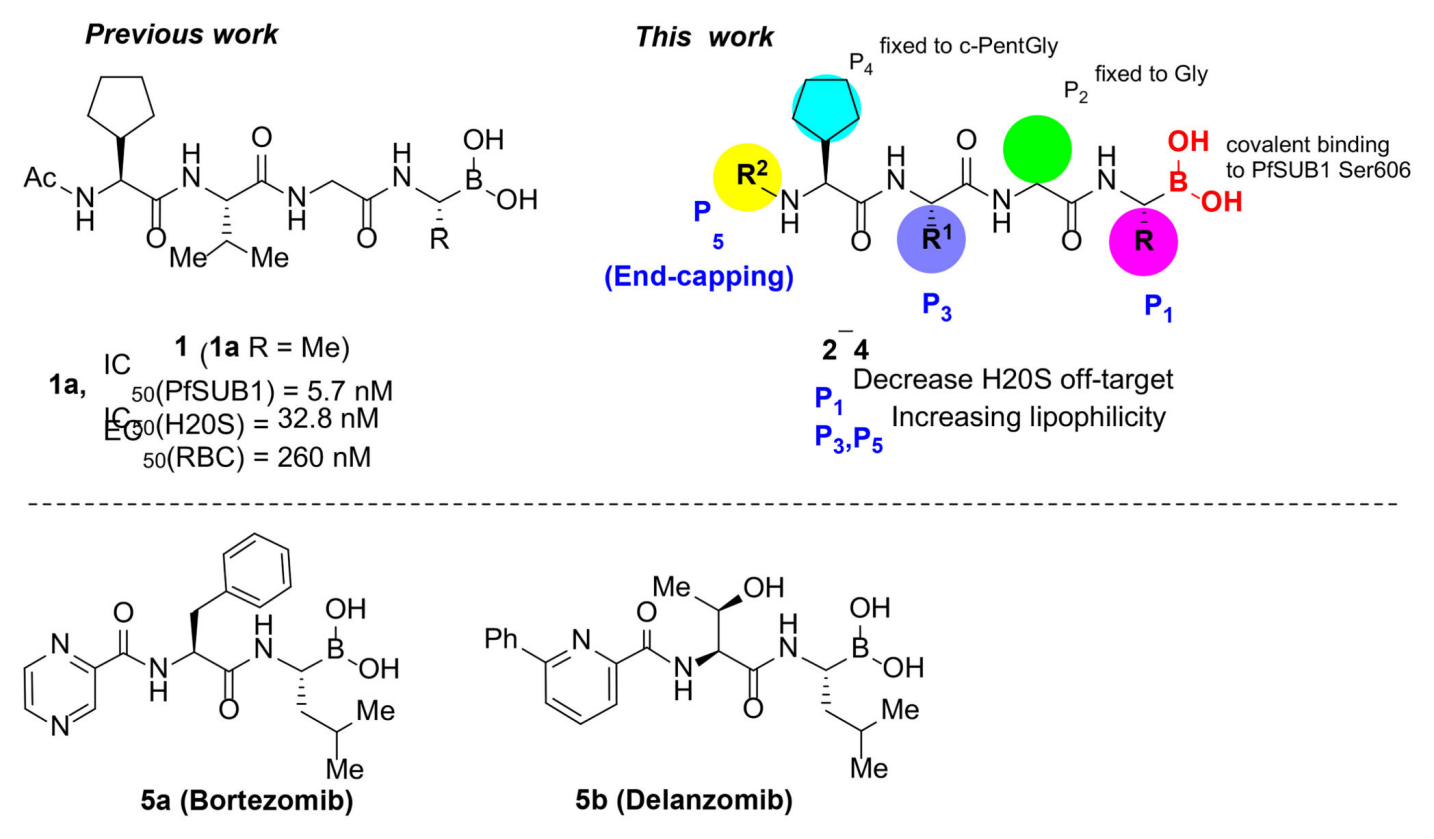
Development of peptidic boronic acids 1a into more selective PfSUB1 inhibitors

Over recent years, SUB1 has emerged as an attractive potential target for antimalarial drug discovery.^10^ The enzyme is initially stored in a set of merozoite secretory organelles, then discharged into the PV lumen just prior to egress where it cleaves and activate a number of proteins of the PV and merozoite surface. This rapidly leads to explosive rupture of the PV membrane (PVM) and RBC membrane to allow the release of invasive merozoites.^11–14^ A single orthologue of SUB1 is found in the genomes of all known *Plasmodium* species, and a critical role of SUB1 for parasite survival has been genetically confirmed through the demonstration that *SUB1* gene disruption results in a complete block in merozoite egress in asexual blood stages of the parasite life cycle and the preceding liver stages of infection. ^15–17^ Several small molecule SUB1 inhibitors have been discovered either by screening of compound libraries or by rational design based on the established substrate specificity and structure of the enzyme.^18–27^ Recently, we have developed peptidic boronic acids **1** as *P. falciparum* SUB1 (PfSUB1) inhibitors with low nanomolar potency (Figure 1).^28^ Selected inhibitors from these series prevented *P. falciparum* egress and inhibited parasite growth *in vitro* at submicromolar concentrations.

Follow up investigations of PfSUB1 inhibitor **1a** revealed that it also inhibits human proteasome (H20S) which may lead to off-target effects of an anti-malarial drug (Figure 1). Inhibition of human H20S by boronic acid **1a** is not unexpected given its structural resemblance to the anticancer drug bortezomib **5a**^29,30^ and the clinical candidate delanzomib (CEP-18770) **5b**,^31,32^ both of which are proteasome inhibitors. Peptidic boronic acids are also polar compounds which hampers membrane penetration and consequently efficient access to the parasite exonemes and the PV lumen where PfSUB1 is respectively stored and active against its substrates.^10^ Here we describe SAR studies of inhibitors **2−4** with the aim of improving the PfSUB1 inhibitory potency and lipophilicity of the compounds as well as to improve selectivity against H20S (Figure 1). Based on previous work^27,28^, the P_2_ position was fixed to glycine and the P_4_ position was fixed to cyclopentylglycine as these were found to be most optimal for potency of peptidic inhibitors. Provisional modeling studies has indicated that P_1_ position could be a key for achieving selectivity of PfSUB1 *vs* H20S inhibition, while lipophilic groups are tolerated at P_3_ and P_5_ (end-capping) positions.

## Results and discussion

### Synthesis

**Scheme 1:**
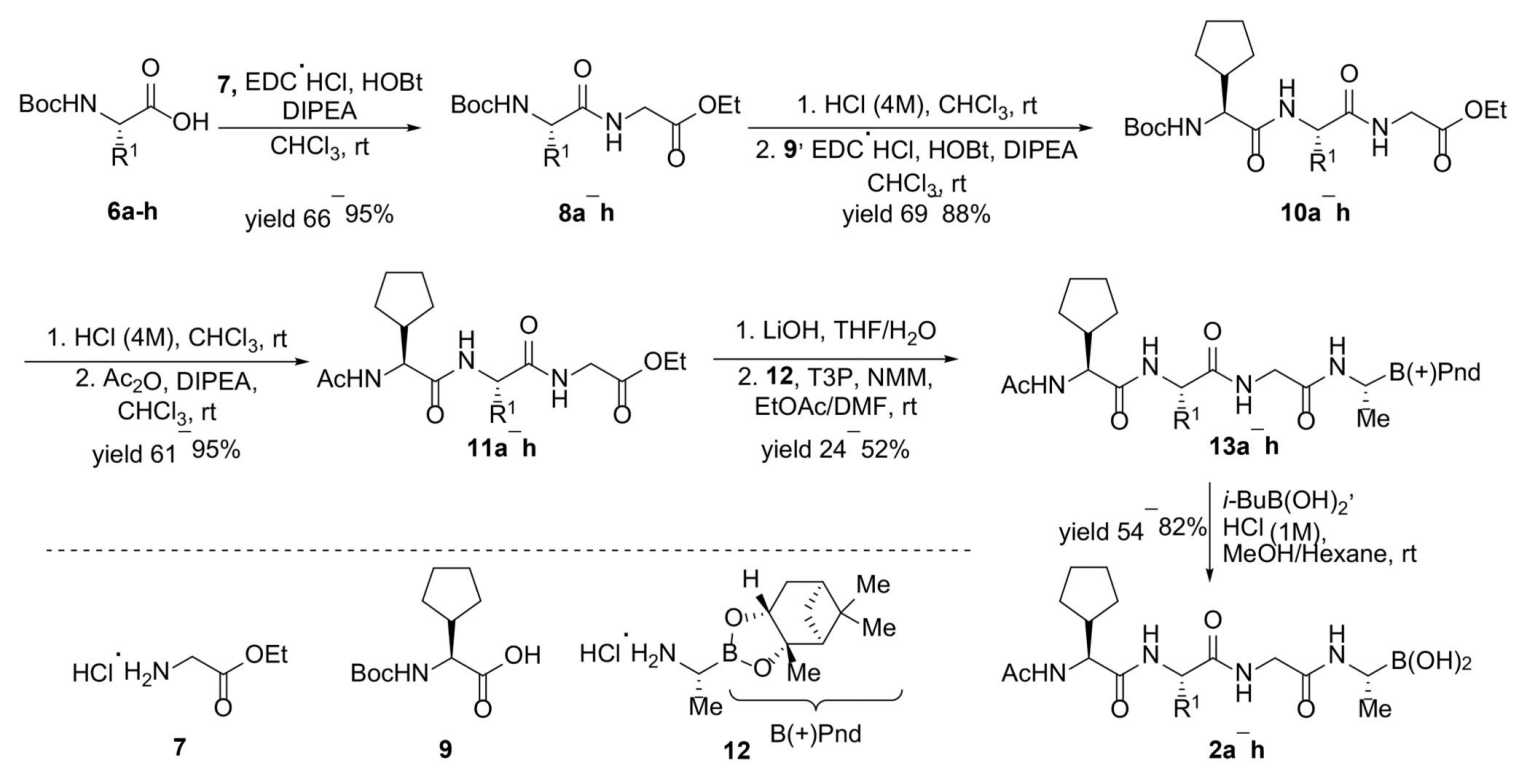
Synthesis of inhibitors 2a−h

Synthesis of inhibitors **2a−h** was performed starting from amino acids **6a−h** with various side chains (Scheme 1). These were coupled with glycine ethyl ester (**7**) to give dipeptides **8a−h**. After deprotection of the *N*-terminal Boc group, the intermediates were *N*-acetylated to give tripeptides **11a−h**. Hydrolysis of ethyl ester with LiOH and coupling of the resulting acids with an α-amino boronic acid ester building block **12** gave intermediates **13a−h**. The last step involved a transesterification reaction with isobutyl boronic acid leading to peptidic boronic acids **2a−h**.

**Scheme 2:**
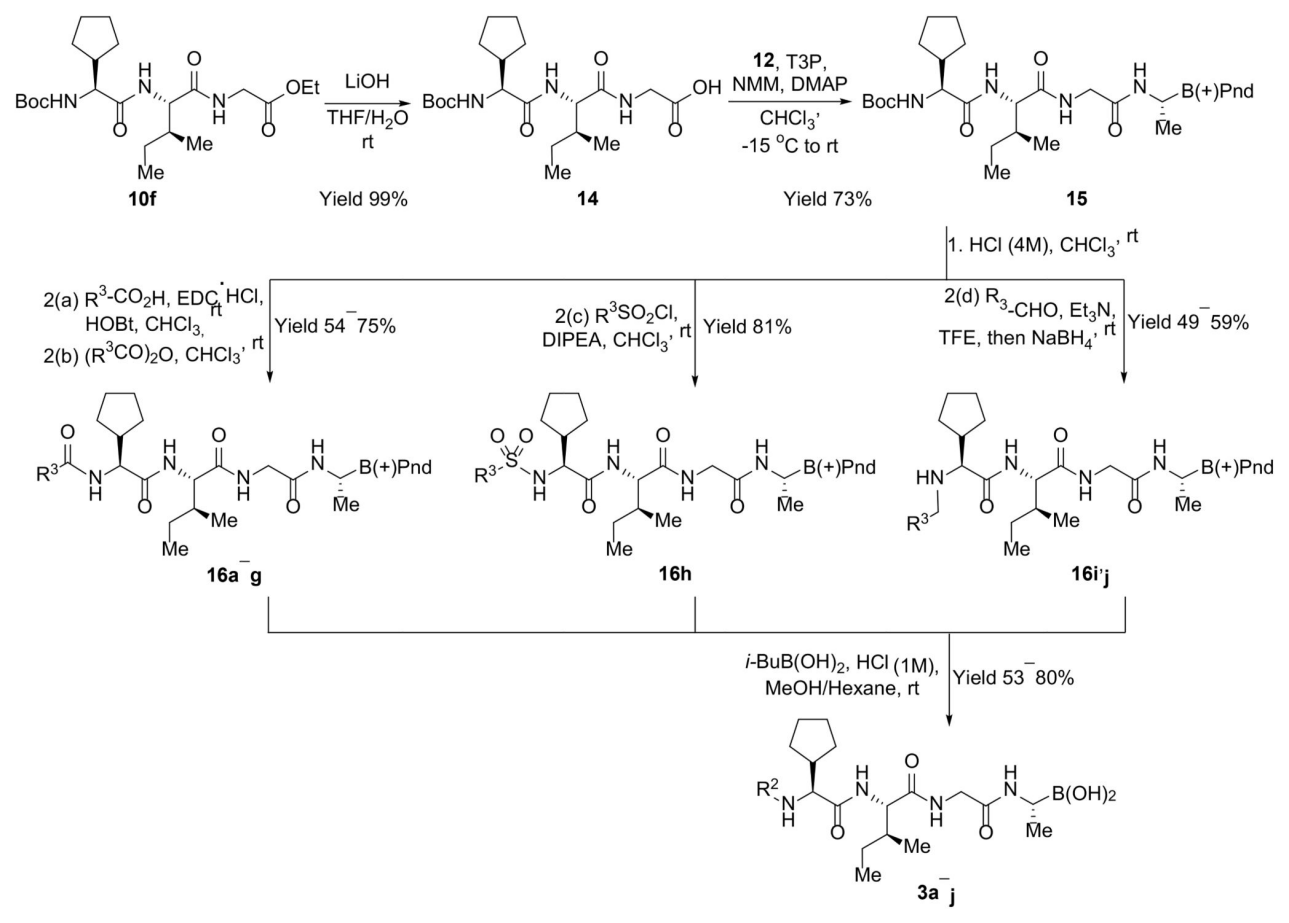
Synthesis of inhibitors 3a−j

Synthesis of inhibitors **3a-j** commenced with hydrolysis of the ester in a protected dipeptide **10f** to give an acid **14** (Scheme 2). Coupling of the acid **14** with α-amino boronic acid building block **12** provided the key intermediate **15**. This was subjected to Boc-deprotection and the resulting free amine was coupled with an either acid, acylated, sulfonylated or transformed to an *N*-monoalkylated amine via reductive amination to give protected intermediates **16a−g, 16h**, and **16i,j** respectively. The last step towards peptidic boronic acids **3a−j** was carried out as described above using transesterification with isobutyl boronic acid.

**Scheme 3:**
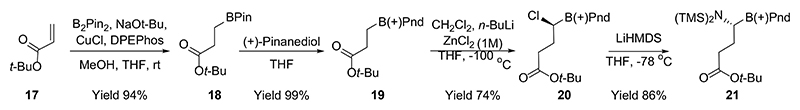
Synthesis of building block 21

For the synthesis of inhibitors **4a−c** containing glutamic acid at the P_1_ position, a synthetic route was established to access α-amino boronic acid building block **21** (Scheme 3). The double bond in *tert*-butylacrylate (**17**) was hydroborated using bis(pinacolato)diboron and a catalytic system comprising copper(I) chloride, sodium *tert*-butoxide and bis[(2-diphenylphosphino)phenyl] ether (DPEPhos) to form β-boryl ester **18** in excellent yield.^33^ After transesterification of intermediate **18** with (+)-pinanediol the subsequent Matteson homologation provided the desired chloride **20** as a pure diastereomer. The reaction of chloride **20** with LiHMDS proceeded with inversion of the stereo center at the α-carbon of boronic acid, providing bis(trimethylsilyl)amine derivative **21** as a single diastereomer.

**Scheme 4:**
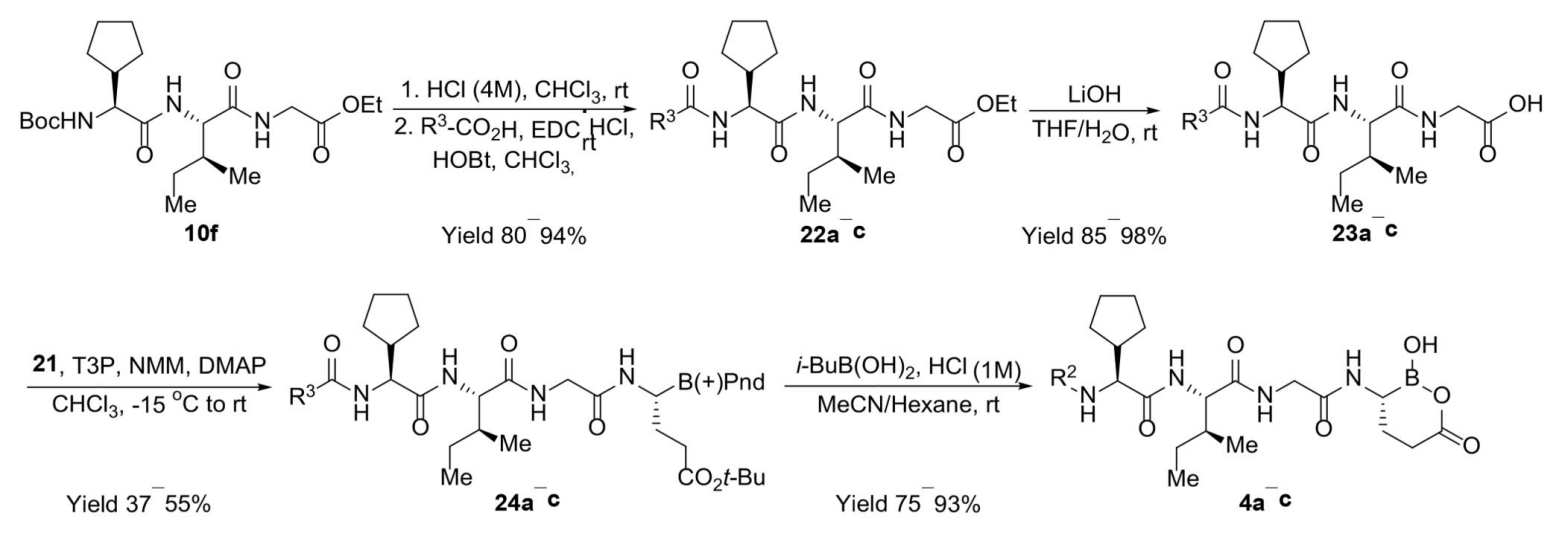
Synthesis of inhibitors 4a−c

In order to prepare inhibitors **4a−c**, the previously obtained protected dipeptide **10f** (Scheme 1) was *N*-deprotected and the resulting amine coupled with different acids to form products **22a−c** (Scheme 4). These were hydrolyzed to acids **23a−c** which were coupled with building block **21**. The resulting intermediates **24a−c** were subjected to transesterification reactions with isobutyl boronic acid and cleavage of the tert-butyl ester to obtain the final products **4a−c**. In this case, it was important to avoid methanol as a solvent for the last step since it tended to form a methyl ester of cyclic boronic acid; thus, MeCN was used instead.

### Structure-activity and selectivity relationships of PfSUB1 inhibition

Since blood-stage malaria parasites replicate in a PV within RBC, antimalarial compounds are required to cross at least 3 membranes (the RMC membrane, the PVM and the parasite plasma membrane) in order to access the intracellular parasite. Our previous work has demonstrated that replacement of threonine with leucine at the P_3_ position of peptidic boronic acids (Figure 1) significantly increased parasite growth inhibition potency without altering PfSUB1 enzyme inhibitory capacity. This pointed to beneficial effects of a lipophilic side chain in this position, likely through improving membrane permeability of the compounds. To investigate the SAR of PfSUB1 inhibition in this position we incorporated various non-proteinogenic or proteinogenic lipophilic amino acids into the structure of inhibitors **2a−h** (Table 1). Examination of the PfSUB1 enzyme inhibitory capacity of these compounds, using recombinant PfSUB1 (rPfSUB1) in an *in vitro* enzyme activity assay, revealed that **2a−h** were similarly potent inhibitors. This is probably explained by the P_3_ side chain extending out of the active site cleft of the enzyme and so playing little or no part in ligand binding affinity.^28^ The only exception was compound **2e** which showed ~20-fold decreased potency compared to its homologue **2a**.

In addition to modifications at the P_3_ position, we reasoned that the end-capping group of the peptidic boronic acids provides another option for increasing compound lipophilicity and membrane permeability, especially since this group is not predicted to be accommodated within the substrate binding cleft of the enzyme.^28^ To examine the SAR of the end-capping group, compounds **3a−j** (Table 2) were generated. These were based on inhibitor **2f** as a template since this possesses at the P_3_ position isoleucine, a naturally-occurring amino acid which confers slightly better inhibitory activity compared to analogues **2g,h**. Inhibitors **3a−c**, possessing bulky and lipophilic *N*-(quinoline-2-carboxyl)-, *N*-(5-phenylpyridine-2-carboxyl)-, or *N*-(3,5-dimethylphenylcarboxyl) groups showed comparable PfSUB1 inhibitory potency to the *N*-benzoyl analogue **3d**. Branched *N*-alkylcarboxyl substituents in inhibitors **3e−g** and an *N*-sulfonamide group in inhibitor **3h** were also well tolerated. In contrast, *N*-benzyl and *N*-pyridylmethyl substitutions (compounds **3i,j**) resulted in decreased PfSUB1 enzyme inhibitory potency.

Peptidyl boronic acid compounds such as bortezomib, ixazomib and delanzomib are potent H20S inhibitors, conferring them with anti-cancer activity. The structural similarity between our boronic acid compounds and these drugs raised concerns that our compounds might display off-target effects against H20S. Indeed, as mentioned above the previously studied peptidic boronic acid PfSUB1 inhibitor **1a** inhibits H20S with low nanomolar potency (Table 3). Replacing the P_3_ residue with threonine (compound **1b**) had practically no impact in reducing H20S inhibition. However, replacement of the P_1_ side chain substituent with a carboxyethyl group, forming the cyclic boronic acid analogue **1c**, resulted in a remarkable loss of inhibitory potency against H20S and consequently increased selectivity for PfSUB1. Inspired by this result, we installed lipophilic end-capping groups into inhibitors **4a−c**, maintaining the carboxyethyl group as the P_1_ substituent. Compounds **4a,b**, still potent PfSUB1 inhibitors, showed improved selectivity *versus* H20S compared to compounds **3a,b**, though this was less pronounced compared to the difference between compounds **1a** and **1c**. Gratifyingly however, compound **4c** showed high selectivity for PfSUB1 *versus* H20S and therefore was used for more detailed biological investigation (*vide supra*).

**Figure 2:**
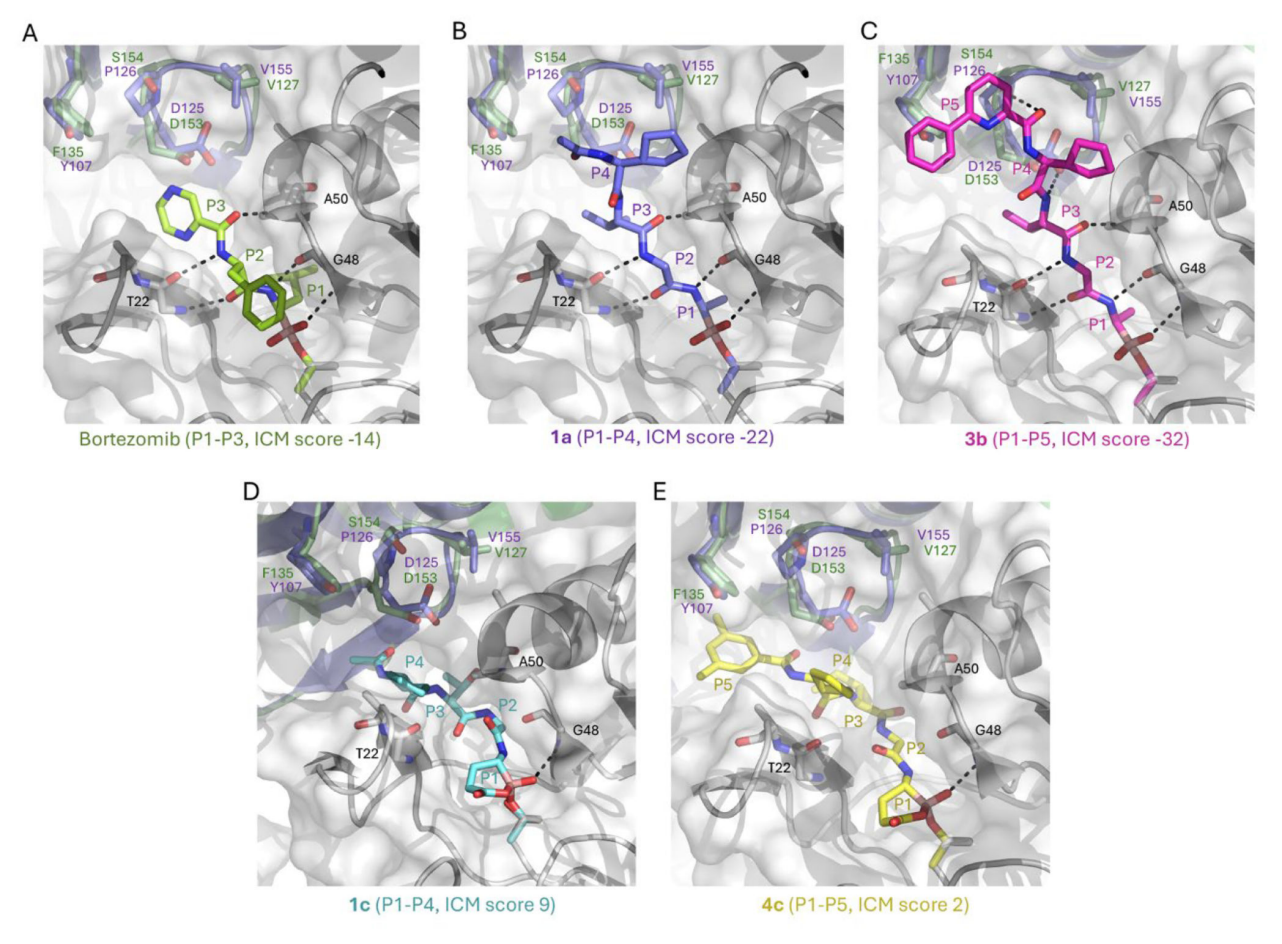
Bortezomib, compounds 1a, 3b, 1c and 4c docked into the H20S chymotrypsin-like β5 enzyme. The human 20S proteasome (H20S, PDB: 5lf4) is shown as a cartoon with a semi-transparent molecular surface, the chymotrypsin-like β5 unit (5lf4: chain K) is in light grey with its backbone chain colored by elements (O: red, N: blue), the labelled side chains of a neighboring molecule (5lf4: chain L) are shown as purple sticks with the structurally equivalent *P. falciparum* proteasome (Pf20S) molecule (7lxt: chain M) overlayed with side chains in green and labelled. The inhibitors positions P1 to P5 are indicated, the boron atom in pink. Stabilizing H-bonds are shown as black dashed lines. **A**. :bortezomib docked into the active site of β5 (ICM score -14) is shown as green sticks colored by elements. The P1 Leu side chain filled the S1 pocket. **B**.: compound **1a** docked into the active site of β5 (ICM score -22) is shown as purple sticks colored by elements. The P1 Ala side chain fitted the S1 pocket. **C**. : compound **3b** docked into the active site of β5 (ICM score -32) is shown as magenta sticks colored by elements. The P1 Ala side chain fitted the S1 pocket. The P5 phenyl pyridine capping group was stabilized in a T-shaped Pi-stacking interaction with Y107 (chain L) (structurally equivalent residue F135 in Pf20S). D.: compound **1c** docked into the active site of β5 (ICM score 9) is shown as teal sticks colored by elements. The boralactone group did not fill the S1 pocket. E.: compound **4c** docked into the active site of β5 (ICM score 2) is shown as yellow sticks colored by elements. The boralactone group did not fill the S1 pocket. The P5 dimethyl phenyl group was stabilized by neighboring 5lf4 chain L in purple (equivalent to chain M in Pf20S 7lxt in green).

The selectivity determining factors for H20S *versus* PfSUB1 were modelled by *in silico* docking of the PfSUB1 inhibitors into the active site of the H20S β5 subunit (PDB: 5lf4, delanzomib bound to H20S). Compounds **1a−c** and **3a-c** bearing a methyl group as the P_1_ side chain docked well, with best docking scores for compound **3b** (ICM score -32, Figure 2C). Bortezomib (**5a**) and delanzomib (**5b**) were included in the docking procedure which showed good agreement for delanzomib between the experimental (PDB: 5lf4) and docking poses (rmsd < 0.8Å). Interestingly **3b** appeared to dock better than bortezomib **5a** and our reference compound **1a** due additional hydrogen bonds and hydrophobic interactions at the P_5_ capping end (Figure 2ABC). These findings agreed with the experimental H20S inhibitory potency where compound **3b** was more potent than bortezomib **5a** against the chymotrypsin-like activity (β5) of H20S (see Supporting Information). Conversely, **1c** showed very weak inhibition of H20S β5 activity. When docked (Figure 2D), only one of the five hydrogen bonds involving the enzyme backbone remained with the boralactone carbonyl group facing outside of the S1 pocket and inducing an overall destabilizing effect within the active site. This effect was slightly mitigated when extending the compound at the P5 capping end (Figure 2E) possibly due to additional hydrophobic stabilization. When docking the same compounds into PfSUB1 (PDB:4lvn, Supporting Information Figure S3), bortezomib performed poorly (ICM score -7) compared to the four PfSUB1 inhibitors **1a, 3b, 1c** and **4c** (ICM scores ranging from - 26 to -41). The boralactone group in **1c** and **4c** fitted the polar S1 pocket nicely with an additional hydrogen bond and good docking scores.

### Use of a PfSUB1 knock-down *P. falciparum* line to confirm the on-target efficacy of PfSUB1 inhibitor 4c

**Figure 3:**
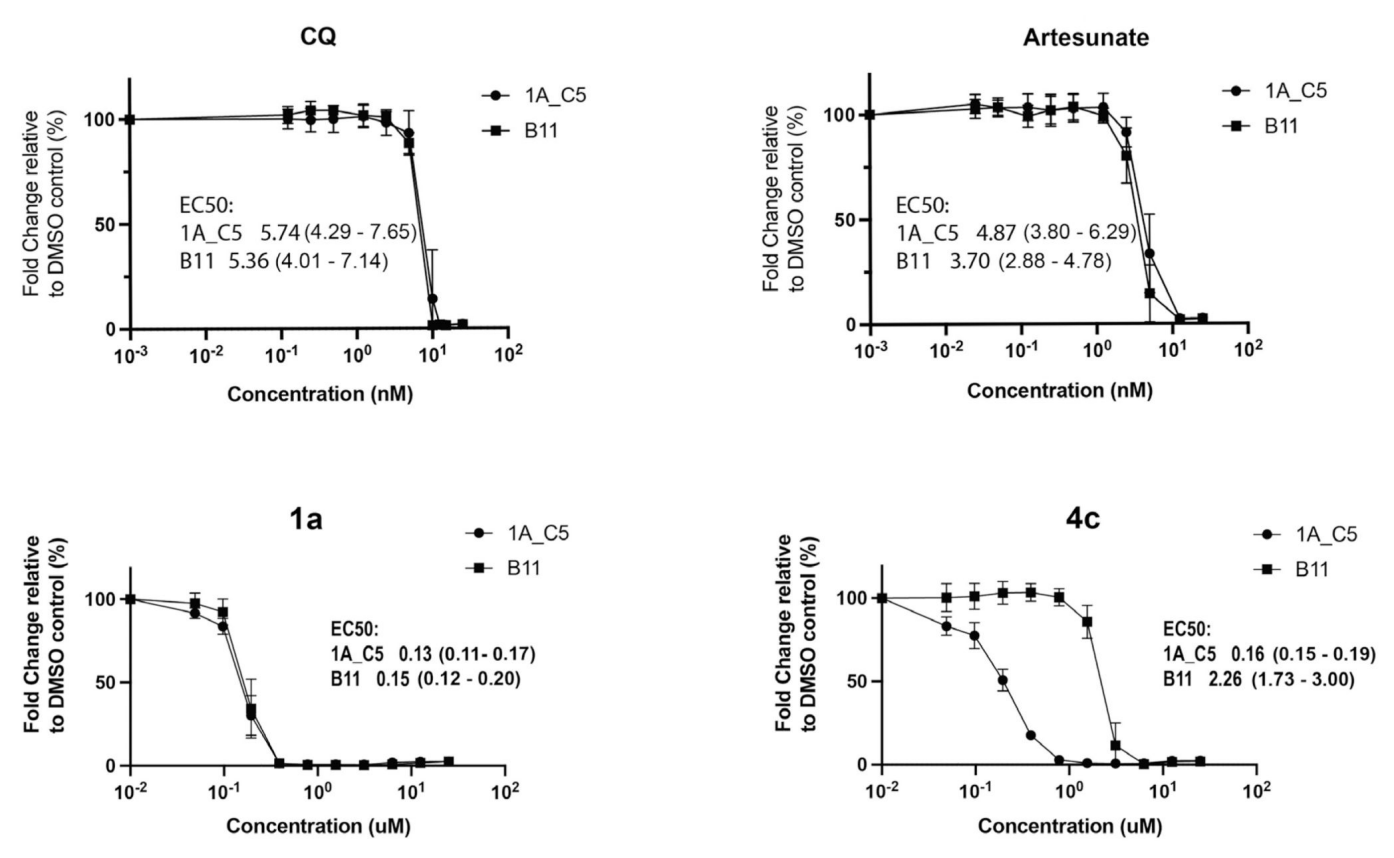
Dose-response curves showing the effects on parasite growth over 2 complete erythrocytic cycles of control antimalarial compounds chloroquine (CQ) and artesunate, or peptidyl boronic acid compounds **1a** and **4c**. Calculated EC_50_ values (in nM for CQ and artesunate, in uM for the boronic acid compounds) and 95% confidence intervals (in parentheses) are shown. The 1AC5 parasite line, which expresses 8-10-fold less PfSUB1 than the B11 line, was ~13-fold more sensitive to inhibitor **4c** than the B11 line, whilst the sensitivities of the two lines to the other drugs were similar. All assays were performed in triplicate using different sources of blood in each case. Error bars, S.D.

*P. falciparum* proteasome (Pf20S) inhibitors have been shown to exert strong antimalarial effects ^35–37^ implying that non-selective peptidic boronic acid inhibitors designed to target PfSUB1 might in part produce parasite growth inhibition effects through (co)inhibition of Pf20S. To evaluate the on-target efficacy of the most selective new compound **4c** compared to our previously-characterized compound **1a**, a genetically modified *P. falciparum* line (called 1AC5) was generated that expresses 8-10-fold reduced levels of PfSUB1 compared to wild-type B11 parasites. Growth inhibition assays (Figure 3) showed that, as expected, both parasite lines 1AC5 and B11 displayed similar sensitivity to the antimalarial compounds chloroquine and artesunate which have established modes of action that do not involve direct inhibition of PfSUB1 or other enzymes of the egress cascade. The two parasite cell lines were also similarly sensitive to PfSUB1 inhibitor **1a** which displays low selectivity against H20S, pointing to off-target (possibly proteasome-inhibitory) effects in its mode of action. In contrast, the PfSUB1 knockdown line 1AC5 was consistently ~13-fold more sensitive to PfSUB1 inhibitor **4c** than its wild-type counterpart B11 line. These results provide strong evidence that compound **4c** exerts its antimalarial growth-inhibitory activity through direct inhibition of PfSUB1.

### Highly selective PfSUB1 inhibitors display low mammalian cell toxicity

**Figure 4:**
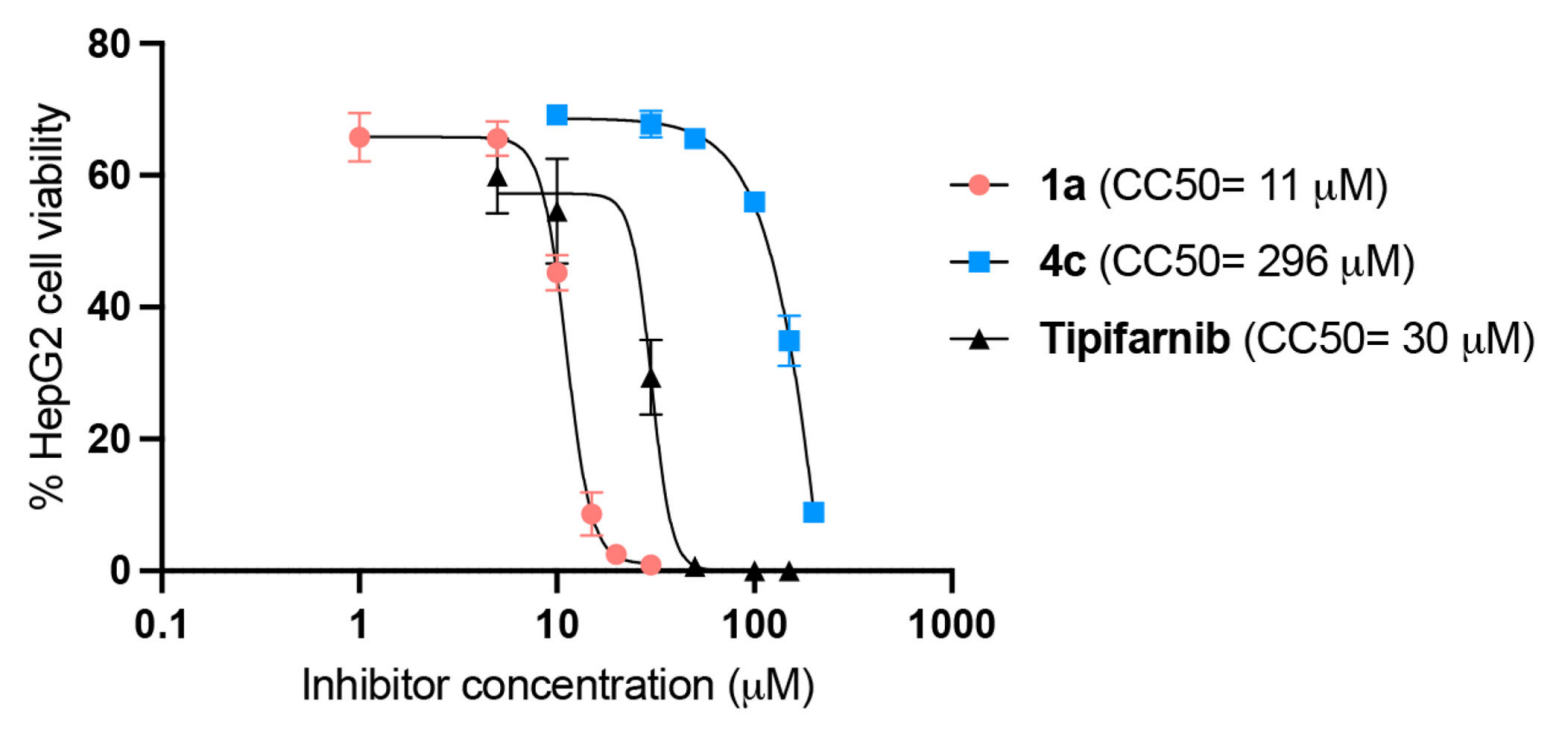
HepG2 cells cytotoxicity profile for compounds **1a** and **4c**. Tipifarnib, a FDA approved non-peptidomimetic quinolinone used in cancer therapy was used as a positive control for the CC50 calculations (concentration that reduced cell viability by 50%). Measurements were performed in triplicate. Error bars, S.D.

The cytotoxicity of the two inhibitors **1a** and **4c** was determined against the HepG2 cell line (Figure 4). Importantly, compound **4c** with reduced H20S inhibitory potency was less toxic than non-selective inhibitor **1a** by a factor of 27, consistent with toxicity being a consequence of H20S inhibition.

## Conclusion

P_3_ amino acid and *N*-capping groups of peptidic boronic acids PfSUB1 inhibitors can be modified to improve their lipophilicity. Particularly bulky and lipophilic *N*-capping groups were simple to install and were well tolerated in retaining PfSUB1 inhibitory potency. Off-target H20S inhibition by peptidic boronic acids was solved by substituting the methyl group P_1_ substituent by a carboxyethyl group, leading to boralactone derived PfSUB1 inhibitors with >1000 fold selectivity. Combination of lipophilic *N*-capping groups with the boralactone warhead reduced selectivity over H20S due to favorable interactions of these groups with the active site of H20S. However compound **4c** retained low nanomolar PfSUB1 inhibitory potency and >60 fold selectivity *versus* H20S. Importantly this compound also showed ~13 fold improved potency in inhibiting growth of a genetically modified parasite line (1AC5) expressing reduced levels of PfSUB1, compared to the wild-type parasite line (B11). In contrast, non-selective inhibitor **1a** had practically equal inhibitory potency against both lines. Moreover, selective inhibitor **4c** had 27-fold reduced HepG2 cell toxicity compared to inhibitor **1a**. Consequently boralactone containing peptidic inhibitors are new leads for anti-malarial drug discovery.

## Experimental section

Reagents and starting materials were obtained from commercial sources and used as received. The solvents were purified and dried by standard procedures prior to use. Flash chromatography was carried out using silica gel (230−400 mesh). Thin layer chromatography (TLC) was performed on silica gel and was visualized by UV lamp or staining with KMnO_4_. NMR spectra were recorded on 300 and 400 MHz spectrometers with chemical shift values (δ) in parts per million using residual chloroform, methanol or dimethyl sulfoxide signal as the internal standard. Conversion of starting material was detected with UPLC Waters Acquity, column: Acquity UPLC BEH-C18, 1.7 μm, 2.1 mm x 50 mm, column temperature (30.0±5.0) °C, gradient: 0.01% TFA in water/CH_3_CN 90%/10% − 5%/95%; flow: 0.500 mL/min; time: 8 min; detector: PDA, 220 − 320 nm, SQ detector with an electrospray ion source (ESI/APCI). Gas chromatographic (GC) analysis was performed on Agilent Technologies gas chromatographer with triple-axis detector, heating range 40 − 280 °C, column 30 m x 0.25 mm, 0.25 μm, 7 inch cage. Exact molecular masses (HRMS) were determined on a hybrid quadrupole time-of-flight mass spectrometer equipped with an electrospray ion source. For reversed phase column chromatography Biotage KP-C18-HS SNAP cartridge was used (gradient − water/CH_3_CN).

All compounds tested in biological assays are >95% pure by HPLC analysis.

## Chemistry

### General procedure A for amide bond formation

A mixture of amine (1.0 equiv), acid (1.0 equiv), HOBt (1.1 equiv), EDC·HCl (1.2 equiv) and DIPEA (3.0 equiv) were dissolved in DCM (or CHCl_3_) and stirred overnight at room temperature. The reaction mixture was washed with 1 M aqueous HCl (or 5% aqueous KHSO_4_) and brine. Organic phase was dried over Na_2_SO_4_, filtered and evaporated *in vacuo*. The residue was purified by flash chromatography on silica gel eluting with hexane:EtOAc mixture to provide the desired product.

### General procedure B for Boc deprotection

Starting material (1.0 equiv) was dissolved in DCM (or CHCl_3_) and treated with 4 M HCl in dioxane (4 equiv.). After a full conversion of the starting material (UPLC-MS control), the solvent was evaporated and the residue (based on a theoretical yield of a 100%) was utilized in the next step without purification.

### General procedure C for acylation

Under argon atmosphere starting material (1 equiv) was dissolved in DCM (or CHCl_3_), then anhydride (1.5 equiv) and DIPEA (3.0 equiv) were added. Reaction mixture was stirred at room temperature, then washed with 1 M aqueous HCl and brine. Organic phase was dried over Na_2_SO_4_, filtered and evaporated in vacuo. The crude mixture was purified by flash chromatography on silica gel eluting with Hexane:EtOAc − EtOAc to provide the desired product.

### General procedure D for hydrolysis

Starting material (1.0 equiv) was dissolved in THF:H_2_O (20:1) mixture, then LiOH (10 equiv) was added and the reaction was stirred at room temperature. When full conversion of starting material was observed, water was added and the reaction mixture was acidified by the addition of 1M HCl solution and the product was extracted with chloroform (3×). Organic phase was washed with brine, dried over Na_2_SO_4_, filtered and evaporated *in vacuo* to provide the desired product.

### General procedure E for amide bond formation

An acid (1.0 equiv) was dissolved in dry EtOAc, then *N*-methylmorpholine (3.0 equiv) and a solution of propylphosphonic acid anhydride (T3P, 2.0 equiv) was added sequentially under argon atmosphere. Reaction mixture was stirred for 30 min before α-aminoboronic acid derivative (1.2 equiv) dissolved in a small amount of DMF was added. After reaction was complete (UPLC-MS control) it was diluted with 5% citric acid solution in water. Layers were separated and the aqueous layer was extracted with EtOAc (2×). The combined organic layers were washed with sat. NaHCO_3_ and brine, dried over Na_2_SO_4_, filtered and concentrated under reduced pressure. Crude mixture was purified by flash chromatography on silica gel eluting with 0−5% MeOH in EtOAc to provide product.

### General procedure F for transesterification of boronic acid esters

A solution of boronic acid ester (1 equiv) in MeOH (or MeCN) and *n*-hexane (1:1) was treated with isobutylboronic acid (3−4 equiv) and 1 M HCl. After 18 h at room temperature, the methanolic phase was washed with *n*-hexane (2×) and the combined *n*-hexane layers were washed with MeOH (2×). The combined methanol phase was evaporated *in vacuo*. Crude mixture was purified by flash chromatography on reversed phase silica gel eluting with 10−100% MeCN in H_2_O to provide desired compound.

### General procedure G for amide bond formation

Based on the synthesis of bortezomib^1^: Under argon atmosphere an acid (1 equiv) was mixed with α-aminoboronic acid derivative (1.2 equiv) and DMAP (0.3 equiv) in anhydrous CHCl_3_ at room temperature. The white suspension was cooled to −15°C and then *N*-methylmorpholine (3−4 equiv) was added while the internal temperature was kept below −10°C. T3P reagent (1.5−2 equiv) was added at the same temperature. The reaction mixture was stirred allowed to warm up from −10 to −15°C to room temperature overnight. It was then diluted with CHCl_3_ and equal amount of 5% KHSO_4_ and extracted. Organic phase was extracted once more with 5% KHSO_4_. Combined water phase was back-extracted with CHCl_3_. Organic phase was washed with brine and dried over Na_2_SO_4_, filtered and concentrated under reduced pressure. Crude mixture was purified by flash chromatography on silica gel eluting with 0−5% MeOH in EtOAc to provide the desired compound.

#### Ethyl (S)-(2-((tert-butoxycarbonyl)amino)-3,3-dimethylbutanoyl)glycinate (8a)

Prepared according to the general procedure A from glycine ethyl ester hydrochloride **7** (243 mg, 1.74 mmol), *N*-Boc-L*-tert*-leucine **6a** (402 mg, 1.74 mmol), HOBt (258 mg, 1.91 mmol), EDC·HCl (400 mg, 2.09 mmol) and DIPEA (0.90 mL, 5.20 mmol) in DCM (30 mL). The product was purified by flash chromatography on silica gel eluting with hexane:EtOAc (4:1 − 2:1) to provide **8a** (445 mg, 81%) as a white solid compound.

^1^H NMR (400 MHz, Chloroform-*d*) δ 6.46 (t, *J* = 5.4 Hz, 1H), 5.27 (s, 1H), 4.25 − 4.10 (m, 3H), 3.97 − 3.82 (m, 2H), 1.42 (s, 9H), 1.27 (t, *J* = 7.2 Hz, 3H), 1.01 (s, 9H). ^13^C NMR (101 MHz, Chloroform-*d*) δ 171.3, 169.7, 155.9, 79.8, 62.4, 61.6, 41.4, 34.7, 28.4, 26.7, 14.3. HR-MS (ESI/TOF) calcd for C_15_H_28_N_2_O_5_Na [M+Na]^+^ 339.1896, found 339.1913.

#### Ethyl (S)-(2-((tert-butoxycarbonyl)amino)-2-phenylacetyl)glycinate (8b)

Prepared according to the general procedure A from glycine ethyl ester hydrochloride **7** (248 mg, 1.78 mmol), *N*-Boc-L-phenylglycine **6b** (447 mg, 1.78 mmol), HOBt (264 mg, 1.95 mmol), EDC·HCl (410 mg, 2.14 mmol) and DIPEA (0.92 mL, 5.32 mmol) in DCM (30 mL). The product was purified by flash chromatography on silica gel eluting with hexane:EtOAc (4:1 − 2:1) to provide **8b** (354 mg, 59%) as a white solid compound.

^1^H NMR (400 MHz, Chloroform-*d*) δ 7.45 − 7.28 (m, 5H), 6.33 (t, *J* = 5.3 Hz, 1H), 5.72 (s, 1H), 5.20 (s, 1H), 4.17 (q, *J* = 7.2 Hz, 2H), 4.05 (dd, *J* = 18.3, 5.3 Hz, 1H), 3.95 (dd, *J* = 18.4, 5.1 Hz, 1H), 1.41 (s, 9H), 1.24 (t, *J* = 7.2 Hz, 3H). ^13^C NMR (101 MHz, Chloroform-*d*) δ 170.4, 169.5, 155.2, 138.2, 129.2, 128.6, 127.5, 80.3, 61.8, 58.8, 41.7, 28.4, 14.2. HR-MS (ESI/TOF) calcd for C_17_H_24_N_2_O_5_Na [M+Na]^+^ 359.1583, found 359.1590.

#### Ethyl (S)-(2-((tert-butoxycarbonyl)amino)-3-cyclopentylpropanoyl)glycinate (8c)

Prepared according to the general procedure A from glycine ethyl ester hydrochloride **7** (206 mg, 1.48 mmol), *N*-Boc-L-cyclopentylalanine **6c** (380 mg, 1.48 mmol), HOBt (220 mg, 1.63 mmol), EDC·HCl (340 mg, 1.77 mmol), and DIPEA (0.78 mL, 4.51 mmol) in DCM (30 mL). The product was purified by flash chromatography on silica gel eluting with hexane:EtOAc (4:1 − 2:1) to provide **8c** (447 mg, 88%) as a white solid compound.

^1^H NMR (400 MHz, Chloroform-*d*) δ 6.59 (s, 1H), 4.91 (s, 1H), 4.21 (q, *J* = 7.2 Hz, 2H), 4.16 − 4.07 (m, 1H), 4.02 (d, *J* = 5.2 Hz, 2H), 1.92 − 1.73 (m, 4H), 1.65 − 1.56 (m, 3H), 1.56 − 1.47 (m, 2H), 1.44 (s, 9H), 1.28 (t, *J* = 7.2 Hz, 3H), 1.19 − 1.07 (m, 2H).^13^C NMR (101 MHz, Chloroform-*d*) δ 172.6, 169.7, 155.7, 80.2, 61.5, 54.2, 41.3, 38.6, 36.6, 32.8, 32.5, 28.3, 25.2, 25.0, 14.1.HR-MS (ESI/TOF) calcd for C_17_H_30_N_2_O_5_Na [M+Na]^+^ 365.2052, found 365.2059.

#### Ethyl (S)-(2-((tert-butoxycarbonyl)amino)-3-cyclohexylpropanoyl)glycinate (8d)

Prepared according to the general procedure A from glycine ethyl ester hydrochloride **7** (250 mg, 1.79 mmol), *N*-Boc-L-cyclohexylalanine **6d** (486 mg, 1.79 mmol), HOBt (266 mg, 1.97 mmol), EDC·HCl (412 mg, 2.15 mmol) and DIPEA (0.93 mL, 5.38 mmol) in DCM (35 mL). The product was purified by flash chromatography on silica gel eluting with hexane:EtOAc (4:1 − 2:1) to provide **8d** (549 mg, 86%) as a white solid compound.

^1^H NMR (400 MHz, Chloroform-*d*) δ 6.59 (s, 1H), 4.83 (s, 1H), 4.30 − 4.16 (m, 3H), 4.02 (d, *J* = 5.2 Hz, 2H), 1.84 − 1.62 (m, 6H), 1.54 − 1.41 (m, 10H), 1.40 − 1.32 (m, 1H), 1.28 (t, *J* = 7.2 Hz, 3H), 1.25 − 1.07 (m, 3H), 1.04 − 0.82 (m, 2H). ^13^C NMR (101 MHz, Chloroform-*d*) δ 173.0, 169.8, 155.9, 80.4, 61.7, 52.4, 41.5, 40.0, 34.2, 33.8, 32.6, 28.4, 26.5, 26.4, 26.2, 14.3. HR-MS (ESI/TOF) calcd for C_18_H_32_N_2_O_5_Na [M+Na]^+^ 379.2209, found 379.2212.

#### Ethyl (S)-(2-((tert-butoxycarbonyl)amino)-4,4-dimethylpentanoyl)glycinate (8e)

Prepared according to the general procedure A from glycine ethyl ester hydrochloride **7** (204 mg, 1.45 mmol), *N*-Boc-L*-tert*-butylalanine **6e** (354 mg, 1.44 mmol), HOBt (215 mg, 1.59 mmol), EDC·HCl (332 mg, 1.73 mmol), and DIPEA (0.75 mL, 4.33 mmol) in DCM (25 mL). The product was purified by flash chromatography on silica gel eluting with hexane:EtOAc (4:1 − 2:1) to provide **8e** (455 mg, 95%) as a white solid compound.

^1^H NMR (400 MHz, Chloroform-*d*) δ 6.72 (t, *J* = 5.5 Hz, 1H), 4.85 (d, *J* = 8.5 Hz, 1H), 4.27 − 4.14 (m, 3H), 4.00 (dd, *J* = 5.4, 1.7 Hz, 2H), 1.92 (dd, *J* = 14.5, 3.6 Hz, 1H), 1.44 (s, 9H), 1.39 (dd, *J* = 14.5, 8.9 Hz, 1H), 1.27 (t, *J* = 7.2 Hz, 3H), 0.96 (s, 9H).^13^C NMR (101 MHz, Chloroform-*d*) δ 173.3, 169.8, 155.6, 80.4, 61.6, 52.3, 45.7, 41.5, 30.6, 29.8, 28.5, 14.3. HR-MS (ESI/TOF) calcd for C_16_H_30_N_2_O_5_Na [M+Na]^+^ 353.2052, found 353.2057.

#### Ethyl (tert-butoxycarbonyl)-L-isoleucylglycinate (8f)

Prepared according to the general procedure A from glycine ethyl ester hydrochloride **7** (200 mg, 1.42 mmol), *N*-Boc-L-isoleucine **6f** (330 mg, 1.43 mmol), HOBt (211 mg, 1.56 mmol), EDC·HCl (326 mg, 1.70 mmol) and DIPEA (0.74 mL, 4.28 mmol) in DCM (30 mL). The product was purified by flash chromatography on silica gel eluting with hexane:EtOAc (4:1 − 2:1) to provide **8f** (412 mg, 92%) as a white solid compound.

^1^H NMR (400 MHz, Chloroform-*d*) δ 6.54 (t, *J* = 5.3 Hz, 1H), 5.05 (d, *J* = 8.2 Hz, 1H), 4.20 (q, *J* = 7.2 Hz, 2H), 4.12 − 3.92 (m, 3H), 1.96 − 1.86 (m, 1H), 1.56 − 1.46 (m, 1H), 1.43 (s, 9H), 1.27 (t, *J* = 7.2 Hz, 3H), 1.20 − 1.06 (m, 1H), 0.94 (d, *J* = 6.9 Hz, 3H), 0.91 (t, *J* = 7.4 Hz, 3H).

^13^C NMR (101 MHz, Chloroform-*d*) δ 172.0, 169.8, 155.9, 80.1, 61.7, 59.3, 41.4, 37.4, 28.4, 24.8, 15.7, 14.3, 11.6. HR-MS (ESI/TOF) calcd for C_15_H_28_N_2_O_5_Na [M+Na]^+^ 339.1896, found 339.1911.

#### Ethyl (tert-butoxycarbonyl)-L-leucylglycinate (8g)

Prepared according to the general procedure A: glycine ethyl ester hydrochloride **7** (200 mg, 1.42 mmol), *N*-Boc-L- leucine **6g** (328 mg, 1.42 mmol), HOBt (211 mg, 1.56 mmol), EDC·HCl (326 mg, 1.70 mmol) and DIPEA (0.74 mL, 4.28 mmol) in DCM (30 mL). The product was purified by flash chromatography on silica gel eluting with hexane:EtOAc (4:1 − 2:1) to provide **8g** (297 mg, 66%) as a white solid compound.

^1^H NMR (400 MHz, Chloroform-*d*) δ 6.66 (t, *J* = 5.5 Hz, 1H), 4.91 (d, *J* = 7.4 Hz, 1H), 4.24 − 4.12 (m, 3H), 4.01 (dd, *J* = 5.3, 1.4 Hz, 2H), 1.74 − 1.64 (m, 2H), 1.48 (dd, *J* = 9.5, 8.2 Hz, 1H), 1.44 (s, 9H), 1.27 (t, *J* = 7.2 Hz, 3H), 0.94 (d, *J* = 4.2 Hz, 3H), 0.93 (d, *J* = 3.9 Hz, 3H). ^13^C NMR (101 MHz, Chloroform-*d*) δ 173.0, 169.8, 155.8, 80.3, 61.6, 53.1, 41.4, 28.4, 24.9, 23.1, 22.0, 14.3. HR-MS (ESI/TOF) calcd for C_15_H_28_N_2_O_5_Na [M+Na]^+^ 339.1896, found 339.1906.

#### Ethyl (tert-butoxycarbonyl)-L-phenylalanylglycinate (8h)

Prepared according to the general procedure A from glycine ethyl ester hydrochloride **7** (200 mg, 1.42 mmol), *N*-Boc-L-phenylalanine **6h** (376 mg, 1.42 mmol), HOBt (211 mg, 1.56 mmol), EDC·HCl (326 mg, 1.70 mmol), and DIPEA (0.74 mL, 4.28 mmol) in DCM (30 mL). The residue was purified by flash chromatography on silica gel eluting with hexane:EtOAc (4:1 − 2:1) to provide **8h** (394 mg, 79%) as a white solid compound.

^1^H NMR (400 MHz, Chloroform-*d*) δ 7.33 − 7.27 (m, 2H), 7.26 − 7.19 (m, 3H), 6.47 − 6.40 (m, 1H), 5.01 (s, 1H), 4.46 − 4.33 (m, 1H), 4.19 (q, *J* = 7.1 Hz, 2H), 4.02 (dd, *J* = 18.3, 5.4 Hz, 1H), 3.91 (dd, *J* = 18.4, 5.0 Hz, 1H), 3.16 − 3.00 (m, 2H), 1.39 (s, 9H), 1.26 (t, *J* = 7.2 Hz, 3H). ^13^C NMR (101 MHz, Chloroform-*d*) δ 171.6, 169.5, 155.5, 136.7, 129.4, 128.8, 127.1, 80.4, 61.7, 55.8, 41.5, 38.5, 28.4, 14.3. HR-MS (ESI/TOF) calcd for C_18_H_26_N_2_O_5_Na [M+Na]^+^ 373.1739, found 373.1740

#### Ethyl ((S)-2-((S)-2-((tert-butoxycarbonyl)amino)-2-cyclopentylacetamido)-3,3-dimethylbutano-yl)glycinate (10a)

Deprotection was performed according general procedure B from starting material **8a** (435 mg, 1.37 mmol) and 4 M HCl in dioxane (1.4 mL) in DCM (10 mL). After a full conversion of the starting material, the intermediate was subjected to the coupling reaction according to general procedure A using *N*-Boc-cyclopentyl-Gly-OH **9** (334 mg, 1.37 mmol), EDC·HCl (316 mg, 1.65 mmol), HOBt (204 mg, 1.51 mmol) and DIPEA (0.72 mL, 4.16 mmol) in DCM (40 mL). The product was purified by flash chromatography on silica gel eluting with hexane:EtOAc (2:1 − 1:1) to provide **10a** (532 mg, 88%) as a white solid compound.

^1^H NMR (400 MHz, Chloroform-*d*) δ 6.88 − 6.74 (m, 2H), 5.21 (d, *J* = 8.1 Hz, 1H), 4.36 (d, *J* = 9.2 Hz, 1H), 4.25 − 4.08 (m, 3H), 3.94 (t, *J* = 8.9 Hz, 1H), 3.86 (dd, *J* = 18.2, 4.7 Hz, 1H), 2.21 (h, *J* = 8.5 Hz, 1H), 1.78 − 1.46 (m, 6H), 1.42 (s, 9H), 1.36 − 1.20 (m, 5H), 1.01 (s, 9H). ^13^C NMR (101 MHz, Chloroform-*d*) δ 172.3, 170.6, 169.7, 156.1, 80.0, 61.6, 60.6, 59.1, 41.9, 41.4, 34.8, 29.6, 29.0, 28.4, 26.7, 25.5, 25.2, 14.36. HR-MS (ESI/TOF) calcd for C_22_H_39_N_3_O_6_Na [M+Na]^+^ 464.2737, found 464.2742.

#### Ethyl ((S)-2-((S)-2-((tert-butoxycarbonyl)amino)-2-cyclopentylacetamido)-2-phenylacetyl) glycinate (10b)

Deprotection was performed according to general procedure B from starting material **8b** (340 mg, 1.01 mmol) and 4 M HCl in dioxane (1.0 mL) in DCM (5 mL). After a full conversion of the starting material, the intermediate was subjected to the coupling reaction according to general procedure A using *N*-Boc-cyclopentyl-Gly-OH **9** (246 mg, 1.01 mmol), EDC·HCl (233 mg, 1.22 mmol), HOBt (150 mg, 1.11 mmol), and DIPEA (0.52 mL, 3.01 mmol) in DCM (40 mL). The product was purified by flash chromatography on silica gel eluting with hexane:EtOAc (2:1 − 1:1) to provide **10b** (331 mg, 71%) as a white solid compound.

^1^H NMR (400 MHz, Chloroform-*d*) δ 7.42 − 7.28 (m, 5H), 7.22 (d, *J* = 6.8 Hz, 1H), 6.73 (s, 1H), 5.55 (d, *J* = 7.0 Hz, 1H), 5.12 (d, *J* = 7.4 Hz, 1H), 4.16 (q, *J* = 7.2 Hz, 2H), 4.10 − 3.89 (m, 3H), 2.21 (h, *J* = 6.9, 6.2 Hz, 1H), 1.74 − 1.46 (m, 6H), 1.40 (s, 9H), 1.36 − 1.27 (m, 2H), 1.23 (t, *J* = 7.2 Hz, 3H). ^13^C NMR (101 MHz, Chloroform-*d*) δ 171.7, 170.0, 169.5, 156.1, 137.5, 129.1, 128.6, 127.5, 80.2, 61.7, 58.5, 57.3, 42.3, 41.7, 29.5, 28.7, 28.4, 25.5, 25.2, 14.2. HR-MS (ESI/TOF) calcd for C_22_H_39_N_3_O_6_Na [M+Na]^+^ 464.2424, found 484.2433.

#### Ethyl ((S)-2-((S)-2-((tert-butoxycarbonyl)amino)-2-cyclopentylacetamido)-3-cyclo-pentylpropanoyl)glycinate (10c)

Deprotection was performed according to general procedure B from starting material **8c** (416 mg, 1.21 mmol) and 4 M HCl in dioxane (1.2 mL) in DCM (10 mL). After a full conversion of the starting material, the intermediate was subjected to the coupling reaction according to general procedure A using *N*-Boc-cyclopentyl-Gly-OH **9** (296 mg, 1.21 mmol), EDC·HCl (280 mg, 1.46 mmol), HOBt (181 mg, 1.34 mmol), and DIPEA (0.64 mL, 3.70 mmol) in DCM (30 mL). The product was purified by flash chromatography on silica gel eluting with hexane:EtOAc (2:1 − 1:1) to provide **10c** (421 mg, 74%) as a white solid compound.

^1^H NMR (400 MHz, Chloroform-*d*) δ 6.90 (t, *J* = 5.5 Hz, 1H), 6.62 (d, *J* = 8.1 Hz, 1H), 5.12 (d, *J* = 8.0 Hz, 1H), 4.48 (td, *J* = 8.5, 5.3 Hz, 1H), 4.18 (q, *J* = 7.2 Hz, 2H), 4.02 − 3.96 (m, 2H), 3.91 (t, *J* = 7.9 Hz, 1H), 2.22 (h, *J* = 8.6 Hz, 1H), 1.93 − 1.63 (m, 7H), 1.65 − 1.46 (m, 8H), 1.42 (s, 9H), 1.36 − 1.22 (m, 5H), 1.19 − 1.04 (m, 2H). ^13^C NMR (101 MHz, Chloroform-*d*) δ 172.3, 172.1, 169.7, 156.2, 80.3, 61.5, 59.0, 52.8, 42.0, 41.5, 38.2, 36.6, 32.9, 32.5, 29.5, 28.9, 28.4, 25.5, 25.3, 25.2, 25.1, 14.3. HR-MS (ESI/TOF) calcd for C_24_H_42_N_3_O_6_ [M+H]^+^ 468.3074, found 468.3077.

#### Ethyl ((S)-2-((S)-2-((tert-butoxycarbonyl)amino)-2-cyclopentylacetamido)-3-cyclo-hexylpropanoyl)glycinate (10d)

Deprotection was performed according to general procedure B from starting material **8d** (530 mg, 1.49 mmol) and 4 M HCl in dioxane (1.5 mL) in DCM (10 mL). After a full conversion of the starting material, the intermediate was dissolved in DCM (30 mL), *N*-Boc-cyclopentyl-Gly-OH **9** (362 mg, 1.49 mmol), EDC·HCl (342 mg, 1.78 mmol), HOBt (222 mg, 1.64 mmol) and DIPEA (0.78 mL, 4.51 mmol) were added and mixture was stirred overnight at room temperature. The residue was purified by flash chromatography on silica gel eluting with hexane:EtOAc (2:1 − 1:1) to provide **10d** (496 mg, 69%) as a white solid compound.

^1^H NMR (400 MHz, Chloroform-*d*) δ 6.90 (s, 1H), 6.51 (d, *J* = 8.3 Hz, 1H), 5.07 (d, *J* = 7.0 Hz, 1H), 4.55 (ddd, *J* = 9.5, 8.3, 5.4 Hz, 1H), 4.18 (q, *J* = 7.2 Hz, 2H), 3.97 (d, *J* = 5.5 Hz, 2H), 3.90 (t, *J* = 7.7 Hz, 1H), 2.22 (h, *J* = 8.2, 7.7 Hz, 1H), 1.83 − 1.48 (m, 14H), 1.43 (s, 9H), 1.37 − 1.23 (m, 6H), 1.21 − 1.07 (m, 2H), 1.03 − 0.81 (m, 2H). ^13^C NMR (101 MHz, Chloroform-*d*) δ 172.4, 172.3, 169.7, 156.2, 80.4, 61.5, 59.1, 50.8, 42.1, 41.5, 39.3, 34.1, 33.8, 32.5, 29.5, 28.9, 28.4, 26.5, 26.3, 26.1, 25.5, 25.2, 14.3. HR-MS (ESI/TOF) calcd for C_25_H_44_N_3_O_6_ [M+H]^+^ 482.3230, found 482.3230.

#### Ethyl ((S)-2-((S)-2-((tert-butoxycarbonyl)amino)-2-cyclopentylacetamido)-4,4-di-methylpentanoyl)glycinate (10e)

Deprotection was performed according to general procedure B from starting material **8e** (440 mg, 1.33 mmol, 1.1 equiv) and 4 M HCl in dioxane (1.4 mL) in DCM (20 mL). After a full conversion of the starting material, the intermediate was subjected to the coupling reaction according to general procedure A using *N*-Boc-cyclopentyl-Gly-OH **9** (294 mg, 1.21 mmol), EDC·HCl (300 mg, 1.56 mmol), HOBt (200 mg, 1.48 mmol), and DIPEA (0.70 mL, 4.05 mmol) in DCM (30 mL). The product was purified by flash chromatography on silica gel eluting with hexane:EtOAc (2:1 − 1:1) to provide **10e** (405 mg, 74%) as a white solid compound.

^1^H NMR (400 MHz, Chloroform-*d*) δ 7.07 − 7.00 (m, 1H), 6.73 (d, *J* = 8.3 Hz, 1H), 5.19 (d, *J* = 7.9 Hz, 1H), 4.53 (td, *J* = 8.4, 3.7 Hz, 1H), 4.17 (q, *J* = 7.2 Hz, 2H), 4.03 − 3.87 (m, 3H), 2.22 (h, *J* = 8.8 Hz, 1H), 1.97 (dd, *J* = 14.5, 3.8 Hz, 1H), 1.78 − 1.50 (m, 6H), 1.47 (dd, *J* = 14.5, 8.4 Hz, 1H), 1.41 (s, 9H), 1.34 − 1.22 (m, 5H), 0.93 (s, 9H). ^13^C NMR (101 MHz, Chloroform-*d*) δ 172.7, 172.2, 169.7, 156.2, 80.3, 61.5, 59.2, 50.7, 45.4, 41.9, 41.5, 30.6, 29.7, 29.5, 29.1, 28.4, 25.5, 25.2, 14.3. HR-MS (ESI/TOF) calcd for C_23_H_41_N_3_O_6_Na [M+Na]^+^ 478.2893, found 478.2900.

#### Ethyl ((S)-2-((tert-butoxycarbonyl)amino)-2-cyclopentylacetyl)-L-isoleucylglycinate (10f)

Deprotection was performed according to according general procedure B from starting material **8f** (400 mg, 1.26 mmol) and 4 M HCl in dioxane (1.3 mL) in DCM (20 mL). After a full conversion of the starting material, the intermediate was subjected to the coupling reaction according to general procedure A using the residue *N*-Boc-cyclopentyl-Gly-OH **9** (308 mg, 1.27 mmol), EDC·HCl (291 mg, 1.52 mmol), HOBt (188 mg, 1.39 mmol) and DIPEA (0.66 mL, 3.81 mmol) in DCM (40 mL). The residue was purified by flash chromatography on silica gel eluting with hexane:EtOAc (2:1 − 1:1) to provide **10f** (471 mg, 84%) as a white solid compound.

^1^H NMR (400 MHz, Chloroform-*d*) δ 6.91 − 6.82 (m, 1H), 6.69 (d, *J* = 8.8 Hz, 1H), 5.13 (d, *J* = 6.9 Hz, 1H), 4.38 (dd, *J* = 8.8, 6.3 Hz, 1H), 4.18 (q, *J* = 7.2 Hz, 2H), 4.08 − 3.87 (m, 3H), 2.24 (h, *J* = 8.5 Hz, 1H), 2.03 − 1.93 (m, 1H), 1.78 − 1.66 (m, 2H), 1.65 − 1.47 (m, 5H), 1.42 (s, 9H), 1.36 − 1.23 (m, 5H), 1.19 − 1.05 (m, 1H), 0.93 (d, *J* = 6.8 Hz, 3H), 0.89 (t, *J* = 7.4 Hz, 3H). ^13^C NMR (101 MHz, Chloroform-*d*) δ 172.3, 171.4, 169.7, 156.2 80.4, 61.6, 59.2, 57.8, 41.8, 41.4, 36.9, 29.6, 28.9, 28.4, 25.5, 25.3, 24.7, 15.6, 14.3, 11.5. HR-MS (ESI/TOF) calcd for C_22_H_39_N_3_O_6_Na [M+Na]^+^ 464.2737, found 464.2758.

#### Ethyl ((S)-2-((tert-butoxycarbonyl)amino)-2-cyclopentylacetyl)-L-leucylglycinate (10g)

Deprotection was performed according to general procedure B from starting material **8g** (287 mg, 0.90 mmol) and 4 M HCl in dioxane (0.92 mL) in DCM (10 mL). After a full conversion of the starting material, the intermediate was subjected to the coupling reaction according to general procedure A using *N*-Boc-cyclopentyl-Gly-OH **9** (220 mg, 0.90 mmol), EDC·HCl (208 mg, 1.08 mmol), HOBt (135 mg, 1.00 mmol) and DIPEA (0.47 mL, 2.72 mmol) in DCM (40 mL). The residue was purified by flash chromatography on silica gel eluting with hexane:EtOAc (2:1 − 1:1) to provide **10g** (313 mg, 78%) as a white solid compound.

^1^H NMR (400 MHz, Chloroform-*d*) δ 6.91 (t, *J* = 5.8 Hz, 1H), 6.57 (d, *J* = 8.2 Hz, 1H), 5.10 (d, *J* = 8.1 Hz, 1H), 4.52 (ddd, *J* = 9.5, 8.2, 5.2 Hz, 1H), 4.18 (q, *J* = 7.2 Hz, 2H), 3.97 (d, *J* = 5.4 Hz, 2H), 3.89 (t, *J* = 8.0 Hz, 1H), 2.23 (h, *J* = 8.6 Hz, 1H), 1.79 − 1.48 (m, 9H), 1.42 (s, 9H), 1.26 (t, *J* = 7.2 Hz, 5H), 0.92 (d, *J* = 6.4 Hz, 3H), 0.90 (d, *J* = 6.2 Hz, 3H). ^13^C NMR (101 MHz, Chloroform-*d*) δ 172.4, 172.2, 169.7, 156.2, 80.4, 61.5, 59.1, 51.6, 41.9, 41.4, 40.8, 29.5, 28.9, 28.4, 25.5, 25.3, 24.8, 23.1, 21.9, 14.3. HR-MS (ESI/TOF) calcd for C_22_H_39_N_3_O_6_Na [M+Na]^+^ 464.2737, found 464.2741.

#### Ethyl ((S)-2-((tert-butoxycarbonyl)amino)-2-cyclopentylacetyl)-L-phenylalanyl-glycinate (10h)

Deprotection was performed according to general procedure B from starting material **8h** (377 mg, 1.08 mmol) and 4M HCl in dioxane (1.1 mL) in DCM (20 mL). After a full conversion of the starting material, the intermediate was subjected to the coupling reaction according to general procedure A using *N*-Boc-cyclopentyl-Gly-OH **9** (262 mg, 1.08 mmol), EDC·HCl (248 mg, 1.30 mmol), HOBt (160 mg, 1.18 mmol). and DIPEA (0.56 mL, 3.24 mmol) in DCM (40 mL). The product was purified by flash chromatography on silica gel eluting with hexane:EtOAc (2:1 − 1:1) to provide **10h** (391 mg, 76%) as a white solid compound.

^1^H NMR (400 MHz, Chloroform-*d*) δ 7.33 − 7.25 (m, 2H), 7.24 − 7.19 (m, 3H), 6.76 (s, 1H), 6.54 (d, *J* = 8.2 Hz, 1H), 4.91 (d, *J* = 6.2 Hz, 1H), 4.76 (q, *J* = 7.0 Hz, 1H), 4.17 (q, *J* = 7.2 Hz, 2H), 4.00 (dd, *J* = 17.9, 5.5 Hz, 1H), 3.91 − 3.81 (m, 2H), 3.18 (dd, *J* = 14.0, 6.6 Hz, 1H), 3.08 (dd, *J* = 14.0, 7.3 Hz, 1H), 2.13 − 2.02 (m, 1H), 1.65 − 1.43 (m, 6H), 1.40 (s, 9H), 1.25 (t, *J* = 7.2 Hz, 3H), 1.23 − 1.12 (m, 2H). ^13^C NMR (101 MHz, Chloroform-*d*) δ 172.0, 171.1, 169.4, 156.2, 136.7, 129.4, 128.8, 127.1, 80.6, 61.5, 59.2, 54.0, 42.0, 41.5, 37.9, 29.3, 28.6, 28.4, 25.4, 25.1, 14.3. HR-MS (ESI/TOF) calcd for C_25_H_37_N_3_O_6_Na [M+Na]^+^ 498.2580, found 498.2580.

#### Ethyl ((S)-2-((S)-2-acetamido-2-cyclopentylacetamido)-3,3-dimethylbutanoyl)glycin-ate (11a)

Deprotection was performed according to general procedure B from starting material **10a** (507 mg, 1.15 mmol) and 4M HCl in dioxane (1.2 mL) in DCM (20 mL). After a full conversion of the starting material, the intermediate was subjected to the acylation reaction according to general procedure C with acetic anhydride (160 μL, 1.70 mmol) and DIPEA (600 μL, 3.47 mmol) in DCM (30 mL),. The crude mixture was purified by flash chromatography on silica gel eluting with Hexane:EtOAc (1:1) − EtOAc to provide **11a** (379 mg, 86%) as a white solid compound.

^1^H NMR (400 MHz, Chloroform-*d*) δ 7.31 − 7.27 (m, 1H), 7.18 (d, *J* = 9.4 Hz, 1H), 6.80 (d, *J* = 8.8 Hz, 1H), 4.51 (t, *J* = 8.9 Hz, 1H), 4.49 (d, *J* = 9.3 Hz, 1H), 4.24 − 4.15 (m, 3H), 3.82 (dd, *J* = 18.2, 4.5 Hz, 1H), 2.15 (h, *J* = 8.9 Hz, 1H), 2.00 (s, 3H), 1.75 − 1.44 (m, 6H), 1.27 (t, *J* = 7.2 Hz, 5H), 1.00 (s, 9H). ^13^C NMR (101 MHz, Chloroform-*d*) δ 172.2, 170.8, 170.4, 170.0, 61.6, 60.7, 57.2, 43.0, 41.4, 34.6, 29.4, 29.3, 26.7, 25.5, 25.0, 23.2, 14.3. HR-MS (ESI/TOF) calcd for C_19_H_33_N_3_O_5_Na [M+Na]^+^ 406.2318, found 406.2325

#### Ethyl ((S)-2-((S)-2-acetamido-2-cyclopentylacetamido)-2-phenylacetyl)glycinate (11b)

Deprotection was performed according to general procedure B from starting material **10b** (315 mg, 0.68 mmol) and 4M HCl in dioxane (0.70 mL) in DCM (10 mL). After a full conversion of the starting material, the intermediate was subjected to the acylation reaction according to general procedure C with acetic anhydride (100 μL, 1.06 mmol) and DIPEA (360 μL, 2.08 mmol) in DCM (15 mL). The crude mixture was purified by flash chromatography on silica gel eluting with hexane:EtOAc (1:1) − EtOAc to provide **11b** (210 mg, 76%) as a white solid compound.

^1^H NMR (400 MHz, Dimethylsulfoxide-*d_6_*) δ 8.68 (t, *J* = 5.9 Hz, 1H), 8.39 (d, *J* = 8.2 Hz, 1H), 8.01 (d, *J* = 8.5 Hz, 1H), 7.44 − 7.39 (m, 2H), 7.37 − 7.24 (m, 3H), 5.53 (d, *J* = 8.1 Hz, 1H), 4.28 (t, *J* = 8.5 Hz, 1H), 4.04 (q, *J* = 7.1 Hz, 2H), 3.92 − 3.77 (m, 2H), 2.12 (h, *J* = 8.6 Hz, 1H), 1.83 (s, 3H), 1.68 − 1.37 (m, 6H), 1.35 − 1.18 (m, 2H), 1.13 (t, *J* = 7.1 Hz, 3H). ^13^C NMR (101 MHz, Dimethylsulfoxide-*d_6_*) δ 171.1, 170.2, 169.5, 169.3, 138.4, 128.2, 127.6, 127.2, 60.5, 56.0, 55.7, 41.8, 40.9, 28.7, 28.6, 24.9, 24.6, 22.5, 14.0. HR-MS (ESI/TOF) calcd for C_21_H_29_N_3_O_5_Na [M+Na]^+^ 426.2005, found 426.2019

#### Ethyl ((S)-2-((S)-2-acetamido-2-cyclopentylacetamido)-3-cyclopentylpropanoyl) glycinate (11c)

Deprotection was performed according to general procedure B from starting material **10c** (404 mg, 0.86 mmol) and 4M HCl in dioxane (0.90 mL) in DCM (20 mL). After a full conversion of the starting material, the intermediate was subjected to the acylation reaction according to general procedure C with acetic anhydride (120 μL, 1.27 mmol), and DIPEA (460 μL, 2.66 mmol) in DCM (30 mL). The crude mixture was purified by flash chromatography on silica gel eluting with hexane:EtOAc (1:1) − EtOAc to provide **11c** (288 mg, 81%) as a white solid compound.

^1^H NMR (400 MHz, Dimethylsulfoxide-*d_6_*) δ 8.27 (t, *J* = 5.9 Hz, 1H), 7.94 (d, *J* = 8.5 Hz, 1H), 7.88 (d, *J* = 8.2 Hz, 1H), 4.28 (td, *J* = 8.9, 5.7 Hz, 1H), 4.16 (t, *J* = 8.5 Hz, 1H), 4.07 (q, *J* = 7.2 Hz, 2H), 3.85 (dd, *J* = 17.3, 6.0 Hz, 1H), 3.75 (dd, *J* = 17.3, 5.8 Hz, 1H), 2.11 (h, *J* = 8.5 Hz, 1H), 1.84 (s, 3H), 1.83 − 1.68 (m, 2H), 1.68 − 1.48 (m, 9H), 1.48 − 1.40 (m, 4H), 1.34 − 1.14 (m, 5H), 1.14 − 1.00 (m, 2H). ^13^C NMR (101 MHz, Dimethylsulfoxide-*d_6_*) δ 172.3, 171.2, 169.6, 169.2, 60.4, 56.1, 51.8, 41.8, 40.7, 38.2, 36.0, 32.3, 31.7, 28.6, 28.5, 24.9, 24.8, 24.6, 24.5, 22.5, 14.0. HR-MS (ESI/TOF) calcd for C_21_H_36_N_3_O_5_ [M+H]^+^ 410.2655, found 410.2654.

#### Ethyl ((S)-2-((S)-2-acetamido-2-cyclopentylacetamido)-3-cyclohexylpropanoyl)-glycinate (11d)

Deprotection was performed according to general procedure B from starting material **10d** (480 mg, 1.00 mmol) and 4M HCl in dioxane (1 mL) in DCM (20 mL). After a full conversion of the starting material, the intermediate was subjected to the acylation reaction according to general procedure C with acetic anhydride (140 μL, 1.48 mmol), and DIPEA (520 μL, 3.00 mmol) in DCM (30 mL). The crude mixture was purified by flash chromatography on silica gel eluting with hexane:EtOAc (1:1) − EtOAc to provide **11d** (400 mg, 95%) as a white solid compound.

^1^H NMR (400 MHz, Dimethylsulfoxide-*d_6_*) δ 8.21 (t, *J* = 5.9 Hz, 1H), 7.96 (d, *J* = 8.5 Hz, 1H), 7.85 (d, *J* = 8.3 Hz, 1H), 4.41 − 4.30 (m, 1H), 4.13 (t, *J* = 8.5 Hz, 1H), 4.07 (q, *J* = 7.2 Hz, 2H), 3.84 (dd, *J* = 17.3, 6.0 Hz, 1H), 3.74 (dd, *J* = 17.3, 5.9 Hz, 1H), 2.11 (h, *J* = 8.3 Hz, 1H), 1.84 (s, 3H), 1.73 − 1.39 (m, 14H), 1.34 − 1.20 (m, 3H), 1.18 (t, *J* = 7.1 Hz, 3H), 1.14 − 1.06 (m, 2H), 0.96 − 0.75 (m, 2H).^13^C NMR (101 MHz, Dimethylsulfoxide-*d_6_*) δ 172.5, 171.2, 169.6, 169.2, 60.4, 56.3, 49.9, 41.7, 40.7, 33.2, 31.7, 28.6, 28.5, 26.1, 25.8, 25.6, 24.9, 24.5, 22.5, 14.0. HR-MS (ESI/TOF) calcd for C_22_H_38_N_3_O_5_ [M+H]^+^ 424.2811, found 424.2812.

#### Ethyl ((S)-2-((S)-2-acetamido-2-cyclopentylacetamido)-4,4-dimethylpentanoyl)glycin-ate (11e)

Deprotection was performed according to general procedure B from starting material **10e** (389 mg, 0.85 mmol) and 4 M HCl in dioxane (0.86 mL) in DCM (20 mL). After a full conversion of the starting material, the residue was utilized in the acylation reaction according to general procedure C with acetic anhydride (120 μL, 1.27 mmol), and DIPEA (440 μL, 2.54 mmol) in DCM (15 mL). The crude mixture was purified by flash chromatography on silica gel eluting with hexane:EtOAc (1:1) − EtOAc to provide **11e** (297 mg, 87%) as a white solid compound.

^1^H NMR (400 MHz, Chloroform-*d*) δ 7.46 (d, *J* = 8.5 Hz, 1H), 7.33 (t, *J* = 5.5 Hz, 1H), 6.78 (d, *J* = 8.8 Hz, 1H), 4.59 (td, *J* = 8.3, 4.4 Hz, 1H), 4.44 (t, *J* = 9.2 Hz, 1H), 4.17 (q, *J* = 7.2 Hz, 2H), 4.04 (dd, *J* = 18.0, 5.8 Hz, 1H), 3.87 (dd, *J* = 18.1, 5.1 Hz, 1H), 2.21 (h, *J* = 9.0 Hz, 1H), 1.99 (s, 3H), 1.93 − 1.84 (m, 2H), 1.79 − 1.44 (m, 7H), 1.26 (t, *J* = 7.2 Hz, 5H), 0.91 (s, 9H). ^13^C NMR (101 MHz, Chloroform-*d*) δ 172.8, 171.9, 170.4, 169.8, 61.4, 57.3, 50.8, 45.3, 42.9, 41.5, 30.5, 29.7, 29.4, 29.4, 25.5, 25.0, 23.1, 14.3. HR-MS (ESI/TOF) calcd for C_20_H_35_N_3_O_5_Na [M+Na]^+^ 420.2474, found 420.2473.

#### Ethyl ((S)-2-acetamido-2-cyclopentylacetyl)-L-isoleucylglycinate (11f)

Deprotection was performed according to general procedure B from starting material **10f** (456 mg, 1.03 mmol) and 4 M HCl in dioxane (1.05 mL) in DCM (20 mL). After a full conversion of the starting material, the intermediate was subjected to the acylation reaction according to general procedure C with acetic anhydride (150 μL, 1.59 mmol), and DIPEA (540 μL, 3.12 mmol) in DCM (20 mL). The crude mixture was purified by flash chromatography on silica gel eluting with hexane:EtOAc (1:1) − EtOAc to provide **11f** (240 mg, 61%) as a white solid compound.

^1^H NMR (400 MHz, Dimethylsulfoxide-*d_6_*) δ 8.35 (t, *J* = 5.8 Hz, 1H), 7.98 (d, *J* = 8.6 Hz, 1H), 7.69 (d, *J* = 8.9 Hz, 1H), 4.23 − 4.14 (m, 2H), 4.07 (q, *J* = 7.1 Hz, 2H), 3.85 (dd, *J* = 17.3, 6.0 Hz, 1H), 3.76 (dd, *J* = 17.3, 5.8 Hz, 1H), 2.11 (h, *J* = 8.2 Hz, 1H), 1.84 (s, 3H), 1.75 − 1.66 (m, 1H), 1.66 − 1.36 (m, 7H), 1.32 − 1.13 (m, 5H), 1.12 − 1.00 (m, 1H), 0.90 − 0.74 (m, 6H). ^13^C NMR (101 MHz, Dimethylsulfoxide-*d_6_*) δ 171.4, 171.3, 169.6, 169.2, 60.4, 56.5, 56.2, 41.6, 40.7, 36.8, 28.7, 28.6, 24.9, 24.6, 24.2, 22.5, 15.2, 14.0, 11.1. HR-MS (ESI/TOF) calcd for C_19_H_33_N_3_O_5_Na [M+Na]^+^ 406.2318, found 406.2328.

#### Ethyl ((S)-2-acetamido-2-cyclopentylacetyl)-L-leucylglycinate (11g)

Deprotection was performed according to general procedure B from starting material **10g** (313 mg, 0.71 mmol) and 4 M HCl in dioxane (0.71 mL) in DCM (20 mL). After a full conversion of the intermediate was subjected to the acylation reaction according to general procedure C with acetic anhydride (100 μL, 1.06 mmol) and DIPEA (370 μL, 2.14 mmol) in DCM (20 mL). The crude mixture was purified by flash chromatography on silica gel eluting with hexane:EtOAc (1:1) − EtOAc to provide **11g** (232 mg, 85%) as a white solid compound.

^1^H NMR (400 MHz, Dimethylsulfoxide-*d_6_*) δ 8.26 (t, *J* = 5.9 Hz, 1H), 7.94 (d, *J* = 8.5 Hz, 1H), 7.88 (d, *J* = 8.3 Hz, 1H), 4.32 (q, *J* = 7.9 Hz, 1H), 4.15 (t, *J* = 8.5 Hz, 1H), 4.07 (q, *J* = 7.0 Hz, 2H), 3.84 (dd, *J* = 17.3, 5.9 Hz, 1H), 3.75 (dd, *J* = 17.3, 5.8 Hz, 1H), 2.10 (h, *J* = 8.6 Hz, 1H), 1.84 (s, 3H), 1.70 − 1.40 (m, 9H), 1.34 − 1.20 (m, 2H), 1.17 (t, *J* = 7.1 Hz, 3H), 0.88 (d, *J* = 6.6 Hz, 3H), 0.83 (d, *J* = 6.6 Hz, 3H). ^13^C NMR (101 MHz, Dimethylsulfoxide-*d_6_*) δ 172.4, 171.3, 169.6, 169.2, 60.4, 56.1, 50.6, 41.8, 41.0, 40.7, 28.6, 28.5, 24.9, 24.5, 24.0, 23.0, 22.5, 21.7, 14.0. HR-MS (ESI/TOF) calcd for C_19_H_33_N_3_O_5_Na [M+Na]^+^ 406.2318, found 406.2322.

#### Ethyl ((S)-2-acetamido-2-cyclopentylacetyl)-L-phenylalanylglycinate (11h)

Deprotection was performed according to general procedure B from starting material **10h** (391 mg, 0.82 mmol) and 4 M HCl in dioxane (0.82 mL) in DCM (20 mL). After a full conversion of the starting material, the intermediate was subjected to the acylation reaction according to general procedure C with acetic anhydride (110 μL, 1.16 mmol) and DIPEA (430 μL, 2.48 mmol) in DCM (30 mL). The crude mixture was purified by flash chromatography on silica gel eluting with hexane:EtOAc (1:1) − EtOAc to provide **11h** (297 mg, 86%) as a white solid compound.

^1^H NMR (300 MHz, Dimethylsulfoxide-*d_6_*) δ 8.36 (t, *J* = 5.9 Hz, 1H), 7.93 (d, *J* = 8.6 Hz, 1H), 7.87 (d, *J* = 8.5 Hz, 1H), 7.28 − 7.14 (m, 5H), 4.55 (td, *J* = 9.7, 4.5 Hz, 1H), 4.19 − 4.03 (m, 3H), 3.94 − 3.73 (m, 2H), 3.03 (dd, *J* = 13.9, 4.6 Hz, 1H), 2.79 (dd, *J* = 13.9, 9.7 Hz, 1H), 2.08 − 1.92 (m, 1H), 1.81 (s, 3H), 1.62 − 1.28 (m, 6H), 1.25 − 1.09 (m, 5H). ^13^C NMR (101 MHz, Dimethylsulfoxide-*d_6_*) δ 171.5, 171.2, 169.6, 169.2, 137.7, 129.2, 128.0, 126.2, 60.4, 56.3, 53.4, 41.8, 40.8, 37.5, 28.6, 28.5, 24.8, 24.5, 22.5, 14.1. HR-MS (ESI/TOF) calcd for C_22_H_31_N_3_O_5_Na [M+Na]^+^ 440.2161, found 440.2167.

#### (S)-2-((S)-2-acetamido-2-cyclopentylacetamido)-3,3-dimethyl-N-(2-oxo-2-(((R)-1-((3aS,4S,6S, 7aR)-3a,5,5-trimethylhexahydro-4,6-methanobenzo[d][1,3,2]dioxaborol-2-yl)ethyl)amino)ethyl) butanamide (13a)

Hydrolysis was performed according to general procedure D from starting material **11a** (369 mg, 0.96 mmol), LiOH (230 mg, 9.6 mmol) in THF:H_2_O (10.5 mL). Intermediate acid (300 mg, 88%) was isolated as white solid compound. Amide bond coupling was performed according to general procedure E from an intermediate acid (200 mg, 0.56 mmol), NMM (200 μL, 1.82 mmol), and T3P (670 μL, 1.12 mmol) in 10 mL EtOAc and **12** (175 mg, 0.67 mmol) in DMF (1 mL). Flash chromatography on silica gel eluting with 0−5 % MeOH in EtOAc provided **13a** (148 mg, 47%) as an amorphous solid. ^1^H NMR (400 MHz, Methanol-*d_4_*) δ 4.23 (d, *J* = 9.5 Hz, 1H), 4.19 − 4.13 (m, 2H), 4.06 (s, 1H), 3.96 (dd, *J* = 17.4, 1.0 Hz, 1H), 2.65 (q, *J* = 7.3 Hz, 1H), 2.39 − 2.27 (m, 1H), 2.23 (h, *J* = 8.6 Hz, 1H), 2.19 − 2.06 (m, 1H), 1.97 (s, 3H), 1.94 (t, *J* = 5.5 Hz, 1H), 1.89 − 1.75 (m, 3H), 1.72 − 1.63 (m, 3H), 1.62 − 1.50 (m, 2H), 1.44 (d, *J* = 10.3 Hz, 1H), 1.38 − 1.27 (m, 8H), 1.16 (d, *J* = 7.3 Hz, 3H), 1.03 (s, 9H), 0.87 (s, 3H). ^13^C NMR (101 MHz, Methanol-*d_4_*) δ 175.8, 174.8, 173.4, 173.3, 84.3, 77.3, 63.5, 58.9, 53.6, 42.8, 41.4, 39.9 (overlaps with carbon CHB (broad)), 39.2, 37.7, 34.6, 30.5, 30.2, 29.6, 27.8, 27.5, 27.1, 26.3, 26.0, 24.6, 22.3, 16.4. HR-MS (ESI/TOF) calcd for C_29_H_50_BN_4_O_6_ [M+H]^+^ 561.3823, found 561.3842.

#### (S)-2-acetamido-2-cyclopentyl-N-((S)-2-oxo-2-((2-oxo-2-(((R)-1-((3aS,4S,6S,7aR)-3a,5,5-trimethylhexahydro-4,6-methanobenzo[d][1,3,2]dioxaborol-2-yl)ethyl)amino)ethyl)amino)-1-phenylethyl)acetamide (13b)

Hydrolysis was performed according to general procedure D from starting material **11b** (177 mg, 0.44 mmol) and LiOH (105 mg, 4.44 mmol) in THF:H_2_O (10.5 mL). Intermediate acid (120 mg, 73%) was isolated as white solid compound. Amide bond coupling was performed according to general procedure E from an intermediate acid (100 mg, 0.27 mmol), NMM (100 μL, 0.91 mmol), and T3P (320 μL, 0.54 mmol) in EtOAc (5 mL) and **12** (83 mg, 0.32 mmol) in DMF (1 mL). Flash chromatography on silica gel eluting with 0−5 % MeOH in EtOAc provided **13b** (54 mg, 35%) as an amorphous solid.

^1^H NMR (400 MHz, Methanol-*d_4_*) δ 7.50 − 7.42 (m, 2H), 7.43 − 7.31 (m, 3H), 5.31 (s, 1H), 4.26 − 4.19 (m, 1H), 4.19 − 4.06 (m, 2H), 3.88 (dd, *J* = 17.6, 1.0 Hz, 1H), 2.67 (q, *J* = 7.4 Hz, 1H), 2.39 − 2.27 (m, 1H), 2.29 − 2.16 (m, 1H), 2.19 − 2.07 (m, 1H), 1.97 (s, 3H), 1.94 (t, *J* = 5.5 Hz, 1H), 1.88 − 1.72 (m, 4H), 1.71 − 1.54 (m, 4H), 1.43 (d, *J* = 10.4 Hz, 1H), 1.39 − 1.24 (m, 8H), 1.17 (d, *J* = 7.3 Hz, 3H), 0.87 (s, 3H). ^13^C NMR (101 MHz, Methanol-*d_4_*) δ 175.7, 174.4, 173.4, 172.9, 137.4, 129.9, 129.6, 129.0, 84.3, 77.3, 59.6, 58.6, 53.6, 43.1, 41.4, 40.3, 39.8 (CHB (broad)), 39.2, 37.7, 30.3, 30.2, 29.6, 27.8, 27.5, 26.3, 25.9, 24.6, 24.5, 22.3, 16.4. HR-MS (ESI/TOF) calcd for C_31_H_46_BN_4_O_6_ [M+H]^+^ 581.3510, found 581.3528.

#### (S)-2-((S)-2-acetamido-2-cyclopentylacetamido)-3-cyclopentyl-N-(2-oxo-2-(((R)-1-((3aS,4S,6S,7aR)-3a,5,5-trimethylhexahydro-4,6-methanobenzo[d][1,3,2]dioxaborol-2-yl)ethyl)amino)ethyl)propanamide (13c)

Hydrolysis was performed according to general procedure D from starting material **11c** (258 mg, 0.63 mmol) and LiOH (151 mg, 6.30 mmol) in THF:H_2_O (30 mL). Intermediate acid (220 mg, 92%) was isolated as white solid compound. Amide bond coupling was performed according to general procedure E from an intermediate acid (200 mg, 0.52 mmol), NMM (170 μL, 1.55 mmol), T3P (620 μL, 1.04 mmol) in EtOAc (5 mL) and **12** (163 mg, 0.63 mmol) in DMF (1 mL) was added. Flash chromatography on silica gel eluting with 0−5 % MeOH in EtOAc provided **13c** (75 mg, 24%) as an amorphous solid.

^1^H NMR (400 MHz, Methanol-*d_4_*) δ 4.26 − 4.08 (m, 4H), 3.93 (d, *J* = 17.6 Hz, 1H), 2.64 (q, *J* = 7.3 Hz, 1H), 2.38 − 2.28 (m, 1H), 2.26 − 2.15 (m, 1H), 2.18 − 2.07 (m, 1H), 1.98 (s, 3H), 1.95 (t, *J* = 5.5 Hz, 1H), 1.92 − 1.73 (m, 8H), 1.72 − 1.50 (m, 9H), 1.44 (d, *J* = 10.3 Hz, 1H), 1.40 − 1.26 (m, 8H), 1.21 − 1.09 (m, 5H), 0.87 (s, 3H). ^13^C NMR (101 MHz, Methanol-*d_4_*) δ 175.9, 175.2, 174.9, 173.4, 84.3, 77.3, 58.9, 55.2, 53.6, 43.1, 41.4, 40.1, 39.8 (CHB (broad)), 39.2, 38.4, 37.74, 37.70, 33.8, 33.2, 30.4, 30.2, 29.6, 27.8, 27.5, 26.3, 26.1, 26.0, 25.9, 24.6, 22.3, 16.4. HR-MS (ESI/TOF) calcd for C_31_H_51_BN_4_O_6_Na [M+Na]^+^ 609.3799, found 609.3820

#### (S)-2-((S)-2-acetamido-2-cyclopentylacetamido)-3-cyclohexyl-N-(2-oxo-2-(((R)-1-((3aS,4S,6S, 7aR)-3a,5,5-trimethylhexahydro-4,6-methanobenzo[d][1,3,2]dioxaborol-2-yl)ethyl)amino)ethyl) propanamide (13d)

Hydrolysis was performed according to general procedure D from starting material **11d** (420 mg, 0.99 mmol) and LiOH (238 mg, 9.94 mmol) in THF:H_2_O (30 mL). Intermediate acid (350 mg, 89%) was isolated as white solid compound. Amide bond coupling was performed according to general procedure E from an intermediate acid (200 mg, 0.50 mmol), NMM (170 μL, 1.55 mmol), solution of T3P (600 μL, 1.01 mmol) in EtOAc (5 mL) and **12** (158 mg, 0.61 mmol) in DMF (1 mL). Flash chromatography on silica gel eluting with 0−5 % MeOH in EtOAc provided **13d** (90 mg, 30%) as an amorphous solid.

^1^H NMR (400 MHz, Methanol-*d_4_*) δ 4.27 (t, *J* = 7.6 Hz, 1H), 4.21 − 4.10 (m, 3H), 3.93 (dd, *J* = 17.6, 1.0 Hz, 1H), 2.65 (q, *J* = 7.2 Hz, 1H), 2.38 − 2.29 (m, 1H), 2.29 − 2.15 (m, 1H), 2.17 − 2.09 (m, 1H), 1.98 (s, 3H), 1.95 (t, *J* = 5.5 Hz, 1H), 1.90 − 1.84 (m, 1H), 1.82 − 1.52 (m, 14H), 1.44 (d, *J* = 10.4 Hz, 1H), 1.41 − 1.20 (m, 12H), 1.17 (d, *J* = 7.3 Hz, 3H), 1.05 − 0.84 (m, 5H). ^13^C NMR (101 MHz, Methanol-*d_4_*) δ 175.9, 175.4, 175.0, 173.5, 84.3, 77.3, 59.0, 53.6, 53.1, 43.0, 41.4, 40.1, 39.8 (CHB (broad)), 39.6, 39.2, 37.7, 35.2, 34.8, 33.4, 30.4, 30.2, 29.6, 27.8, 27.6, 27.5, 27.4, 27.2, 26.2, 25.9, 24.6, 22.4, 16.4. HR-MS (ESI/TOF) calcd for C_32_H_54_BN_4_O_6_ [M+H]^+^ 601.4136, found 601.4150.

#### (S)-2-((S)-2-acetamido-2-cyclopentylacetamido)-4,4-dimethyl-N-(2-oxo-2-(((R)-1-((3aS,4S,6S, 7aR)-3a,5,5-trimethylhexahydro-4,6-methanobenzo[d][1,3,2]dioxaborol-2-yl)ethyl)amino) ethyl)pentanamide (13e)

Hydrolysis was performed according to general procedure D from starting material **11e** (289 mg, 0.73 mmol) and LiOH (174 mg, 7.27 mmol) in THF:H_2_O (21 mL). Intermediate acid (253 mg, 94%) was isolated as white solid compound. Amide bond coupling was performed according to general procedure E from an intermediate acid (210 mg, 0.57 mmol), NMM (200 μL, 1.82 mmol), and T3P (700 μL, 1.18 mmol in EtOAc (7 mL) and **12** (177 mg, 0.68 mmol) in DMF (1 mL). Flash chromatography on silica gel eluting with 0−5 % MeOH in EtOAc provided **13e** (171 mg, 52%) as an amorphous solid.

^1^H NMR (400 MHz, Methanol-*d_4_*) δ 4.26 (dd, *J* = 8.0, 4.3 Hz, 1H), 4.19 − 4.09 (m, 3H), 3.93 (dd, *J* = 17.5, 1.0 Hz, 1H), 2.65 (q, *J* = 7.2 Hz, 1H), 2.39 − 2.28 (m, 1H), 2.28 − 2.15 (m, 1H), 2.17 − 2.08 (m, 1H), 1.97 (s, 3H), 1.95 (t, *J* = 5.6 Hz, 1H), 1.89 − 1.74 (m, 4H), 1.72 − 1.51 (m, 6H), 1.44 (d, *J* = 10.4 Hz, 1H), 1.32 (d, *J* = 29.0 Hz, 8H), 1.17 (d, *J* = 7.3 Hz, 3H), 0.96 (s, 9H), 0.87 (s, 3H). ^13^C NMR (101 MHz, Methanol-*d_4_*) δ 175.8, 175.6, 174.7, 173.4, 84.3, 77.3, 59.0, 53.6, 52.9, 45.6, 43.0, 41.4, 40.2, 39.8 (CHB (broad)), 39.2, 37.7, 31.3, 30.5, 30.2, 30.0, 29.6, 27.8, 27.5, 26.3, 25.9, 24.5, 22.3, 16.4. HR-MS (ESI/TOF) calcd for C_30_H_52_BN_4_O_6_ [M+H]^+^ 575.3980, found 575.3998.

#### (2S,3S)-2-((S)-2-acetamido-2-cyclopentylacetamido)-3-methyl-N-(2-oxo-2-(((R)-1-((3aS,4S,6S, 7aR)-3a,5,5-trimethylhexahydro-4,6-methanobenzo[d][1,3,2]dioxaborol-2-yl)ethyl)amino) ethyl)pentanamide (13f)

Hydrolysis was performed according to general procedure D from starting material **11f** (223 mg, 0.58 mmol) and LiOH (140 mg, 5.84 mmol) in THF:H_2_O (21 mL), was added and the reaction was stirred at room temperature. Intermediate acid (193 mg, 93%) was isolated as white solid compound. Amide bond coupling was performed according to general procedure E from an intermediate acid an acid (180 mg, 0.51 mmol), NMM (170 μL, 1.55 mmol), T3P (600 μL, 1.01 mmol) in EtOAc (10 mL) and **12** (158 mg, 0.61 mmol) in DMF (1 mL). Flash chromatography on silica gel eluting with 0−5 % MeOH in EtOAc provided **13f** (94 mg, 33%) as an amorphous solid.

^1^H NMR (400 MHz, Methanol-*d_4_*) δ 4.24 − 4.13 (m, 3H), 4.02 (d, *J* = 8.5 Hz, 1H), 3.94 (dd, *J* = 17.5, 1.0 Hz, 1H), 2.64 (q, *J* = 7.3 Hz, 1H), 2.40 − 2.29 (m, 1H), 2.28 − 2.15 (m, 1H), 2.18 − 2.07 (m, 1H), 1.97 (s, 3H), 1.94 (t, *J* = 5.5 Hz, 1H), 1.89 − 1.74 (m, 4H), 1.72 − 1.51 (m, 6H), 1.44 (d, *J* = 10.4 Hz, 1H), 1.39 − 1.27 (m, 8H), 1.26 − 1.19 (m, 1H), 1.17 (d, *J* = 7.3 Hz, 3H), 0.97 − 0.90 (m, 6H), 0.87 (s, 3H). ^13^C NMR (101 MHz, Methanol-*d_4_*) δ 175.8, 175.0, 174.4, 173.3, 84.3, 77.3, 60.2, 58.8, 53.6, 43.0, 41.4, 40.0 (overlaps with carbon CHB (broad)), 39.2, 37.7, 37.2, 30.4, 30.2, 29.6, 27.8, 27.5, 26.3, 26.3, 25.9, 24.6, 22.3, 16.4, 15.8, 11.2. LC-MS (ESI) calcd for C_29_H_50_BN_4_O_6_ [M+H]^+^ 561.55, found 561.86

#### (S)-2-((S)-2-acetamido-2-cyclopentylacetamido)-4-methyl-N-(2-oxo-2-(((R)-1-((3aS,4S,6S, 7aR)-3a,5,5-trimethylhexahydro-4,6-methanobenzo[d][1,3,2]dioxaborol-2-yl)ethyl)amino) ethyl)pentanamide (13g)

Hydrolysis was performed according to general procedure D from starting material **11g** (222 mg, 0.58 mmol and LiOH (140 mg, 5.84 mmol)) in THF:H_2_O (50 mL). Intermediate acid (202 mg, 98%) was isolated as white solid compound. Amide bond coupling was performed according to general procedure E from an intermediate acid (174 mg, 0.49 mmol), NMM (160 μL, 1.45 mmol), T3P (580 μL, 0.98 mmol) in EtOAc (6 mL) and **12** (153 mg, 0.59 mmol) in DMF (1 mL). Flash chromatography on silica gel eluting with 0−5 % MeOH in EtOAc provided **13g** (111 mg, 41%) as an amorphous solid.

^1^H NMR (400 MHz, Methanol-*d_4_*) δ 4.27 − 4.09 (m, 4H), 3.94 (dd, *J* = 17.6, 1.0 Hz, 1H), 2.65 (q, *J* = 7.3 Hz, 1H), 2.38 − 2.29 (m, 1H), 2.26 − 2.17 (m, 1H), 2.17 − 2.10 (m, 1H), 1.98 (s, 3H), 1.95 (t, *J* = 5.5 Hz, 1H), 1.89 − 1.75 (m, 3H), 1.73 − 1.52 (m, 8H), 1.44 (d, *J* = 10.3 Hz, 1H), 1.41 − 1.27 (m, 8H), 1.17 (d, *J* = 7.3 Hz, 3H), 0.97 (d, *J* = 6.3 Hz, 3H), 0.93 (d, *J* = 6.4 Hz, 3H), 0.88 (s, 3H).^13^C NMR (101 MHz, Methanol-*d_4_*) δ 175.9, 175.2, 175.0, 173.5, 84.3, 77.3, 59.0, 53.9, 53.6, 43.0, 41.4, 41.0, 40.1, 39.8 (CHB (broad)), 39.2, 37.7, 30.4, 30.2, 29.6, 27.8, 27.5, 26.2, 25.9, 25.8, 24.6, 23.3, 22.3, 22.1, 16.4. HR-MS (ESI/TOF) calcd for C_29_H_50_BN_4_O_6_ [M+H]^+^ 561.3823, found 561.3846

#### (S)-2-((S)-2-acetamido-2-cyclopentylacetamido)-N-(2-oxo-2-(((R)-1-((3aS,4S,6S, 7aR)-3a,5,5-trimethylhexahydro-4,6-methanobenzo[d][1,3,2]dioxaborol-2-yl)ethyl) amino)ethyl)-3-phenylpropanamide (13h)

Hydrolysis was performed according to general procedure D from starting material **11h** (289 mg, 0.69 mmol) and LiOH (166 mg, 6.93 mmol) in THF:H_2_O (50 mL). Intermediate acid (242 mg, 90%) was isolated as white solid compound. Amide bond coupling was performed according to general procedure E from an intermediate acid (201 mg, 0.52 mmol), NMM (180 μL, 1.64 mmol), T3P (680 μL, 1.14 mmol) in EtOAc (6 mL) and **12** (176 mg, 0.68 mmol) in DMF (2 mL). Flash chromatography on silica gel eluting with 0−5 % MeOH in EtOAc provided **13h** (116 mg, 38%) as an amorphous solid.

^1^H NMR (400 MHz, Methanol-*d_4_*) δ 7.31 − 7.18 (m, 5H), 4.45 (dd, *J* = 8.5, 6.8 Hz, 1H), 4.17 − 4.15 (m, 1H), 4.13 (dd, *J* = 11.8, 2.0 Hz, 1H), 4.07 (d, *J* = 9.2 Hz, 1H), 3.82 (dd, *J* = 17.6, 1.0 Hz, 1H), 3.16 (dd, *J* = 13.8, 6.8 Hz, 1H), 2.99 (dd, *J* = 13.9, 8.4 Hz, 1H), 2.65 (q, *J* = 7.6 Hz, 1H), 2.37 − 2.29 (m, 1H), 2.19 − 2.05 (m, 2H), 1.98 − 1.92 (m, 4H), 1.88 − 1.82 (m, 1H), 1.81 − 1.71 (m, 2H), 1.67 − 1.47 (m, 5H), 1.43 (d, *J* = 10.4 Hz, 1H), 1.35 (s, 3H), 1.29 − 1.20 (m, 5H), 1.16 (d, *J* = 7.3 Hz, 3H), 0.87 (s, 3H). ^13^C NMR (101 MHz, Methanol-*d_4_*) δ 175.7, 174.8, 174.1, 173.5, 138.2, 130.3, 129.5, 127.9, 84.4, 77.3, 59.2, 56.8, 53.6, 42.9, 41.3, 40.2, 39.6 (CHB (broad)), 39.2, 37.9, 37.7, 30.4, 30.1, 29.6, 27.8, 27.5, 26.2, 25.8, 24.5, 22.4, 16.4. HR-MS (ESI/TOF) calcd for C_32_H_48_BN_4_O_6_ [M+H]^+^ 595.3667, found 595.3693.

#### ((2R,8S,11S)-8-(tert-butyl)-11-cyclopentyl-4,7,10,13-tetraoxo-3,6,9,12-tetraazatetra decan-2-yl)boronic acid (2a)

Was prepared according to general procedure F from a solution of **13a** (135 mg, 0.24 mmol) in MeOH/*n*-hexane (9.2 mL), isobutylboronic acid (74 mg, 0.72 mmol) and 1 M HCl (600 μL). Purification by flash chromatography on reversed phase silica gel provided **2a** (76 mg, 74%) as a white solid.

^1^H NMR (400 MHz, Methanol-*d_4_*) δ 4.24 (d, *J* = 9.5 Hz, 1H), 4.20 (dd, *J* = 17.5, 1.6 Hz, 1H), 4.07 (s, 1H), 3.98 (dd, *J* = 17.4, 1.0 Hz, 1H), 2.66 (q, *J* = 7.2 Hz, 1H), 2.22 (h, *J* = 8.9 Hz, 1H), 1.98 (s, 3H), 1.86 − 1.77 (m, 1H), 1.73 − 1.62 (m, 3H), 1.62 − 1.49 (m, 2H), 1.39 − 1.24 (m, 2H), 1.12 (d, *J* = 7.2 Hz, 3H), 1.04 (s, 9H). ^13^C NMR (101 MHz, Methanol-*d_4_*) δ 176.2, 174.8, 173.5, 173.4, 63.5, 58.9, 42.9, 41.9 (CHB (broad)), 39.5, 34.6, 30.4, 30.3, 27.0, 26.3, 25.9, 22.3, 15.9. HR-MS (ESI/TOF) calcd for C_19_H_35_BN_4_O_6_Na [M+Na]^+^ 449.2547, found 449.2560.

#### ((2R,8S,11S)-11-cyclopentyl-4,7,10,13-tetraoxo-8-phenyl-3,6,9,12-tetraazatetradecan -2-yl)boronic acid (2b)

Was prepared according to general procedure F from a solution of **13b** (50 mg, 0.086 mmol) in MeOH/*n*-hexane (3.3 mL), isobutylboronic acid (27 mg, 0.26 mmol) and 1 M HCl (215 μL). Purification by flash chromatography on reversed phase silica gel provided **2b** (29 mg, 75%) as a white solid.

^1^H NMR (400 MHz, Methanol-*d_4_*) δ 7.49 − 7.44 (m, 2H), 7.40 − 7.32 (m, 3H), 5.33 (s, 1H), 4.26 − 4.10 (m, 2H), 3.95 (dd, *J* = 17.6, 1.0 Hz, 1H), 2.67 (q, *J* = 7.2 Hz, 1H), 2.23 (h, *J* = 9.0 Hz, 1H), 1.97 (s, 3H), 1.86 − 1.51 (m, 6H), 1.47 − 1.24 (m, 2H), 1.12 (d, *J* = 7.2 Hz, 3H). ^13^C NMR (101 MHz, Methanol-*d_4_*) δ 176.2, 174.4, 173.40 173.1, 137.3, 129.9, 129.7, 129.0, 59.4, 58.6, 43.2, 41.9 (CHB (broad)), 39.9, 30.3, 30.2, 26.2, 25.9, 22.3, 15.9. HR-MS (ESI/TOF) calcd for C_21_H_31_BN_4_O_6_Na [M+Na]^+^ 469.2234, found 469.2235

#### ((2R,8S,11S)-11-cyclopentyl-8-(cyclopentylmethyl)-4,7,10,13-tetraoxo-3,6,9,12-tetra-azatetradecan-2-yl)boronic acid (2c)

Was prepared according to general procedure F from a solution of **13c** (72 mg, 0.123 mmol) in MeOH/*n*-hexane (4.7 mL), isobutylboronic acid (40 mg, 0.39 mmol) and 1 M HCl (310 μL). Purification by flash chromatography on reversed phase silica gel provided **2c** (33 mg, 59%) as a white solid.

^1^H NMR (400 MHz, Methanol-*d_4_*) δ 4.25 − 4.16 (m, 2H), 4.13 (d, *J* = 9.2 Hz, 1H), 3.97 (dd, *J* = 17.6, 1.0 Hz, 1H), 2.66 (q, *J* = 7.2 Hz, 1H), 2.20 (h, *J* = 8.9 Hz, 1H), 1.98 (s, 3H), 1.94 − 1.73 (m, 6H), 1.72 − 1.49 (m, 9H), 1.43 − 1.26 (m, 2H), 1.23 − 1.10 (m, 5H). ^13^C NMR (101 MHz, Methanol-*d_4_*) δ 176.3, 175.3, 175.0, 173.5, 59.0, 55.1, 43.1, 42.0 (CHB (broad)), 39.8, 38.3, 37.7, 33.7, 33.2, 30.4, 30.2, 26.2, 26.1, 26.0, 25.9, 22.3, 15.9. HR-MS (ESI/TOF) calcd for C_21_H_37_BN_4_O_6_Na [M+Na]^+^ 475.2704, found 475.2723.

#### ((2R,8S,11S)-8-(cyclohexylmethyl)-11-cyclopentyl-4,7,10,13-tetraoxo-3,6,9,12-tetra-azatetradecan-2-yl)boronic acid (2d)

Was prepared according to general procedure F from a solution of **13d** (74 mg, 0.123 mmol) in MeOH/*n*-hexane (4.7 mL), with isobutylboronic acid (40 mg, 0.39 mmol) and 1 M HCl (310 μL). Purification by flash chromatography on reversed phase silica gel provided **2d** (31 mg, 54%) as a white solid.

^1^H NMR (400 MHz, Methanol-*d_4_*) δ 4.28 (t, *J* = 7.7 Hz, 1H), 4.20 (dd, *J* = 17.6, 1.7 Hz, 1H), 4.13 (d, *J* = 9.3 Hz, 1H), 3.97 (dd, *J* = 17.6, 1.0 Hz, 1H), 2.66 (q, *J* = 7.1 Hz, 1H), 2.21 (h, *J* = 8.8 Hz, 1H), 1.98 (s, 3H), 1.88 − 1.50 (m, 13H), 1.44 − 1.16 (m, 6H), 1.12 (d, *J* = 7.2 Hz, 3H), 1.05 − 0.86 (m, 2H). ^13^C NMR (101 MHz, Methanol-*d_4_*) δ 176.3, 175.5, 175.0, 173.5, 59.1, 53.1, 43.0, 41.9 (CHB (broad)), 39.8, 39.5, 35.2, 34.8, 33.5, 30.4, 30.2, 27.6, 27.4, 27.2, 26.2, 25.9, 22.4, 16.0.HR-MS (ESI/TOF) calcd for C_22_H_39_BN_4_O_6_Na [M+Na]^+^ 489.2860, found 489.2850.

#### ((2R,8S,11S)-11-cyclopentyl-8-neopentyl-4,7,10,13-tetraoxo-3,6,9,12-tetraazatetra-decan-2-yl)boronic acid (2e)

Was prepared according to general procedure F from a solution of **13e** (151 mg, 0.263 mmol) in MeOH/*n*-hexane (10 mL), isobutylboronic acid (107 mg, 1.05 mmol) and 1 M HCl (650 μL). Purification by flash chromatography on reversed phase silica gel provided **2e** (95 mg, 82%) as a white solid.

^1^H NMR (400 MHz, Methanol-*d_4_*) δ 4.26 (dd, *J* = 8.0, 4.3 Hz, 1H), 4.19 (dd, *J* = 17.6, 1.7 Hz, 1H), 4.11 (d, *J* = 9.5 Hz, 1H), 3.96 (dd, *J* = 17.6, 1.0 Hz, 1H), 2.65 (q, *J* = 7.2 Hz, 1H), 2.21 (h, *J* = 8.6 Hz, 1H), 1.97 (s, 3H), 1.86 − 1.75 (m, 2H), 1.74 − 1.50 (m, 6H), 1.42 − 1.25 (m, 2H), 1.12 (d, *J* = 7.2 Hz, 3H), 0.97 (s, 9H). ^13^C NMR (101 MHz, Methanol-*d_4_*) δ 176.3, 175.7, 174.7, 173.5, 59.1, 52.9, 45.5, 43.0, 41.8 (CHB (broad)), 39.8, 31.3, 30.5, 30.2, 30.0, 26.3, 25.9, 22.3, 16.0. HR-MS (ESI/TOF) calcd for C_20_H_37_BN_4_O_6_Na [M+Na]^+^ 463.2704, found 463.2701

#### ((2R,8S,11S)-8-((S)-sec-butyl)-11-cyclopentyl-4,7,10,13-tetraoxo-3,6,9,12-tetraaza-tetradecan-2-yl)boronic acid (2f)

Was prepared according to general procedure F from a solution of a solution of **13f** (84 mg, 0.150 mmol) in MeOH/*n*-hexane (5.8 mL), isobutylboronic acid (61 mg, 0.60 mmol) and 1 M HCl (375 μL). Purification by flash chromatography on reversed phase silica gel provided **2f** (52 mg, 81%) as a white solid.

^1^H NMR (400 MHz, Methanol-*d_4_*) δ 4.23 (dd, *J* = 17.5, 1.7 Hz, 1H), 4.18 (d, *J* = 9.5 Hz, 1H), 4.04 (d, *J* = 8.3 Hz, 1H), 3.98 (dd, *J* = 17.6, 1.0 Hz, 1H), 2.66 (q, *J* = 6.9 Hz, 1H), 2.28 − 2.13 (m, 1H), 1.98 (s, 3H), 1.89 − 1.76 (m, 2H), 1.72 − 1.50 (m, 6H), 1.41 − 1.27 (m, 2H), 1.26 − 1.17 (m, 1H), 1.12 (d, *J* = 7.3 Hz, 3H), 0.95 (d, *J* = 6.8 Hz, 3H), 0.92 (t, *J* = 7.5 Hz, 3H). ^13^C NMR (101 MHz, Methanol-*d_4_*) δ 176.3, 175.0, 174.6, 173.3, 60.1, 58.9, 43.1, 41.9 (CHB (broad)), 39.6, 37.2, 30.4, 30.2, 26.29, 26.27, 25.9, 22.3, 15.9, 15.7, 11.2. HR-MS (ESI/TOF) calcd for C_19_H_35_BN_4_O_6_Na [M+Na]^+^ 449.2547, found 449.2549.

#### ((2R,8S,11S)-11-cyclopentyl-8-isobutyl-4,7,10,13-tetraoxo-3,6,9,12-tetraazatetra-decan-2-yl)boronic acid (2g)

Was prepared according to general procedure F from a solution of **13g** (97 mg, 0.173 mmol) in MeOH/*n*-hexane (6.6 mL) was treated with isobutylboronic acid (71 mg, 0.70 mmol) and 1 M HCl (425 μL). Purification by flash chromatography on reversed phase silica gel provided **2g** (58 mg, 79%) as a white solid.

^1^H NMR (400 MHz, Methanol-*d_4_*) δ 4.27 − 4.17 (m, 2H), 4.12 (d, *J* = 9.3 Hz, 1H), 3.97 (dd, *J* = 17.6, 1.0 Hz, 1H), 2.66 (q, *J* = 7.0 Hz, 1H), 2.20 (h, *J* = 8.9 Hz, 1H), 1.98 (s, 3H), 1.87 − 1.77 (m, 1H), 1.74 − 1.51 (m, 8H), 1.43 − 1.26 (m, 2H), 1.12 (d, *J* = 7.2 Hz, 3H), 0.97 (d, *J* = 6.4 Hz, 3H), 0.93 (d, *J* = 6.4 Hz, 3H). ^13^C NMR (101 MHz, Methanol-*d_4_*) δ 176.3, 175.4, 175.1, 173.5, 59.0, 53.9, 43.1, 41.9 (CHB (broad)), 40.9, 39.8, 30.4, 30.2, 26.2, 25.9, 25.8, 23.3, 22.3, 22.1, 15.9. HR-MS (ESI/TOF) calcd for C_19_H_35_BN_4_O_6_Na [M+Na]^+^ 449.2547, found 449.2554.

#### ((2R,8S,11S)-8-benzyl-11-cyclopentyl-4,7,10,13-tetraoxo-3,6,9,12-tetraazatetradecan-2-yl)boronic acid (2h)

Was prepared according to general procedure F from a solution of **13h** (102 mg, 0.172 mmol) in MeOH/*n*-hexane (6.6 mL), isobutylboronic acid (70 mg, 0.69 mmol) and 1 M HCl (425 μL). Purification by flash chromatography on reversed phase silica gel provided **2h** (59 mg, 75%) as a white solid.

^1^H NMR (400 MHz, Methanol-*d_4_*) δ 7.32 − 7.16 (m, 5H), 4.46 (dd, *J* = 8.4, 6.9 Hz, 1H), 4.16 (dd, *J* = 17.6, 1.8 Hz, 1H), 4.06 (d, *J* = 9.3 Hz, 1H), 3.86 (dd, *J* = 17.6, 1.0 Hz, 1H), 3.16 (dd, *J* = 13.7, 6.8 Hz, 1H), 3.00 (dd, *J* = 13.8, 8.5 Hz, 1H), 2.66 (q, *J* = 7.0 Hz, 1H), 2.09 (h, *J* = 9.3 Hz, 1H), 1.96 (s, 3H), 1.81 − 1.71 (m, 1H), 1.66 − 1.46 (m, 5H), 1.29 − 1.20 (m, 2H), 1.11 (d, *J* = 7.2 Hz, 3H). ^13^C NMR (101 MHz, Methanol-*d_4_*) δ 176.2, 174.8, 174.2, 173.6, 138.2, 130.3, 129.5, 127.9, 59.2, 56.8, 43.0, 41.8 (CHB (broad)), 39.7, 37.8, 30.3, 30.1, 26.1, 25.8, 22.4, 16.0 HR-MS (ESI/TOF) calcd for C_19_H_35_BN_4_O_6_Na [M+Na]^+^ 483.2391, found 483.2398.

#### ((S)-2-((tert-butoxycarbonyl)amino)-2-cyclopentylacetyl)-L-isoleucylglycine (14)

Was prepared according to general procedure D from starting material **10f** (940 mg, 2.13 mmol), LiOH (510 mg, 21.3 mmol) in THF:H_2_O (52.5 mL) the reaction was stirred for 6 h at room temperature. Water (15 mL) was added and the reaction mixture was acidified with 1M HCl solution and the product was extracted with chloroform (4×20mL). Organic phase was washed with brine, dried over Na_2_SO_4_, filtered and evaporated in vacuo to provide product **14** (878 mg, 99%) as a white solid.

^1^H NMR (400 MHz, Methanol-*d_4_*) δ 4.29 (d, *J* = 7.7 Hz, 1H), 3.98 (d, *J* = 17.8 Hz, 1H), 3.91 − 3.79 (m, 2H), 2.24 − 2.10 (m, 1H), 1.92 − 1.73 (m, 2H), 1.70 − 1.51 (m, 6H), 1.44 (s, 9H), 1.38 − 1.26 (m, 2H), 1.26 − 1.14 (m, 1H), 0.97 (d, *J* = 6.8 Hz, 3H), 0.90 (t, *J* = 7.4 Hz, 3H). ^13^C NMR (101 MHz, Methanol-*d_4_*) δ 174.8, 173.8, 172.5, 158.0, 80.6, 60.3, 58.8, 43.1, 41.7, 38.4, 30.3, 28.7, 26.3, 26.0, 25.7, 15.8, 11.4. HR-MS (ESI/TOF) calcd for C_20_H_35_N_3_O_6_Na [M+Na]^+^ 436.2424, found 436.2425.

#### tert-Butyl ((S)-1-cyclopentyl-2-(((2S,3S)-3-methyl-1-oxo-1-((2-oxo-2-(((R)-1-((3aS,4S, 6S,7aR)-3a,5,5-trimethylhexahydro-4,6-methanobenzo[d][1,3,2]dioxaborol-2-yl) ethyl)amino)ethyl)amino)pentan-2-yl)amino)-2-oxoethyl)carbamate (15)

Was prepared according to general procedure D from acid **14** (770 mg, 1.86 mmol), **12** (580 mg, 2.23 mmol), DMAP (68 mg, 0.56 mmol), NMM (820 μl, 7.46 mmol) and T3P (1.7 mL, 2.84 mmol) in of anhydrous CHCl_3_ (10 mL). Crude mixture was purified by flash chromatography on silica gel eluting with 0−5% MeOH in EtOAc to provide **15** (842 mg, 73%) as a solid compound.

^1^H NMR (400 MHz, Methanol-*d_4_*) δ 4.22 − 4.13 (m, 2H), 4.08 (d, *J* = 8.0 Hz, 1H), 3.99 − 3.84 (m, 2H), 2.65 (q, *J* = 7.3 Hz, 1H), 2.38 − 2.29 (m, 1H), 2.23 − 2.08 (m, 2H), 1.95 (t, *J* = 5.5 Hz, 1H), 1.89 − 1.72 (m, 4H), 1.71 − 1.50 (m, 6H), 1.47 − 1.40 (m, 10H), 1.35 (s, 3H), 1.34 − 1.17 (m, 6H), 1.16 (d, *J* = 7.3 Hz, 3H), 0.98 − 0.88 (m, 6H), 0.87 (s, 3H). ^13^C NMR (101 MHz, Methanol-*d_4_*) δ 175.7, 175.5, 174.3, 158.0, 84.3, 80.6, 77.3, 59.9, 53.6, 43.2, 41.3, 40.0 (overlaps with CHB (broad)), 39.2, 37.7, 37.5, 30.2, 29.6, 28.7, 27.8, 27.5, 26.3, 26.2, 26.0, 24.5, 16.4, 15.8, 11.2. HR-MS (ESI/TOF) calcd for C_32_H_56_BN_4_O_7_ [M+H]^+^ 619.4242, found 619.4258.

#### N-((S)-1-cyclopentyl-2-(((2S,3S)-3-methyl-1-oxo-1-((2-oxo-2-(((R)-1-((3aS,4S,6S, 7aR)-3a,5,5-trimethylhexahydro-4,6-methanobenzo[d][1,3,2]dioxaborol-2-yl)ethyl) amino) ethyl)amino)pentan-2-yl)amino)-2-oxoethyl)quinoline-2-carboxamide (16a)

Prepared according to general procedure B from starting material **15** (100 mg, 0.162 mmol) and 4 M HCl in dioxane (170 μL) in CHCl_3_ (2 mL). After a full conversion of the starting material, the intermediate was subjected to the coupling reaction according to general procedure A using quinaldic acid (28 mg, 0.162 mmol), EDC·HCl (37 mg, 0.193 mmol), HOBt (24 mg, 0.178 mmol) and DIPEA (84 μL, 0.486 mmol) in CHCl_3_ (5 mL), were added and mixture was stirred overnight at room temperature. The product was purified by flash chromatography on silica gel eluting with 0−5% MeOH in EtOAc to provide **16a** (69 mg, 63%) as an amorphous solid.

^1^H NMR (400 MHz, Methanol-*d_4_*) δ 8.48 (dd, *J* = 8.6, 1.0 Hz, 1H), 8.20 (d, *J* = 8.5 Hz, 1H), 8.14 (dd, *J* = 8.6, 1.1 Hz, 1H), 8.00 (dd, *J* = 8.2, 1.7 Hz, 1H), 7.84 (ddd, *J* = 8.5, 6.8, 1.4 Hz, 1H), 7.69 (ddd, *J* = 8.2, 6.9, 1.3 Hz, 1H), 4.66 (d, *J* = 8.0 Hz, 1H), 4.25 (dd, *J* = 17.6, 1.7 Hz, 1H), 4.16 (dd, *J* = 8.6, 2.3 Hz, 1H), 4.05 (d, *J* = 8.5 Hz, 1H), 3.93 (dd, *J* = 17.6, 0.9 Hz, 1H), 2.73 (q, *J* = 7.2 Hz, 1H), 2.46 (h, *J* = 7.7 Hz, 1H), 2.37 − 2.29 (m, 1H), 2.18 − 2.10 (m, 1H), 1.94 (t, *J* = 5.5 Hz, 1H), 1.89 − 1.75 (m, 5H), 1.74 − 1.57 (m, 5H), 1.53 − 1.42 (m, 3H), 1.35 (s, 3H), 1.28 (s, 3H), 1.26 − 1.21 (m, 4H), 0.95 (d, *J* = 6.8 Hz, 3H), 0.92 (t, *J* = 7.4 Hz, 3H), 0.86 (s, 3H). ^13^C NMR (101 MHz, Methanol-*d_4_*) δ 175.7, 174.6, 174.4, 166.2, 150.4, 148.0, 139.2, 131.7, 130.9, 130.7, 129.5, 129.1, 119.5, 84.4, 77.4, 60.5, 57.7, 53.6, 44.5, 41.3, 40.1, 39.6 (CHB (broad)), 39.2, 37.6, 37.1, 30.3, 29.8, 29.6, 27.8, 27.5, 26.5, 26.3, 26.1, 24.5, 16.4, 15.7, 11.2. HR-MS (ESI/TOF) calcd for C_37_H_53_BN_5_O_6_ [M+H]^+^ 674.4089, found 674.4103.

#### N-((S)-1-cyclopentyl-2-(((2S,3S)-3-methyl-1-oxo-1-((2-oxo-2-(((R)-1-((3aS,4S, 6S,7aR)-3a,5,5-trimethylhexahydro-4,6-methanobenzo[d][1,3,2]dioxaborol-2-yl)ethyl)amino)ethyl)amino)pentan-2-yl)amino)-2-oxoethyl)-6-phenylpicolinamide (16b)

Prepared according to general procedure B from starting material **15** (103 mg, 0.167 mmol) and 4 M HCl in dioxane (170 μL) in CHCl_3_ (2 mL). After a full conversion of the starting material, the residue was subjected to the coupling reaction according to general procedure A using 6-phenylpyridine-2-carboxylic acid (34 mg, 0.171 mmol), EDC·HCl (38 mg, 0.198 mmol), HOBt (25 mg, 0.185 mmol) and DIPEA (60 μL, 0.347 mmol) in CHCl_3_ (5 mL). The product was purified by flash chromatography on silica gel eluting with 0−5% MeOH in EtOAc to provide **16b** (74 mg, 64%) as an amorphous solid.

^1^H NMR (400 MHz, Methanol-*d_4_*) δ 8.13 − 8.00 (m, 5H), 7.54 − 7.42 (m, 3H), 4.70 (d, *J* = 7.4 Hz, 1H), 4.28 (dd, *J* = 17.6, 1.7 Hz, 1H), 4.15 (dd, *J* = 8.6, 2.3 Hz, 1H), 4.02 (d, *J* = 8.5 Hz, 1H), 3.91 (dd, *J* = 17.6, 0.8 Hz, 1H), 2.69 (q, *J* = 7.2 Hz, 1H), 2.44 (h, *J* = 8.7 Hz, 1H), 2.36 − 2.28 (m, 1H), 2.18 − 2.09 (m, 1H), 1.94 (t, *J* = 5.5 Hz, 1H), 1.88 − 1.74 (m, 5H), 1.71 − 1.57 (m, 5H), 1.52 − 1.41 (m, 3H), 1.35 (s, 3H), 1.27 (s, 3H), 1.26 − 1.20 (m, 1H), 1.14 (d, *J* = 7.3 Hz, 3H), 0.98 − 0.90 (m, 6H), 0.86 (s, 3H). ^13^C NMR (101 MHz, Methanol-*d_4_*) δ 175.7, 174.7, 174.4, 166.1, 157.6, 150.2, 139.9, 139.4, 130.7, 130.0, 127.9, 124.6, 121.6, 84.4, 77.3, 60.6, 57.1, 53.6, 44.7, 41.3, 40.1, 39.6 (CHB (broad)), 39.2, 37.6, 36.9, 30.3, 29.6, 29.5, 27.8, 27.5, 26.6, 26.4, 26.1, 24.5, 16.5, 15.7, 11.2. HR-MS (ESI/TOF) calcd for C_39_H_55_BN_5_O_6_ [M+H]^+^ 700.4245, found 700.4265.

#### N-((S)-1-cyclopentyl-2-(((2S,3S)-3-methyl-1-oxo-1-((2-oxo-2-(((R)-1-((3aS,4S,6S, 7aR)-3a,5,5-trimethylhexahydro-4,6-methanobenzo[d][1,3,2]dioxaborol-2-yl)ethyl) amino)ethyl)amino)pentan-2-yl)amino)-2-oxoethyl)-3,5-dimethylbenzamide (16c)

Prepared according to general procedure B from starting material **15** (100 mg, 0.162 mmol) and 4 M HCl in dioxane (160 μL) in CHCl_3_ (3 mL). After a full conversion of the starting material, the intermediate was subjected to the coupling reaction according to general procedure A using 3,5-dimethylbenzoic acid (25 mg, 0.166 mmol), EDC·HCl (37 mg, 0.193 mmol), HOBt (24 mg, 0.178 mmol) and DIPEA (84 μL, 0.489 mmol) in CHCl_3_ (5 mL). The product was purified by flash chromatography on silica gel eluting with 0−5% MeOH in EtOAc to provide **16c** (57 mg, 64%) as an amorphous solid.

^1^H NMR (400 MHz, Methanol-*d_4_*) δ 7.45 − 7.40 (m, 2H), 7.22 − 7.17 (m, 1H), 4.40 (d, *J* = 10.0 Hz, 1H), 4.20 (dd, *J* = 17.5, 1.6 Hz, 1H), 4.15 (dd, *J* = 8.6, 2.3 Hz, 1H), 4.07 (d, *J* = 8.2 Hz, 1H), 3.95 (dd, *J* = 17.5, 1.0 Hz, 1H), 2.67 (q, *J* = 7.3 Hz, 1H), 2.44 − 2.28 (m, 8H), 2.17 − 2.09 (m, 1H), 1.94 (t, *J* = 5.6 Hz, 1H), 1.90 − 1.56 (m, 10H), 1.46 − 1.33 (m, 6H), 1.28 (s, 3H), 1.26 − 1.19 (m, 1H), 1.17 (d, *J* = 7.3 Hz, 3H), 0.97 − 0.90 (m, 6H), 0.87 (s, 3H). ^13^C NMR (101 MHz, Methanol-*d_4_*) δ 175.7, 175.0, 174.3, 170.8, 139.4, 135.4, 134.3, 126.2, 84.4, 77.3, 60.2, 59.4, 53.6, 43.0, 41.3, 40.0, 39.8 (CHB (broad)), 39.2, 37.7, 37.3, 30.7, 30.4, 29.6, 27.8, 27.5, 26.4, 26.3, 26.0, 24.5, 21.3, 16.4, 15.8, 11.2. HR-MS (ESI/TOF) calcd for C_36_H_56_BN_4_O_6_ [M+H]^+^ 651.4293, found 651.4313.

#### N-((S)-1-cyclopentyl-2-(((2S,3S)-3-methyl-1-oxo-1-((2-oxo-2-(((R)-1-((3aS,4S, 6S,7aR)-3a,5,5-trimethylhexahydro-4,6-methanobenzo[d][1,3,2]dioxaborol-2-yl) ethyl)amino)ethyl)amino)pentan-2-yl)amino)-2-oxoethyl)benzamide (16d)

Prepared according to general procedure B from starting material **15** (100 mg, 0.162 mmol) and 4 M HCl in dioxane (160 μL) in CHCl_3_ (3 mL). After a full conversion of the starting material, the intermediate was subjected to the coupling reaction according to general procedure A: the residue was dissolved in CHCl_3_ (5 mL), benzoic acid (20 mg, 0.164 mmol), EDC·HCl (37 mg, 0.193 mmol), HOBt (24 mg, 0.178 mmol) and DIPEA (84 μL, 0.489 mmol) were added and mixture was stirred overnight at room temperature. The residue was purified by flash chromatography on silica gel eluting with 0−5% MeOH in EtOAc to provide **16d** (62 mg, 62%) as an amorphous solid.

^1^H NMR (400 MHz, Methanol-*d_4_*) δ 7.84 − 7.79 (m, 2H), 7.58 − 7.52 (m, 1H), 7.50 − 7.44 (m, 2H), 4.41 (d, *J* = 10.0 Hz, 1H), 4.20 (dd, *J* = 17.5, 1.6 Hz, 1H), 4.15 (dd, *J* = 8.6, 2.3 Hz, 1H), 4.07 (d, *J* = 8.2 Hz, 1H), 3.96 (dd, *J* = 17.5, 1.0 Hz, 1H), 2.67 (q, *J* = 7.3 Hz, 1H), 2.47 − 2.27 (m, 2H), 2.17 − 2.09 (m, 1H), 1.95 (t, *J* = 5.5 Hz, 1H), 1.92 − 1.55 (m, 10H), 1.47 − 1.34 (m, 6H), 1.28 (s, 3H), 1.27 − 1.19 (m, 1H), 1.17 (d, *J* = 7.4 Hz, 3H), 0.98 − 0.89 (m, 6H), 0.87 (s, 3H). ^13^C NMR (101 MHz, Methanol-*d_4_*) δ 175.8 174.9, 174.3, 170.5, 135.4, 132.8, 129.6, 128.5, 84.3, 77.3, 60.2, 59.6, 53.6, 42.9, 41.4, 40.0, 39.8 (CHB (broad)), 39.2, 37.7, 37.3, 30.8, 30.4, 29.6, 27.8, 27.5, 26.4, 26.3, 26.0, 24.5, 16.4, 15.8, 11.2. HR-MS (ESI/TOF) calcd for C_34_H_52_BN_4_O_6_ [M+H]^+^ 623.3980, found 623.4008.

#### (2S,3S)-2-((S)-2-cyclopentyl-2-isobutyramidoacetamido)-3-methyl-N-(2-oxo-2-(((R)-1-((3aS,4S,6S,7aR)-3a,5,5-trimethylhexahydro-4,6-methanobenzo[d][1,3,2]dioxa-borol-2-yl)ethyl)amino)ethyl)pentanamide (16e)

Prepared according to general procedure B from starting material **15** (103 mg, 0.17 mmol) and 4 M HCl in dioxane (170 μL) in CHCl_3_ (2 mL). After a full conversion of the starting material, the intermediate was subjected to the acylation reaction according to general procedure C using isobutyric anhydride (41 μL, 0.25 mmol) and DIPEA (58 μL, 0.33 mmol) in dry CHCl_3_ (5 mL). The crude mixture was purified by flash chromatography on silica gel eluting with 0−5% MeOH in EtOAc to provide **16e** (73 mg, 75%) as an amorphous solid.

^1^H NMR (400 MHz, Methanol-*d_4_*) δ 4.23 − 4.13 (m, 3H), 4.03 (d, *J* = 8.1 Hz, 1H), 3.94 (dd, *J* = 17.5, 1.0 Hz, 1H), 2.64 (q, *J* = 7.2 Hz, 1H), 2.53 (hept, *J* = 6.9 Hz, 1H), 2.40 − 2.28 (m, 1H), 2.30 − 2.17 (m, 1H), 2.18 − 2.08 (m, 1H), 1.94 (t, *J* = 5.5 Hz, 1H), 1.89 − 1.75 (m, 4H), 1.72 − 1.52 (m, 6H), 1.44 (d, *J* = 10.3 Hz, 1H), 1.35 (s, 3H), 1.33 − 1.19 (m, 6H), 1.17 (d, *J* = 7.5 Hz, 3H), 1.11 (d, *J* = 6.9 Hz, 3H), 1.09 (d, *J* = 6.8 Hz, 3H), 0.94 (d, *J* = 6.8 Hz, 3H), 0.92 (t, *J* = 7.4 Hz, 3H), 0.87 (s, 3H). ^13^C NMR (101 MHz, Methanol-*d_4_*) δ 180.1, 175.8, 174.9, 174.3, 84.3, 77.3, 60.1, 58.5, 53.6, 42.9, 41.4, 40.0, 39.8 (CHB (broad)), 39.2, 37.7, 37.3, 35.9, 30.4, 30.3, 29.6, 27.8, 27.5, 26.3, 26.3, 26.0, 24.5, 20.1, 19.7, 16.4, 15.8, 11.2. HR-MS (ESI/TOF) calcd for C_31_H_54_BN_4_O_6_ [M+H]^+^ 589.4136, found 589.4151.

#### (2S,3S)-2-((S)-2-cyclopentyl-2-pivalamidoacetamido)-3-methyl-N-(2-oxo-2-(((R)-1-((3aS,4S,6S,7aR)-3a,5,5-trimethylhexahydro-4,6-methanobenzo[d][1,3,2]dioxaborol-2-yl)ethyl)amino)ethyl)pentanamide (16f)

Deprotection followed general procedure B: starting material **15** (92 mg, 0.15 mmol) and 4 M HCl in dioxane (150 μL) in CHCl_3_ (2 mL). After a full conversion of the starting material, the intermediate was subjected to the acylation reaction according to general procedure C using trimethyl acetic anhydride (45 μL, 0.22 mmol) and DIPEA (52 μL, 0.30 mmol) in dry CHCl_3_ (5 mL). The crude mixture was purified by flash chromatography on silica gel eluting with 0−5% MeOH in EtOAc to provide **16f** (60 mg, 67%) as an amorphous solid.

^1^H NMR (400 MHz, Methanol-*d_4_*) δ 4.28 − 4.13 (m, 3H), 4.05 (d, *J* = 8.1 Hz, 1H), 3.95 (dd, *J* = 17.4, 1.0 Hz, 1H), 2.65 (q, *J* = 7.2 Hz, 1H), 2.38 − 2.25 (m, 2H), 2.18 − 2.09 (m, 1H), 1.95 (t, *J* = 5.5 Hz, 1H), 1.89 − 1.73 (m, 4H), 1.72 − 1.52 (m, 6H), 1.44 (d, *J* = 10.4 Hz, 1H), 1.36 (s, 3H), 1.34 − 1.21 (m, 6H), 1.20 (s, 9H), 1.17 (d, *J* = 7.3 Hz, 3H), 0.96 − 0.89 (m, 6H), 0.87 (s, 3H). ^13^C NMR (101 MHz, Methanol-*d_4_*) δ 181.1, 175.6, 174.9, 174.2, 84.3, 77.3, 60.0, 58.5, 53.6, 43.1, 41.4, 40.0, 39.8 (overlaps with CHB (broad)), 39.2, 37.7, 37.4, 30.4, 30.3, 29.6, 27.8, 27.5, 26.3, 26.24, 26.0, 24.6, 16.4, 15.8, 11.2. HR-MS (ESI/TOF) calcd for C_32_H_56_BN_4_O_6_ [M+H]^+^ 603.4293, found 602.4308.

#### N-((S)-1-cyclopentyl-2-(((2S,3S)-3-methyl-1-oxo-1-((2-oxo-2-(((R)-1-((3aS,4S,6S, 7aR)-3a,5,5-trimethylhexahydro-4,6-methanobenzo[d][1,3,2]dioxaborol-2-yl)ethyl) amino)ethyl)amino)pentan-2-yl)amino)-2-oxoethyl)cyclobutanecarboxamide (16g)

Prepared according to general procedure B from starting material **15** (113 mg, 0.183 mmol) and 4 M HCl in dioxane (180 μL) in CHCl_3_ (2 mL). After a full conversion of the starting material, the intermediate was subjected to general procedure A using cyclobutanecarboxylic acid (20 μL, 0.21 mmol), EDC·HCl (42 mg, 0.22 mmol), HOBt (28 mg, 0.21 mmol) and DIPEA (63 μL, 0.37 mmol) in CHCl_3_ (5 mL). The product was purified by flash chromatography on silica gel eluting with 0−5% MeOH in EtOAc to provide **16g** (60 mg, 55%) as an amorphous solid.

^1^H NMR (400 MHz, Methanol-*d_4_*) δ 4.23 − 4.13 (m, 3H), 4.03 (d, *J* = 8.2 Hz, 1H), 3.94 (dd, *J* = 17.6, 1.0 Hz, 1H), 3.17 (pd, *J* = 8.4, 1.1 Hz, 1H), 2.64 (q, *J* = 7.3 Hz, 1H), 2.38 − 2.29 (m, 1H), 2.27 − 2.18 (m, 3H), 2.18 − 2.08 (m, 3H), 2.05 − 1.91 (m, 2H), 1.90 − 1.73 (m, 5H), 1.71 − 1.50 (m, 6H), 1.44 (d, *J* = 10.3 Hz, 1H), 1.35 (s, 3H), 1.33 − 1.19 (m, 6H), 1.16 (d, *J* = 7.3 Hz, 3H), 0.94 (d, *J* = 6.8 Hz, 3H), 0.92 (t, *J* = 7.5 Hz, 3H), 0.87 (s, 3H).^13^C NMR (101 MHz, Methanol-*d_4_*) δ 177.7, 175.8, 175.0, 174.3, 84.3, 77.3, 60.1, 58.6, 53.6, 43.0, 41.4, 40.5, 40.0, 39.8 (CHB (broad)), 39.2, 37.7, 37.3, 30.4, 30.3, 29.6, 27.8, 27.5, 26.3, 26.2, 26.04, 25.96, 24.5, 19.1, 16.4, 15.8, 11.2. HR-MS (ESI/TOF) calcd for C_32_H_54_BN_4_O_6_ [M+H]^+^ 601.4136, found 601.4141

#### (2S,3S)-2-((S)-2-cyclopentyl-2-(thiazole-2-sulfonamido)acetamido)-3-methyl-N-(2-oxo-2-(((R)-1-((3aS,4S,6S,7aR)-3a,5,5-trimethylhexahydro-4,6-methanobenzo[d]-[1,3,2]dioxaborol-2-yl)ethyl)amino)ethyl)pentanamide (16h)

Prepared according to general procedure B from starting material **15** (100 mg, 0.162 mmol) and 4 M HCl in dioxane (160 μL) in CHCl_3_ (3 mL). After a full conversion of the starting material, the intermediate was subjected to the sulfonylation reaction: the residue was dissolved in CHCl_3_ (5 mL), thiazole-2-sulfonyl chloride (35 μL, 0.25 mmol, 1.5 equiv) and DIPEA (60 μL, 0.35 mmol, 2.0 equiv) were added and mixture was stirred for 2 h at room temperature, then washed with 5% KHSO_4_ (5 mL), with sat. NaHCO_3_ (5 mL), and brine (10 mL). Organic phase was dried over Na_2_SO_4_, filtered and evaporated in vacuo. The producy was purified by flash chromatography on silica gel eluting with 0−5% MeOH in EtOAc to provide **16h** (87 mg, 81%) as an amorphous solid.

^1^H NMR (400 MHz, Methanol-*d_4_*) δ 7.95 (d, *J* = 3.1 Hz, 1H), 7.92 (d, *J* = 3.1 Hz, 1H), 4.19 (dd, *J* = 17.5, 1.7 Hz, 1H), 4.15 (dd, *J* = 8.6, 2.3 Hz, 1H), 3.97 (d, *J* = 8.6 Hz, 1H), 3.90 (dd, *J* = 17.6, 0.9 Hz, 1H), 3.85 (d, *J* = 8.6 Hz, 1H), 2.62 (q, *J* = 7.3 Hz, 1H), 2.39 − 2.28 (m, 1H), 2.25 − 2.07 (m, 2H), 1.94 (t, *J* = 5.6 Hz, 1H), 1.88 − 1.82 (m, 1H), 1.81 − 1.71 (m, 2H), 1.70 − 1.46 (m, 7H), 1.43 (d, *J* = 10.4 Hz, 1H), 1.40 − 1.33 (m, 4H), 1.28 (s, 3H), 1.23 − 1.13 (m, 5H), 0.93 (t, *J* = 7.4 Hz, 3H), 0.91 (d, *J* = 6.8 Hz, 3H), 0.87 (s, 3H). ^13^C NMR (101 MHz, Methanol-*d_4_*) δ 175.75, 175.73, 174.2, 174.1, 168.0, 145.1, 126.4, 84.3, 77.3, 61.6, 60.4, 53.6, 44.0, 41.3, 40.0, 39.8 (CHB (broad)), 39.2, 37.7, 37.1, 29.94, 29.86, 29.6, 27.8, 27.5, 26.5, 26.3, 25.8, 24.5, 16.4, 15.7, 11.3. HR-MS (ESI/TOF) calcd for C_30_H_49_BN_5_O_7_S_2_ [M+H]^+^ 666.3166, found 666.3162.

#### (2S,3S)-2-((S)-2-(benzylamino)-2-cyclopentylacetamido)-3-methyl-N-(2-oxo-2-(((R)-1-((3aS,4S,6S,7aR)-3a,5,5-trimethylhexahydro-4,6-methanobenzo [d][1,3,2]dioxa-borol-2-yl)ethyl)amino)ethyl)pentanamide **(16i)**

Prepared according to general procedure B from starting material **15** (172 mg, 0.278 mmol) and 4 M HCl in dioxane (280 μL) in CHCl_3_ (5 mL). After a full conversion of the starting material, the intermediate was subjected to the reductive amination reaction: the residue was dissolved in 2 mL 2,2,2-trifluoroethanol (TFE) and cooled to 0 °C, triethylamine (43 μL, 0.31 mmol, 1.1 equiv) and benzaldehyde (60 μL, 0.59 mmol, 2.0 equiv) were added and mixture was allowed to warm up to room temperature overnight. Then it was cooled to 0 °C and NaBH_4_ (53 mg, 1.40 mmol, 5.0 equiv) was added, followed by few drops of MeOH. Reaction was stirred 1 h, then acidified with 5% KHSO_4_ and extracted with CHCl_3_ (3×). Organic phase was dried over Na_2_SO_4_, filtered and evaporated *in vacuo*. The residue was purified by flash chromatography on reversed phase silica gel eluting with 10−100 % MeOH in H_2_O to provide **16i** (83 mg, 49%) as an amorphous solid.

^1^H NMR (600 MHz, Methanol-*d_4_*) δ 7.35 − 7.29 (m, 4H), 7.25 − 7.22 (m, 1H), 4.20 (dd, *J* = 17.5, 1.6 Hz, 1H), 4.15 (dd, *J* = 8.6, 2.3 Hz, 1H), 4.12 (d, *J* = 8.2 Hz, 1H), 3.96 (dd, *J* = 17.5, 0.9 Hz, 1H), 3.78 (d, *J* = 13.1 Hz, 1H), 3.57 (d, *J* = 13.1 Hz, 1H), 2.98 (d, *J* = 8.1 Hz, 1H), 2.67 (q, *J* = 7.2 Hz, 1H), 2.36 − 2.29 (m, 1H), 2.16 − 2.10 (m, 1H), 2.07 − 1.99 (m, 1H), 1.94 (t, *J* = 5.6 Hz, 1H), 1.87 − 1.76 (m, 2H), 1.66 − 1.48 (m, 4H), 1.43 (d, *J* = 10.3 Hz, 1H), 1.39 − 1.32 (m, 4H), 1.28 (s, 3H), 1.27 − 1.21 (m, 1H), 1.16 (d, *J* = 7.3 Hz, 3H), 0.97 (d, *J* = 6.8 Hz, 3H), 0.94 (t, *J* = 7.5 Hz, 3H), 0.87 (s, 3H). ^13^C NMR (151 MHz, Methanol-*d_4_*) δ 177.6, 175.7, 174.4, 141.0, 129.5, 129.4, 128.2, 84.4, 77.3, 66.9, 59.5, 53.6, 53.0, 44.9, 41.3, 40.1, 39.6 (CHB (broad)), 39.2, 37.6, 37.4, 30.6, 30.1, 29.6, 27.8, 27.5, 26.3, 26.2, 26.1, 24.5, 16.4, 15.9, 11.1. HR-MS (ESI/TOF) calcd for C_34_H_54_BN_4_O_5_ [M+H]^+^ 609.4187, found 609.4200.

#### (2S,3S)-2-((S)-2-cyclopentyl-2-((pyridin-2-ylmethyl)amino)acetamido)-3-methyl-N-(2-oxo-2-(((R)-1-((3aS,4S,6S,7aR)-3a,5,5-trimethylhexahydro-4,6-methano benzo[d]-[1,3,2]dioxaborol-2-yl)ethyl)amino)ethyl)pentanamide (16j)

Prepared according to general procedure B from starting material **15** (150 mg, 0.24 mmol) and 4 M HCl in dioxane (250 μL) in CHCl_3_ (5 mL). After a full conversion of the starting material, the intermediate was subjected to the reductive amination reaction: the residue was dissolved in TFE (2 mL) and cooled to 0 °C, triethylamine (40 μL, 0.29 mmol) and pyridine-2-carboxaldehyde (46 μL, 0.48 mmol) were added and mixture was allowed to warm up to room temperature overnight. Then it was cooled to 0 °C and NaBH_4_ (46 mg, 1.22 mmol) was added, followed by few drops of MeOH. Reaction was stirred 1h, then acidified with 5% KHSO_4_ and extracted with CHCl_3_ (3×). Organic phase was dried over Na_2_SO_4_, filtered and evaporated in vacuo. The product was purified by flash chromatography on reversed phase silica gel eluting with 10−100 % MeOH in H_2_O to provide **16j** (87 mg, 59%) as an amorphous solid. ^1^H NMR (400 MHz, Methanol-*d_4_*) δ 8.48 (ddd, *J* = 4.9, 1.8, 1.0 Hz, 1H), 7.80 (td, *J* = 7.7, 1.8 Hz, 1H), 7.51 (dt, *J* = 7.9, 1.2 Hz, 1H), 7.30 (ddd, *J* = 7.6, 5.0, 1.3 Hz, 1H), 4.20 (dd, *J* = 17.5, 1.6 Hz, 1H), 4.15 (dd, *J* = 8.6, 2.3 Hz, 1H), 4.12 (d, *J* = 8.1 Hz, 1H), 3.95 (dd, *J* = 17.4, 1.0 Hz, 1H), 3.89 (d, *J* = 14.2 Hz, 1H), 3.74 (d, *J* = 14.2 Hz, 1H), 3.00 (d, *J* = 8.0 Hz, 1H), 2.66 (q, *J* = 7.2 Hz, 1H), 2.37 − 2.28 (m, 1H), 2.18 − 2.04 (m, 2H), 1.94 (t, *J* = 5.5 Hz, 1H), 1.88 − 1.75 (m, 4H), 1.69 − 1.49 (m, 6H), 1.44 (d, *J* = 10.3 Hz, 1H), 1.42 − 1.36 (m, 2H), 1.35 (s, 3H), 1.28 (s, 3H), 1.26 − 1.18 (m, 1H), 1.16 (d, *J* = 7.3 Hz, 3H), 1.00 − 0.89 (m, 6H), 0.87 (s, 3H). ^13^C NMR (101 MHz, Methanol-*d_4_*) δ 177.4, 175.7, 174.4, 160.6, 149.7, 138.7, 124.2, 123.7, 84.4, 77.3, 67.4, 59.6, 54.0, 53.6, 44.9, 41.3, 40.1, 39.6 (CHB (broad)), 39.2, 37.7, 37.4, 30.5, 30.2, 29.6, 27.8, 27.5, 26.4, 26.2, 26.1, 24.5, 16.4, 15.9, 11.2. HR-MS (ESI/TOF) calcd for C_33_H_53_BN_5_O_5_ [M+H]^+^ 610.4140, found 610.4152

#### ((3S,6S,12R)-6-((S)-sec-butyl)-3-cyclopentyl-1,4,7,10-tetraoxo-1-(quinolin-2-yl)-2,5,8,11-tetraazatridecan-12-yl)boronic acid (3a)

Prepared according to general procedure F from a solution of **16a** (59 mg, 0.088 mmol) in MeOH/*n*-hexane (3.4 mL), isobutylboronic acid (36 mg, 0.35 mmol) and 1 M HCl (220 μL). Purification by flash chromatography on reversed phase silica gel provided **3a** (35 mg, 74%) as a white solid.

^1^H NMR (400 MHz, Methanol-*d_4_*) δ 8.48 (dd, *J* = 8.6, 1.0 Hz, 1H), 8.19 (d, *J* = 8.5 Hz, 1H), 8.13 (dd, *J* = 8.5, 1.0 Hz, 1H), 8.00 (dd, *J* = 8.2, 1.8 Hz, 1H), 7.83 (ddd, *J* = 8.5, 6.9, 1.5 Hz, 1H), 7.69 (ddd, *J* = 8.2, 6.9, 1.3 Hz, 1H), 4.67 (d, *J* = 8.1 Hz, 1H), 4.27 (dd, *J* = 17.6, 1.7 Hz, 1H), 4.08 (d, *J* = 8.5 Hz, 1H), 3.98 (dd, *J* = 17.6, 0.9 Hz, 1H), 2.72 (q, *J* = 6.8 Hz, 1H), 2.53 − 2.39 (m, 1H), 1.89 − 1.74 (m, 3H), 1.73 − 1.54 (m, 5H), 1.53 − 1.40 (m, 2H), 1.29 − 1.21 (m, 1H), 1.19 (d, *J* = 7.3 Hz, 3H), 0.96 (d, *J* = 6.9 Hz, 3H), 0.92 (t, *J* = 7.5 Hz, 3H). ^13^C NMR (101 MHz, Methanol-*d_4_*) δ 176.3, 174.7, 174.5, 166.2, 150.4, 147.9, 139.2, 131.7, 130.9, 130.7, 129.5, 129.1, 119.5, 60.4, 57.7, 44.5, 41.8 (CHB (broad)), 39.7, 37.1, 30.3, 29.8, 26.4, 26.3, 26.0, 16.0, 15.7, 11.2. HR-MS (ESI/TOF) calcd for C_27_H_38_BN_5_O_6_Na [M+Na]^+^ 562.2813, found 562.2822

#### ((3S,6S,12R)-6-((S)-sec-butyl)-3-cyclopentyl-1,4,7,10-tetraoxo-1-(6-phenylpyridin-2-yl)-2,5,8,11-tetraazatridecan-12-yl)boronic acid (3b)

Prepared according to general procedure F from a solution of **16b** (130 mg, 0.186 mmol) in MeOH/*n*-hexane (7.2 mL), isobutylboronic acid (76 mg, 0.75 mmol) and 1 M HCl (460 μL). Purification by flash chromatography on reversed phase silica gel provided **3b** (74 mg, 70%) as a white solid.

^1^H NMR (400 MHz, Methanol-*d_4_*) δ 8.13 − 7.99 (m, 5H), 7.53 − 7.43 (m, 3H), 4.71 (d, *J* = 7.6 Hz, 1H), 4.29 (dd, *J* = 17.6, 1.7 Hz, 1H), 4.06 (d, *J* = 8.5 Hz, 1H), 3.97 (dd, *J* = 17.6, 0.9 Hz, 1H), 2.69 (q, *J* = 7.2 Hz, 1H), 2.44 (h, *J* = 8.6 Hz, 1H), 1.90 − 1.72 (m, 3H), 1.71 − 1.53 (m, 5H), 1.53 − 1.41 (m, 2H), 1.31 − 1.17 (m, 1H), 1.11 (d, *J* = 7.2 Hz, 3H), 0.96 (d, *J* = 6.8 Hz, 3H), 0.91 (t, *J* = 7.5 Hz, 3H). ^13^C NMR (101 MHz, Methanol-*d_4_*) δ 176.2, 174.7, 174.5, 166.1, 157.6, 150.2, 139.9, 139.3, 130.7, 130.0, 127.9, 124.6, 121.6, 60.4, 57.2, 44.7, 41.7 (CHB (broad)), 39.6, 37.0, 30.2, 29.6, 26.5, 26.4, 26.1, 16.1, 15.6, 11.1. HR-MS (ESI/TOF) calcd for C_29_H_40_BN_5_O_6_Na [M+Na]^+^ 588.2969, found 588.2975.

#### ((3S,6S,12R)-6-((S)-sec-butyl)-3-cyclopentyl-1-(3,5-dimethylphenyl)-1,4,7,10-tetra-oxo-2,5,8,11-tetraazatridecan-12-yl)boronic acid (3c)

Prepared according to general procedure F from a solution of **16c** (48 mg, 0.074 mmol) in MeOH/*n*-hexane (2.8 mL), isobutylboronic acid (30 mg, 0.29 mmol) and 1 M HCl (185 μL). Purification by flash chromatography on reversed phase silica gel provided **3c** (27 mg, 71%) as a white solid.

^1^H NMR (400 MHz, Methanol-*d_4_*) δ 7.44 − 7.41 (m, 2H), 7.20 − 7.18 (m, 1H), 4.40 (d, *J* = 10.0 Hz, 1H), 4.22 (dd, *J* = 17.5, 1.6 Hz, 1H), 4.10 (d, *J* = 8.2 Hz, 1H), 4.00 (dd, *J* = 17.5, 0.9 Hz, 1H), 2.67 (q, *J* = 7.0 Hz, 1H), 2.45 − 2.31 (m, 7H), 1.93 − 1.80 (m, 2H), 1.77 − 1.54 (m, 6H), 1.47 − 1.32 (m, 2H), 1.30 − 1.17 (m, 1H), 1.12 (d, *J* = 7.2 Hz, 3H), 0.95 (d, *J* = 6.8 Hz, 3H), 0.92 (t, *J* = 7.4 Hz, 3H). ^13^C NMR (101 MHz, Methanol-*d_4_*) δ 176.3, 175.0, 174.5, 170.8, 139.4, 135.3, 134.3, 126.2, 60.0, 59.5, 43.0, 41.9 (CHB (broad)), 39.6, 37.4, 30.7, 30.5, 26.3, 26.3, 25.9, 21.29, 21.27, 21.2, 15.9, 15.7, 11.2. HR-MS (ESI/TOF) calcd for C_26_H_41_BN_4_O_6_Na [M+Na]^+^ 539.3017, found 539.3034.

#### ((3S,6S,12R)-6-((S)-sec-butyl)-3-cyclopentyl-1-(3,5-dimethylphenyl)-1,4,7,10-tetra-oxo-2,5,8,11-tetraazatridecan-12-yl)boronic acid (3d)

Prepared according to general procedure F from a solution of **16d** (53 mg, 0.085 mmol) in MeOH/*n*-hexane (3.2 mL), isobutylboronic acid (35 mg, 0.34 mmol) and 1 M HCl (210 μL). Purification by flash chromatography on reversed phase silica gel provided **3d** (28 mg, 67%) as a white solid.

^1^H NMR (400 MHz, Methanol-*d_4_*) δ 7.84 − 7.80 (m, 2H), 7.57 − 7.52 (m, 1H), 7.49 − 7.44 (m, 2H), 4.41 (d, *J* = 10.0 Hz, 1H), 4.22 (dd, *J* = 17.5, 1.7 Hz, 1H), 4.10 (d, *J* = 8.2 Hz, 1H), 3.99 (dd, *J* = 17.5, 1.0 Hz, 1H), 2.67 (q, *J* = 6.7 Hz, 1H), 2.47 − 2.32 (m, 1H), 1.95 − 1.80 (m, 2H), 1.76 − 1.53 (m, 6H), 1.47 − 1.33 (m, 2H), 1.30 − 1.17 (m, 1H), 1.12 (d, *J* = 7.2 Hz, 3H), 0.95 (d, *J* = 6.8 Hz, 3H), 0.92 (t, *J* = 7.5 Hz, 3H). ^13^C NMR (101 MHz, Methanol-*d_4_*) δ 176.2, 175.0, 174.5, 170.5, 135.4, 132.8, 129.6, 128.5, 60.1, 59.6, 43.0, 42.0 (CHB (broad)), 39.6, 37.4, 30.8, 30.5, 26.35, 26.26, 25.9, 15.9, 15.7, 11.2. HR-MS (ESI/TOF) calcd for C_24_H_37_BN_4_O_6_Na [M+Na]^+^ 511.2704, found 511.2705.

#### ((2R,8S,11S)-8-((S)-sec-butyl)-11-cyclopentyl-14-methyl-4,7,10,13-tetraoxo-3,6,9,12-tetraazapentadecan-2-yl)boronic acid (3e)

Prepared according to general procedure F from a solution of **16e** (67 mg, 0.11 mmol) in MeOH/*n*-hexane (4.4 mL), isobutylboronic acid (46 mg, 0.45 mmol) and 1 M HCl (280 μL). Purification by flash chromatography on reversed phase silica gel provided **3e** (34 mg, 66%) as a white solid.

^1^H NMR (400 MHz, Methanol-*d_4_*) δ 4.23 (dd, *J* = 17.5, 1.7 Hz, 1H), 4.18 (d, *J* = 9.7 Hz, 1H), 4.06 (d, *J* = 8.1 Hz, 1H), 3.99 (dd, *J* = 17.6, 1.0 Hz, 1H), 2.67 (q, *J* = 6.9 Hz, 1H), 2.53 (hept, *J* = 6.9 Hz, 1H), 2.24 (h, *J* = 8.7 Hz, 1H), 1.88 − 1.76 (m, 2H), 1.72 − 1.51 (m, 6H), 1.40 − 1.16 (m, 3H), 1.12 (d, *J* = 7.2 Hz, 3H), 1.11 (d, *J* = 6.8 Hz, 3H), 1.09 (d, *J* = 6.9 Hz, 3H), 0.96 − 0.89 (m, 6H). ^13^C NMR (101 MHz, Methanol-*d_4_*) δ 180.2, 176.4, 175.0, 174.5, 59.9, 58.6, 43.0, 42.0 (CHB (broad)), 39.6, 37.4, 35.8, 30.4, 30.3, 26.3, 26.2, 25.9, 20.1, 19.7, 15.9, 15.7, 11.2. HR-MS (ESI/TOF) calcd for C_21_H_39_BN_4_O_6_Na [M+Na]^+^ 477.2860, found 477.2869.

#### ((2R,8S,11S)-8-((S)-sec-butyl)-11-cyclopentyl-14,14-dimethyl-4,7,10,13-tetraoxo-3,6,9,12-tetraazapentadecan-2-yl)boronic acid (3f)

Prepared according to general procedure F from a solution of **16f** (58 mg, 0.096 mmol) in MeOH/*n*-hexane (3.7 mL), isobutylboronic acid (40 mg, 0.39 mmol) and 1 M HCl (240 μL). Purification by flash chromatography on reversed phase silica gel provided **3f** (24 mg, 53%) as a white solid.

^1^H NMR (400 MHz, Methanol-*d_4_*) δ 4.24 (d, *J* = 9.5 Hz, 1H), 4.22 (dd, *J* = 17.5, 1.7 Hz, 1H), 4.07 (d, *J* = 8.0 Hz, 1H), 3.98 (dd, *J* = 17.5, 1.0 Hz, 1H), 2.67 (q, *J* = 7.2 Hz, 1H), 2.30 (dq, *J* = 16.7, 8.6 Hz, 1H), 1.89 − 1.73 (m, 2H), 1.72 − 1.52 (m, 6H), 1.38 − 1.30 (m, 1H), 1.29 − 1.17 (m, 11H), 1.12 (d, *J* = 7.2 Hz, 3H), 0.97 − 0.87 (m, 6H). ^13^C NMR (101 MHz, Methanol-*d_4_*) δ 181.1, 176.3, 174.9, 174.4, 59.9, 58.6, 43.1, 41.9 (CHB (broad)), 39.8, 39.6, 37.5, 30.4, 30.3, 27.8, 26.3, 26.2, 25.9, 15.9, 15.7, 11.2. HR-MS (ESI/TOF) calcd for C_22_H_41_BN_4_O_6_Na [M+Na]^+^ 491.3030, found 491.3040.

#### ((3S,6S,12R)-6-((S)-sec-butyl)-1-cyclobutyl-3-cyclopentyl-1,4,7,10-tetraoxo-2,5,8,11-tetraazatridecan-12-yl)boronic acid (3g)

Prepared according to general procedure F from a solution of **16g** (58 mg, 0.097 mmol) in MeOH/*n*-hexane (3.7 mL), isobutylboronic acid (40 mg, 0.39 mmol) and 1 M HCl (240 μL). Purification by flash chromatography on reversed phase silica gel provided **3g** (36 mg, 80%) as a white solid.

^1^H NMR (400 MHz, Methanol-*d_4_*) δ 4.23 (dd, *J* = 17.6, 1.7 Hz, 1H), 4.18 (d, *J* = 9.5 Hz, 1H), 4.05 (d, *J* = 8.1 Hz, 1H), 3.99 (dd, *J* = 17.6, 1.0 Hz, 1H), 3.17 (pd, *J* = 8.4, 1.0 Hz, 1H), 2.67 (q, *J* = 7.2 Hz, 1H), 2.31 − 2.17 (m, 3H), 2.20 − 2.05 (m, 2H), 2.06 − 1.90 (m, 1H), 1.91 − 1.73 (m, 3H), 1.70 − 1.49 (m, 6H), 1.39 − 1.17 (m, 3H), 1.12 (d, *J* = 7.2 Hz, 3H), 0.94 (d, *J* = 6.8 Hz, 3H), 0.92 (t, *J* = 7.5 Hz, 3H). ^13^C NMR (101 MHz, Methanol-*d_4_*) δ 177.7, 176.4, 175.0, 174.5, 60.0, 58.7, 43.0, 42.1 (CHB (broad)), 40.4, 39.6, 37.4, 30.4, 30.3, 26.3, 26.25, 26.22, 26.0, 25.9, 19.1, 15.9, 15.7, 11.2. HR-MS (ESI/TOF) calcd for C_22_H_39_BN_4_O_6_Na [M+Na]^+^ 489.2860, found 489.2865.

#### ((R)-1-(2-((2S,3S)-2-((S)-2-cyclopentyl-2-(thiazole-2-sulfonamido)acetamido)-3-methylpentanamido)acetamido)ethyl)boronic acid (3h)

Prepared according to general procedure F from a solution of **16h** (84 mg, 0.126 mmol) in MeOH/*n*-hexane (5.0 mL), isobutylboronic acid (52 mg, 0.51 mmol) and 1 M HCl (320 μL). Purification by flash chromatography on reversed phase silica gel provided **3h** (50 mg, 75%) as a white solid.

^1^H NMR (400 MHz, Methanol-*d_4_*) δ 7.96 (d, *J* = 3.2 Hz, 1H), 7.92 (d, *J* = 3.2 Hz, 1H), 4.22 (dd, *J* = 17.6, 1.7 Hz, 1H), 3.99 − 3.91 (m, 2H), 3.86 (d, *J* = 8.5 Hz, 1H), 2.64 (q, *J* = 7.2 Hz, 1H), 2.17 (h, *J* = 8.6 Hz, 1H), 1.82 − 1.72 (m, 1H), 1.70 − 1.46 (m, 7H), 1.43 − 1.32 (m, 1H), 1.26 − 1.14 (m, 2H), 1.11 (d, *J* = 7.3 Hz, 3H), 0.98 − 0.89 (m, 6H). ^13^C NMR (101 MHz, Methanol-*d_4_*) δ 176.3, 174.4, 174.1, 168.0, 145.1, 126.4, 61.6, 60.3, 44.1, 42.0 (CHB (broad)), 39.6, 37.1, 29.95, 29.91, 26.5, 26.3, 25.8, 15.9, 15.6, 11.3. HR-MS (ESI/TOF) calcd for C_20_H_34_BN_5_O_7_S_2_Na [M+Na]^+^ 554.1890, found 554.1889.

#### ((3S,6S,12R)-6-((S)-sec-butyl)-3-cyclopentyl-4,7,10-trioxo-1-phenyl-2,5,8,11-tetra-azatridecan-12-yl)boronic acid (3i)

Prepared according to general procedure F from a solution of **16i** (83 mg, 0.136 mmol) in MeOH/*n*-hexane (5.2 mL), isobutylboronic acid (56 mg, 0.55 mmol) and 1 M HCl (340 μL). Purification by flash chromatography on reversed phase silica gel provided **3i** (44 mg, 65%) as a white solid.

^1^H NMR (400 MHz, Methanol-*d_4_*) δ 7.37 − 7.27 (m, 4H), 7.28 − 7.19 (m, 1H), 4.22 (d, *J* = 17.5 Hz, 1H), 4.15 (d, *J* = 8.1 Hz, 1H), 3.99 (d, *J* = 17.5 Hz, 1H), 3.79 (d, *J* = 13.1 Hz, 1H), 3.57 (d, *J* = 13.1 Hz, 1H), 2.99 (d, *J* = 8.0 Hz, 1H), 2.68 (q, *J* = 7.2 Hz, 1H), 2.03 (h, *J* = 7.6 Hz, 1H), 1.90 − 1.77 (m, 2H), 1.69 − 1.44 (m, 6H), 1.43 − 1.29 (m, 2H), 1.29 − 1.18 (m, 1H), 1.12 (d, *J* = 7.3 Hz, 3H), 1.00 − 0.92 (m, 6H). ^13^C NMR (101 MHz, Methanol-*d_4_*) δ 177.6, 176.0, 174.5, 140.9, 129.5, 129.4, 128.2, 66.9, 59.4, 53.0, 44.9, 41.7 (CHB (broad)), 39.8, 37.4, 30.6, 30.1, 26.3, 26.2, 26.0, 16.0, 15.8, 11.1. HR-MS (ESI/TOF) calcd for C_24_H_39_BN_4_O_5_Na [M+Na]^+^ 497.2911, found 497.2912.

#### ((3S,6S,12R)-6-((S)-sec-butyl)-3-cyclopentyl-4,7,10-trioxo-1-(pyridin-2-yl)-2,5,8,11-tetraazatridecan-12-yl)boronic acid (3j)

Prepared according to general procedure F from a solution of **16j** (78 mg, 0.128 mmol) in MeOH/*n*-hexane (5.0 mL), isobutylboronic acid (52 mg, 0.51 mmol) and 1 M HCl (320 μL). Purification by flash chromatography on reversed phase silica gel provided **3j** (38 mg, 62%) as a white solid.

^1^H NMR (400 MHz, Methanol-*d_4_*) δ 8.48 (ddd, *J* = 5.0, 1.8, 1.0 Hz, 1H), 7.80 (td, *J* = 7.6, 1.8 Hz, 1H), 7.51 (dt, *J* = 8.0, 1.2 Hz, 1H), 7.30 (ddd, *J* = 7.6, 4.9, 1.4 Hz, 1H), 4.22 (dd, *J* = 17.5, 1.6 Hz, 1H), 4.14 (d, *J* = 7.9 Hz, 1H), 3.99 (dd, *J* = 17.5, 1.0 Hz, 1H), 3.89 (d, *J* = 14.2 Hz, 1H), 3.74 (d, *J* = 14.2 Hz, 1H), 3.01 (d, *J* = 8.1 Hz, 1H), 2.66 (q, *J* = 7.2 Hz, 1H), 2.15 − 2.01 (m, 1H), 1.90 − 1.79 (m, 2H), 1.68 − 1.49 (m, 6H), 1.44 − 1.35 (m, 2H), 1.27 − 1.17 (m, 1H), 1.11 (d, *J* = 7.2 Hz, 3H), 0.97 (d, *J* = 6.8 Hz, 3H), 0.93 (t, *J* = 7.5 Hz, 3H). ^13^C NMR (101 MHz, Methanol-*d_4_*) δ 177.4, 176.2, 174.6, 160.6, 149.7, 138.7, 124.2, 123.7, 67.4, 59.6, 54.0, 45.0, 41.8 (CHB (broad)), 39.6, 37.5, 30.5, 30.2, 26.3, 26.2, 26.1, 15.9, 15.8, 11.2. HR-MS (ESI/TOF) calcd for C_23_H_38_BN_5_O_5_Na [M+Na]^+^ 498.2864, found 498.2870.

#### tert-butyl 3-(4,4,5,5-tetramethyl-1,3,2-dioxaborolan-2-yl)propanoate (18)

Was prepared according to literature procedure.^33^ CuCl (21 mg, 0.212 mmol), NaO*t*-Bu (60 mg, 0.624 mmol) and DPEphos ligand (8.1 mg, 0.015 mmol) were placed in an oven-dried flask and THF (5.5 mL) was added under argon. The reaction mixture was stirred for 30 min at room temperature and then, bis(pinacolato)diboron in THF (4 mL) was added. The reaction mixture was stirred for 10 min and *tert*-butyl acrylate (1 mL, 6.89 mmol) was added, followed by MeOH (600 μL, 14.83 mmol). The reaction was sealed and stirred until no starting material was detected by TLC. The reaction mixture was filtered through a pad of Celite, concentrated and purified by flash chromatography on silica gel eluting with hexane:EtOAc 8:1 to provide boronic ester **18** as a colorless liquid (1.665 g, 94 %).

^1^H NMR (300 MHz, Chloroform-*d*) δ 2.34 (t, *J* = 7.5 Hz, 2H), 1.43 (s, 9H), 1.23 (s, 12H), 0.96 (t, *J* = 7.5 Hz, 2H). The spectral data was identical to that reported in the literature.^38,39^

#### tert-butyl 3-((3aS,4S,6S,7aR)-3a,5,5-trimethylhexahydro-4,6-methanobenzo[d][1,3,2] dioxaborol-2-yl)propanoate (19)

To the solution of boronic ester **18** (1.353 g, 5.28 mmol, 1.0 equiv) in THF (10 mL) was added (+)-pinanediol (1.35 g, 7.93 mmol, 1.5 equiv). The mixture was stirred for 15 hours at room temperature. Then the solution was evaporated and crude mixture was purified by flash chromatography on silica gel eluting with 20:1 − 8:1 hexane:EtOAc to provide **19** (1.615 g, 99%) as a colorless oil.

^1^H NMR (300 MHz,, Chloroform-*d*) δ 4.26 (dd, *J* = 8.8, 2.0 Hz, 1H), 2.40 − 2.26 (m, 3H), 2.24 − 2.14 (m, 1H), 2.05 − 1.99 (m, 1H), 1.93 − 1.78 (m, 2H), 1.43 (s, 9H), 1.37 (s, 3H), 1.28 (s, 3H), 1.18 (d, *J* = 10.8 Hz, 1H), 1.02 (t, *J* = 7.5 Hz, 2H), 0.83 (s, 3H). ^13^C NMR (101 MHz, Chloroform-*d*) δ 174.2, 85.7, 80.0, 77.9, 51.4, 39.6, 38.3, 35.6, 30.2, 28.7, 28.3, 27.2, 26.5, 24.2. Spectral data are in accordance with those reported in the literature.^28^

#### tert-butyl (S)-4-chloro-4-((3aS,4S,6S,7aR)-3a,5,5-trimethylhexahydro-4,6-methano benzo[d][1,3,2]dioxaborol-2-yl)butanoate (20)

Matteson homologation was used to synthesize α-chlorinated boronates.^40^ A stirred solution of anhydrous dichloromethane (1.4 mL, 21.8 mmol, 5.0 equiv) in anhydrous tetrahydrofuran (25 mL) was cooled in liquid nitrogen/ethanol bath to −100 °C and treated with 2.5 M *n*-buthyl lithium (2.6 mL, 6.5 mmol, 1.5 equiv) over a period of 30 min (under argon). A solution of pinanediol alkylboronate **19** (1.318 g, 4.28 mmol, 1.0 equiv) in anhydrous tetrahydrofuran (15 mL) was then added dropwise to the reaction mixture and stirred for 30 min at -100°C. Afterwards 1 M ZnCl_2_ (7.7 mL, 7.7 mmol, 1.8 equiv) was added slowly to mixture. The cooling bath was removed and the reaction was allowed to warm to room temperature. After stirring overnight diethyl ether was added to the reaction mixture and the suspension obtained was washed with a saturated ammonium chloride solution. The solvent was evaporated and the oily residue was dissolved in diethyl ether, washed with brine and organic phase was dried over Na_2_SO_4_, filtered and evaporated in vacuo. The residue was purified by flash chromatography on silica gel eluting with 20:1 − 8:1 hexane:EtOAc to provide boronate **20** (1.124 g, 74%) as a colorless oil.

^1^H NMR (400 MHz, Chloroform-*d*) δ 4.36 (dd, *J* = 8.8, 2.0 Hz, 1H), 3.52 (dd, *J* = 9.2, 5.2 Hz, 1H), 2.53 − 2.41 (m, 2H), 2.40 − 2.30 (m, 1H), 2.28 − 2.13 (m, 2H), 2.10 − 2.00 (m, 2H), 1.95 − 1.86 (m, 2H), 1.44 (s, 9H), 1.42 (s, 3H), 1.29 (s, 3H), 1.16 (d, *J* = 11.1 Hz, 1H), 0.84 (s, 3H). ^13^C NMR (101 MHz, Chloroform-*d*) δ 172.3, 86.8, 80.4, 78.6, 51.2, 42.5 (CHB (broad)), 39.4, 38.2, 35.2, 33.0, 29.3, 28.4, 28.1, 27.0, 26.4, 26.3, 24.0. Spectral data are in accordance with those reported in the literature.^28^

#### tert-butyl (R)-4-(bis(trimethylsilyl)amino)-4-((3aS,4S,6S,7aR)-3a,5,5-trimethylhexa-hydro-4,6-methanobenzo[d][1,3,2]dioxaborol-2-yl)butanoate (21)

To the solution of α-chloroboronic acid ester **20** (1.124 g, 3.15 mmol, 1 equiv) in anhydrous tetrahydrofuran (20 mL) 1 M lithium bis(trimethylsilyl)amide (3.5 mL, 3.5 mmol, 1.1 equiv) was slowly added at −78 ° C. The mixture was allowed to warm up and stirred overnight at room temperature. The solvent was removed in vacuo and hexane (50 mL) was added to the residue. The inorganic precipitates were filtered off through a pad of Celite, and then washed with additional amount of hexane, and filtrate was evaporated to provide **21** (1.31 g, 86%) as a colorless oil.

^1^H NMR (400 MHz, Chloroform-*d*) δ 4.28 (dd, *J* = 8.7, 1.9 Hz, 1H), 2.55 (dd, *J* = 9.1, 6.8 Hz, 1H), 2.44 − 2.14 (m, 4H), 2.02 (dd, *J* = 6.1, 4.9 Hz, 1H), 1.99 − 1.92 (m, 1H), 1.92 − 1.81 (m, 2H), 1.77 − 1.66 (m, 1H), 1.44 (s, 9H), 1.37 (s, 3H), 1.28 (s, 3H), 1.11 (d, *J* = 10.8 Hz, 1H), 0.83 (s, 3H), 0.11 (s, 18H). ^13^C NMR (101 MHz, Chloroform-*d*) δ 173.8, 85.6, 79.9, 78.5, 51.5, 39.6, 38.3, 35.5, 33.8, 30.5, 28.5, 28.3, 27.2, 26.5, 24.1, 3.1. (CHB not visible). GC-MS: 424.4 (M − *t*-Bu), 408.4 (M − Me_3_Si).

#### Ethyl ((S)-2-cyclopentyl-2-(quinoline-2-carboxamido)acetyl)-L-isoleucylglycinate (22a)

Prepared according to general procedure B from starting material **10f** (400 mg, 0.91 mmol) and 4 M HCl in dioxane (900 μL) in CHCl_3_ (5 mL). After a full conversion of the starting material, the intermediate was subjected to the coupling reaction according to general procedure A from quinoline-2-carboxylic acid (157 mg, 0.91 mmol), EDC·HCl (208 mg, 1.09 mmol), HOBt (135 mg, 1.0 mmol) and DIPEA (470 μL, 2.72 mmol) using in dry CHCl_3_ (15 mL). The product was purified by flash chromatography on silica gel eluting with 1:1:1 hexane:EtOAc:CHCl_3_ − 1:1 EtOAc:CHCl_3_ to provide **22a** (358 mg, 80%) as an amorphous white solid.

^1^H NMR (400 MHz, Chloroform-*d*) δ 8.75 (d, *J* = 8.2 Hz, 1H), 8.31 (dd, *J* = 8.5, 0.7 Hz, 1H), 8.28 (d, *J* = 8.5 Hz, 1H), 8.13 (dd, *J* = 8.5, 1.1 Hz, 1H), 7.87 (dd, *J* = 8.2, 1.8 Hz, 1H), 7.77 (ddd, *J* = 8.5, 6.8, 1.4 Hz, 1H), 7.62 (ddd, *J* = 8.1, 6.9, 1.3 Hz, 1H), 6.93 (d, *J* = 8.6 Hz, 1H), 6.75 (t, *J* = 5.4 Hz, 1H), 4.56 (t, *J* = 8.4 Hz, 1H), 4.41 (dd, *J* = 8.6, 6.3 Hz, 1H), 4.19 (q, *J* = 7.2 Hz, 2H), 4.13 − 3.95 (m, 2H), 2.57 (h, *J* = 8.5 Hz, 1H), 2.03 − 1.94 (m, 1H), 1.92 − 1.80 (m, 2H), 1.72 − 1.54 (m, 4H), 1.53 − 1.38 (m, 3H), 1.26 (t, *J* = 7.2 Hz, 3H), 1.17 − 1.04 (m, 1H), 0.90 (d, *J* = 6.8 Hz, 3H), 0.81 (t, *J* = 7.4 Hz, 3H). ^13^C NMR (101 MHz, Chloroform-*d*) δ 171.8, 171.3, 169.7, 165.1, 149.1, 146.7, 137.7, 130.3, 130.1, 129.6, 128.2, 127.8, 118.9, 61.6, 58.1, 58.0, 42.0, 41.5, 36.8, 29.8, 29.2, 25.6, 25.3, 24.8, 15.6, 14.3, 11.5. HR-MS (ESI/TOF) calcd for C_27_H_37_N_4_O_5_ [M+H]^+^ 497.2764, found 497.2770.

#### Ethyl ((S)-2-cyclopentyl-2-(6-phenylpicolinamido)acetyl)-L-isoleucylglycinate (22b)

Prepared according to general procedure B from starting material **10f** (400 mg, 0.91 mmol) and 4 M HCl in dioxane (900 μL) in CHCl_3_ (5 mL). After a full conversion of the starting material, the residue was subjected to the coupling reaction according to general procedure A using 6-phenylpicolinic acid (181 mg, 0.91 mmol), EDC·HCl (208 mg, 1.09 mmol), HOBt (135 mg, 1.0 mmol) and DIPEA (470 μL, 2.72 mmol) in dry CHCl_3_ (15 mL). The product was purified by flash chromatography on silica gel eluting with 1:1:1 hexane:EtOAc:CHCl_3_ − 1:1 EtOAc:CHCl_3_ to provide **22b** (380 mg, 80%) as an amorphous white solid.

^1^H NMR (400 MHz, Chloroform-*d*) δ 8.68 (d, *J* = 8.1 Hz, 1H), 8.13 (dd, *J* = 7.1, 1.5 Hz, 1H), 8.04 − 7.97 (m, 2H), 7.97 − 7.86 (m, 2H), 7.54 − 7.43 (m, 3H), 6.93 (d, *J* = 8.6 Hz, 1H), 6.76 (t, *J* = 5.4 Hz, 1H), 4.54 (t, *J* = 8.2 Hz, 1H), 4.40 (dd, *J* = 8.6, 6.3 Hz, 1H), 4.19 (q, *J* = 7.1 Hz, 2H), 4.13 − 3.95 (m, 2H), 2.55 (h, *J* = 8.3 Hz, 1H), 2.03 − 1.95 (m, 1H), 1.90 − 1.79 (m, 2H), 1.71 − 1.55 (m, 4H), 1.52 − 1.36 (m, 3H), 1.26 (t, *J* = 7.2 Hz, 3H), 1.16 − 1.04 (m, 1H), 0.91 (d, *J* = 6.8 Hz, 3H), 0.83 (t, *J* = 7.4 Hz, 3H). ^13^C NMR (101 MHz, Chloroform-*d*) δ 171.7, 171.3, 169.7, 165.1, 156.3, 149.1, 138.5, 138.3, 129.7, 129.1, 127.1, 123.5, 120.9, 61.6, 57.95, 57.90, 41.9, 41.5, 36.8, 29.8, 29.1, 25.6, 25.4, 24.8, 15.6, 14.3, 11.5. HR-MS (ESI/TOF) calcd for C_29_H_39_N_4_O_5_ [M+H]^+^ 523.2920, found 523.2925.

#### Ethyl ((S)-2-cyclopentyl-2-(3,5-dimethylbenzamido)acetyl)-L-isoleucylglycinate (22c)

Prepared according to general procedure B from starting material **10f** (300 mg, 0.68 mmol) and 4 M HCl in dioxane (680 μL) in CHCl_3_ (5 mL). After a full conversion of the starting material, the residue was subjected to the coupling reaction according to general procedure A using 3,5-dimethylbenzoic acid (102 mg, 0.68 mmol), EDC·HCl (156 mg, 0.81 mmol), HOBt (101 mg, 0.75 mmol) and DIPEA (350 μL, 2.02 mmol) in dry CHCl_3_ (20 mL). The product was purified by flash chromatography on silica gel eluting with 1:1:1 hexane:EtOAc:CHCl_3_ − 1:1 EtOAc:CHCl_3_ to provide **22c** (302 mg, 94%) as an amorphous white solid.

^1^H NMR (400 MHz, Chloroform-*d*) δ 7.37 (s, 2H), 7.13 (s, 1H), 6.85 (d, *J* = 8.7 Hz, 1H), 6.80 (d, *J* = 8.1 Hz, 1H), 6.69 (t, *J* = 5.4 Hz, 1H), 4.54 (t, *J* = 8.6 Hz, 1H), 4.39 (dd, *J* = 8.6, 6.4 Hz, 1H), 4.20 (q, *J* = 7.2 Hz, 2H), 4.10 (dd, *J* = 18.2, 5.6 Hz, 1H), 3.97 (dd, *J* = 18.3, 4.9 Hz, 1H), 2.43 − 2.30 (m, 7H), 2.00 − 1.90 (m, 1H), 1.84 − 1.74 (m, 2H), 1.68 − 1.46 (m, 5H, overlaps with H_2_O in CHCl_3_), 1.43 − 1.32 (m, 2H), 1.27 (t, *J* = 7.2 Hz, 3H), 1.19 − 1.06 (m, 1H), 0.91 (d, *J* = 6.8 Hz, 3H), 0.85 (t, *J* = 7.4 Hz, 3H). ^13^C NMR (101 MHz, Chloroform-*d*) δ 172.0, 171.2, 169.7, 168.2, 138.5, 134.0, 133.5, 125.0, 61.7, 58.0, 57.9, 42.4, 41.5, 37.1, 29.8, 29.2, 25.5, 25.2, 24.9, 21.3, 15.6, 14.3, 11.5. HR-MS (ESI/TOF) calcd for C_26_H_40_N_3_O_5_ [M+H]^+^ 474.2968, found 474.2951.

#### ((S)-2-cyclopentyl-2-(quinoline-2-carboxamido)acetyl)-L-isoleucylglycine (23a)

Pre-pared according to general procedure D from starting material **22a** (345 mg, 0.69 mmol) and LiOH (166 mg, 6.9 mmol) in THF:H_2_O (10.5 mL). Product **23a** (314 mg, 97%) was isolated as white solid compound.

^1^H NMR (400 MHz, Methanol-*d_4_*) δ 8.48 (d, *J* = 8.6 Hz, 1H), 8.18 (t, *J* = 8.5 Hz, 1H), 8.00 (dd, *J* = 8.2, 1.7 Hz, 1H), 7.84 (ddd, *J* = 8.5, 6.9, 1.5 Hz, 1H), 7.69 (ddd, *J* = 8.2, 6.9, 1.2 Hz, 1H), 4.60 (d, *J* = 8.7 Hz, 1H), 4.32 (d, *J* = 8.0 Hz, 1H), 3.98 (d, *J* = 17.6 Hz, 1H), 3.84 (d, *J* = 17.6 Hz, 1H), 2.46 (h, *J* = 8.6 Hz, 1H), 1.93 − 1.82 (m, 2H), 1.81 − 1.66 (m, 3H), 1.66 − 1.54 (m, 3H), 1.54 − 1.42 (m, 2H), 1.25 − 1.13 (m, 1H), 0.96 (d, *J* = 6.8 Hz, 3H), 0.88 (t, *J* = 7.5 Hz, 3H). ^13^C NMR (101 MHz, Methanol-*d_4_*) δ 173.8, 173.7 (overlaps two C=O), 166.3, 150.5, 148.0, 139.1, 131.6, 130.9, 130.8, 129.4, 129.0, 119.5, 59.1, 58.6, 44.2, 42.2, 38.1, 30.4, 30.2, 26.3, 26.0, 25.9, 15.8, 11.3. HR-MS (ESI/TOF) calcd for C_25_H_33_N_4_O_5_ [M+H]^+^ 469.2451, found 469.2449.

#### ((S)-2-cyclopentyl-2-(6-phenylpicolinamido)acetyl)-L-isoleucylglycine (23b)

Prepared according to general procedure D from starting material **22b** (295 mg, 0.56 mmol) and LiOH (124 mg, 5.2 mmol) in THF:H_2_O (11 mL). Product **23b** (273 mg, 98%) was isolated as white solid compound.

^1^H NMR (400 MHz, Methanol-*d_4_*) δ 8.16 − 8.11 (m, 2H), 8.10 − 8.01 (m, 3H), 7.55 − 7.43 (m, 3H), 4.61 (d, *J* = 8.3 Hz, 1H), 4.32 (d, *J* = 8.0 Hz, 1H), 3.99 (d, *J* = 17.6 Hz, 1H), 3.83 (d, *J* = 17.6 Hz, 1H), 2.45 (h, *J* = 8.3 Hz, 1H), 1.93 − 1.74 (m, 4H), 1.71 − 1.54 (m, 4H), 1.52 − 1.41 (m, 2H), 1.25 − 1.13 (m, 1H), 0.97 (d, *J* = 6.8 Hz, 3H), 0.88 (t, *J* = 7.5 Hz, 3H). ^13^C NMR (101 MHz, Methanol-*d_4_*) δ 173.8, 173.7, 172.9, 166.2, 157.6, 150.3, 139.9, 139.4, 130.7, 129.9, 128.0, 124.5, 121.6, 59.1, 58.2, 44.2, 42.0, 38.1, 30.4, 30.0, 26.4, 26.1, 25.9, 15.8, 11.3.HR-MS (ESI/TOF) calcd for C_27_H_35_N_4_O_5_ [M+H]^+^ 495.2607, found 495.2619.

#### ((S)-2-cyclopentyl-2-(3,5-dimethylbenzamido)acetyl)-L-isoleucylglycine (23c)

Prepared according to general procedure D from starting material **22c** (282 mg, 0.60 mmol) and LiOH (143 mg, 6.0 mmol) in THF:H_2_O (31.5 mL). Product **23c** (225 mg, 85%) was isolated as white solid compound.

^1^H NMR (400 MHz, Dimethylsulfoxide-*d_6_*) δ 12.50 (s, 1H), 8.35 (d, *J* = 8.5 Hz, 1H), 8.27 (t, *J* = 5.8 Hz, 1H), 7.75 (d, *J* = 9.1 Hz, 1H), 7.44 (d, *J* = 1.7 Hz, 2H), 7.16 (s, 1H), 4.32 (dd, *J* = 9.7, 8.4 Hz, 1H), 4.24 (dd, *J* = 9.1, 7.2 Hz, 1H), 3.79 (dd, *J* = 17.5, 5.9 Hz, 1H), 3.70 (dd, *J* = 17.5, 5.8 Hz, 1H), 2.38 − 2.27 (m, 7H), 1.79 − 1.66 (m, 2H), 1.64 − 1.53 (m, 3H), 1.52 − 1.40 (m, 3H), 1.40 − 1.31 (m, 1H), 1.31 − 1.19 (m, 1H), 1.15 − 1.01 (m, 1H), 0.83 (d, *J* = 6.8 Hz, 3H), 0.79 (t, *J* = 7.4 Hz, 3H). ^13^C NMR (101 MHz, Dimethylsulfoxide-*d_6_*) δ 171.2, 171.1, 171.0, 166.6, 137.4, 134.4, 132.5, 125.2, 57.7, 56.4, 41.3, 40.6, 37.0, 29.3, 28.9, 25.0, 24.6, 24.1, 20.8, 15.2, 11.0. HR-MS (ESI/TOF) calcd for C_24_H_36_N_3_O_5_ [M+H]^+^ 446.2655, found 446.2655.

#### tert-Butyl (3S,6S,12R)-6-((S)-sec-butyl)-3-cyclopentyl-1,4,7,10-tetraoxo-1-(quinolin-2-yl)-12-((3aS,4S,6S,7aR)-3a,5,5-trimethylhexahydro-4,6-methanobenzo[d][1,3,2] dioxaborol-2-yl)-2,5,8,11-tetraazapentadecan-15-oate (24a)

Prepared according to general procedure G from acid **23a** (120 mg, 0.256 mmol), **21** (154 mg, 0.320 mmol), DMAP (10 mg, 0.077 mmol), NMM (90 μl, 0.82 mmol) and T3P (310 μL, 0.52 mmol) in anhydrous CHCl_3_ (3 mL). Crude mixture was purified by flash chromatography on silica gel eluting with 0−5% MeOH in EtOAc to provide **24a** (111 mg, 55%) as an amorphous solid.

^1^H NMR (400 MHz, Methanol-*d_4_*) δ 8.47 (dd, *J* = 8.6, 0.9 Hz, 1H), 8.19 (d, *J* = 8.6 Hz, 1H), 8.14 (dd, *J* = 8.5, 1.1 Hz, 1H), 8.02 − 7.97 (m, 1H), 7.83 (ddd, *J* = 8.5, 6.8, 1.4 Hz, 1H), 7.68 (ddd, *J* = 8.1, 6.9, 1.2 Hz, 1H), 4.65 (d, *J* = 8.6 Hz, 1H), 4.33 (dd, *J* = 17.6, 1.7 Hz, 1H), 4.16 (dd, *J* = 8.7, 2.4 Hz, 1H), 3.99 (d, *J* = 8.6 Hz, 1H), 3.92 (d, *J* = 17.8 Hz, 1H), 2.66 − 2.60 (m, 1H), 2.53 (t, *J* = 7.6 Hz, 2H), 2.50 − 2.42 (m, 1H), 2.38 − 2.29 (m, 1H), 2.17 − 2.10 (m, 1H), 1.98 − 1.56 (m, 14H), 1.53 − 1.42 (m, 3H), 1.36 (s, 3H), 1.32 (s, 9H), 1.28 (s, 3H), 0.98 − 0.90 (m, 6H), 0.86 (s, 3H). ^13^C NMR (101 MHz, Methanol-*d_4_*) δ 176.4, 174.8, 174.5, 174.4, 166.4, 150.5, 148.0, 139.1, 131.6, 130.9, 129.4, 129.0, 119.6, 84.3, 81.2, 77.4, 60.8, 58.0, 53.6, 44.4, 44.1 (CHB (broad)), 41.4, 40.0, 39.2, 37.7, 36.8, 34.7, 30.2, 30.1, 29.6, 28.3, 27.8, 27.7, 26.7, 26.3, 26.0, 24.6, 15.7, 11.1. HR-MS (ESI/TOF) calcd for C_43_H_63_BN_5_O_8_ [M+H]^+^ 788.4770, found 788.4771.

#### tert-Butyl (3S,6S,12R)-6-((S)-sec-butyl)-3-cyclopentyl-1,4,7,10-tetraoxo-1-(6-phenyl-pyridin-2-yl)-12-((3aS,4S,6S,7aR)-3a,5,5-trimethylhexahydro-4,6-methanobenzo[d] [1,3,2]dioxaborol-2-yl)-2,5,8,11-tetraazapentadecan-15-oate (24b)

Prepared according to general procedure G from acid **23b** (100 mg, 0.202 mmol), **21** (100 mg, 0.208 mmol), DMAP (8 mg, 0.066 mmol), NMM (70 μl, 0.64 mmol) and T3P (240 μL, 0.40 mmol) in anhydrous CHCl_3_ (3 mL). Crude mixture was purified by flash chromatography on silica gel eluting with 0−5% MeOH in EtOAc to provide **24b** (64 mg, 39%) as an amorphous solid.

^1^H NMR (400 MHz, Methanol-*d_4_*) δ 8.16 − 8.01 (m, 5H), 7.55 − 7.41 (m, 3H), 4.66 (d, *J* = 8.1 Hz, 1H), 4.34 (dd, *J* = 17.8, 1.7 Hz, 1H), 4.15 (dd, *J* = 8.7, 2.4 Hz, 1H), 3.98 (d, *J* = 8.6 Hz, 1H), 3.91 (d, *J* = 17.6 Hz, 1H), 2.61 − 2.54 (m, 1H), 2.51 − 2.44 (m, 1H), 2.41 − 2.28 (m, 3H), 2.19 − 2.07 (m, 1H), 1.95 (t, *J* = 5.5 Hz, 1H), 1.90 − 1.74 (m, 6H), 1.74 − 1.55 (m, 6H), 1.52 − 1.42 (m, 3H), 1.36 (s, 3H), 1.33 − 1.09 (m, 13H), 0.95 (dd, *J* = 7.1, 3.9 Hz, 6H), 0.86 (s, 3H). ^13^C NMR (101 MHz, Methanol-*d_4_*) δ 176.4, 174.8, 174.4, 174.3, 166.3, 157.7, 150.5, 139.9, 139.5, 130.7, 130.0, 128.1, 124.6, 121.6, 84.3, 81.1, 77.3, 60.9, 57.6, 53.6, 44.4, 44.0 (CHB (broad)), 41.4, 40.0, 39.2, 37.7, 36.8, 34.4, 30.3, 29.9, 29.7, 28.3, 28.0, 27.8, 27.7, 26.7, 26.4, 26.1, 24.6, 15.7, 11.1. HR-MS (ESI/TOF) calcd for C_45_H_65_BN_5_O_8_ [M+H]^+^ 814.4926, found 814.4910.

#### tert-Butyl (3S,6S,12R)-6-((S)-sec-butyl)-3-cyclopentyl-1-(3,5-dimethylphenyl)-1,4,7, 10-tetraoxo-12-((3aS,4S,6S,7aR)-3a,5,5-trimethylhexahydro-4,6-methanobenzo[d] [1,3,2]dioxaborol-2-yl)-2,5,8,11-tetraazapentadecan-15-oate (24c)

Prepared according to general procedure G from acid **23c** (100 mg, 0.224 mmol), **21** (162 mg, 0.336 mmol), DMAP (9 mg, 0.074 mmol) NMM (80 μl, 0.73 mmol) and T3P (270 μL, 0.45 mmol) in anhydrous CHCl_3_ (3 mL). Crude mixture was purified by flash chromatography on silica gel eluting with 0−5% MeOH in EtOAc to provide **24c** (63 mg, 37%) as an amorphous solid.

^1^H NMR (400 MHz, Methanol-*d_4_*) δ 7.44 (s, 2H), 7.18 (s, 1H), 4.51 (d, *J* = 9.5 Hz, 1H), 4.37 (dd, *J* = 17.9, 1.8 Hz, 1H), 4.16 (dd, *J* = 8.7, 2.4 Hz, 1H), 3.93 (d, *J* = 8.6 Hz, 1H), 3.89 (d, *J* = 17.9 Hz, 1H), 2.68 − 2.57 (m, 1H), 2.53 − 2.39 (m, 2H), 2.38 − 2.27 (m, 8H), 2.20 − 2.08 (m, 1H), 2.02 − 1.90 (m, 2H), 1.90 − 1.51 (m, 12H), 1.48 (d, *J* = 10.4 Hz, 1H), 1.45 − 1.39 (m, 10H), 1.36 (s, 3H), 1.28 (s, 3H), 1.26 − 1.21 (m, 1H), 0.99 − 0.91 (m, 6H), 0.87 (s, 3H). ^13^C NMR (101 MHz, Methanol-*d_4_*) δ 176.8, 175.5, 174.9, 174.5, 170.6, 139.3, 135.3, 134.2, 126.2, 84.1, 81.8, 77.3, 61.1, 58.8, 53.6, 44.1 (CHB (broad)), 43.7, 41.4, 39.9, 39.2, 37.8, 36.7, 34.8, 30.6, 30.3, 29.6, 28.4, 28.4, 27.8, 27.8, 26.7, 26.4, 25.9, 24.6, 21.3, 15.6, 11.2. HR-MS (ESI/TOF) calcd for C_42_H_66_BN_4_O_8_ [M+H]^+^ 765.4974, found 765.5001

#### N-((S)-1-cyclopentyl-2-(((2S,3S)-1-((2-(((R)-2-hydroxy-6-oxo-1,2-oxaborinan-3-yl)amino)-2-oxoethyl)amino)-3-methyl-1-oxopentan-2-yl)amino)-2-oxoethyl) quinoline-2-carboxamide (4a)

Prepared according to general procedure F from a solution of **24a** (95 mg, 0.121 mmol) in MeCN/*n*-hexane (4.6 mL) was treated with isobutylboronic acid (49 mg, 0.48 mmol) and 1 M HCl (300 μL). Purification by flash chromatography on reversed phase silica gel provided **4a** (57 mg, 82%) as a white solid.

^1^H NMR (400 MHz, Methanol-*d_4_*) δ 8.48 (dd, *J* = 8.6, 1.0 Hz, 1H), 8.19 (d, *J* = 8.5 Hz, 1H), 8.15 (dd, *J* = 8.5, 1.0 Hz, 1H), 8.00 (dd, *J* = 8.1, 1.7 Hz, 1H), 7.84 (ddd, *J* = 8.5, 6.9, 1.5 Hz, 1H), 7.69 (ddd, *J* = 8.1, 6.9, 1.2 Hz, 1H), 4.61 (d, *J* = 8.5 Hz, 1H), 4.33 (d, *J* = 17.5 Hz, 1H), 4.19 − 4.10 (m, 2H), 2.91 (t, *J* = 4.0 Hz, 1H), 2.47 (h, *J* = 8.6 Hz, 1H), 2.34 − 2.20 (m, 2H), 1.92 − 1.58 (m, 10H), 1.54 − 1.41 (m, 2H), 1.28 − 1.17 (m, 1H), 0.95 (d, *J* = 6.8 Hz, 3H), 0.91 (t, *J* = 7.4 Hz, 3H). ^13^C NMR (101 MHz, Methanol-*d_4_*) δ 179.8, 176.7, 174.7, 174.5, 166.4, 150.4, 147.9, 139.2, 131.7, 130.9, 130.7, 129.5, 129.1, 119.5, 60.1, 58.2, 44.1, 42.7 (CHB (broad)), 39.3, 37.3, 30.3, 30.1, 29.2, 26.3, 26.0, 25.6, 15.7, 11.2. HR-MS (ESI/TOF) calcd for C_29_H_39_BN_5_O_7_ [M+H]^+^ 580.2943, found 580.2963.

#### N-((S)-1-cyclopentyl-2-(((2S,3S)-1-((2-(((R)-2-hydroxy-6-oxo-1,2-oxaborinan-3-yl)amino)-2-oxoethyl)amino)-3-methyl-1-oxopentan-2-yl)amino)-2-oxoethyl)-6-phenylpicolinamide (4b)

Prepared according to general procedure F from a solution of **24b** (37 mg, 0.046 mmol) in MeCN/*n*-hexane (1.8 mL), isobutylboronic acid (19 mg, 0.19 mmol) and 1 M HCl (115 μL). Purification by flash chromatography on reversed phase silica gel provided **4b** (26 mg, 93%) as a white solid.

^1^H NMR (400 MHz, Methanol-*d_4_*) δ 8.15 − 8.02 (m, 5H), 7.55 − 7.45 (m, 3H), 4.64 (d, *J* = 7.7 Hz, 1H), 4.38 − 4.28 (m, 1H), 4.18 − 4.06 (m, 2H), 2.89 (s, 1H), 2.50 − 2.41 (m, 1H), 2.27 − 2.19 (m, 2H), 1.92 − 1.75 (m, 5H), 1.73 − 1.56 (m, 5H), 1.53 − 1.40 (m, 2H), 1.29 − 1.17 (m, 1H), 0.96 (d, *J* = 6.9 Hz, 3H), 0.91 (t, *J* = 7.5 Hz, 3H). ^13^C NMR (101 MHz, Methanol-*d_4_*) δ 179.8, 176.6, 174.8, 174.5, 166.3, 157.7, 150.2, 140.0, 139.5, 130.7, 130.0, 128.0, 124.7, 121.6, 60.1, 57.7, 44.3, 42.7 (CHB (broad)), 39.3, 37.3, 30.3, 29.7, 29.1, 26.39, 26.37, 26.1, 25.5, 15.7, 11.2. HR-MS (ESI/TOF) calcd for C_31_H_41_BN_5_O_7_ [M+H]^+^ 606.3099, found 606.3125.

#### N-((S)-1-cyclopentyl-2-(((2S,3S)-1-((2-(((R)-2-hydroxy-6-oxo-1,2-oxaborinan-3-yl)amino)-2-oxoethyl)amino)-3-methyl-1-oxopentan-2-yl)amino)-2-oxoethyl)-3,5-dimethylbenzamide (4c)

Prepared according to general procedure F from a solution of **24c** (62 mg, 0.081 mmol) in MeCN/*n*-hexane (4 mL), isobutylboronic acid (33 mg, 0.33 mmol) and 1 M HCl (210 μL). Purification by flash chromatography on reversed phase silica gel provided **4c** (34 mg, 75%) as a white solid.

^1^H NMR (300 MHz, Methanol-*d_4_*) δ 7.43 (s, 2H), 7.19 (s, 1H), 4.39 (d, *J* = 10.1 Hz, 1H), 4.29 (d, *J* = 17.5 Hz, 1H), 4.17 − 4.08 (m, 2H), 2.88 (d, *J* = 4.0 Hz, 1H), 2.45 − 2.30 (m, 7H), 2.30 − 2.20 (m, 2H), 1.94 − 1.80 (m, 4H), 1.78 − 1.51 (m, 6H), 1.48 − 1.31 (m, 2H), 1.28 − 1.14 (m, 1H), 0.98 − 0.86 (m, 6H). ^13^C NMR (101 MHz, Methanol-*d_4_*) δ 179.8, 176.7, 175.1, 174.6, 170.9, 139.5, 135.3, 134.3, 126.2, 59.9, 59.7, 42.8 (overlaps with CHB (broad)), 39.3, 37.5, 30.8, 30.5, 29.2, 26.4, 26.2, 26.0, 25.6, 21.3, 15.8, 11.3. HR-MS (ESI/TOF) calcd for C_28_H_42_BN_4_O_7_ [M+H]^+^ 557.3147, found 557.3158.

### PfSUB1 enzyme assays

The proteolytic activity of rPfSUB1 was monitored by the cleavage of the fluorogenic peptidic substrate SERA4st1F-6R12 (Ac-CKITAQDDEESC-OH) and has been described previously.^28^ Briefly, chymotrypsin treated rPfSUB1 (expressed and purified as described before (ref) was stored at −80 °C as a 228 U/mL stock in 20 mM Tris−HCl pH 8.2, 150 mM NaCl, 10% glycerol, and diluted for use (1:500) in reaction buffer (20 mM Tris−HCl pH 8.2, 150 mM NaCl, 12 mM CaCl2, 25 mM CHAPS). Peptidic boronic acid inhibitors were dissolved in 100% DMSO at 20 mM, then further diluted in DMSO to generate stock solutions ranging from 500 to 0.01 μM and then used diluted 1:100 in the enzyme reactions. All reactions were performed in wells of white 96-well microwell plates (Nunc); 50 μL diluted rPfSUB1 was preincubated for 5 min with 1 μL each of the serially diluted boronic acid inhibitors, followed by addition of 50 μL substrate solution (0.1 μM final). Subsequent fluorescence increase was continuously monitored using a SpectraMax M5e plate reader and SoftMax Pro-6.3 software, with readings taken every 3 min for 60 min using excitation and emission values of 552 and 580 nm, respectively. Initial rates were calculated over the first 24 min of the assay, during which period progress curves were linear, and IC50 values were calculated with GraphPad Prism 9.5.0 using the nonlinear regression, [inhibitor] versus response, variable slope (four parameters). All experiments were performed in duplicate.

### Human 20S proteasome kinetic assays (chymotrypsin-like β5 activity, dose response IC_50_s)

The fluorescent Proteasome substrate LLVY-R110 (ex: 490 nm, Em:525 nm) (#MAK172, Sigma-Aldrich) was used to measure the chymotrypsin-like activity (β5) of the human 20S proteasome and was prepared according to the activity assay kit instructions. Cleavage of this peptide generated intense green fluorescence due to the R110 group and was monitored at 520-530 nm with excitation at 480-500 nm. The purified human 20S proteasome (BML-PW8720-0050, EnzoSciences), stored at 1mg/ml in 20 mM Tris-HCl pH 7.2, 1mM EDTA, 1mM DTT 1mM NaN_3_, 50% glycerol was diluted in TRIS-buffered saline (TBS, pH 7.4) to an intermediate concentration of 0.44 μM. The control inhibitor bortezomib was purchased from Sigma (# 5043140001) and used like other PfSUB1 boronic inhibitors tested in the initial dose response, at a final concentration of 500 nM in the reaction. The reactions were performed in white 96-well fluorescent microtiter-plate format (Nunc). The Substrate solution was added first (100 μl), followed by 1 μl of the inhibitor (50 μM intermediate stock) and 1 μl of the H20S proteasome at 0.44 μM. The IC_50_s were determined for a selection of potent PfSUB1 inhibitors. After sequential dilution of the inhibitors in 100% DMSO, 1 μl of each dilution was used in a 100 μl reaction containing the fluorescent substrate. The reaction was initiated by the addition of 1 μl of the 20S proteasome intermediate stock solution. The fluorescence increase was monitored using a SpectraMax M5e plate reader and SoftMax Pro-6.3 software Measure activity on a plate reader (ex: 490 nm, Em: 525 nm) with the photomultiplier tube (PMT) set to medium with readings every 3 min for one hour. All measurements were done in duplicate. The progress curves were plotted within GraphPad Prism 9.5.0.

### Covalent docking of peptidyl boronic acid inhibitors into H20S and PfSUB1

The PfSUB1 boronic inhibitors **1a, 3b, 1c, 4c** and bortezomib were docked into the chymotrypsin-like β5 enzyme of human 20S proteasome (H20S, Protein Data Bank: 5lf4)^41^ and of the *P.f*. 20S proteasome structure (Protein Data Bank: 7lxt)^42^ using the using the Internal Coordinates Mechanics software (ICM-Pro, version3.9-2d/MacOSX, Molsoft LLC). The 7lxt and 5lf4 structures were inhibited with boronic inhibitors bortezomib and delanzomib respectively, covalently bound to their chymotrypsin-like β5 enzymatic targets. The 5lf4 structure was preferred for the docking step based on model quality (2Å compared to 3.4Å for the cryo-EM 7lxt model), the *P.f*. 20S proteasome model was subsequently superimposed to the docked 5lf4-inhibitor model to compare the amino acid composition in the active site binding pocket. Hydrogen atoms were added to the 5lf4 structure in preparation for the docking procedure and the delanzomib inhibitor used to define the boundaries of the β5 active site pocket. The inhibitor was then moved away from the active site prior to docking. Trivalent and tetravalent boronic reactions involving the cyclic group boralactone were drawn and added to the selected enzymatic reaction list. All potential energy maps were generated after selecting the threonine residue (Thr2, chain K in 5lf4) as the covalent catalytic binder and set up using the program default parameters. The energy terms were based on the all-atom vacuum force field ECEPP/3 and conformational sampling was based on the biased probability Monte Carlo (BPMC) procedure.^43^ Three independent docking runs were performed per ligand, with a length of simulation (thoroughness) varied from 3 to 6 and the selection of 3 docking poses. Ligands were ranked according to their ICM energetics (ICM score, unitless), which weighs the internal force-field energy of the ligand combined with other ligand-receptor energy parameters. In equal conditions, the inhibitors were also docked into the active site of PfSUB1 (Protein Data Bank: 4lvn^44^). Hydrogen atoms were added to the structure and the C-terminal four amino acids of the pro-domain chain used to define the active site receptor boundaries for the docking procedure. The pro-domain ligand was then moved away from the receptor along with all water molecules. The receptor catalytic His428 (Nε2) atom was protonated and the active Ser606 (Oγ) selected for the covalent inhibition procedure as described before.

## Statistical analysis

All statistical analysis was carried out using GraphPad Prism 9.5.0

## Mammalian cell cytotoxicity assays

HepG2 cells were cultured in Dulbecco’s Modified Eagle Medium with F12 GlutaMAX (DMEM/F12 GlutaMAX) (Gibco) supplemented with 10% (v/v) heat-inactivated foetal calf serum (FCS), 1% L-glutamine and 1% penicillin-streptomycin (10,000 U/ml), and maintained in a humidified 5% CO_2_ incubator at 37°C. The culture medium was changed every 2-3 days and cells were passaged when a confluency of 75-80% was reached. At passage 2 or above, HepG2 cells were harvested using 0.25% trypsin-0.53 mM EDTA (Gibco). Cell counts and cell viability were determined by an automated cell counting machine (vi-CELL, Beckman) and seeded at a density of 1x10^4^-5x10^5^ cells per well in a transparent flat-bottom 96-well plate (Corning), and maintained in a humidified 5% CO_2_ incubator at 37°C. After 24 hours, cells were treated in triplicate with compounds **1a, 4c** and tipifarnib (positive control) diluted in culture medium for 48 hours. Concentrations ranged from 30 μM to 1 μM for **1a**, 200 μM to 10 μM for **4c** and 150 μM to 5 μM for tipifarnib. As a vehicle control, 1% DMSO was used. After 48 hs, media was removed and replaced with 100 μl media containing 10% resazurin solution (Invitrogen) and incubated for 4 h at 37°C in 5% CO_2_. After 4 h, the solution was transferred to an opaque 96-well plate (Corning) to measure reduction of resazurin to resorufin using a SpectraMax M5 microplate reader (Molecular Devices LLC) (ex. 560 nm, em. 590 nm). Cells without treatment compounds were used to determine 100% viability. Cell viability was determined by measuring fluorescence as a % of solvent control. Concentration viability response curves, CC50 values, mean CC50 value and standard error were generated using GraphPad Prism 10 (GraphPad Software).

## Generation of a tetracyclin-inducible PfSUB1 knock-down *P. falciparum* line

The DiCre-expressing *P. falciparum* line B11 (derived from a 3D7 background) has been described in full previously.^45^ To generate a mutant line selectively expressing reduced levels of PfSUB1, we exploited the anhydrotetracycline (aTc)-inducible TetR aptamer system described by Niles and colleagues^46^ and subsequently adapted by Rajaram et al.^47^ Briefly, the B11 line was first modified by the incorporation into the genomic *Pfs47* locus of a cassette for constitutive expression of a TetR-DOZI fusion protein. The 3’ flanking region of the *SUB1* gene was then modified by incorporation of 10 Tet repressor-binding aptamers, following by limiting dilution cloning to obtain a genetically homogenous line called 1AC5. Quantification of levels of PfSUB1 expression by western blot and protease activity from 1AC5 schizont extracts showed that when continuously maintained in medium containing 50 nM aTc, the 1AC5 parasites express 8-10 fold lower levels of PfSUB1 than the B11 parental line, without displaying significantly reduced replication rates *in vitro*. Complete details of the characterization of this parasite line will be provided in a separate manuscript.

## Parasite growth assays

The effects of test compounds on *in vitro* growth of the B11 and 1AC5 *P. falciparum* lines was determined by flow cytometry as described previously.^48^ Briefly, synchronous ring-stage parasites at 0.1% parasitaemia and 2% haematocrit were dispensed into flat-bottomed 96-well culture plates (200 ul per well) each containing 1 ul of appropriate serial dilutions of boronic acid or control antimalarial compounds in DMSO. Control wells contained DMSO only. Following incubation in sealed, humidified gassed chambers at 37°C for 96 h to allow the parasites to undergo two entire cycles of erythrocytic growth, cells were fixed, stained with SYBR green and analysed by flow cytometry on a BD FACSVerse™ using BD FACSuite™ software. Data were analysed using FlowJo software. EC50 values and statistical data were determined from dose-response curves generated using GraphPad Prism 10 (GraphPad Software).

## Supplementary Material

Supporting Information

Supporting Information

## Figures and Tables

**Table 1:** Effect of P_3_ sidechain substituents on PfSUB1 inhibitory potency

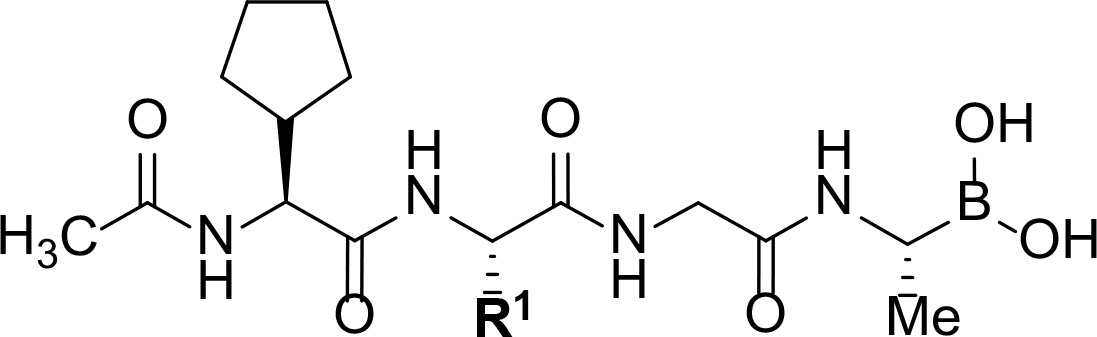			
Entry	Cmpd.	R^1^	IC_50_, nM PfSUB1 inhibition
1	**2a**	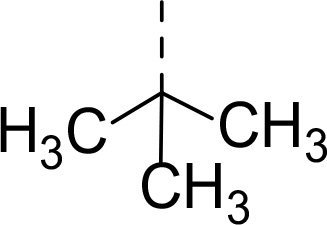	2.1±0.1
2	**2b**	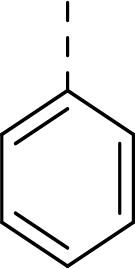	2.8±0.1
3	**2c**	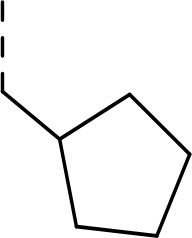	7.5±0.8
4	**2d**	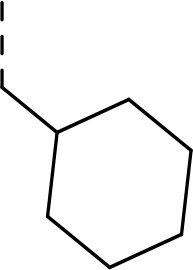	6.7±0.3
5	**2e**	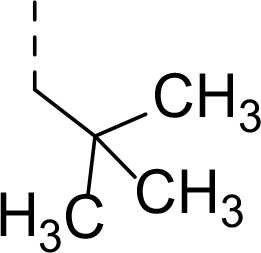	43.5±2.7
6	**2f**	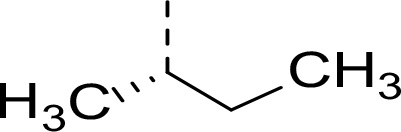	5.4±0.1
7	**2g**	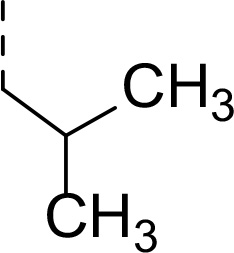	7.8±0.1
8	**2h**	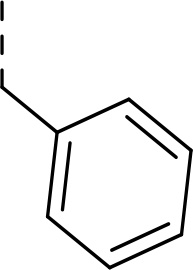	6.9±2.6

**Table 2:** Effect of end-capping groups on PfSUB1 inhibitory potency

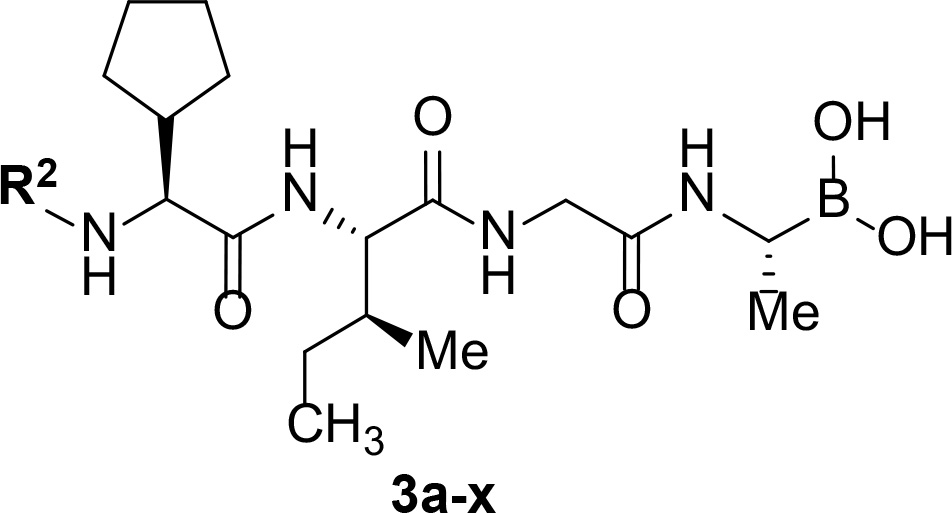			
Entry	Cmpd.	R^2^	IC_50_, nM PfSUB1 inhibition
1	**3a**	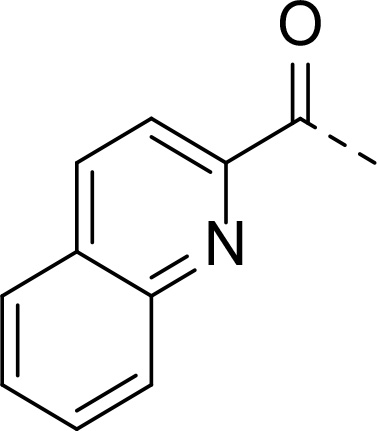	9.5±0.6
2	**3b**	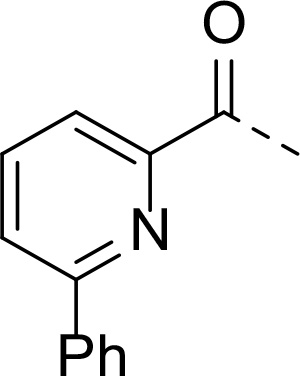	9.3±0.2
3	**3c**	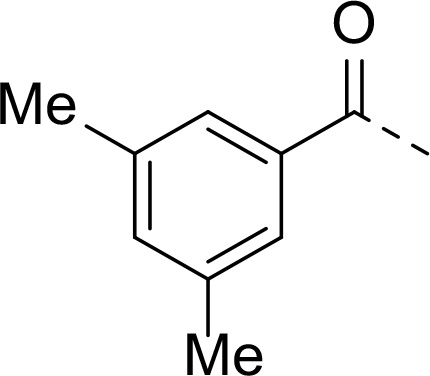	6.9 ±0.3
4	**3d**	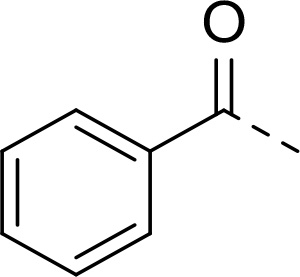	3.2±0.1
5	**3e**	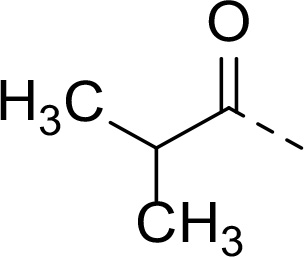	3.9±0.3
6	**3f**	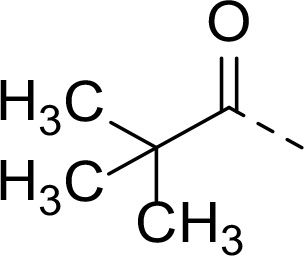	3.9±0.3
7	**3g**	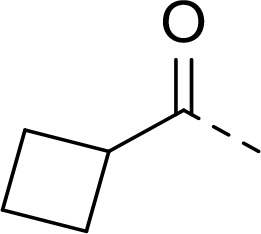	2.0±0.1
8	**3h**	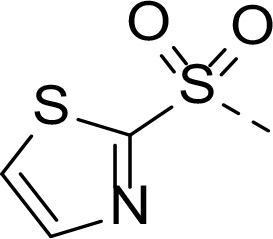	3.4±0.3
9	**3i**	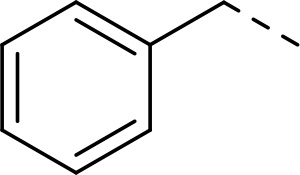	23.2 ±0.3
10	**3j**	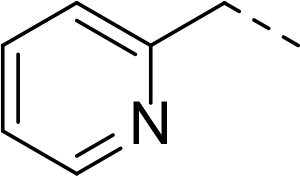	71.0 ±6.8

**Table 3:** Selectivity for PfSUB1 *vs* proteasome H20S inhibition

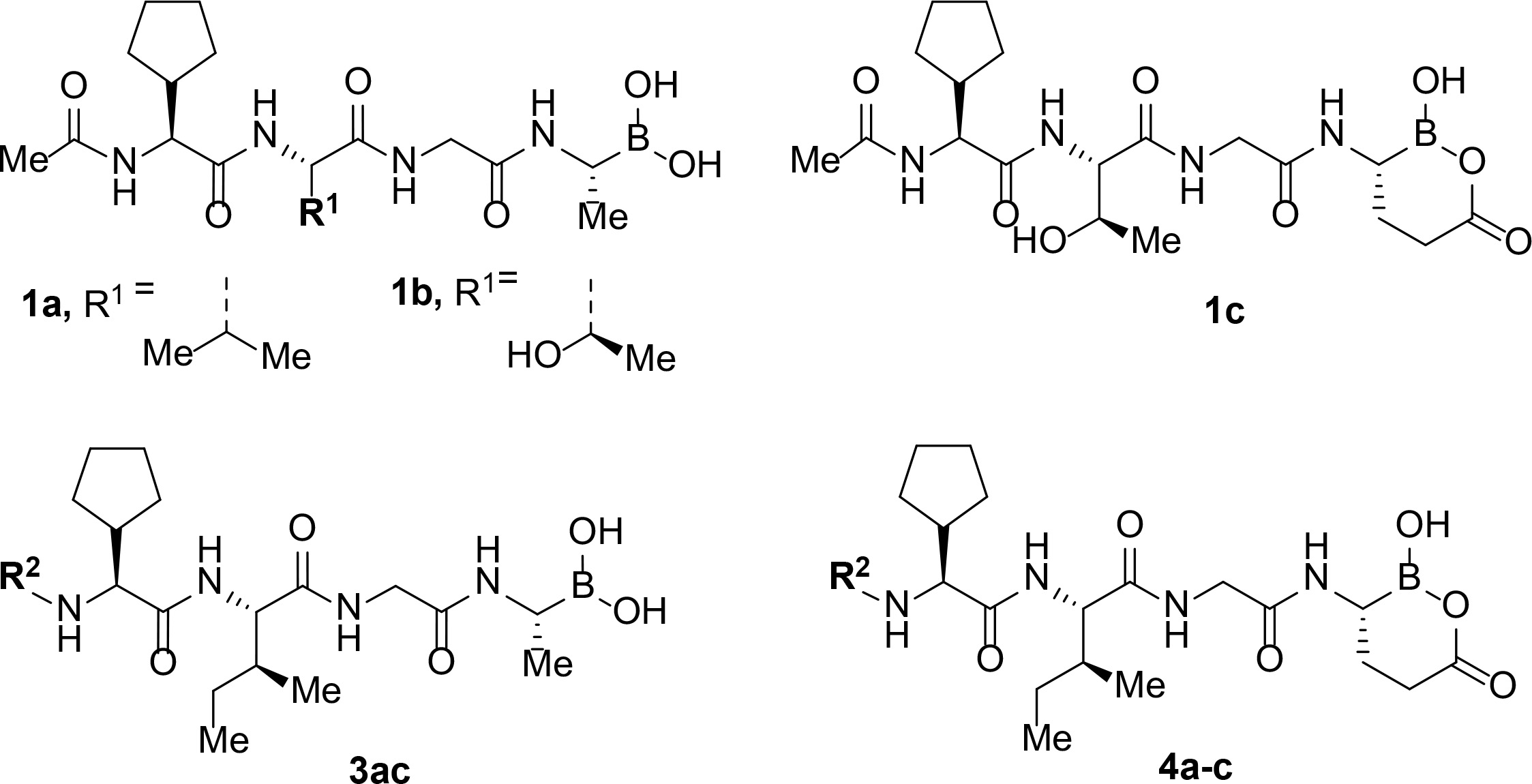					
Entry	Cmpd.	R^2^	IC_50_, nM PfSUB1 inhibition	IC_50_, nM H20S inhibition	S (H20S *vs* PfSUB1 inhibition)
1	**1a**	Ac	5.7±0.2^28^	32.8 ± 1.7	5.7
2	**1b**	Ac	9.3 ± 0.5^28^	35.4 ± 1.7	3.8
3	**1c**	Ac	18.7±1.8^28^	35 640 ± 1 800	1905
4	**3a**	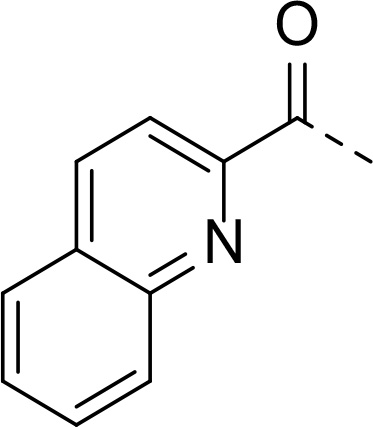	9.5±0.6	6.0 ± 0.5	0.6
5	**4a**		36.1±2.4	410 ± 5	11.3
6	**3b**	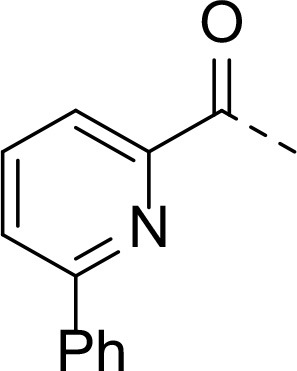	9.3±0.2	7.0 ± 0.1	0.75
7	**4b**		13.0±0.6	160 ± 10	12.3
8	**3c**	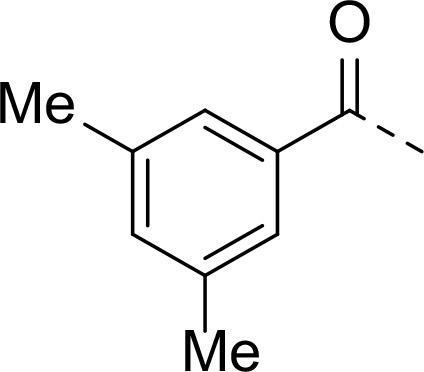	6.9 ±0.3	n.d.	n.d.
9	**4c**		15.3±0.1	1 000 ± 70	65.4
10	**5a** ^a^	**-**	Non inhibitory^b^	3-20^34^	

a Bortezomib control.

b Bortezomib used at 1 μM does not inhibit rPfSUB1 (see Supporting Information).

## Abbreviations used

### Abbreviations

ACT: artemisinin combination therapy
APCI: atmospheric pressure chemical ionization
cPentGly: cyclopentylglycine
DIPEA: *N,N*-diisopropylethylamine
EDC: 1-ethyl-3-(3-dimethylaminopropyl)carbodiimide
FCS: foetal calf serum
H20S: human proteasome
HOBt: 1-hydroxybenzotriazole
ICM: internal coordinate mechanics
*P*: *Plasmodium*
PDA: photo diode array
PfSUB1: *Plasmodium falciparum* subtilisin-like serine protease 1
Pin: pinacol
PMT: photomultiplier tube
Pnd: pinanediol
PV: parasitophorous vacuole
PVM: parasitophorous vacuole membrane
RBC: red blood cell
rPfSUB1: recombinant *Plasmodium falciparum* subtilisin-like serine protease 1
NMM: *N*-methylmorpholine
S.D.: standard deviation
SQ: single quadrupole
T3P: propanephosphonic acid anhydride
TBS: TRIS-buffered saline
TFE: 2,2,2-trifluoroethanol
