# Supplementary material for: Peptidic boronic acid Plasmodium falciparum SUB1 inhibitors with improved selectivity over human proteasome
Source: J Med Chem. Author manuscript; Available in PMC 2024 Oct 18. (PMC7616463; doi:10.1021/acs.jmedchem.4c01005)
Supplement: Supporting Information [file EMS198616-supplement-Supporting_Information.pdf]

## Supporting Information

# Peptidic boronic acid *Plasmodium falciparum* SUB1 inhibitors with improved selectivity over human proteasome

Chrislaine Withers-Martinez,[§a] Elina Lidumniece,[§b] Fiona Hackett,[a] Christine R Collins,[a] Zahie Taha,[a] Michael J. Blackman,*[a,c] and Aigars Jirgensons*[b]

[a]Malaria Biochemistry Laboratory, The Francis Crick Institute, London NW1 1AT, United Kingdom;

[b]Latvian Institute of Organic Synthesis, Riga LV-1006, Latvia;

[c]Faculty of Infectious and Tropical Diseases, London School of Hygiene & Tropical Medicine, London WC1E 7HT, United Kingdom;

[§]These authors contributed equally

## Content

1. Effect of human proteasome inhibitors on activity of recombinant PfSUB1 ............ S2

2. 20S Human Proteasome dose response assay for peptidic boronic acid SUB1 inhibitors ............ S2

3. Docking of bortezomib and inhibitors **1a**, **3b**, **1c** and **4c** into PfSUB1 ............ S3

4. Compound **2a–h**, **3a–j**, **4a–c** characterization spectra ............ S4

5. Representative examples of HPLC purity ............ S46

## 1. Effect of human proteasome inhibitors on activity of recombinant PfSUB1

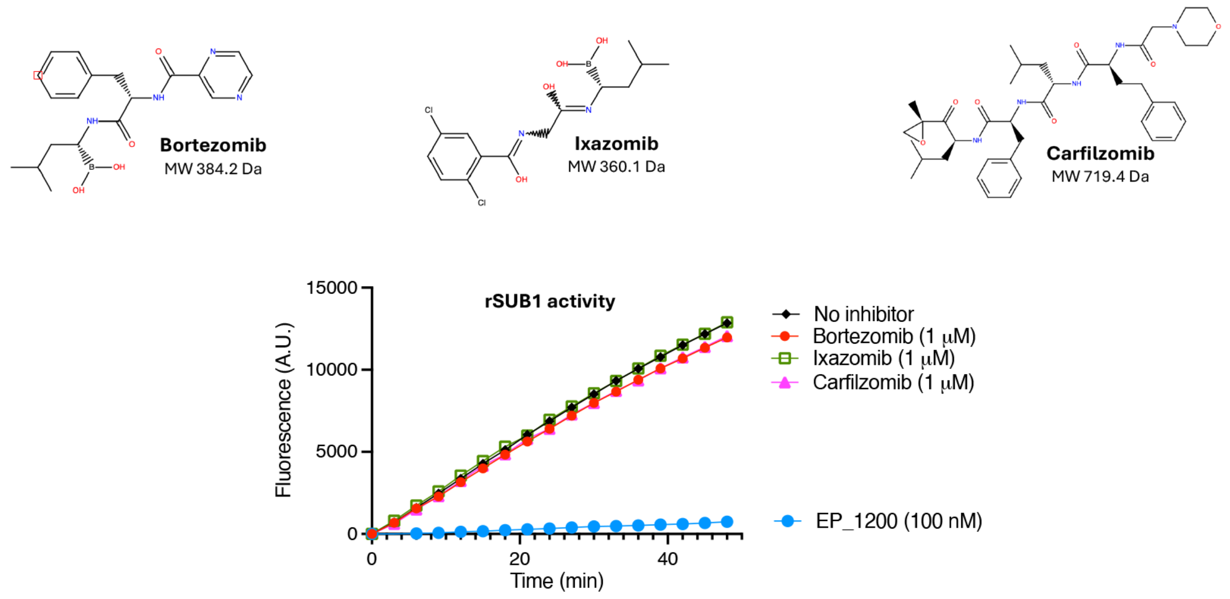


**Figure S1**. Human proteasome inhibitors do not inhibit recombinant PfSUB1. In vitro PfSUB1 activity assay showing lack of inhibition by bortezomib, ixazomib or carfilzomib (1 uM final concentration). EP_1200 (used as a positive control) completely inhibited recombinant PfSUB1 activity at 100 nM. The assay shown is typical of duplicate assays.

## 2. 20S Human Proteasome dose response assay for peptidic boronic acid SUB1 inhibitors

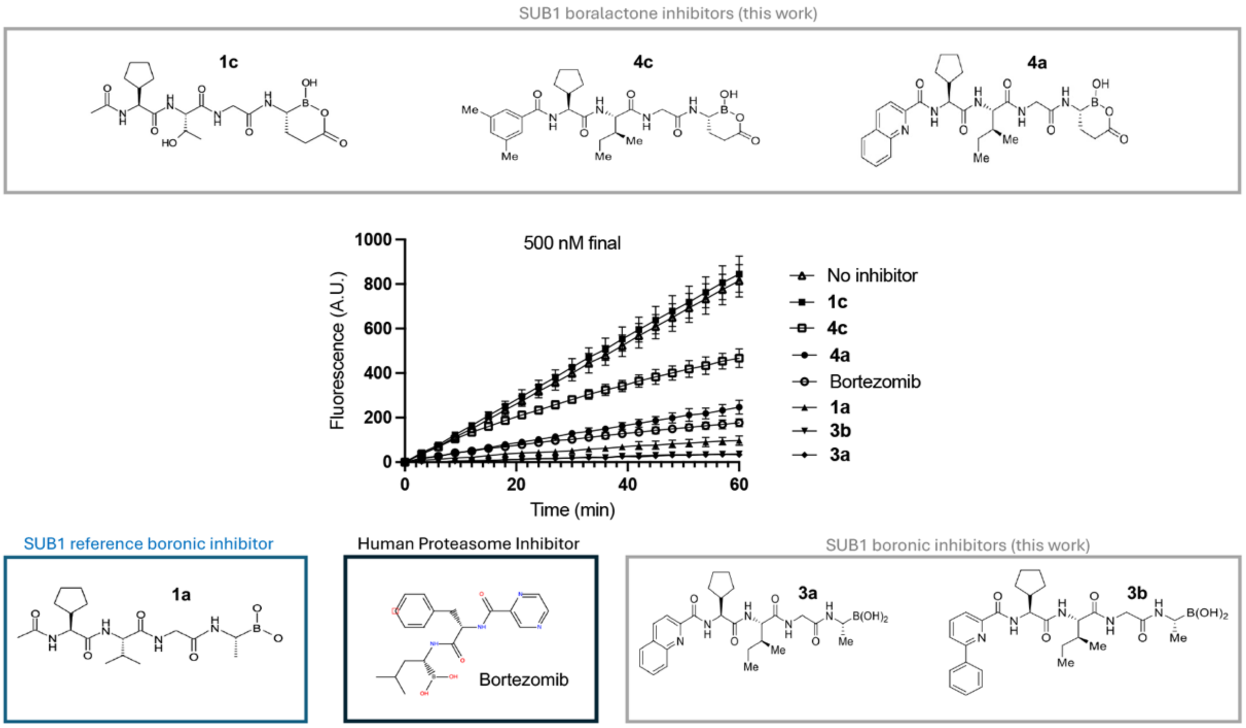


**Figure S2**. 20S Human proteasome dose response assay. The peptidic boronic acid PfSUB1 inhibitors **1a**, **3a**, **3b**, the boralactone PfSUB1 inhibitors **1c**, **4c**, **4a** and the bortezomib control were used at 500 nM final in a fluorescence-based enzymatic assay measuring the chymotrypsin-like activity (β5) of the human 20S proteasome. All measurements were performed in duplicate, with points shown as mean values. Error bars, S.D.

### 3. Docking of bortezomib and inhibitors 1a, 3b, 1c and 4c into PfSUB1

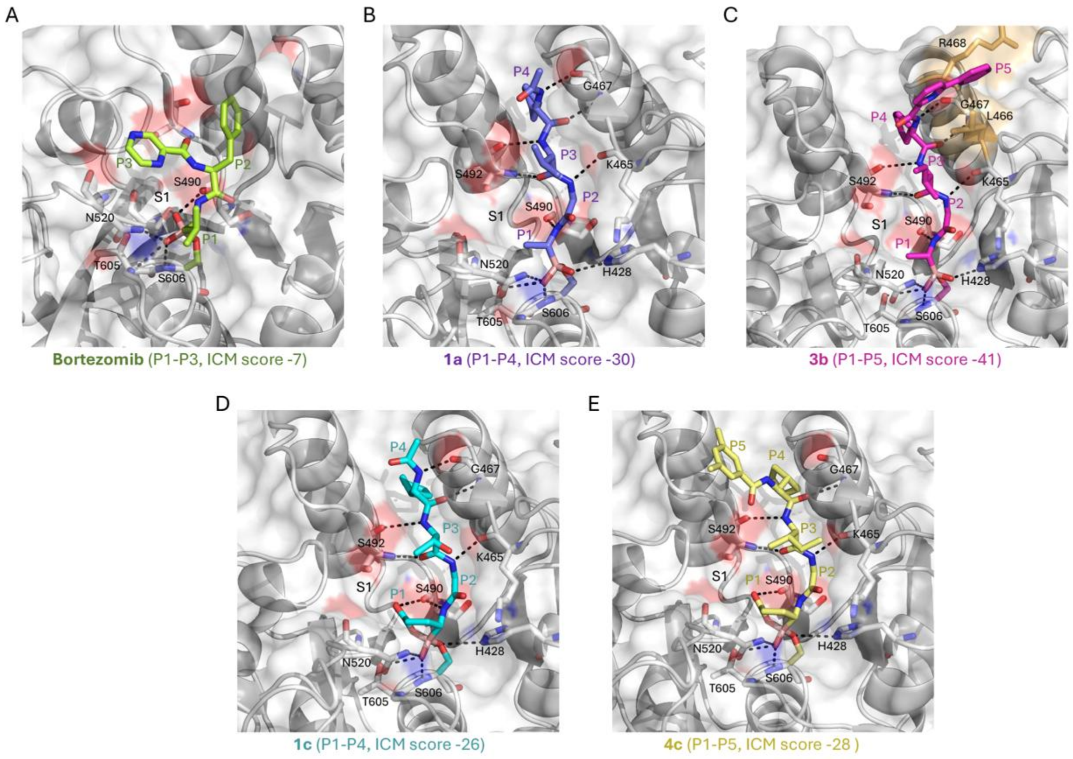


**Figure S3**. Bortezomib, compound **1a**, **3b**, **1c** and **4c** docked into PfSUB1(PDB: 4lvn). SUB1 is shown as a cartoon with a semi-transparent molecular surface colored by elements (O: red, N: blue). Positions P1 to P5 of inhibitors are indicated, the boron atom is colored in pink. Stabilizing H-bonds are shown as black dashed lines. **A**.: Bortezomib docked into the active site of PfSUB1 (ICM score -7) is shown as green sticks colored by elements. The P1 Leu side chain did not fill the S1 polar pocket and faced outwards the pocket. **B**.: compound **1a** docked into the active site PfSUB1 (ICM score -30) is shown as purple sticks colored by elements. The P1 Ala side chain fitted the S1 pocket. **C**: compound **3b** docked into the active site PfSUB1(ICM score -41) is shown as magenta sticks colored by elements. The P1 Ala side chain fitted the S1 pocket. The P5 phenyl pyridine capping group was stabilized by Pi-stacking interactions involving Leu466 and Arg468 (in wheat colour). **D.:** compound **1c** docked into the active site of PfSUB1 (ICM score -26) is shown as teal sticks colored by elements. The boralactone group fitted the S1 polar pocket. E.: compound **4c** docked into the active site of PfSUB1 (ICM score -28) is shown as yellow sticks colored by elements. The boralactone group fitted the S1 polar pocket

## 4. Compound 2a–h, 3a–j, 4a–c characterization spectra

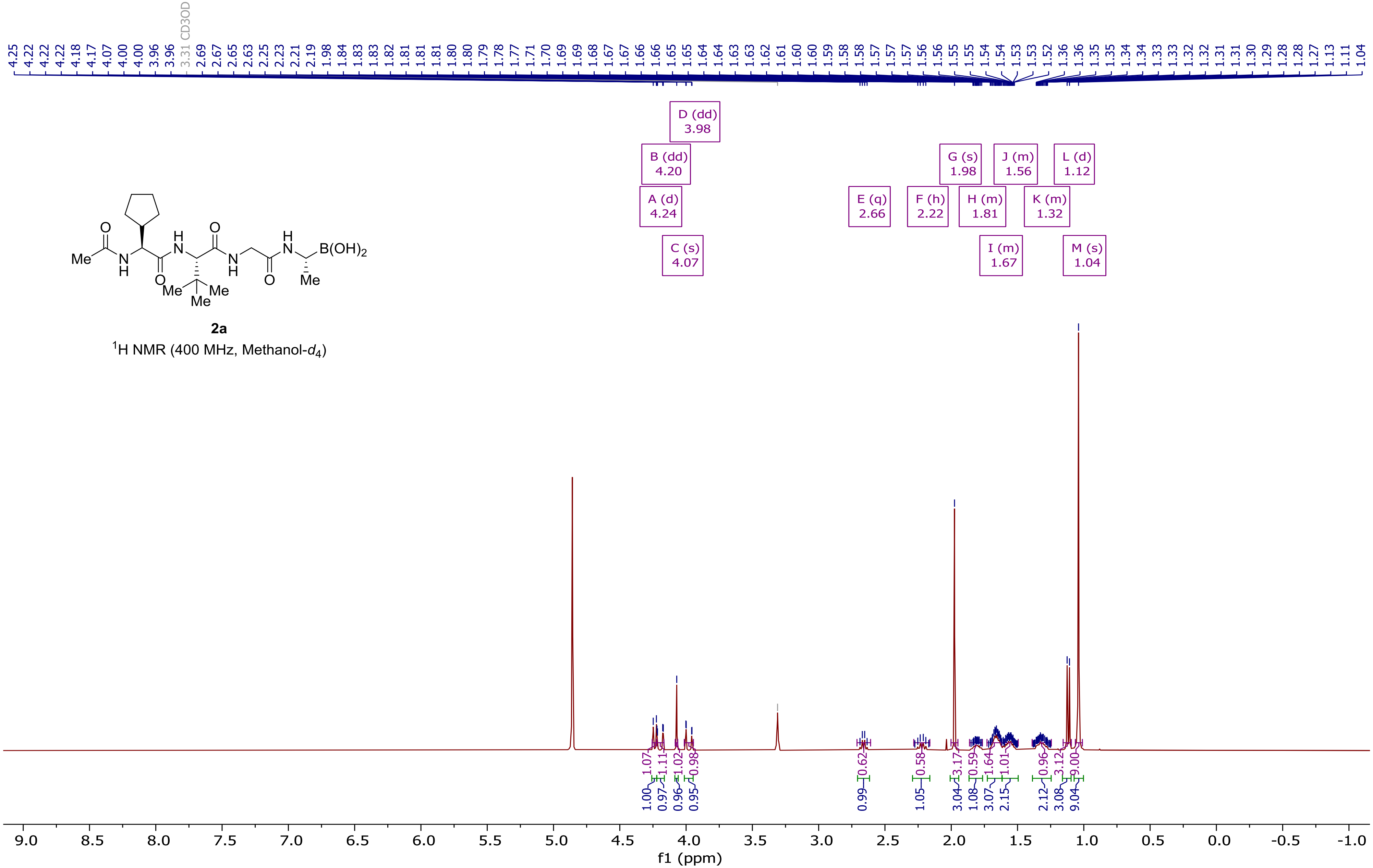


**2a**

$^1H$ NMR (400 MHz, Methanol-$d_4$)

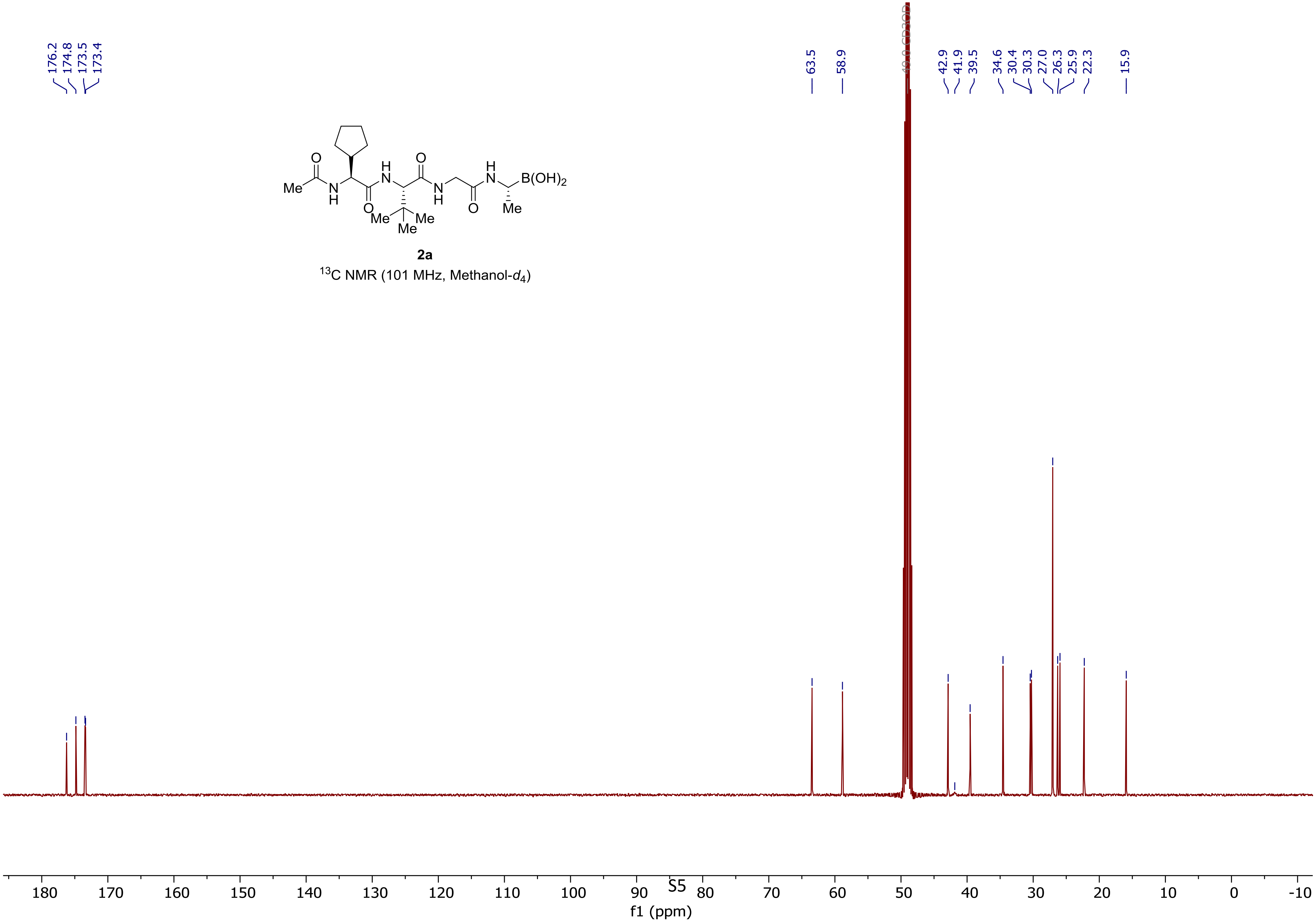
176.2
174.8
173.5
173.4
63.5
58.9
49.0 CD3OD
42.9
41.9
39.5
34.6
30.4
30.3
27.0
26.3
25.9
22.3
15.9
Me
N
H
O
O
H
N
Me
Me
Me
O
N
H
O
H
N
B(OH)2
Me
2a
$^{13}$C NMR (101 MHz, Methanol-$d_4$)
180
170
160
150
140
130
120
110
100
90
80
70
60
50
40
30
20
10
0
-10
f1 (ppm)

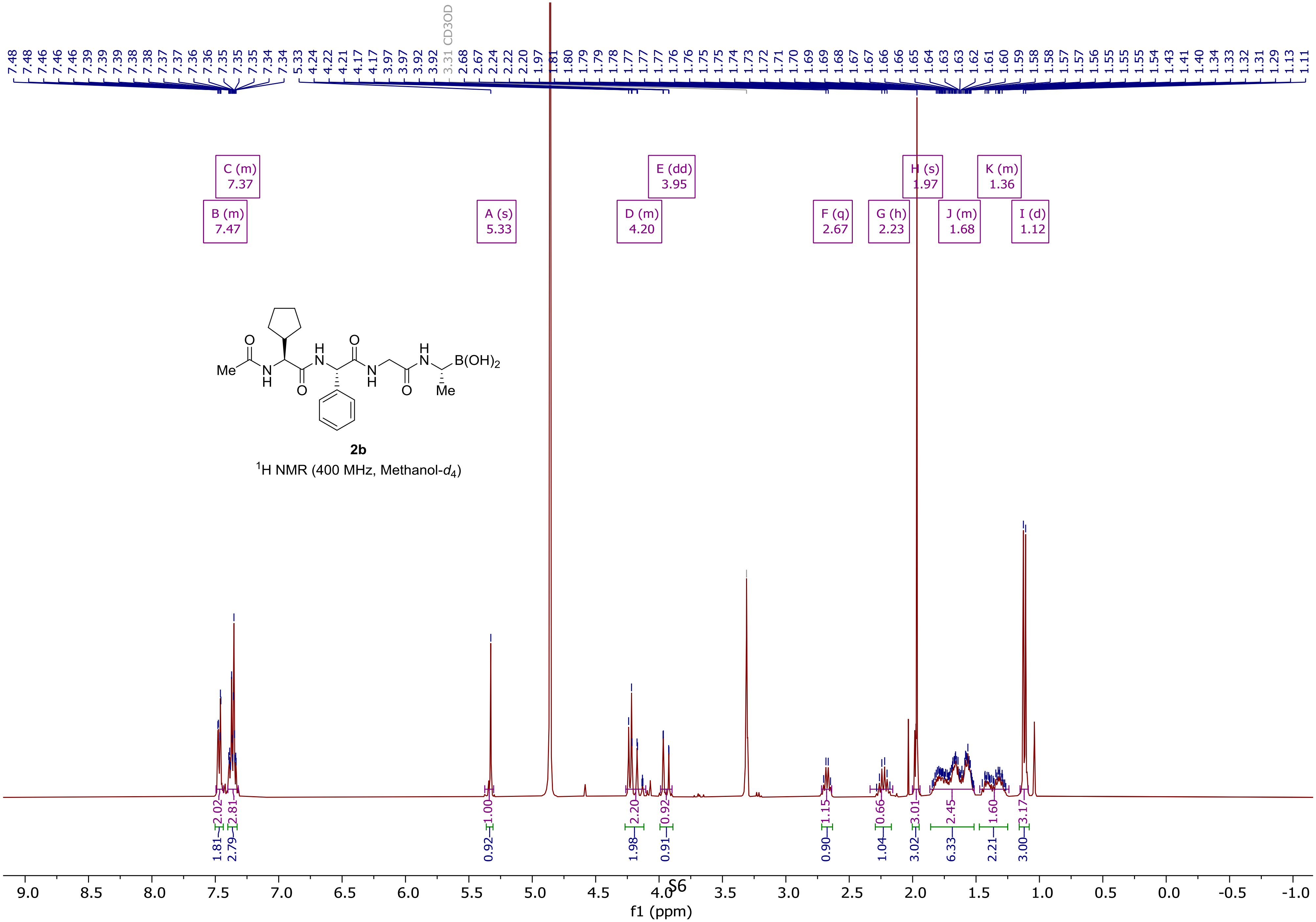


**2b**

$^1$H NMR (400 MHz, Methanol-$d_4$)

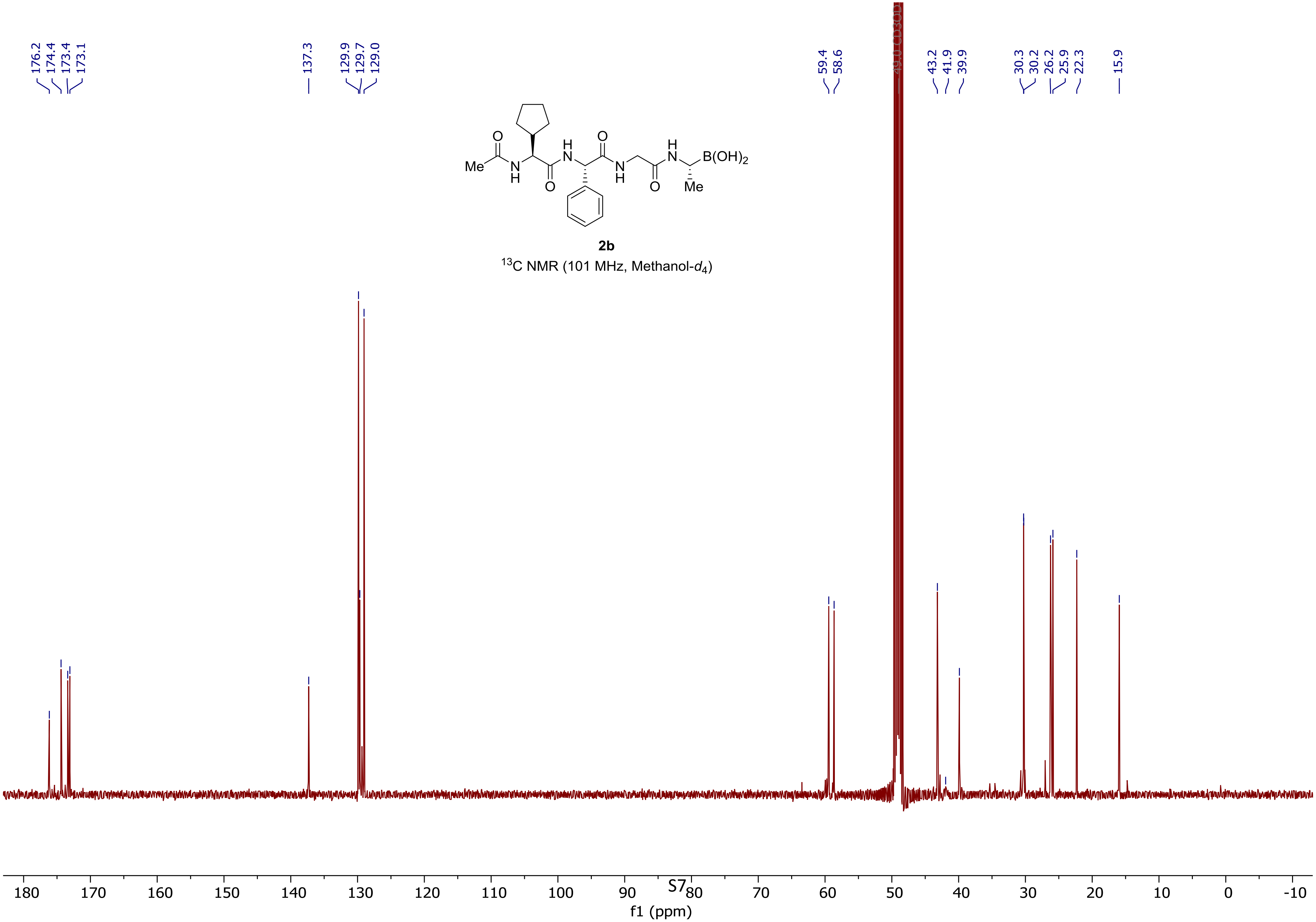


**2b**

$^{13}$C NMR (101 MHz, Methanol-$d_4$)

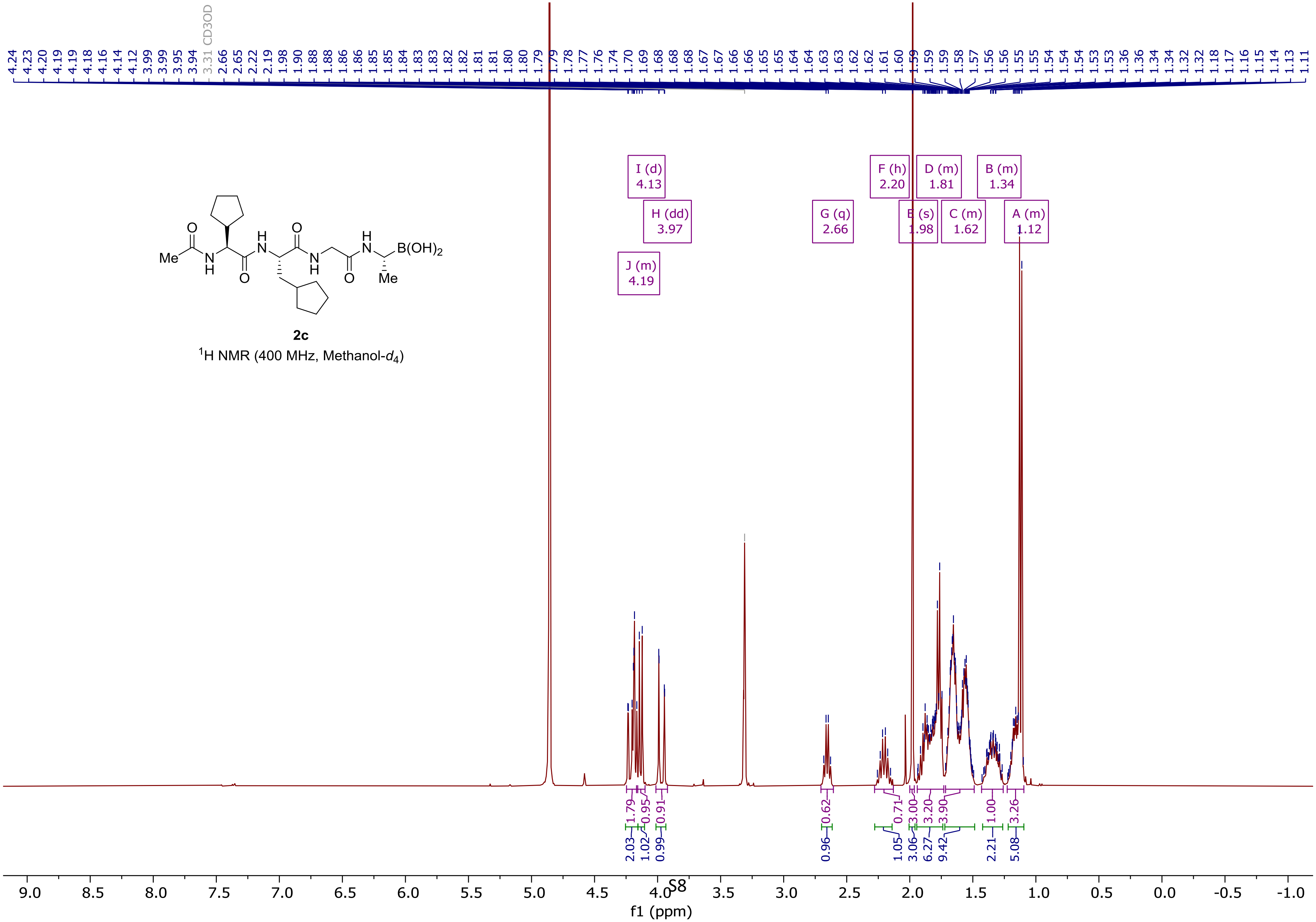
4.24 4.23 4.20 4.19 4.19 4.18 4.16 4.14 4.12 3.99 3.99 3.95 3.94 3.31 CD3OD 2.66 2.65 2.22 2.19 1.98 1.90 1.88 1.88 1.86 1.86 1.85 1.85 1.84 1.83 1.83 1.82 1.82 1.81 1.81 1.80 1.80 1.79 1.79 1.78 1.77 1.76 1.74 1.70 1.69 1.68 1.68 1.67 1.67 1.66 1.66 1.65 1.65 1.64 1.64 1.63 1.63 1.62 1.62 1.61 1.60 1.59 1.59 1.59 1.58 1.57 1.56 1.56 1.55 1.55 1.54 1.54 1.54 1.53 1.53 1.36 1.36 1.34 1.34 1.32 1.32 1.18 1.17 1.16 1.15 1.14 1.13 1.11
I (d) 4.13
H (dd) 3.97
J (m) 4.19
G (q) 2.66
F (h) 2.20
E (s) 1.98
D (m) 1.81
C (m) 1.62
B (m) 1.34
A (m) 1.12
Me
B(OH)2
2c
1H NMR (400 MHz, Methanol-d4)
2.03 1.02 0.99 0.96 1.05 3.06 6.27 9.42 2.21 5.08
1.79 0.95 0.91 0.62 0.71 3.00 3.20 3.90 1.00 3.26
9.0 8.5 8.0 7.5 7.0 6.5 6.0 5.5 5.0 4.5 4.0 3.5 3.0 2.5 2.0 1.5 1.0 0.5 0.0 -0.5 -1.0
f1 (ppm)

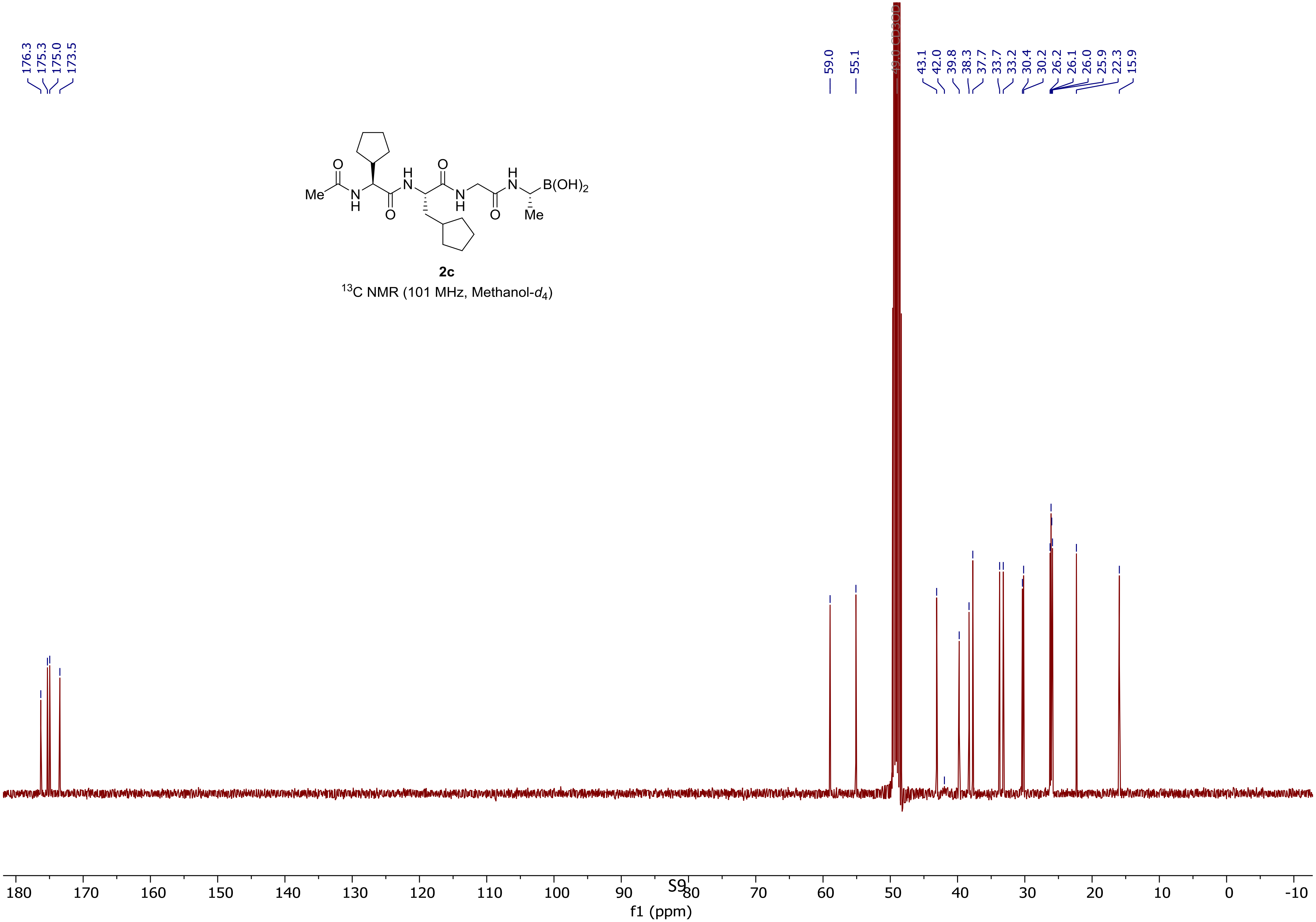
176.3
175.3
175.0
173.5
59.0
55.1
49.0 CD3OD
43.1
42.0
39.8
38.3
37.7
33.7
33.2
30.4
30.2
26.2
26.1
26.0
25.9
22.3
15.9
Me
O
N
H
H
N
B(OH)2
2c
13C NMR (101 MHz, Methanol-d4)
180 170 160 150 140 130 120 110 100 90 80 70 60 50 40 30 20 10 0 -10
f1 (ppm)

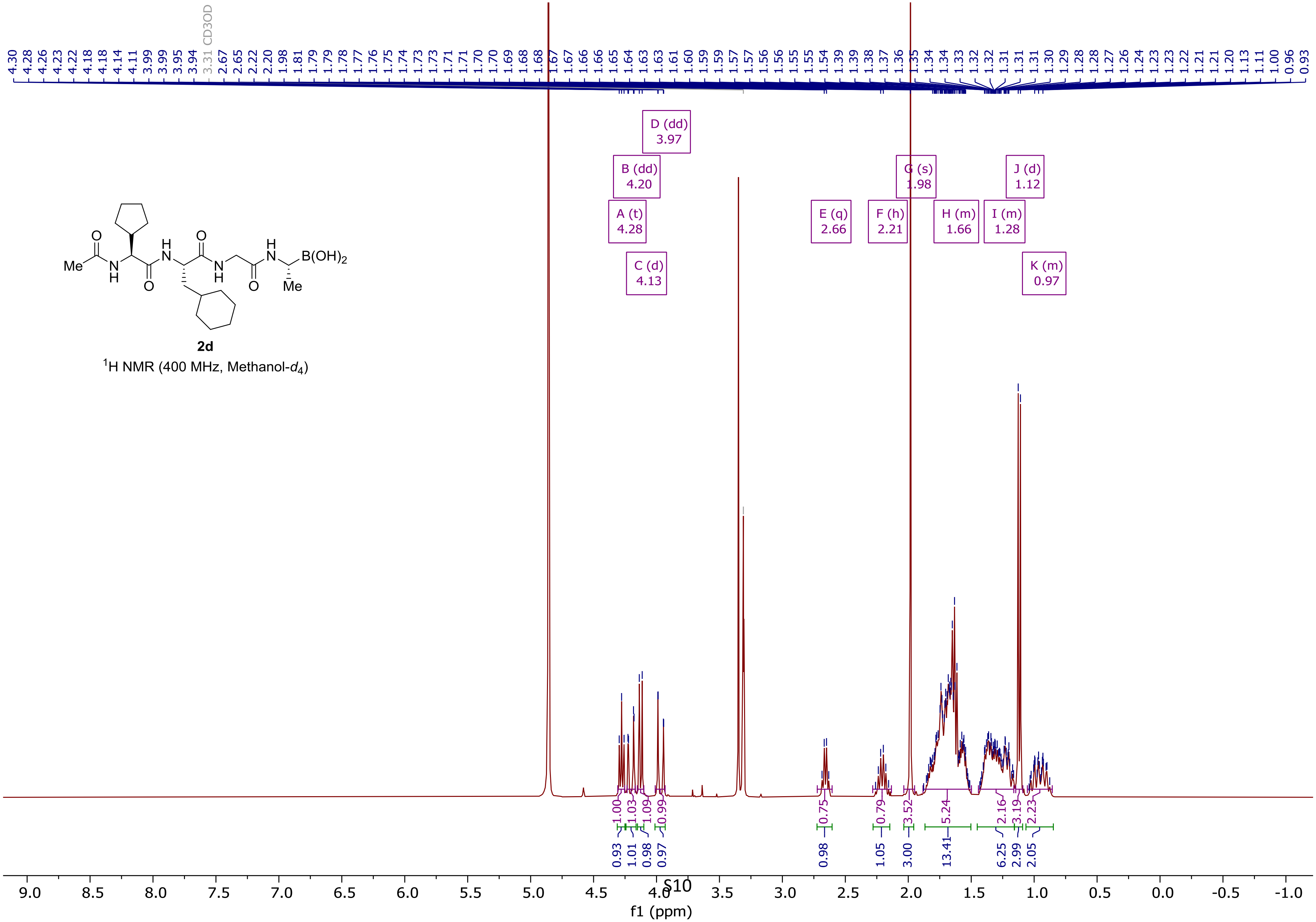

**2d**

$^{1}$H NMR (400 MHz, Methanol-$d_4$)

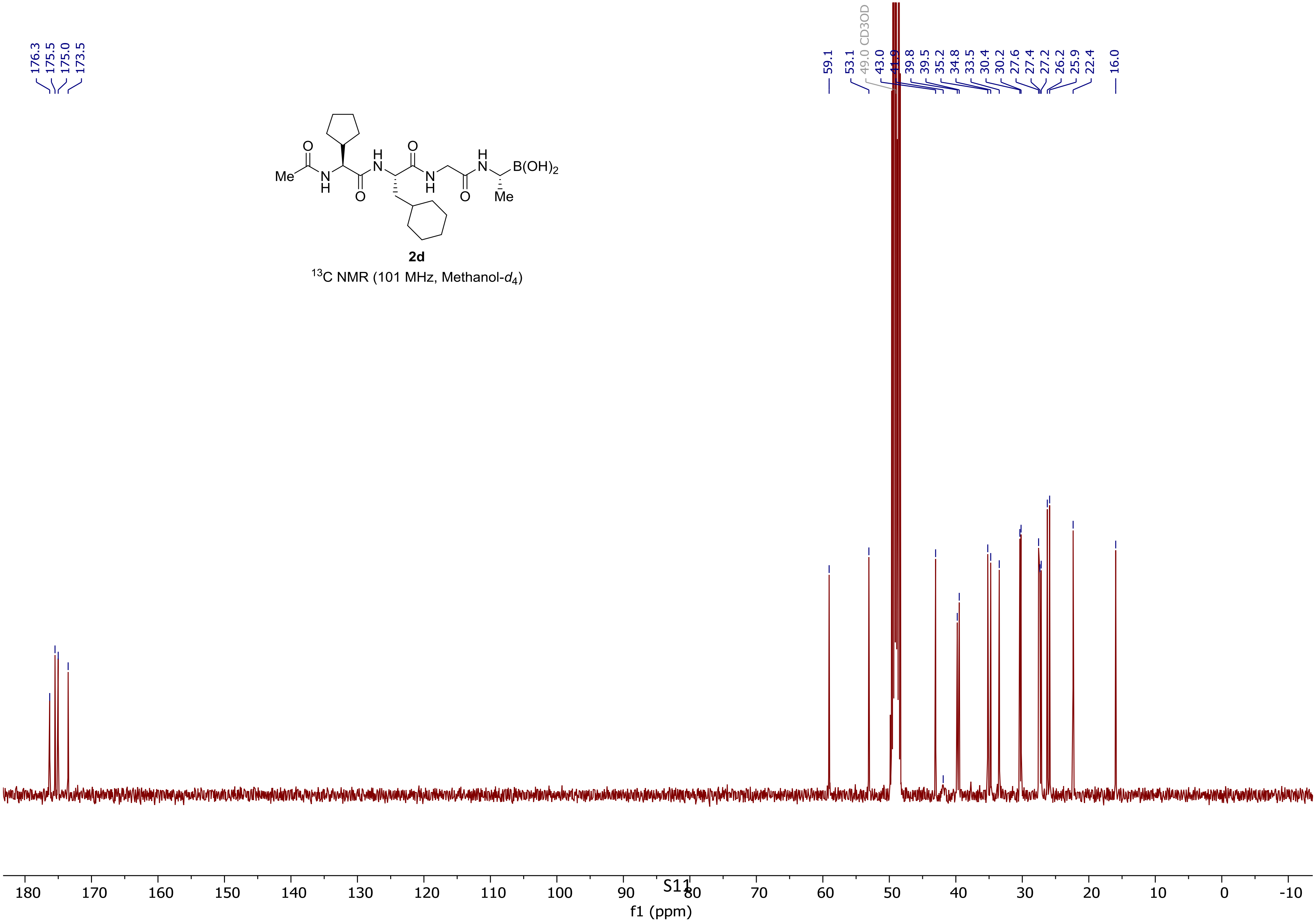
176.3
175.5
175.0
173.5
59.1
53.1
49.0 CD3OD
43.0
41.9
39.8
39.5
35.2
34.8
33.5
30.4
30.2
27.6
27.4
27.2
26.2
25.9
22.4
16.0
Me
N
H
O
H
N
O
N
H
O
H
N
B(OH)2
Me
2d
13C NMR (101 MHz, Methanol-d4)
180
170
160
150
140
130
120
110
100
90
80
70
60
50
40
30
20
10
0
-10
f1 (ppm)

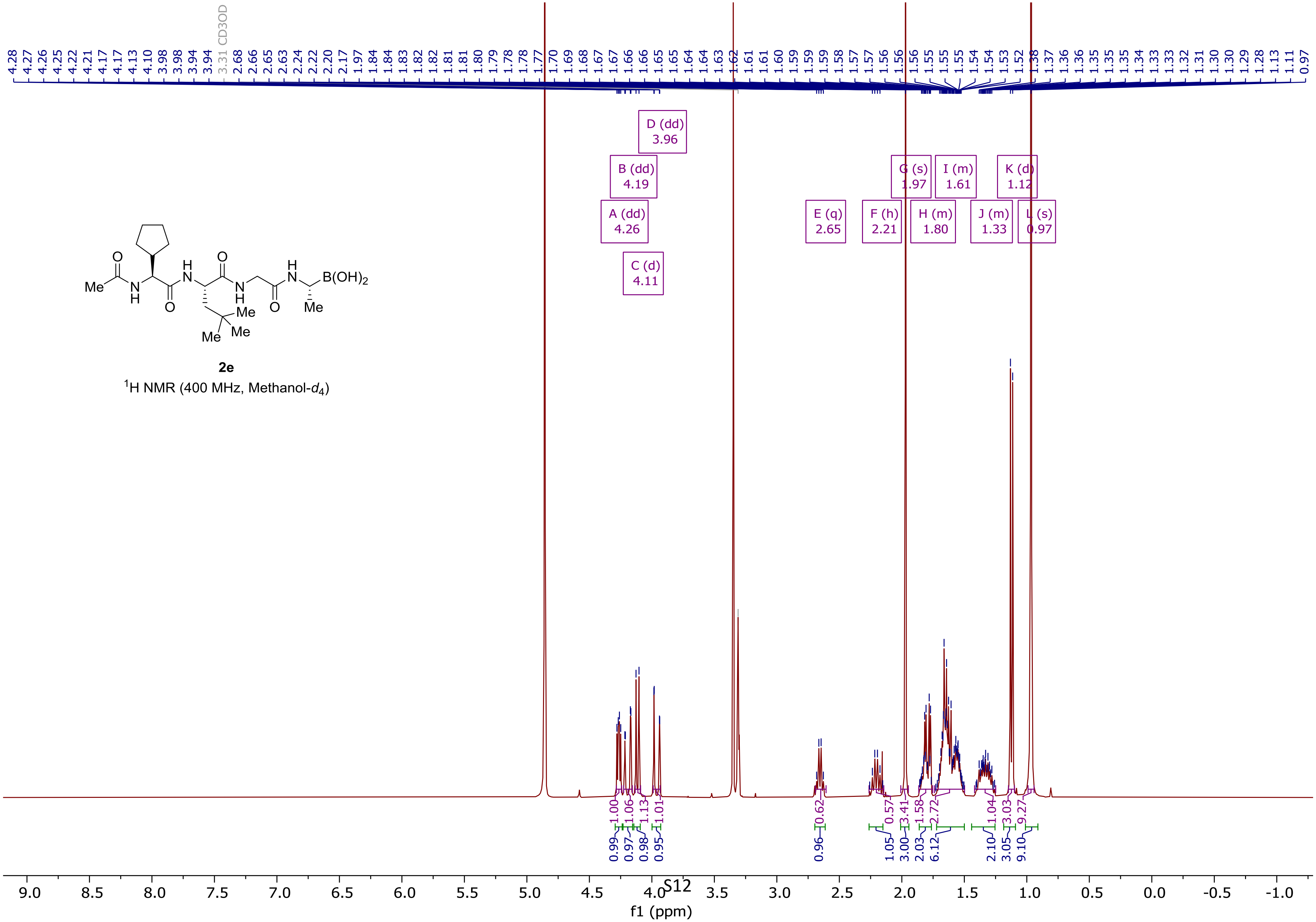

2e
$^{1}$H NMR (400 MHz, Methanol-$d_4$)
A (dd) 4.26
B (dd) 4.19
C (d) 4.11
D (dd) 3.96
E (q) 2.65
F (h) 2.21
G (s) 1.97
H (m) 1.80
I (m) 1.61
J (m) 1.33
K (d) 1.12
L (s) 0.97
CD3OD
f1 (ppm)

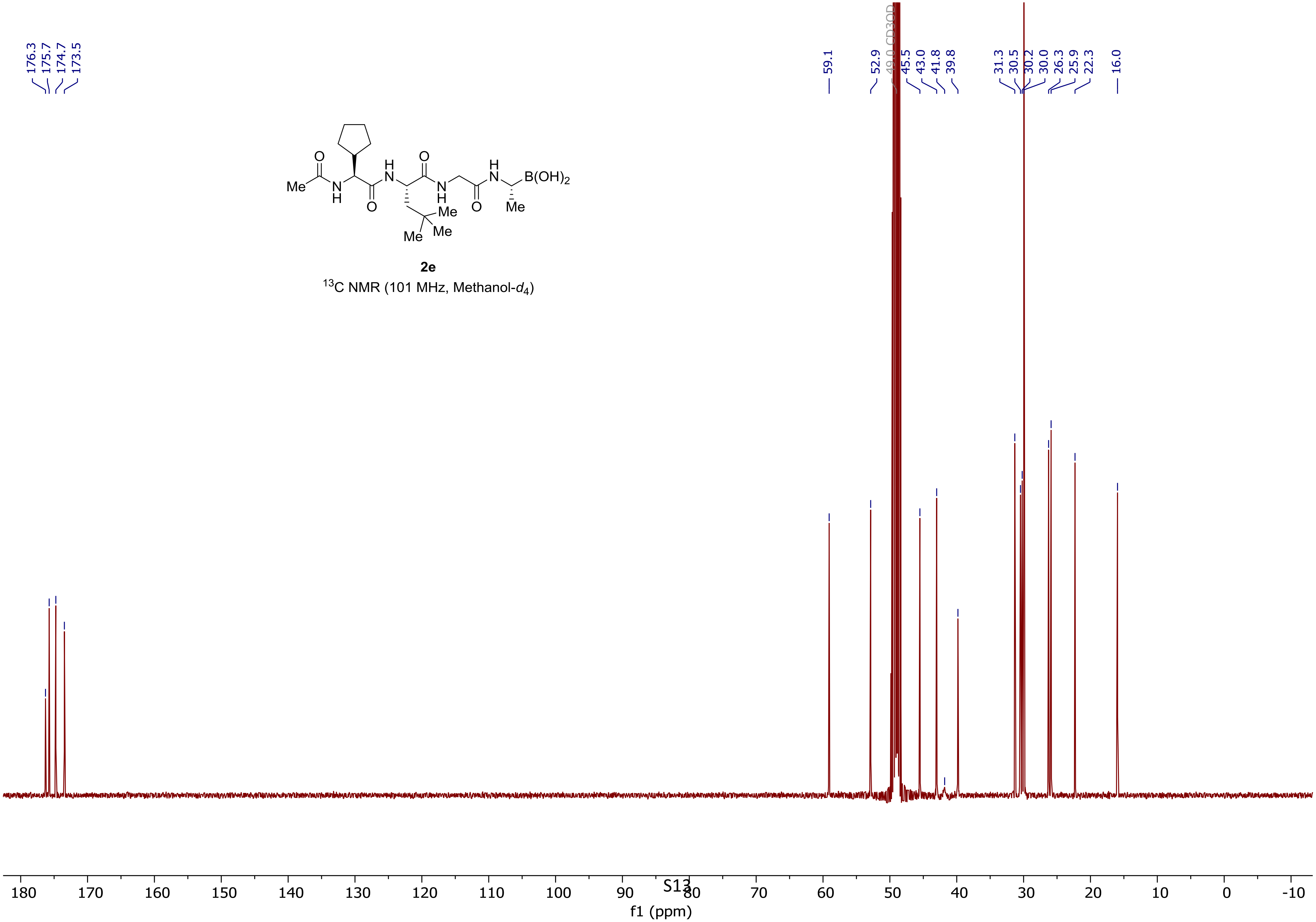


**2e**

$^{13}$C NMR (101 MHz, Methanol-$d_4$)

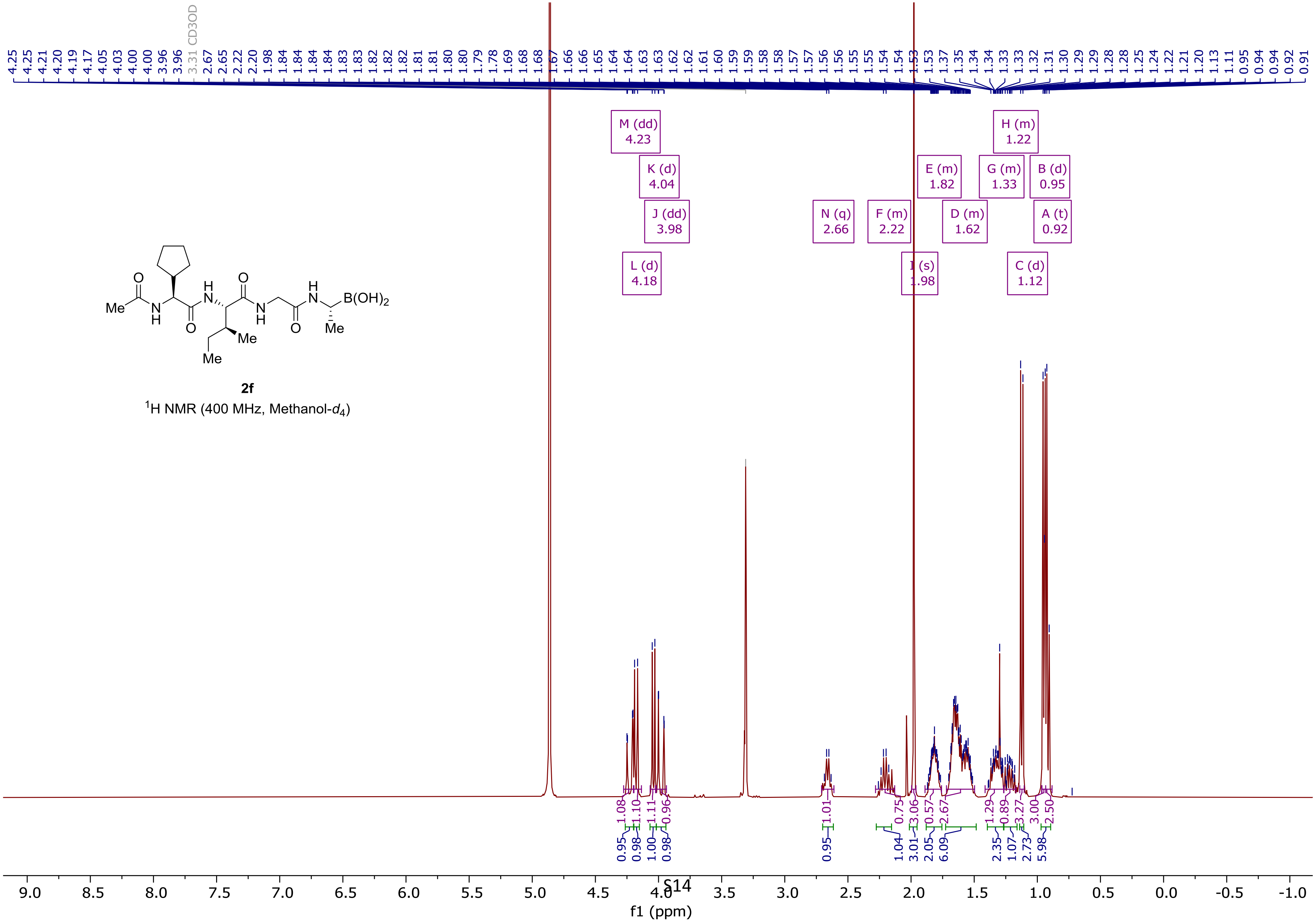


**2f**

$^1$H NMR (400 MHz, Methanol-$d_4$)

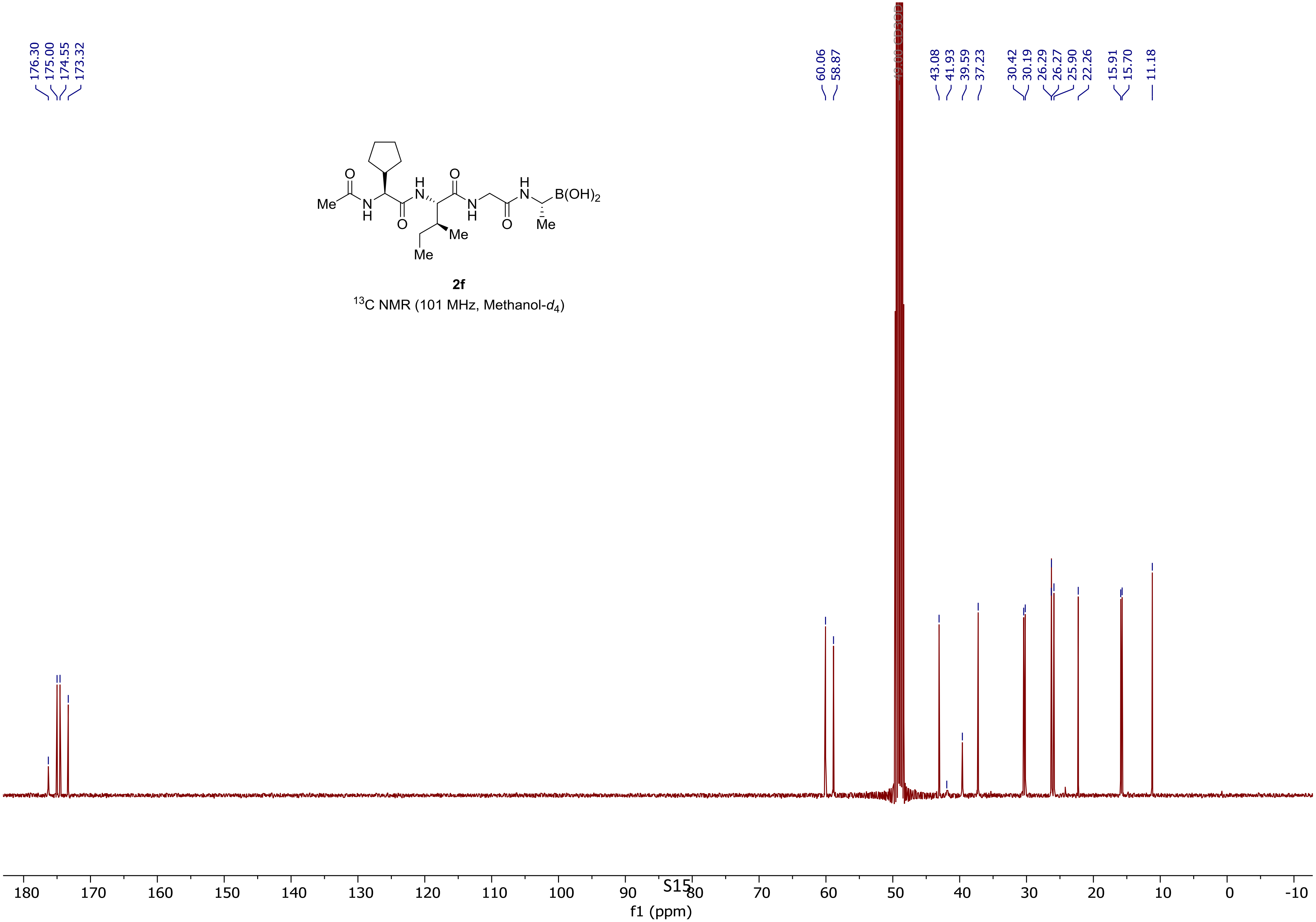


**2f**

$^{13}$C NMR (101 MHz, Methanol-$d_4$)

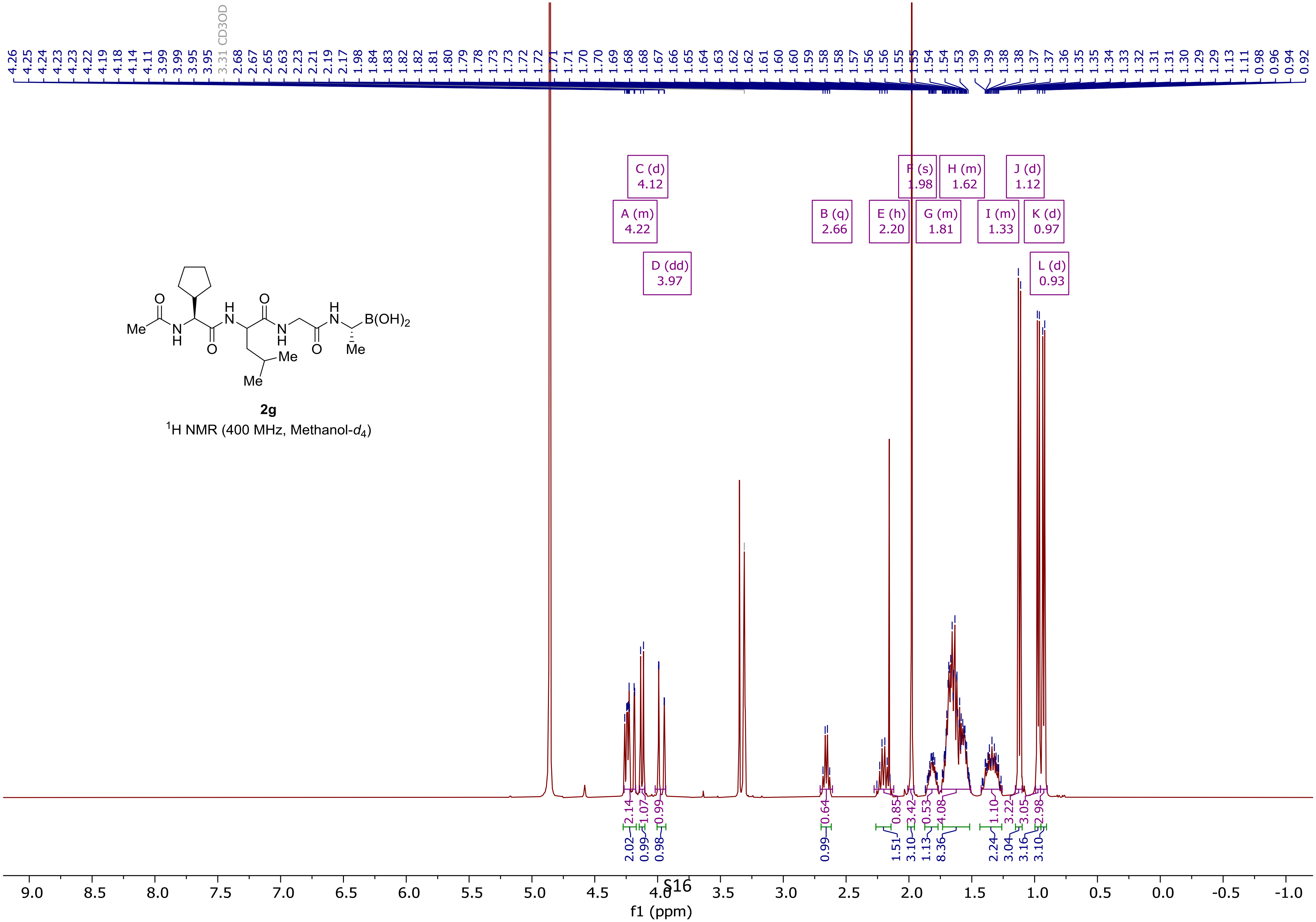


**2g**

$^1$H NMR (400 MHz, Methanol-$d_4$)

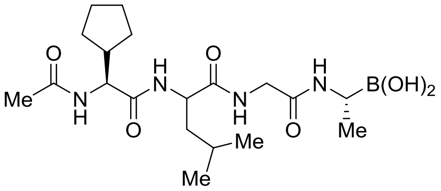


**2g**

$^{13}$C NMR (101 MHz, Methanol-$d_4$)

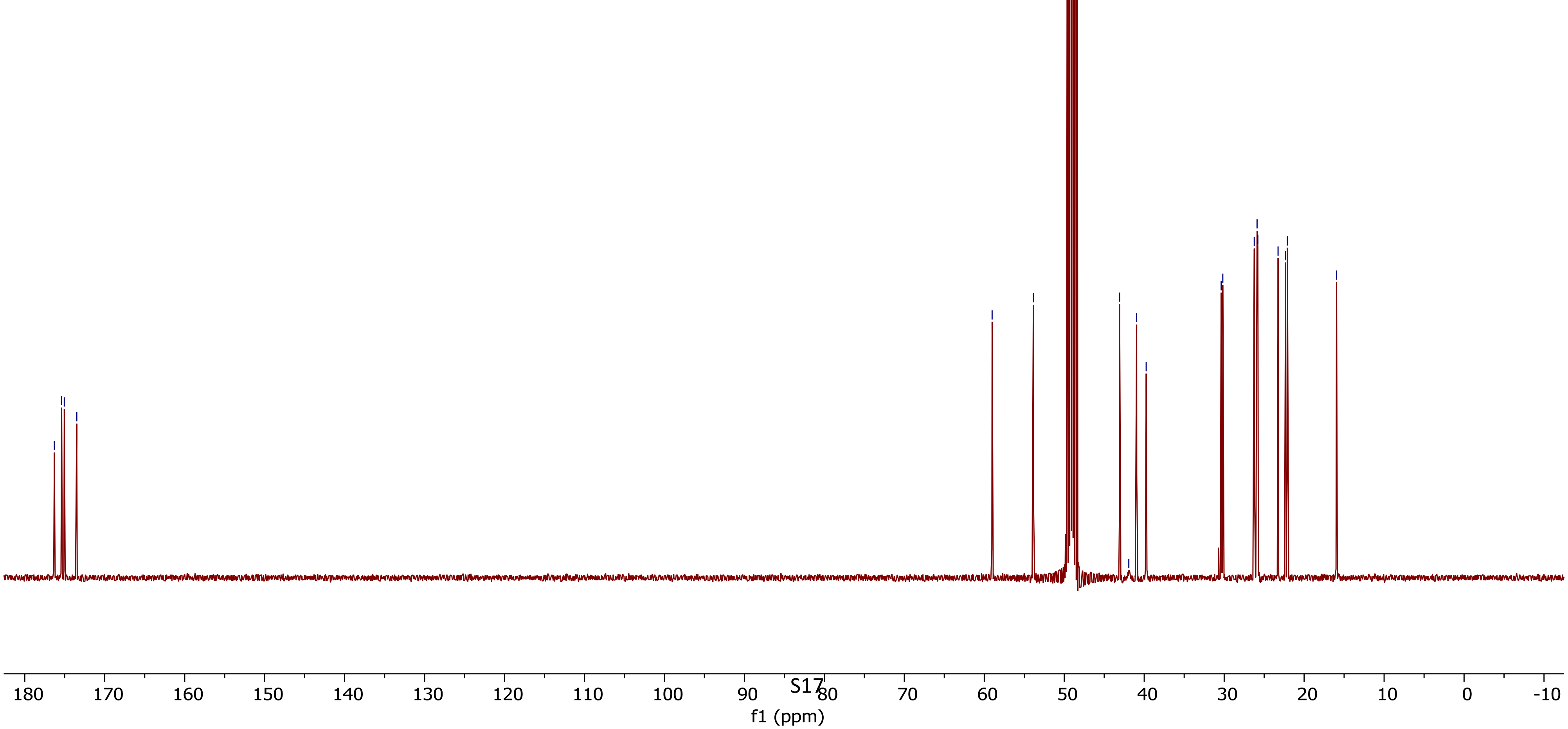

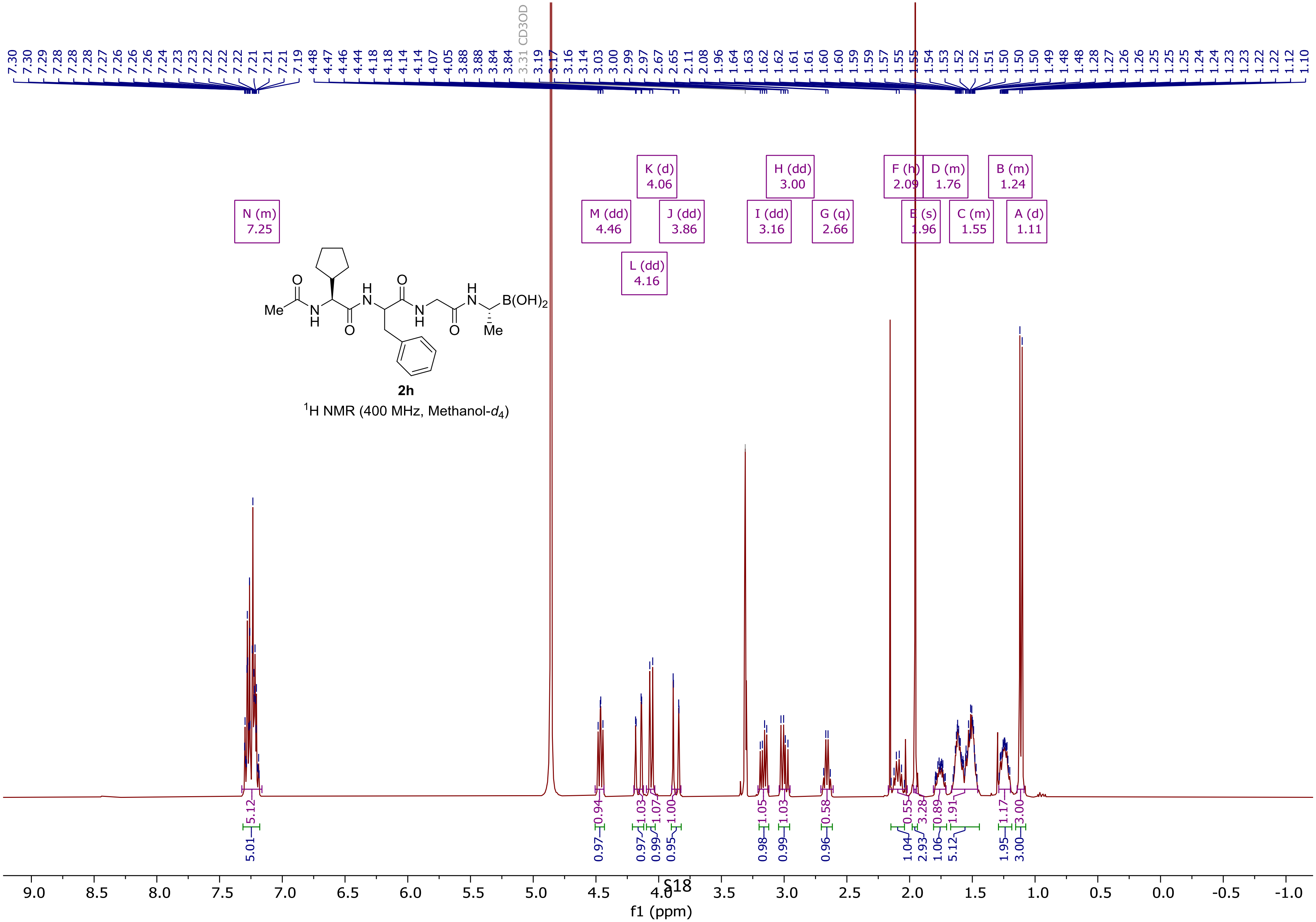


**2h**

$^1$H NMR (400 MHz, Methanol-$d_4$)

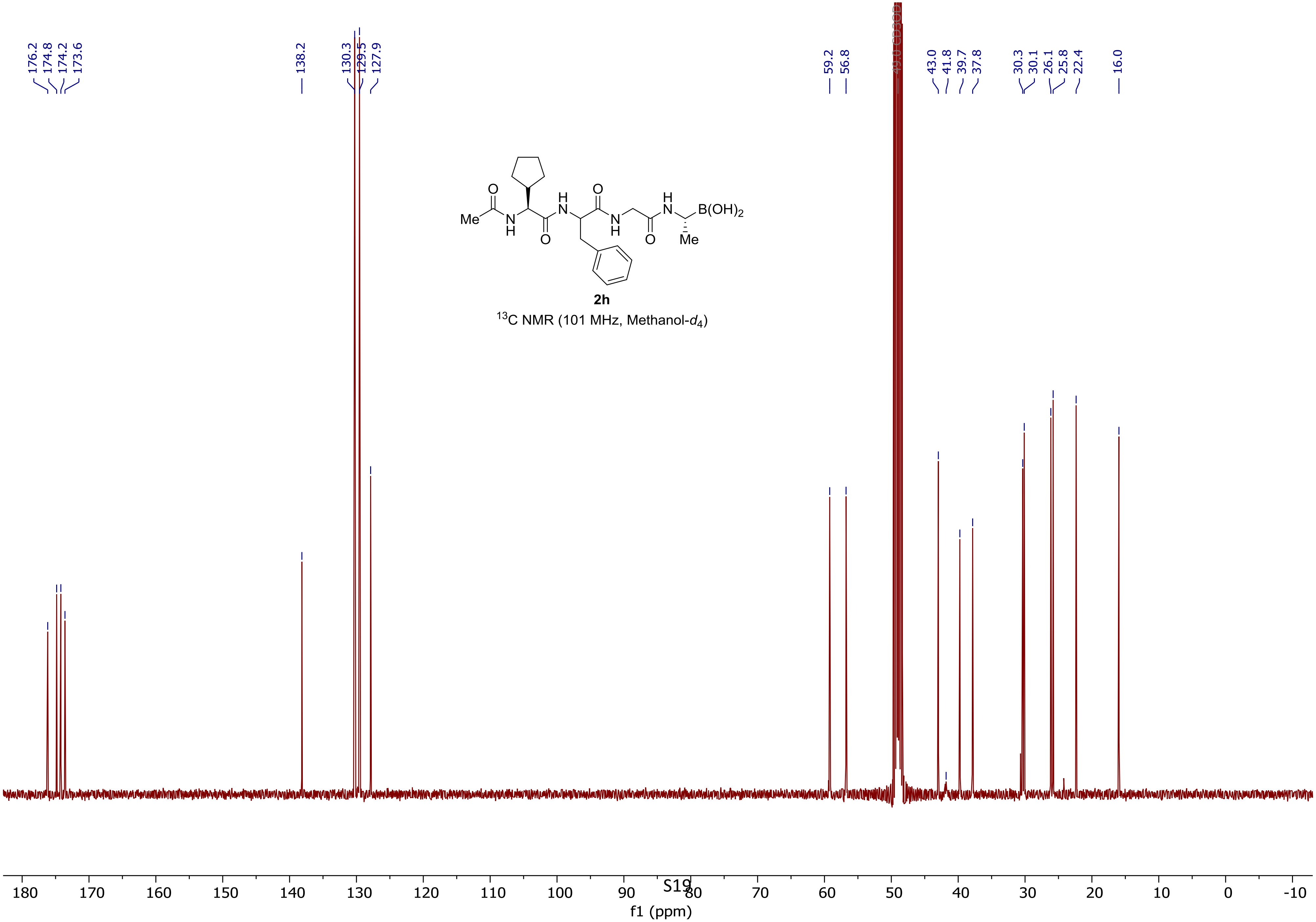


**2h**

$^{13}$C NMR (101 MHz, Methanol-$d_4$)

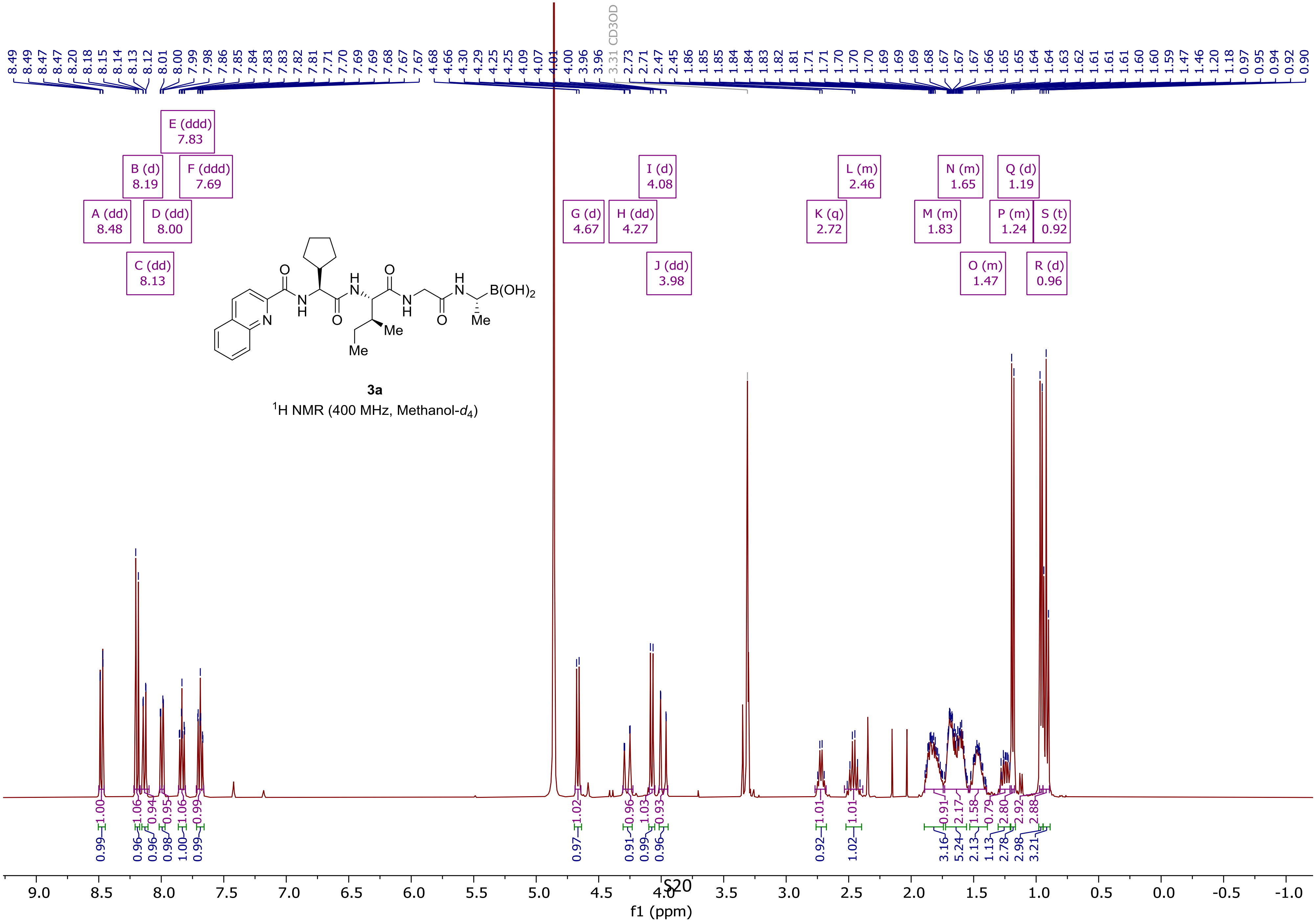


**3a**

$^{1}H$ NMR (400 MHz, Methanol-$d_4$)

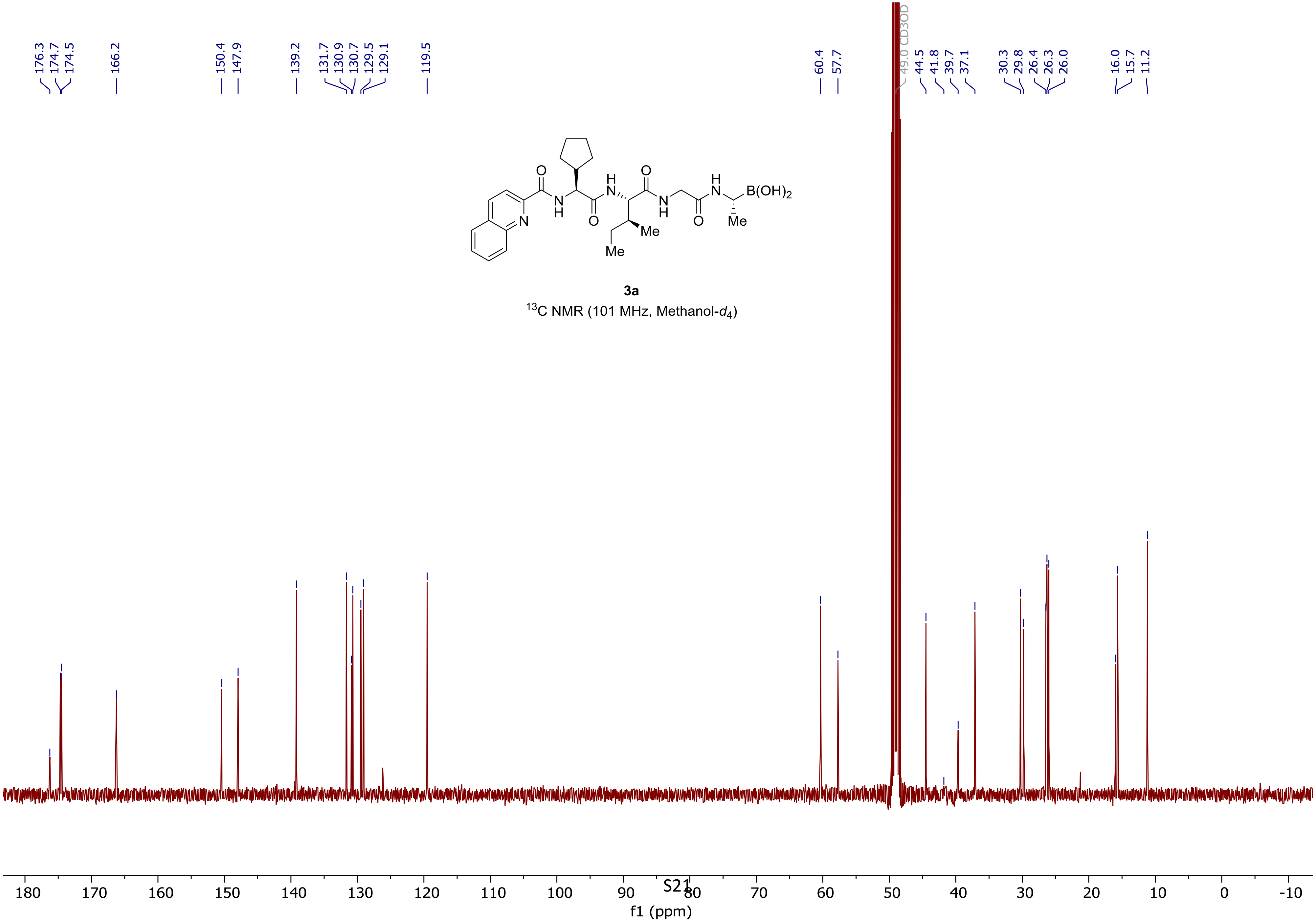
176.3
174.7
174.5
166.2
150.4
147.9
139.2
131.7
130.9
130.7
129.5
129.1
119.5
60.4
57.7
49.0 CD3OD
44.5
41.8
39.7
37.1
30.3
29.8
26.4
26.3
26.0
16.0
15.7
11.2
O
N
H
N
H
N
O
Me
Me
O
N
H
O
H
N
B(OH)2
Me
3a
13C NMR (101 MHz, Methanol-d4)
180
170
160
150
140
130
120
110
100
90
80
70
60
50
40
30
20
10
0
-10
f1 (ppm)

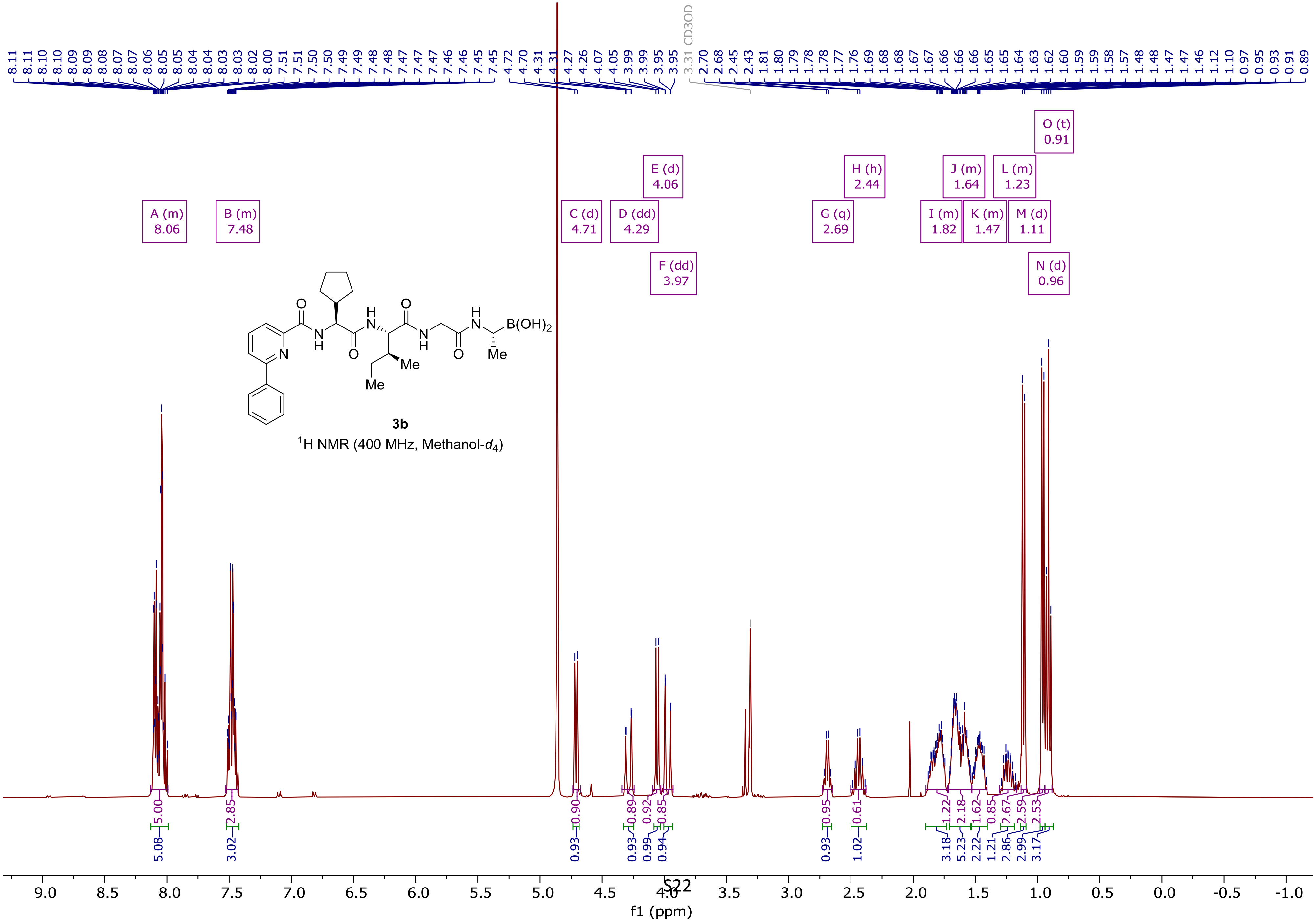
3b
¹H NMR (400 MHz, Methanol-d4)
A (m) 8.06
B (m) 7.48
C (d) 4.71
D (dd) 4.29
E (d) 4.06
F (dd) 3.97
G (q) 2.69
H (h) 2.44
I (m) 1.82
J (m) 1.64
K (m) 1.47
L (m) 1.23
M (d) 1.11
N (d) 0.96
O (t) 0.91
f1 (ppm)

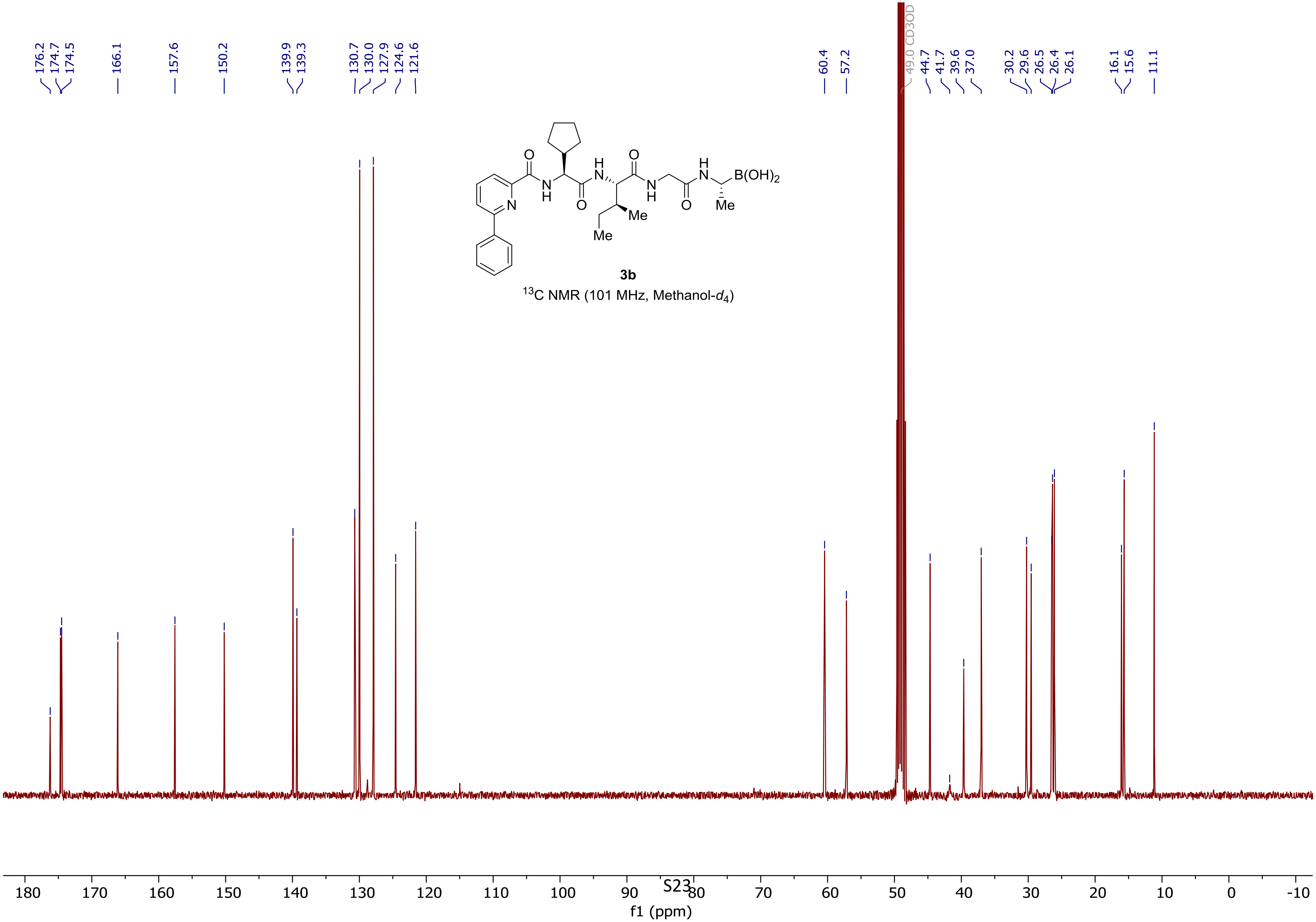
176.2
174.7
174.5
166.1
157.6
150.2
139.9
139.3
130.7
130.0
127.9
124.6
121.6
60.4
57.2
49.0 CD3OD
44.7
41.7
39.6
37.0
30.2
29.6
26.5
26.4
26.1
16.1
15.6
11.1
O
N
H
H
N
O
O
N
H
O
H
N
B(OH)2
Me
Me
Me
N
3b
$^{13}$C NMR (101 MHz, Methanol-$d_4$)
180 170 160 150 140 130 120 110 100 90 80 70 60 50 40 30 20 10 0 -10
f1 (ppm)

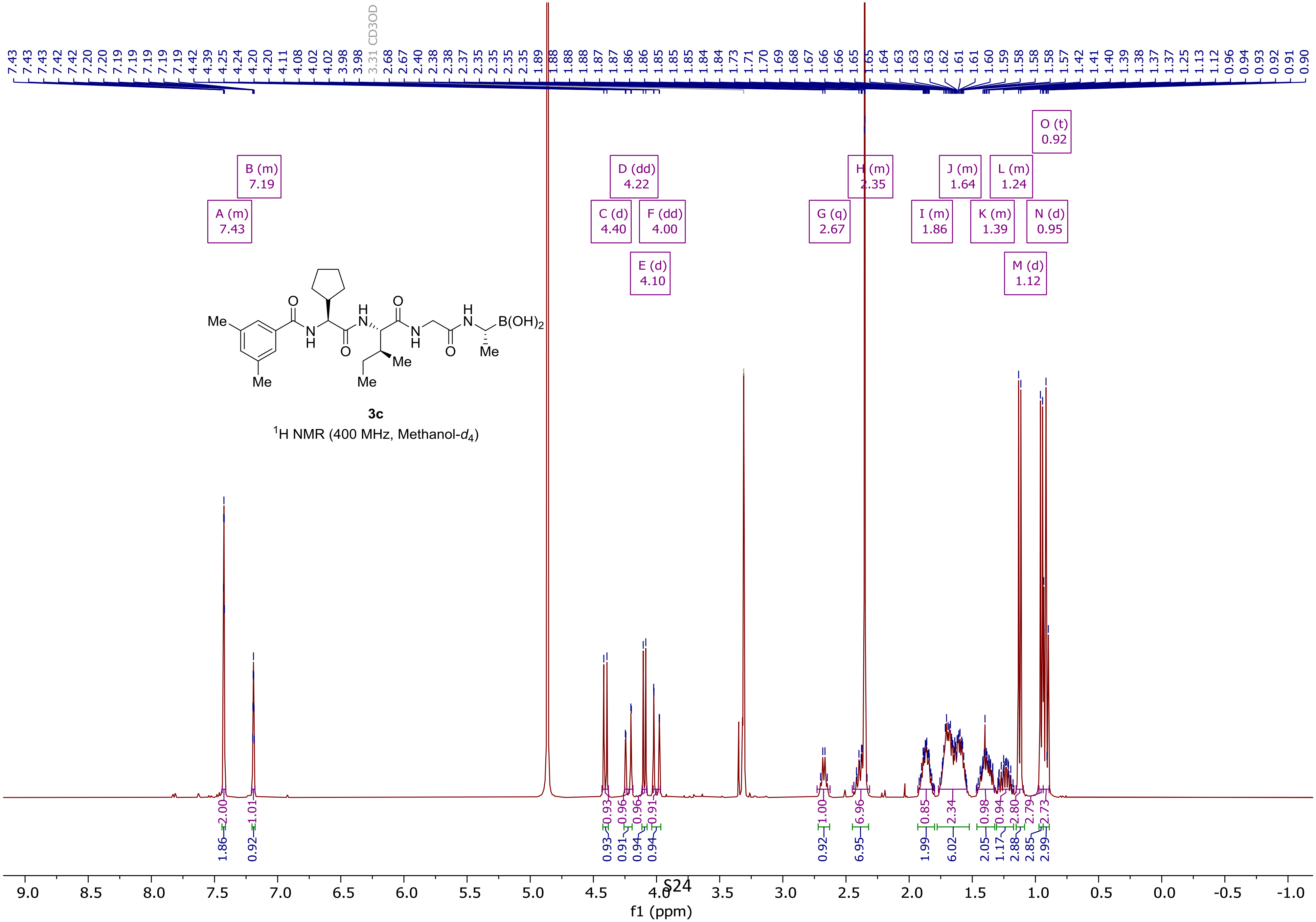
3c
$^{1}$H NMR (400 MHz, Methanol-d4)
A (m) 7.43
B (m) 7.19
C (d) 4.40
D (dd) 4.22
E (d) 4.10
F (dd) 4.00
G (q) 2.67
H (m) 2.35
I (m) 1.86
J (m) 1.64
K (m) 1.39
L (m) 1.24
M (d) 1.12
N (d) 0.95
O (t) 0.92
f1 (ppm)

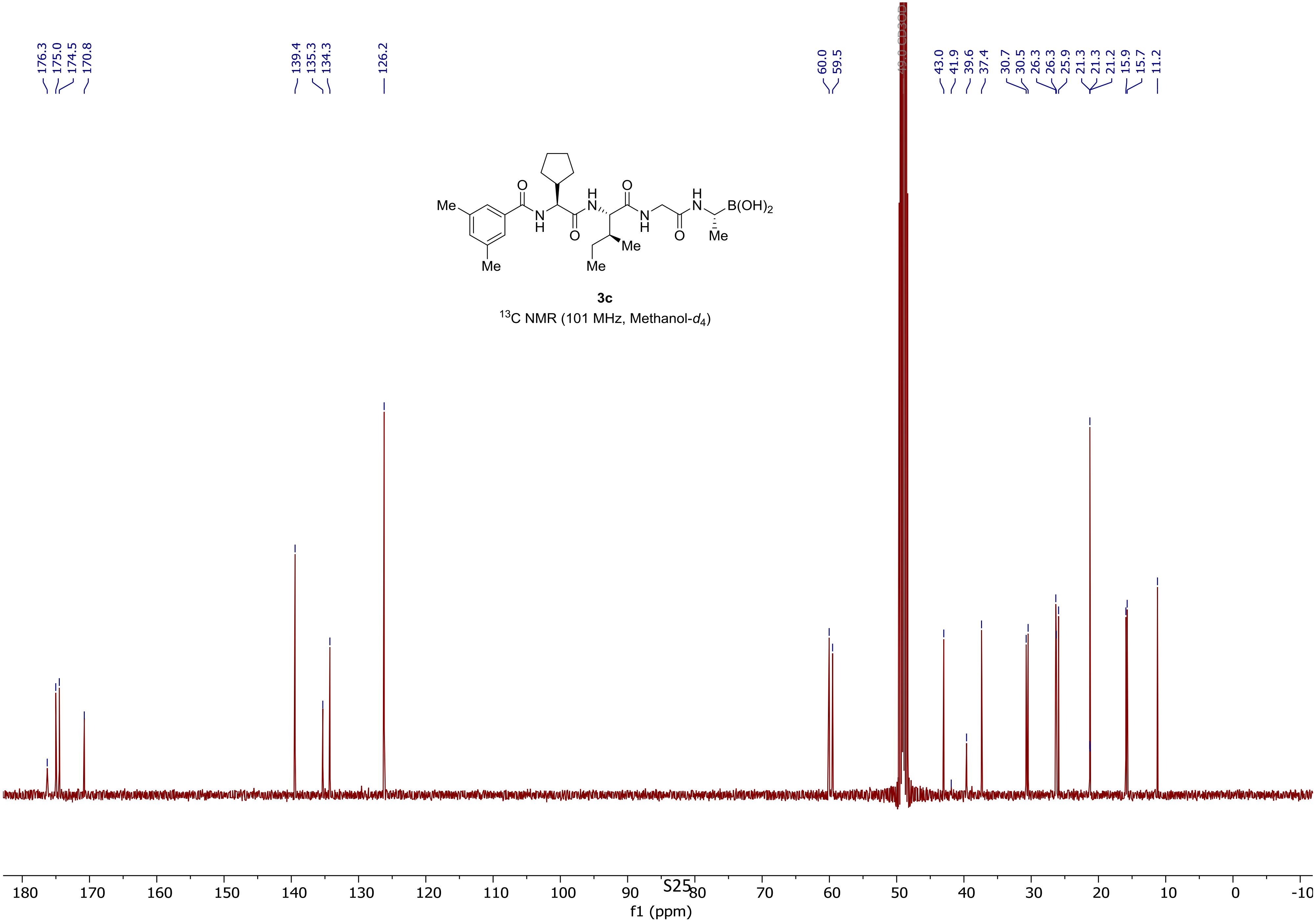
176.3
175.0
174.5
170.8
139.4
135.3
134.3
126.2
60.0
59.5
49.0 CD3OD
43.0
41.9
39.6
37.4
30.7
30.5
26.3
26.3
25.9
21.3
21.3
21.2
15.9
15.7
11.2
Me
O
N
H
O
H
N
O
N
H
O
H
N
B(OH)2
Me
Me
Me
Me
3c
13C NMR (101 MHz, Methanol-d4)
180
170
160
150
140
130
120
110
100
90
80
70
60
50
40
30
20
10
0
-10
f1 (ppm)

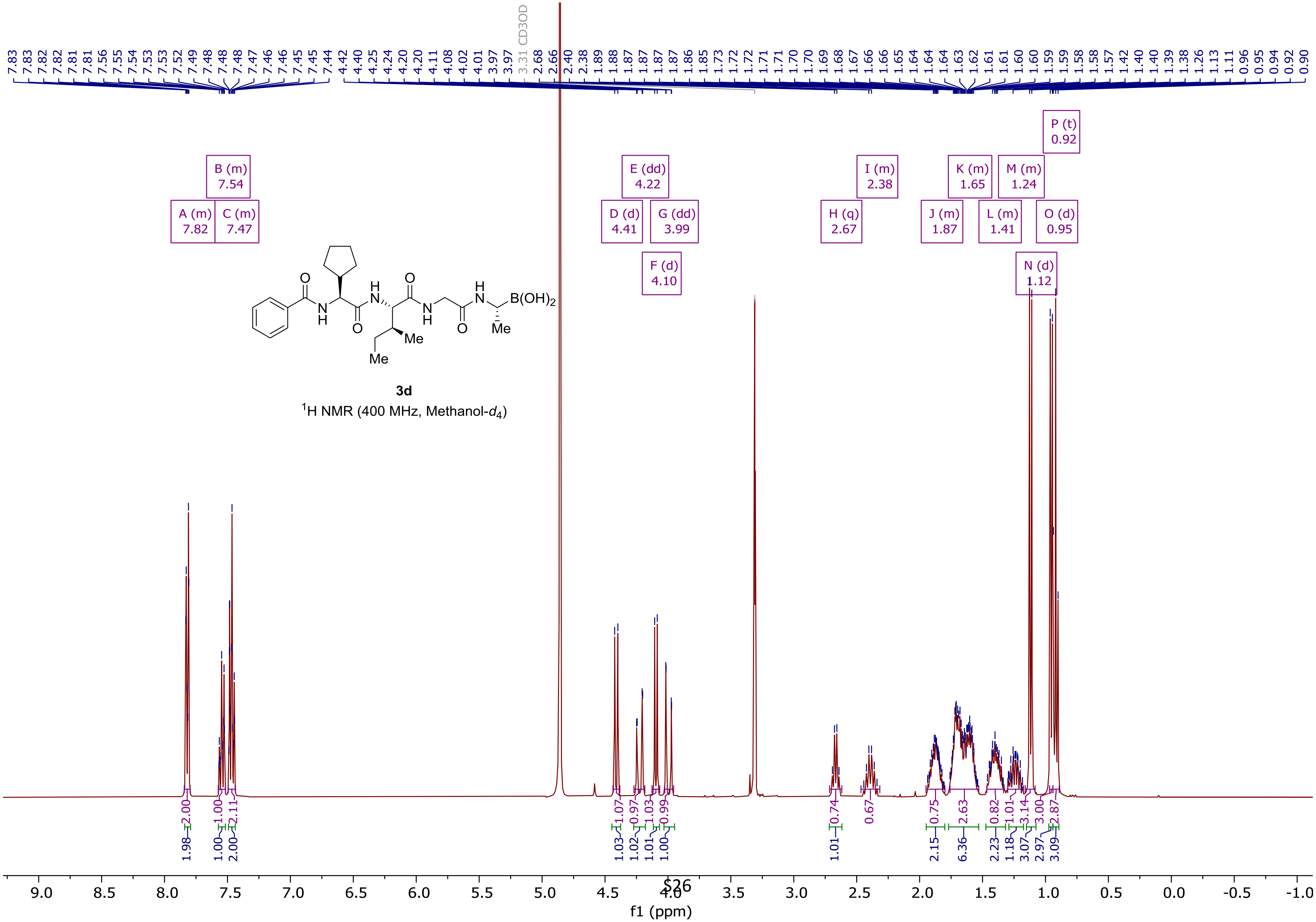
7.83 7.83 7.82 7.82 7.81 7.81 7.56 7.55 7.54 7.53 7.53 7.52 7.49 7.48 7.48 7.47 7.46 7.46 7.45 7.45 7.44 4.42 4.40 4.25 4.24 4.20 4.20 4.11 4.08 4.02 4.01 3.97 3.97 3.31 CD3OD 2.68 2.66 2.40 2.38 1.89 1.88 1.87 1.87 1.87 1.87 1.86 1.85 1.73 1.72 1.72 1.71 1.71 1.70 1.70 1.69 1.68 1.67 1.66 1.66 1.65 1.64 1.64 1.64 1.63 1.62 1.61 1.61 1.60 1.60 1.59 1.59 1.58 1.58 1.57 1.42 1.40 1.40 1.39 1.38 1.26 1.13 1.11 0.96 0.95 0.94 0.92 0.90
A (m) 7.82
B (m) 7.54
C (m) 7.47
D (d) 4.41
E (dd) 4.22
F (d) 4.10
G (dd) 3.99
H (q) 2.67
I (m) 2.38
J (m) 1.87
K (m) 1.65
L (m) 1.41
M (m) 1.24
N (d) 1.12
O (d) 0.95
P (t) 0.92
B(OH)2
Me
3d
1H NMR (400 MHz, Methanol-d4)
2.00 1.00 2.11 1.07 0.97 1.03 0.99 0.74 0.67 0.75 2.63 0.82 1.01 3.14 3.00 2.87
1.98 1.00 2.00 1.03 1.02 1.01 1.00 1.01 2.15 6.36 2.23 1.18 3.07 2.97 3.09
9.0 8.5 8.0 7.5 7.0 6.5 6.0 5.5 5.0 4.5 4.0 3.5 3.0 2.5 2.0 1.5 1.0 0.5 0.0 -0.5 -1.0
f1 (ppm)

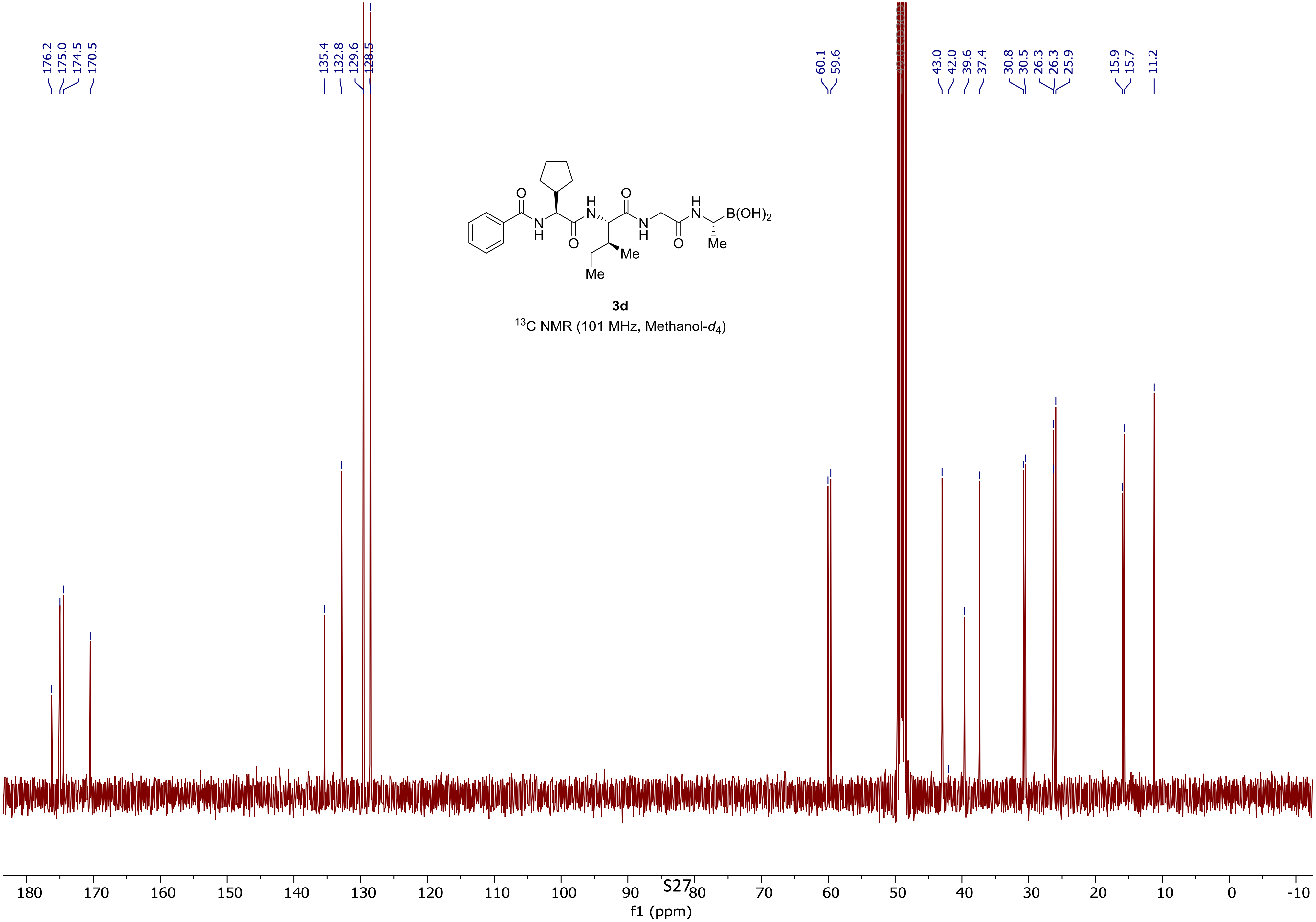
176.2
175.0
174.5
170.5
135.4
132.8
129.6
128.5
60.1
59.6
49.0 CD3OD
43.0
42.0
39.6
37.4
30.8
30.5
26.3
26.3
25.9
15.9
15.7
11.2
B(OH)$_2$
Me
Me
Me
3d
$^{13}$C NMR (101 MHz, Methanol-$d_4$)
180 170 160 150 140 130 120 110 100 90 80 70 60 50 40 30 20 10 0 -10
f1 (ppm)

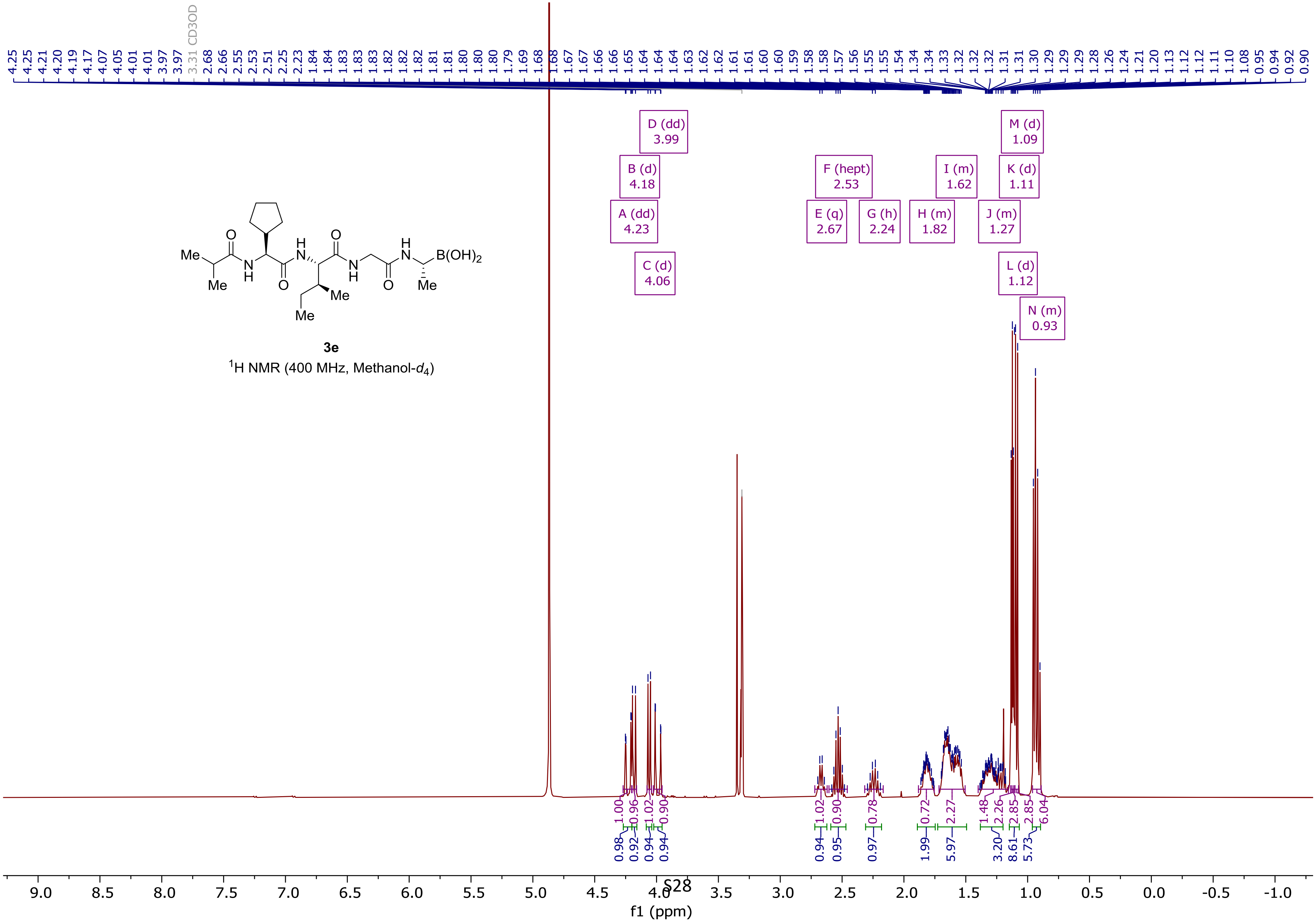


**3e**

$^1$H NMR (400 MHz, Methanol-$d_4$)

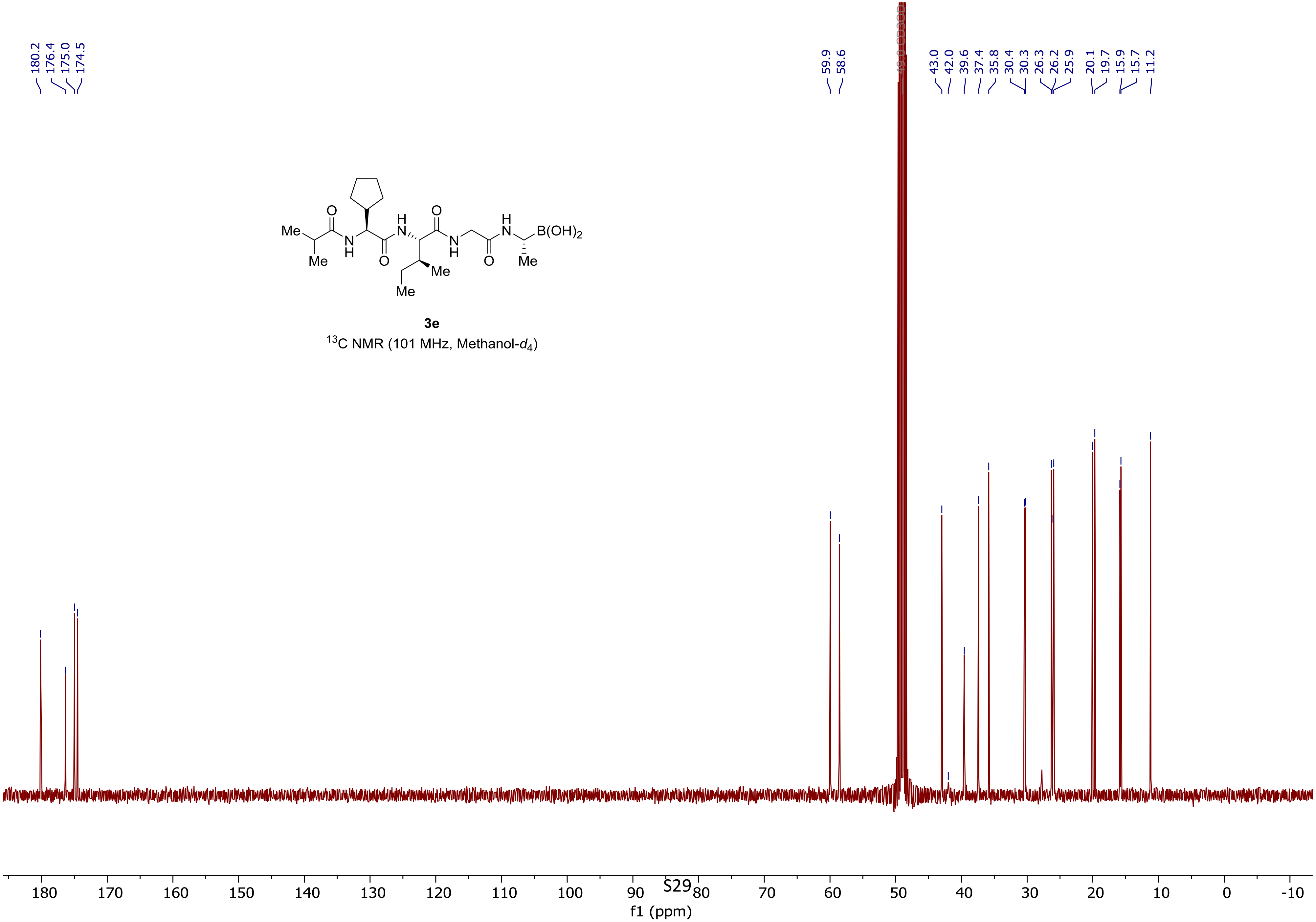


**3e**

$^{13}$C NMR (101 MHz, Methanol-$d_4$)

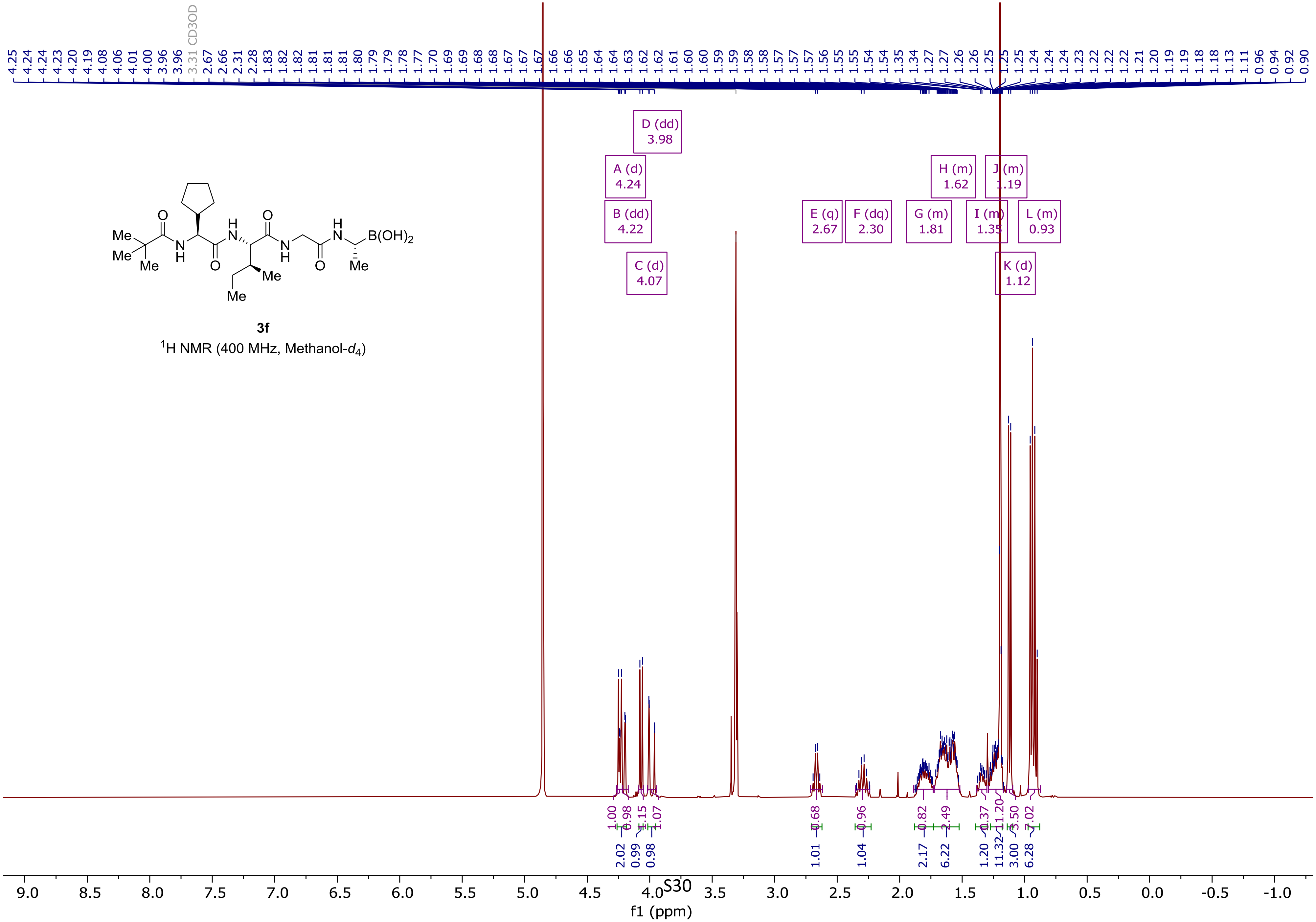
4.25 4.24 4.24 4.23 4.20 4.19 4.08 4.06 4.01 4.00 3.96 3.96 3.31 CD3OD 2.67 2.66 2.31 2.28 1.83 1.82 1.82 1.81 1.81 1.81 1.80 1.79 1.79 1.78 1.77 1.70 1.69 1.69 1.68 1.68 1.67 1.67 1.67 1.66 1.66 1.65 1.64 1.64 1.63 1.62 1.62 1.61 1.60 1.60 1.59 1.59 1.58 1.58 1.57 1.57 1.57 1.56 1.55 1.55 1.54 1.54 1.35 1.34 1.27 1.27 1.26 1.26 1.25 1.25 1.25 1.24 1.24 1.24 1.23 1.22 1.22 1.22 1.21 1.20 1.19 1.19 1.18 1.18 1.13 1.11 0.96 0.94 0.92 0.90
D (dd) 3.98
A (d) 4.24
B (dd) 4.22
C (d) 4.07
E (q) 2.67
F (dq) 2.30
G (m) 1.81
H (m) 1.62
I (m) 1.35
J (m) 1.19
K (d) 1.12
L (m) 0.93
Me
Me
Me
N
H
O
O
H
N
Me
Me
O
N
H
O
H
N
B(OH)$_2$
Me
3f
$^1$H NMR (400 MHz, Methanol-$d_4$)
2.02 0.99 0.98 1.01 1.04 2.17 6.22 1.20 11.32 3.00 6.28
9.0 8.5 8.0 7.5 7.0 6.5 6.0 5.5 5.0 4.5 4.0 3.5 3.0 2.5 2.0 1.5 1.0 0.5 0.0 -0.5 -1.0
f1 (ppm)

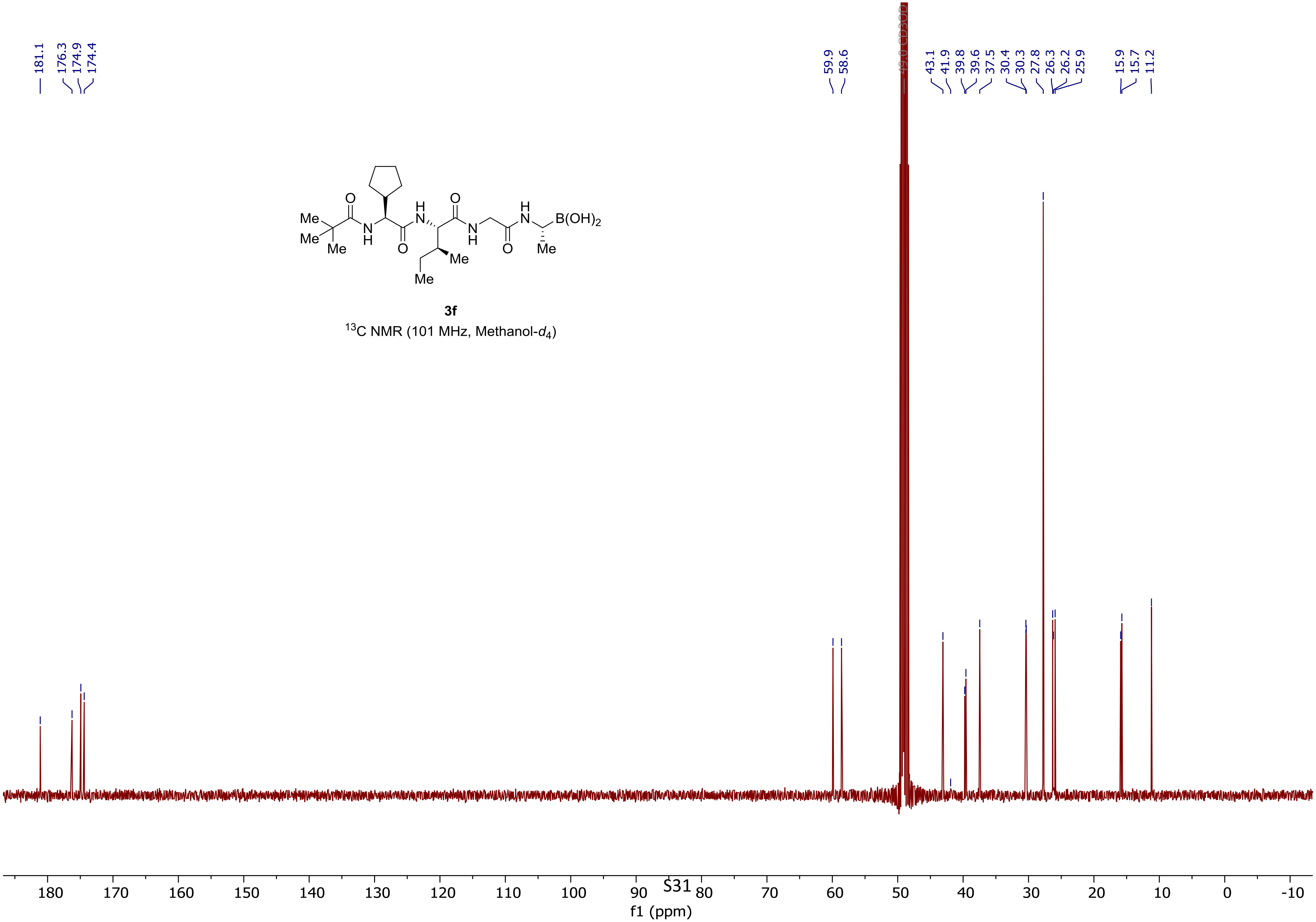
181.1
176.3
174.9
174.4
59.9
58.6
49.0 CD3OD
43.1
41.9
39.8
39.6
37.5
30.4
30.3
27.8
26.3
26.2
25.9
15.9
15.7
11.2
Me
Me
Me
O
N
H
O
H
N
O
N
H
O
H
N
B(OH)2
Me
Me
Me
3f
13C NMR (101 MHz, Methanol-d4)
f1 (ppm)

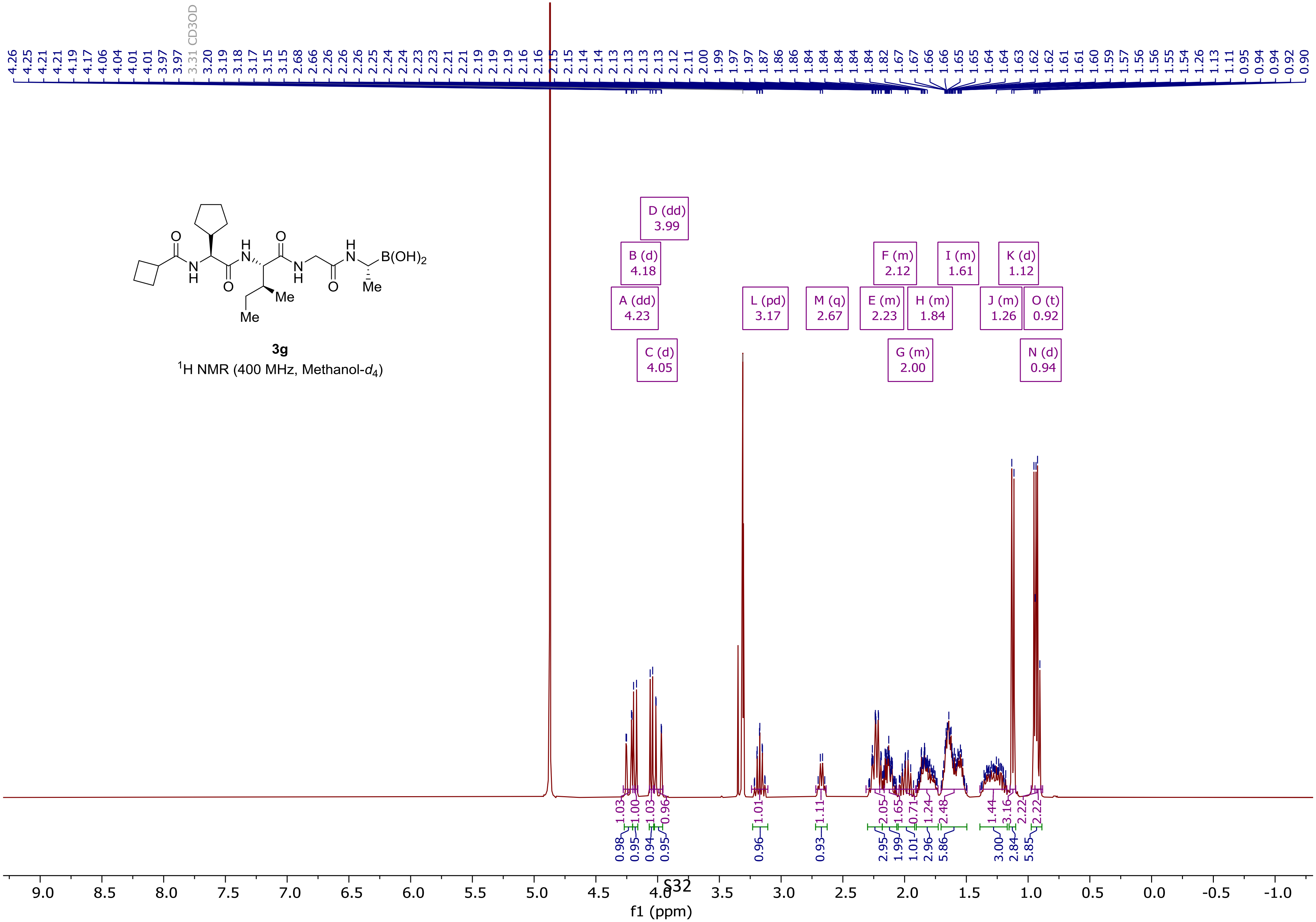
3g
$^1$H NMR (400 MHz, Methanol-$d_4$)
B(OH)$_2$
Me
CD3OD
A (dd) 4.23
B (d) 4.18
C (d) 4.05
D (dd) 3.99
E (m) 2.23
F (m) 2.12
G (m) 2.00
H (m) 1.84
I (m) 1.61
J (m) 1.26
K (d) 1.12
L (pd) 3.17
M (q) 2.67
N (d) 0.94
O (t) 0.92
f1 (ppm)

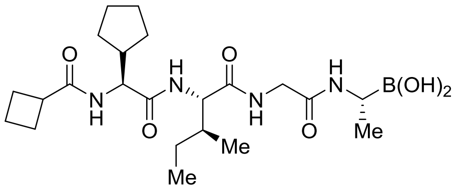

**3g**

$^{13}C$ NMR (101 MHz, Methanol-$d_4$)

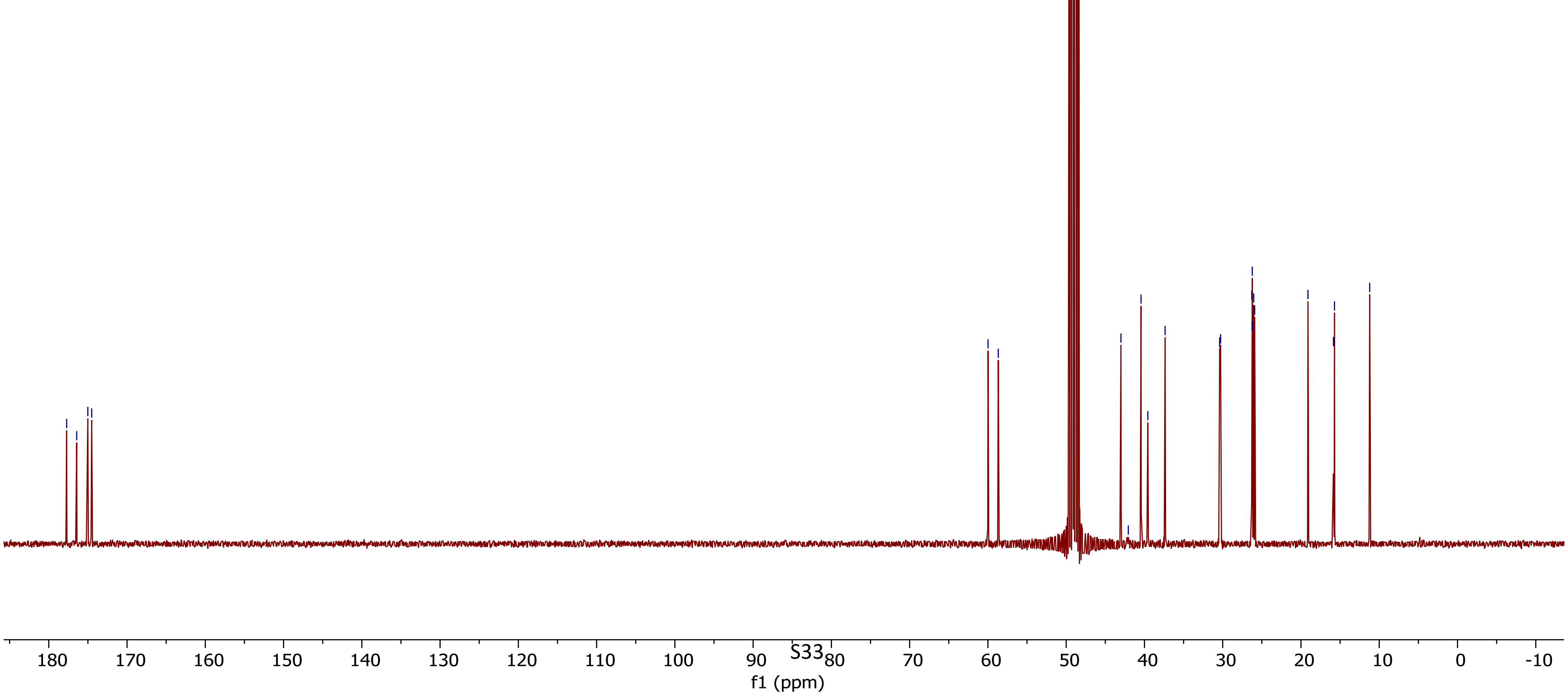

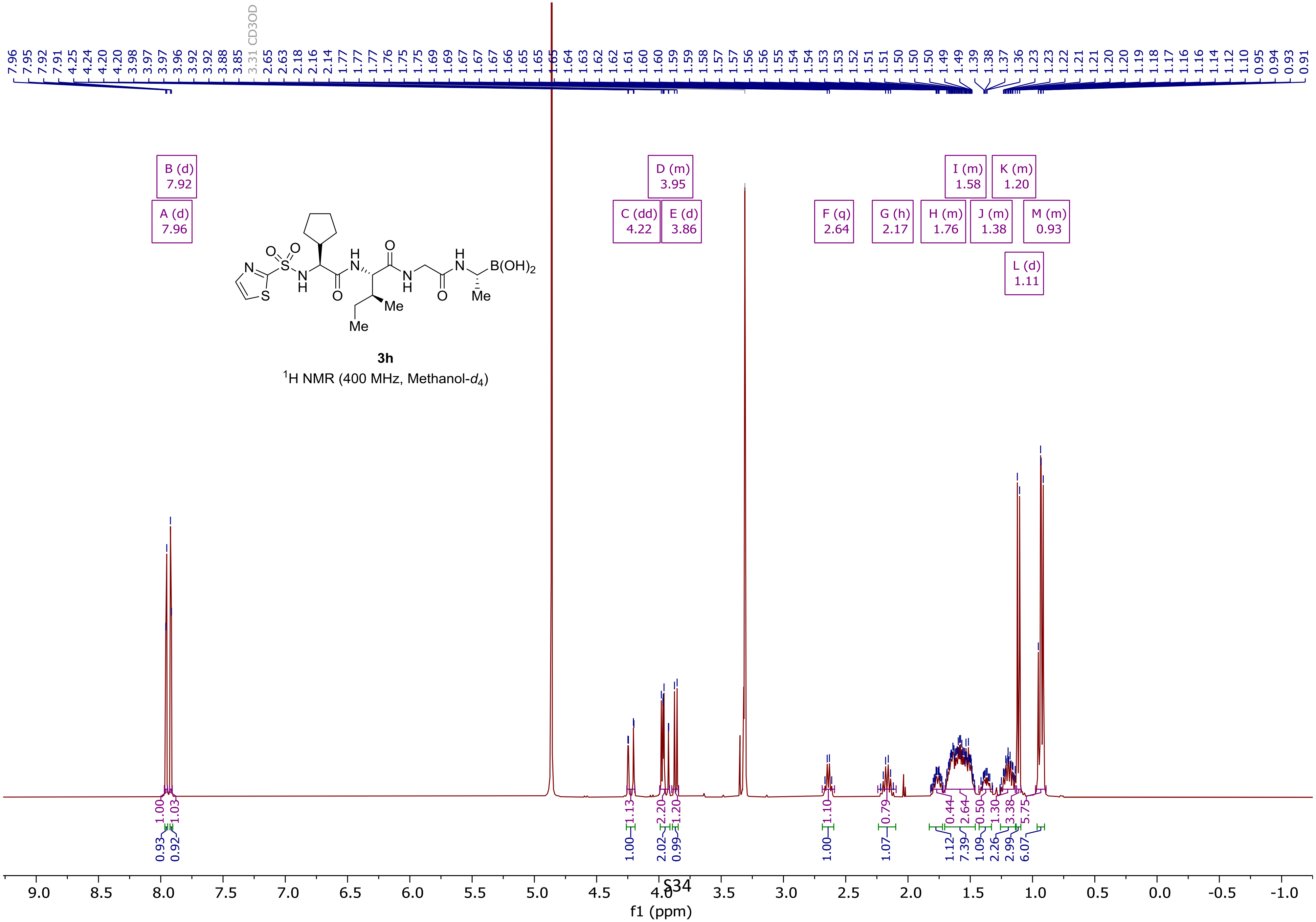
7.96 7.95 7.92 7.91 4.25 4.24 4.20 4.20 3.98 3.97 3.97 3.96 3.92 3.92 3.88 3.85 3.31 CD3OD 2.65 2.63 2.18 2.16 2.14 1.77 1.77 1.77 1.76 1.75 1.75 1.69 1.69 1.67 1.67 1.67 1.66 1.65 1.65 1.65 1.64 1.63 1.62 1.62 1.61 1.60 1.60 1.59 1.59 1.58 1.57 1.57 1.56 1.56 1.55 1.54 1.54 1.53 1.53 1.52 1.51 1.51 1.50 1.50 1.50 1.49 1.49 1.39 1.38 1.37 1.36 1.23 1.23 1.22 1.21 1.21 1.20 1.20 1.19 1.18 1.17 1.16 1.16 1.14 1.12 1.10 0.95 0.94 0.93 0.91
B (d) 7.92
A (d) 7.96
D (m) 3.95
C (dd) 4.22
E (d) 3.86
F (q) 2.64
G (h) 2.17
H (m) 1.76
I (m) 1.58
J (m) 1.38
K (m) 1.20
L (d) 1.11
M (m) 0.93
B(OH)2
Me
Me
Me
3h
1H NMR (400 MHz, Methanol-d4)
f1 (ppm)

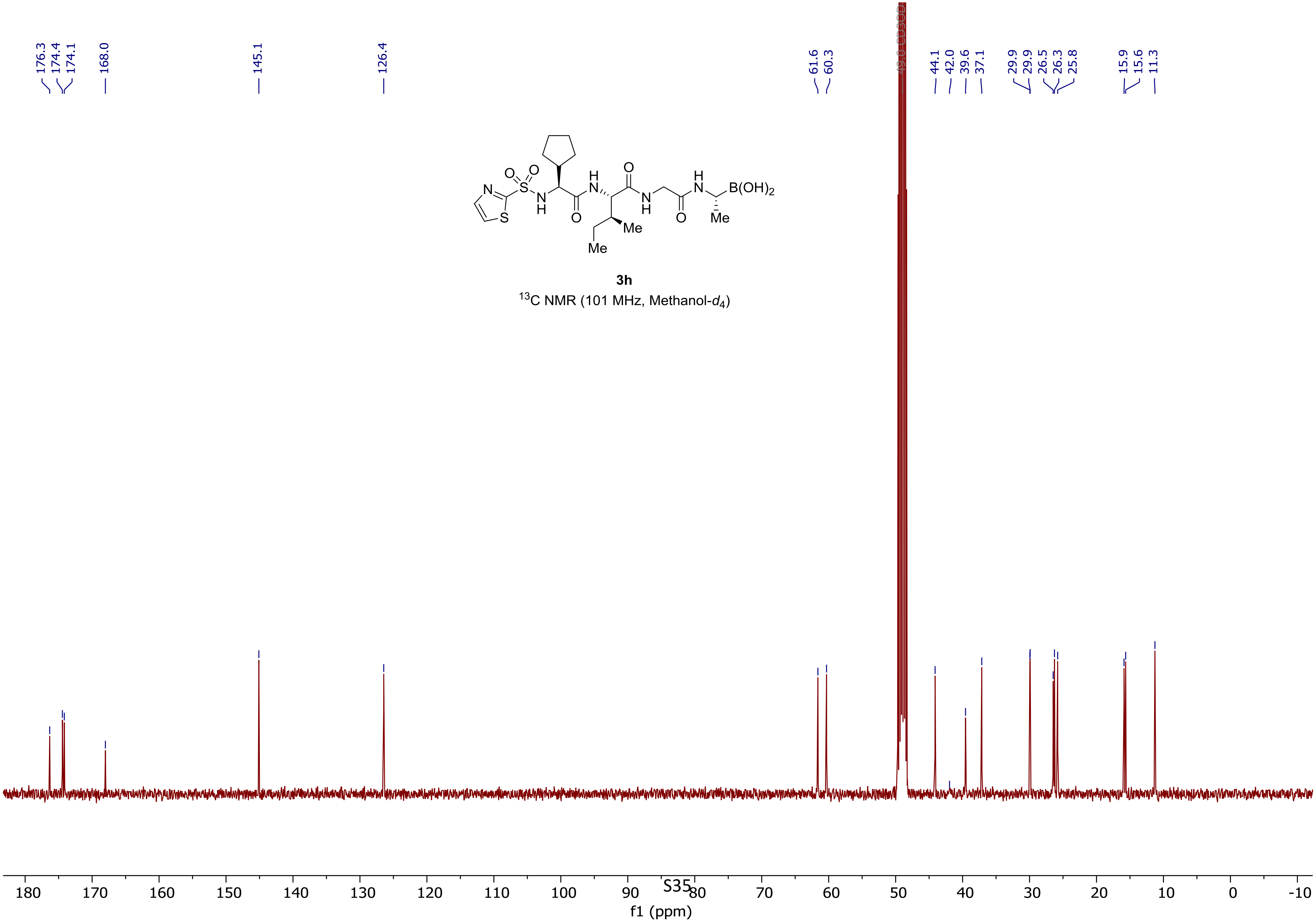
176.3
174.4
174.1
168.0
145.1
126.4
61.6
60.3
49.0 CD3OD
44.1
42.0
39.6
37.1
29.9
29.9
26.5
26.3
25.8
15.9
15.6
11.3
B(OH)2
Me
Me
Me
3h
13C NMR (101 MHz, Methanol-d4)
180
170
160
150
140
130
120
110
100
90
80
70
60
50
40
30
20
10
0
-10
f1 (ppm)

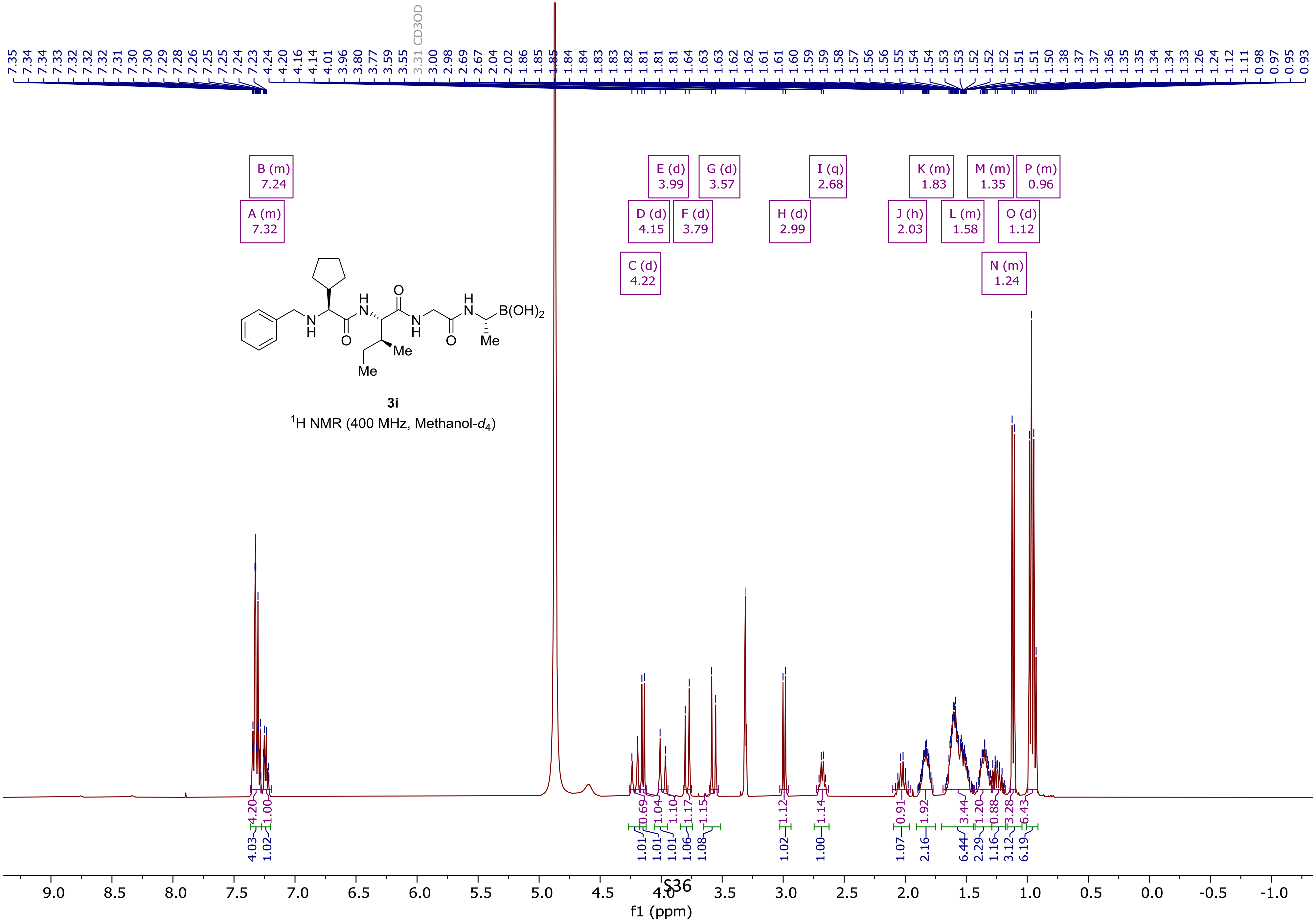


**3i**

$^1$H NMR (400 MHz, Methanol-$d_4$)

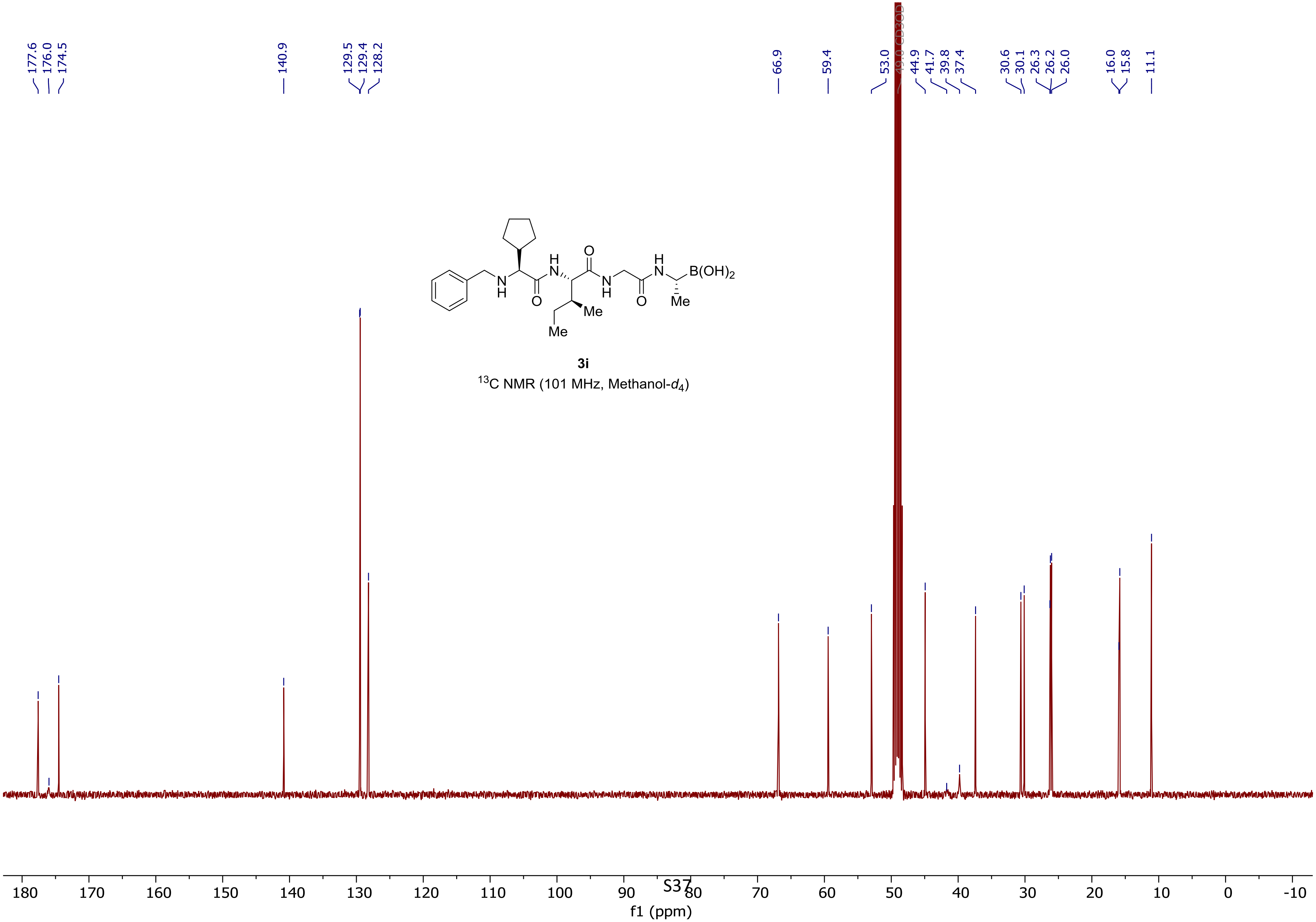
177.6
176.0
174.5
140.9
129.5
129.4
128.2
66.9
59.4
53.0
49.0 CD3OD
44.9
41.7
39.8
37.4
30.6
30.1
26.3
26.2
26.0
16.0
15.8
11.1
3i
$^{13}$C NMR (101 MHz, Methanol-$d_4$)
f1 (ppm)
180 170 160 150 140 130 120 110 100 90 80 70 60 50 40 30 20 10 0 -10
B(OH)$_2$
Me

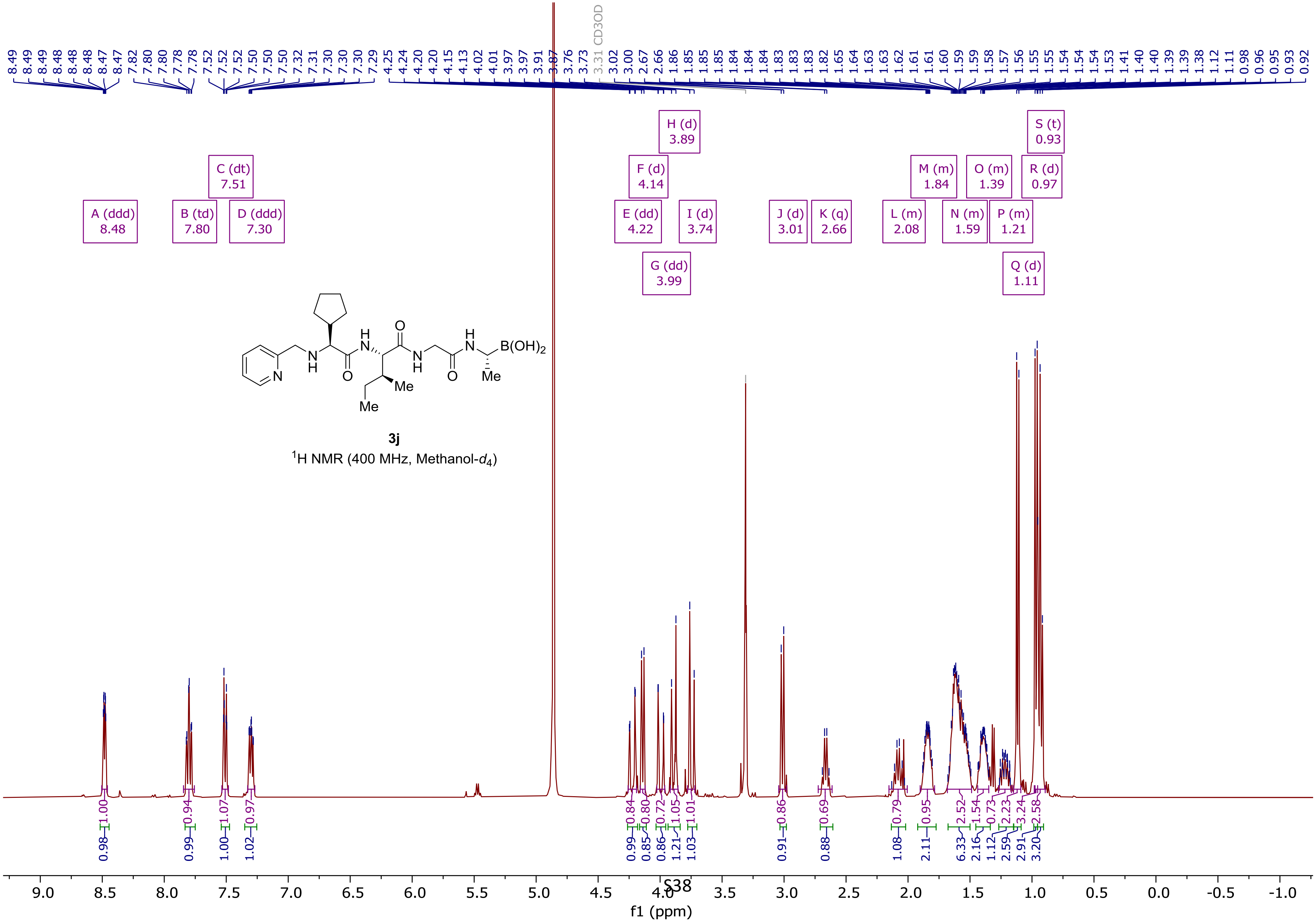


**3j**

$^{1}$H NMR (400 MHz, Methanol-$d_4$)

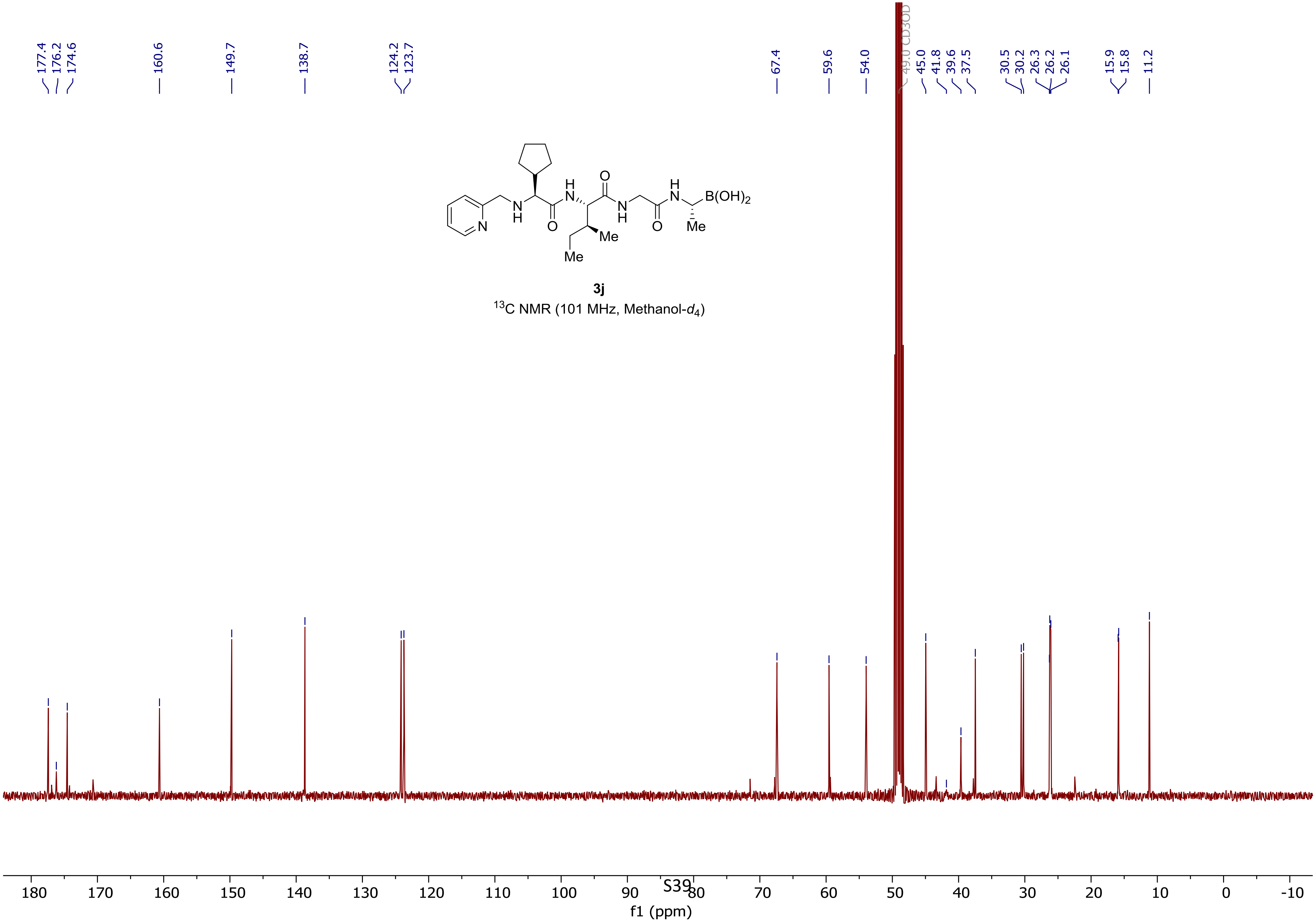


**3j**

$^{13}$C NMR (101 MHz, Methanol-$d_4$)

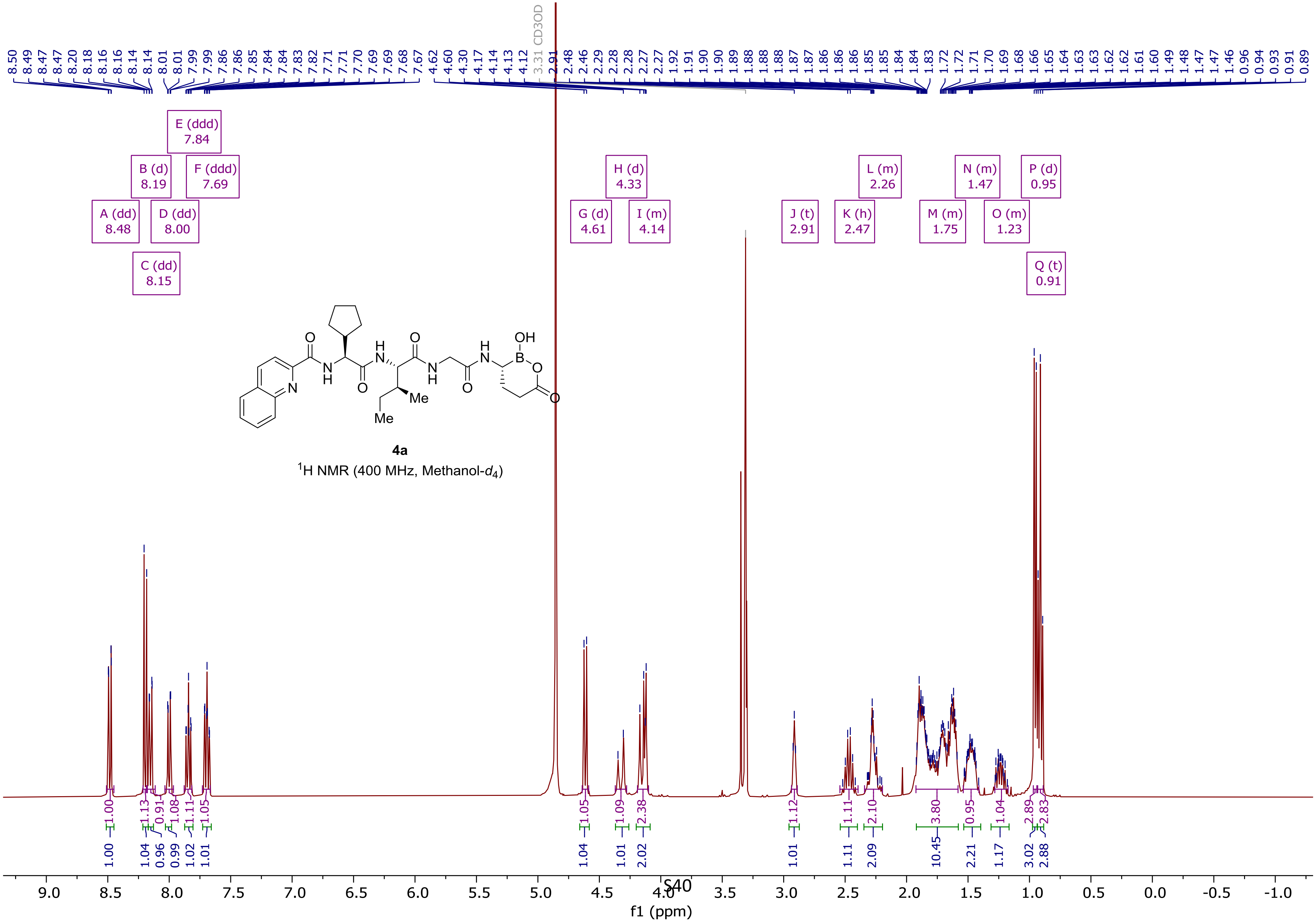


**4a**

$^{1}$H NMR (400 MHz, Methanol-$d_4$)

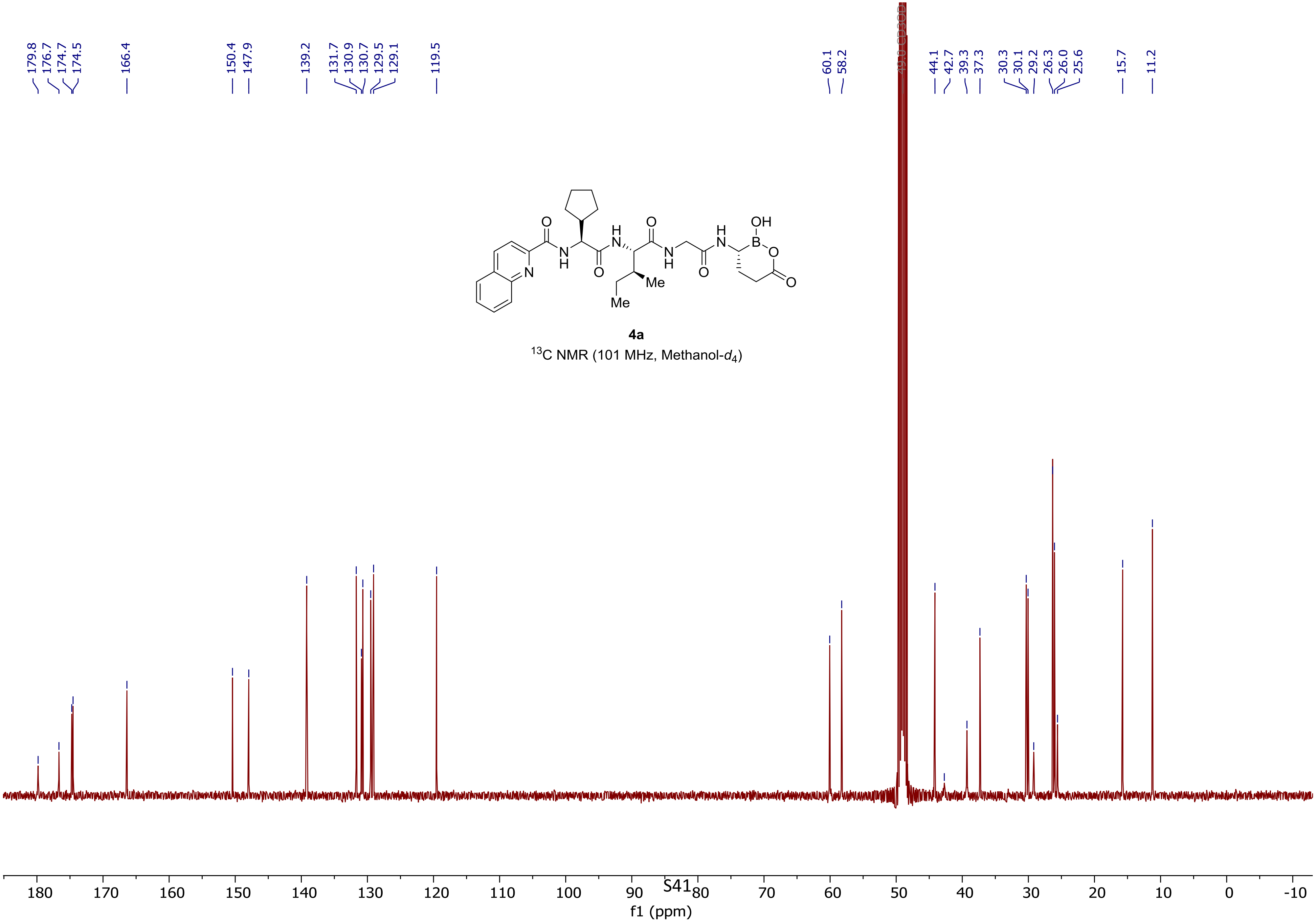
179.8
176.7
174.7
174.5
166.4
150.4
147.9
139.2
131.7
130.9
130.7
129.5
129.1
119.5
60.1
58.2
44.1
42.7
39.3
37.3
30.3
30.1
29.2
26.3
26.0
25.6
15.7
11.2
4a
$^{13}$C NMR (101 MHz, Methanol-$d_4$)
f1 (ppm)

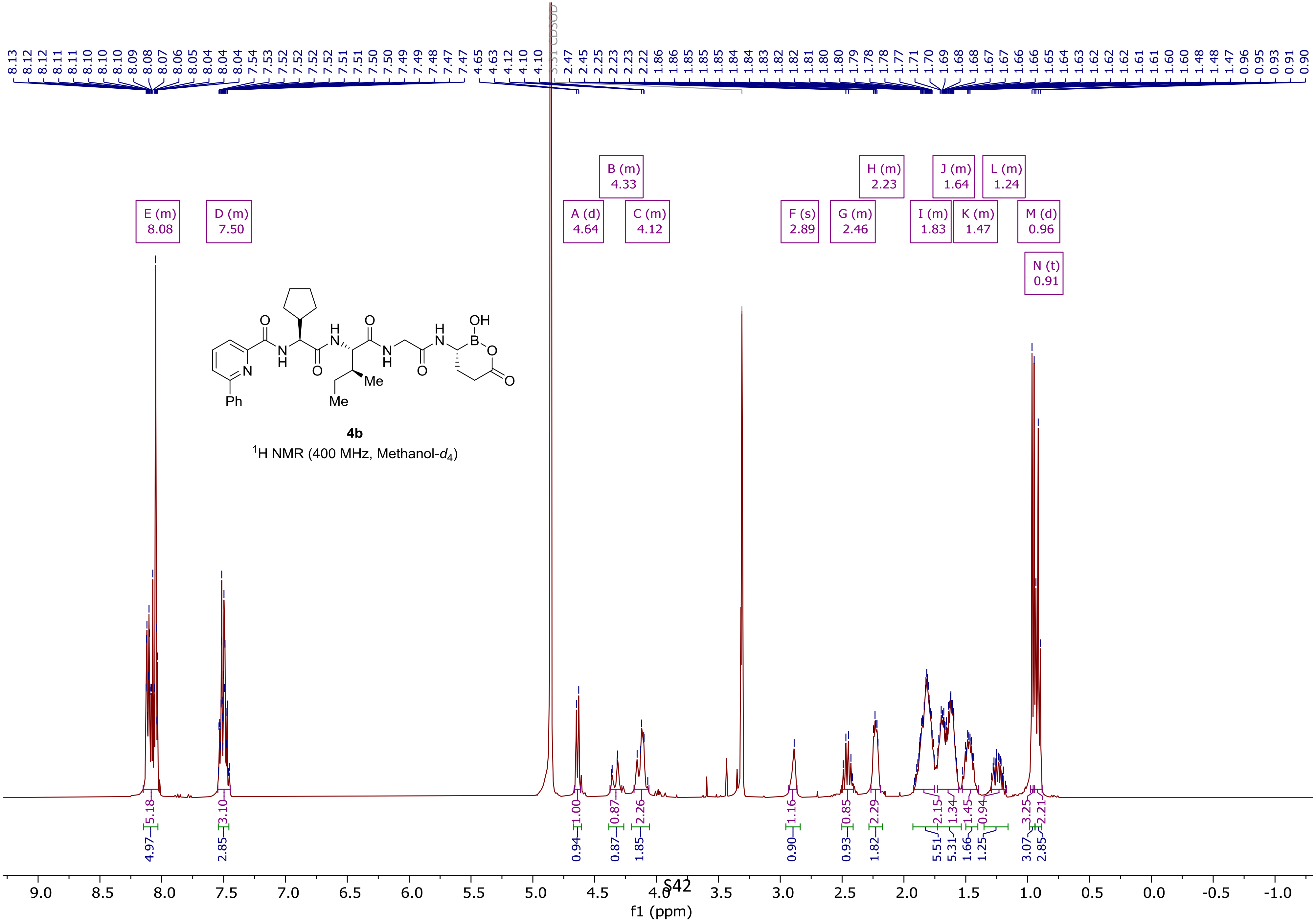
8.13 8.12 8.12 8.11 8.11 8.10 8.10 8.10 8.09 8.08 8.07 8.06 8.05 8.04 8.04 8.04 7.54 7.53 7.52 7.52 7.52 7.52 7.51 7.51 7.50 7.50 7.49 7.49 7.48 7.47 7.47 4.65 4.63 4.12 4.10 4.10 3.31 CD3OD 2.47 2.45 2.25 2.23 2.23 2.22 1.86 1.86 1.85 1.85 1.85 1.84 1.84 1.83 1.82 1.82 1.81 1.80 1.80 1.79 1.78 1.78 1.77 1.71 1.70 1.69 1.68 1.68 1.67 1.67 1.66 1.66 1.65 1.64 1.63 1.62 1.62 1.62 1.61 1.61 1.60 1.60 1.48 1.48 1.47 0.96 0.95 0.93 0.91 0.90
E (m) 8.08
D (m) 7.50
A (d) 4.64
B (m) 4.33
C (m) 4.12
F (s) 2.89
G (m) 2.46
H (m) 2.23
I (m) 1.83
J (m) 1.64
K (m) 1.47
L (m) 1.24
M (d) 0.96
N (t) 0.91
OH
B
O
O
N
H
Me
Ph
Me
4b
1H NMR (400 MHz, Methanol-d4)
f1 (ppm)

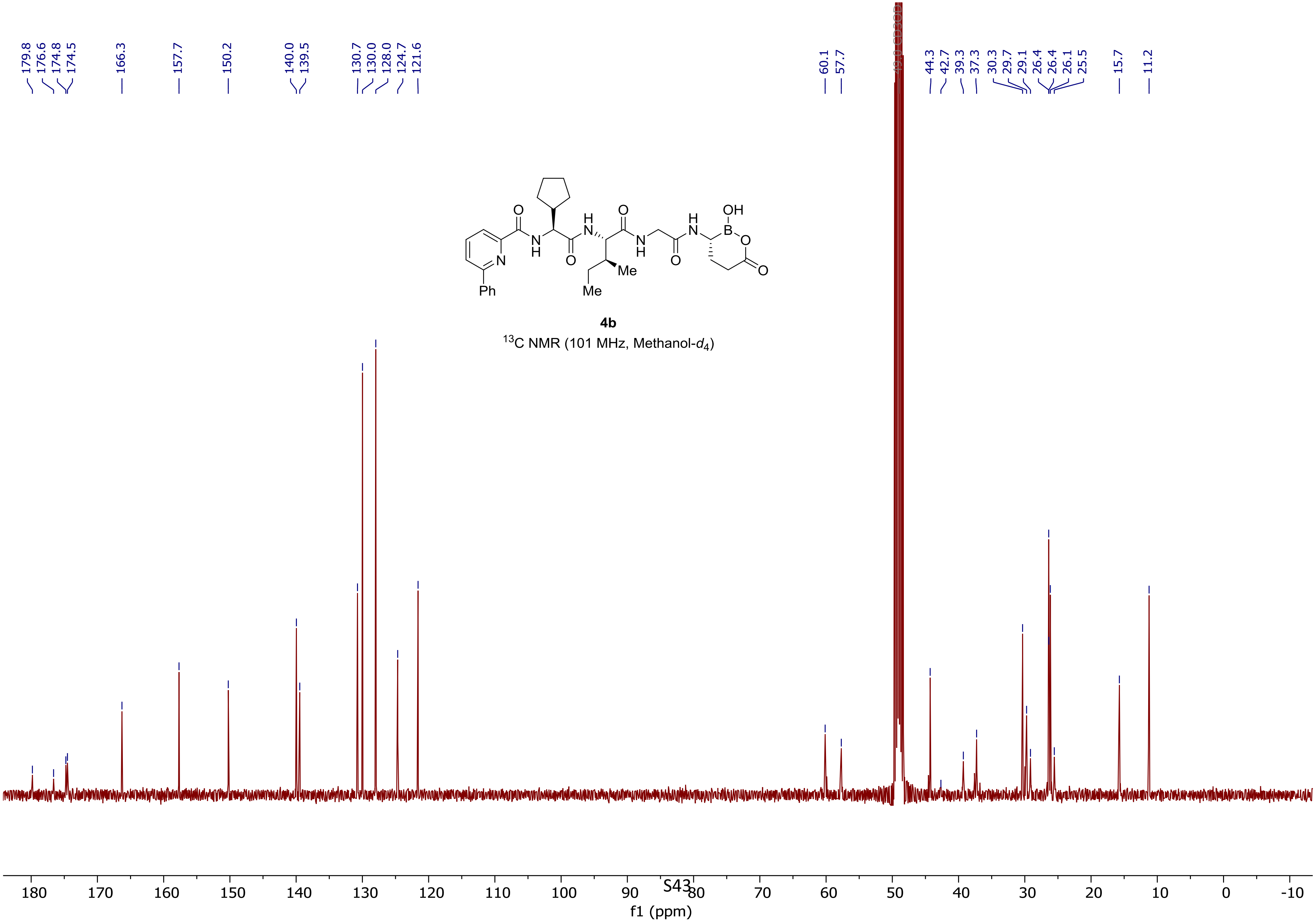


**4b**

$^{13}C$ NMR (101 MHz, Methanol-$d_4$)

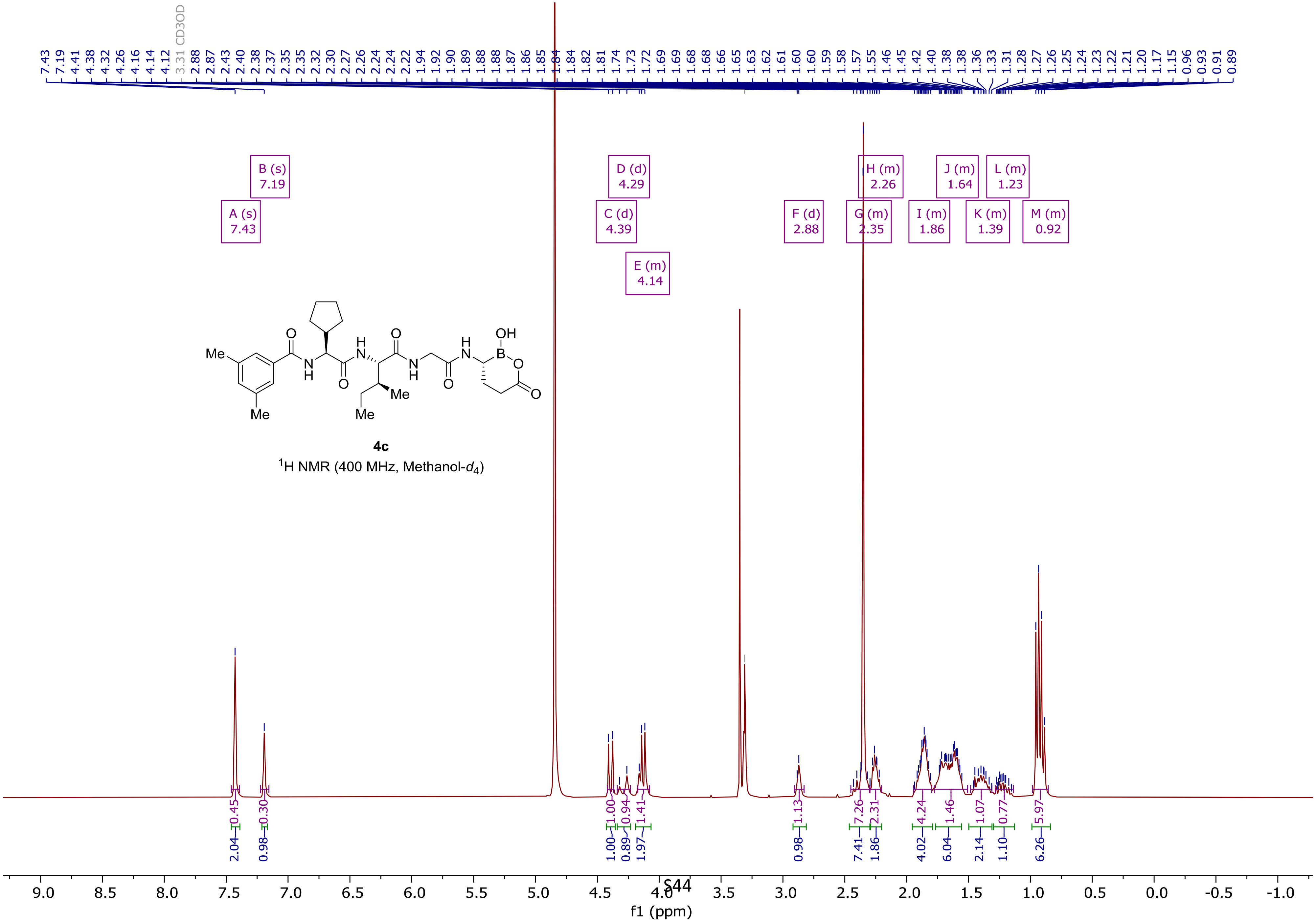


**4c**

$^{1}$H NMR (400 MHz, Methanol-$d_4$)

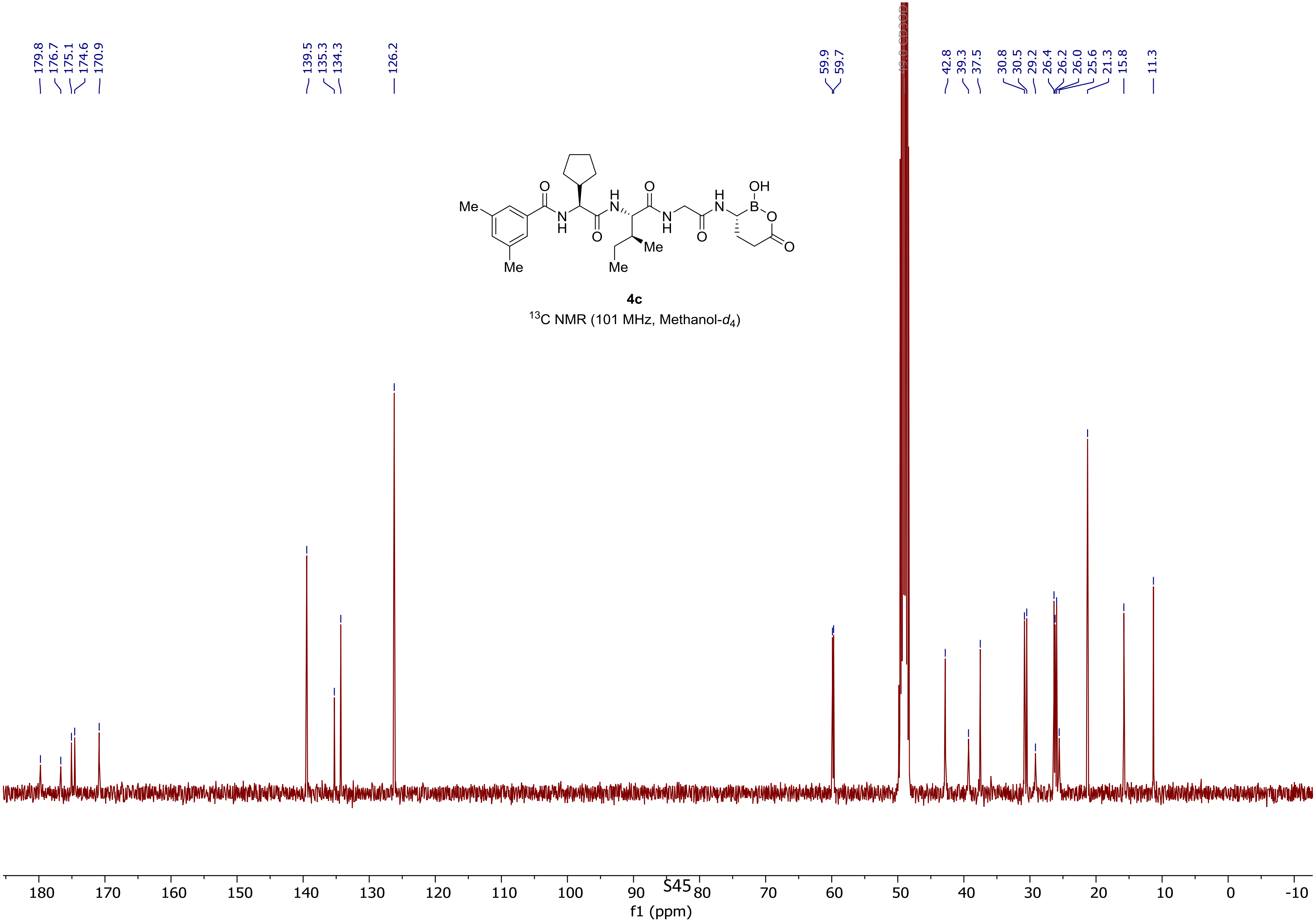
179.8
176.7
175.1
174.6
170.9
139.5
135.3
134.3
126.2
59.9
59.7
49.0 CD3OD
42.8
39.3
37.5
30.8
30.5
29.2
26.4
26.2
26.0
25.6
21.3
15.8
11.3
Me
Me
O
H
N
O
H
N
O
N
H
Me
Me
O
H
N
OH
B
O
O
4c
$^{13}$C NMR (101 MHz, Methanol-$d_4$)
180
170
160
150
140
130
120
110
100
90
80
70
60
50
40
30
20
10
0
-10
f1 (ppm)

## 5. Representative examples of HPLC purity

**SAMPLE INFORMATION**

| | | | |
|---|---|---|---|
| Sample Name: | osm-EP_1506 | Acquired By: | Olita |
| Sample Type: | Unknown | Sample Set Name: | 26042024p |
| Vial: | 5 | Acq. Method Set: | MeCN_0_5min_100% |
| Injection #: | 1 | Processing Method: | Processing |
| Injection Volume: | 30.00 ul | Channel Name: | W2489 ChA |
| Run Time: | 20.0 Minutes | Proc. Chnl. Descr.: | W2489 ChA 210nm |
| Date Acquired: | 2024.04.26. 15:31:28 EET | | |
| Date Processed: | 2024.04.26. 16:24:34 EET | | |

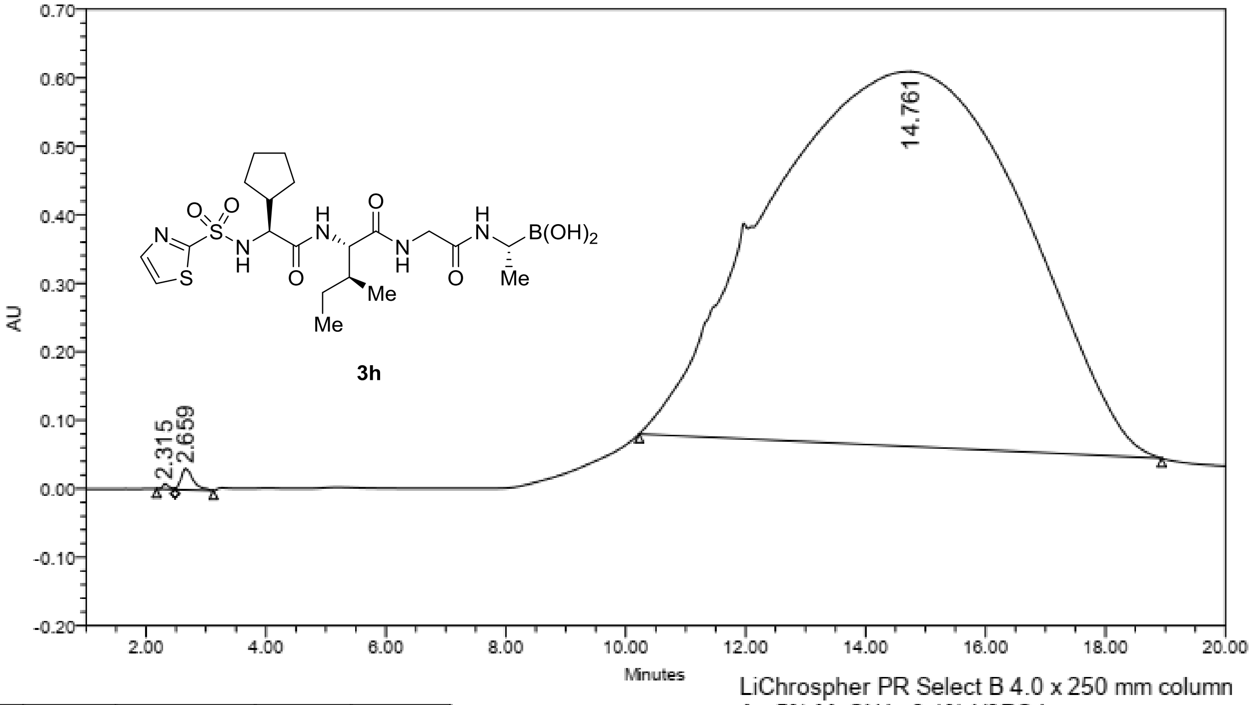


LiChrospher PR Select B 4.0 x 250 mm column
A 5% MeCN in 0.1% H3PO4
B 95% MeCN in 0.1% H3PO4
Gradient 15 min 0% A to 100% B; 5 min 100% B
Column Temperature 40oC
Flow Rate: 1.0 ml/min

| | RT | Area | % Area | Height |
|---|---|---|---|---|
| 1 | 2.315 | 56447 | 0.03 | 7455 |
| 2 | 2.659 | 431249 | 0.27 | 31180 |
| 3 | 14.761 | 161940290 | 99.70 | 547420 |

Reported by User: Olita
Report Method: Default Individual Report
Report Method ID 12125
Page: 1 of 1

Project Name: Martins
Date Printed:
2024.04.26.
16:25:36 Europe/Riga

# SAMPLE INFORMATION

| | | | |
|---|---|---|---|
| Sample Name: | osm-EP_1506 | Acquired By: | Olita |
| Sample Type: | Unknown | Sample Set Name: | 26042024p |
| Vial: | 5 | Acq. Method Set: | MeCN_0_5min_100% |
| Injection #: | 1 | Processing Method: | Processing |
| Injection Volume: | 30.00 ul | Channel Name: | W2489 ChB |
| Run Time: | 20.0 Minutes | Proc. Chnl. Descr.: | W2489 ChB 254nm |
| Date Acquired: | 2024.04.26. 15:31:28 EET | | |
| Date Processed: | 2024.04.26. 16:24:05 EET | | |

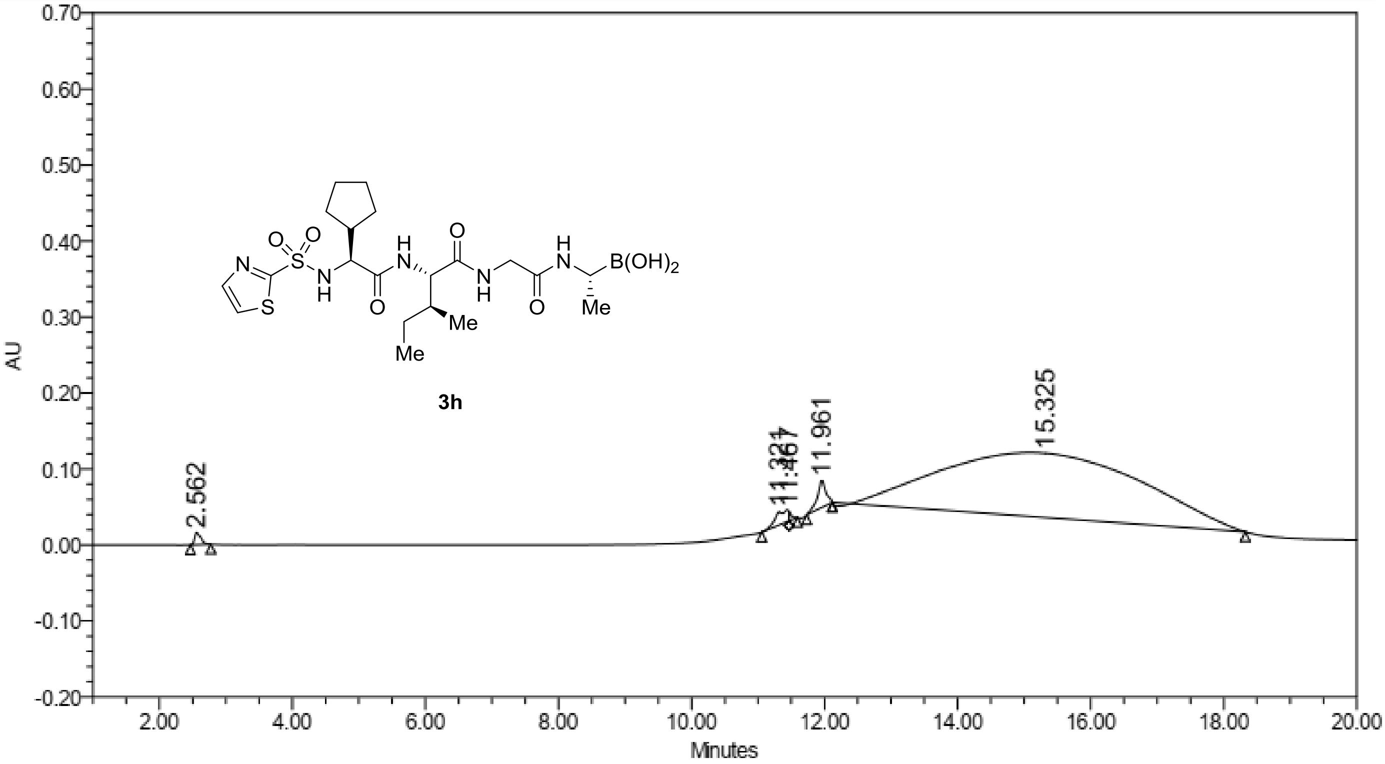


| | RT | Area | % Area | Height |
|---|---|---|---|---|
| 1 | 2.562 | 103532 | 0.56 | 15944 |
| 2 | 11.321 | 220761 | 1.19 | 16922 |
| 3 | 11.467 | 31279 | 0.17 | 12164 |
| 4 | 11.961 | 314452 | 1.69 | 35193 |
| 5 | 15.325 | 17937149 | 96.40 | 84529 |

LiChrospher PR Select B 4.0 x 250 mm column
A 5% MeCN in 0.1% H3PO4
B 95% MeCN in 0.1% H3PO4
Gradient 15 min 0% A to 100% B; 5 min 100% B
Column Temperature 40oC
Flow Rate: 1.0 ml/min

Reported by User: Olita
Report Method: Default Individual Report
Report Method ID 12125
Page: 1 of 1

Project Name: Martins
Date Printed:
2024.04.26.
16:25:05 Europe/Riga

# SAMPLE INFORMATION

| | | | |
|---|---|---|---|
| Sample Name: | osm-EP_1800 | Acquired By: | Olita |
| Sample Type: | Unknown | Sample Set Name: | 26042024 |
| Vial: | 7 | Acq. Method Set: | MeCN_0_5min_100% |
| Injection #: | 1 | Processing Method: | Processing |
| Injection Volume: | 30.00 ul | Channel Name: | W2489 ChA |
| Run Time: | 20.0 Minutes | Proc. Chnl. Descr.: | W2489 ChA 210nm |
| Date Acquired: | 2024.04.26. 12:53:45 EET | | |
| Date Processed: | 2024.04.26. 13:35:35 EET | | |

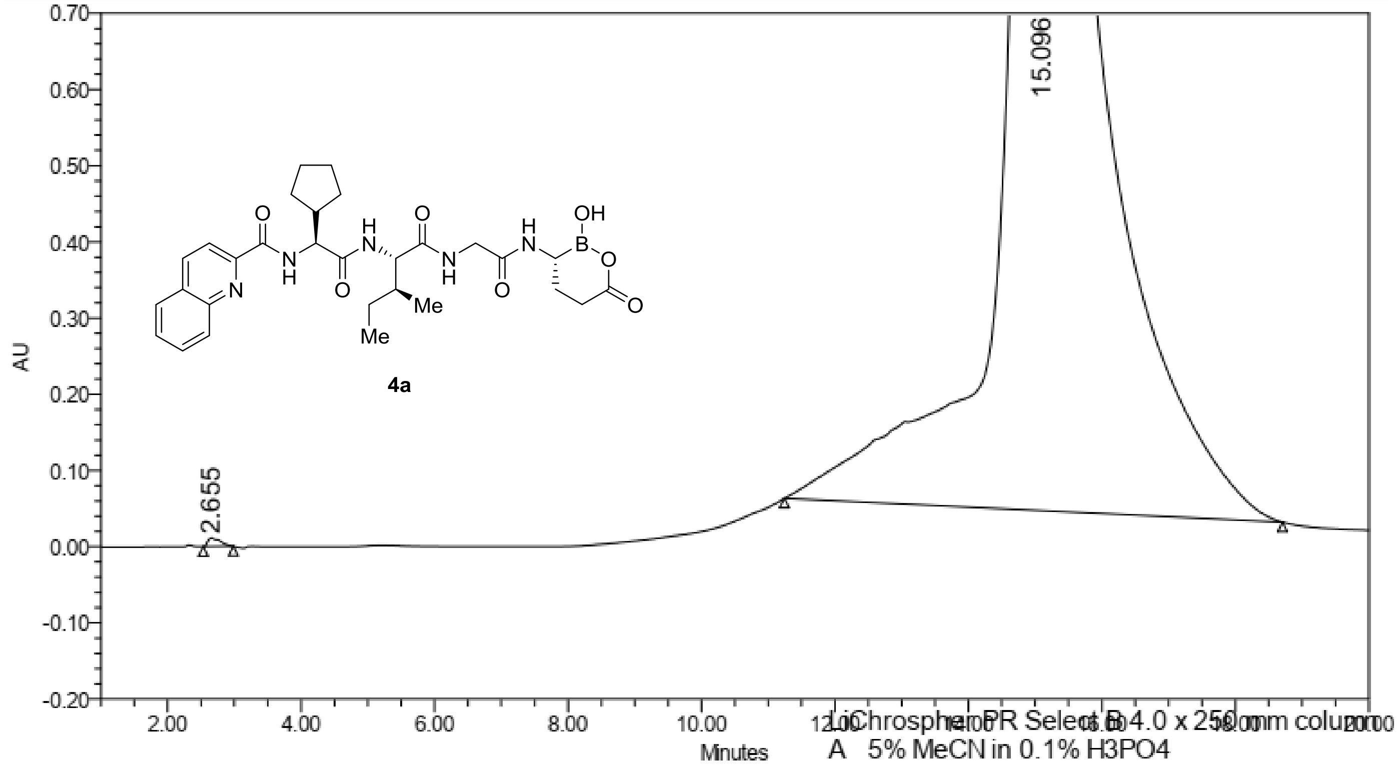


LiChrospher RP Select B 4.0 x 250 mm column
A 5% MeCN in 0.1% H3PO4
B 95% MeCN in 0.1% H3PO4
Gradient 15 min 0% A to 100% B; 5 min 100% B
Column Temperature 40oC
Flow Rate: 1.0 ml/min

| | RT | Area | % Area | Height |
|---|---|---|---|---|
| 1 | 2.655 | 145944 | 0.10 | 11451 |
| 2 | 15.096 | 146271984 | 99.90 | 1576171 |

Reported by User: Olita
Report Method: Default Individual Report
Report Method ID 12125
Page: 1 of 1

Project Name: Martins
Date Printed:
2024.04.26.
13:37:22 Europe/Riga

# SAMPLE INFORMATION

| | | | |
|---|---|---|---|
| Sample Name: | osm-EP_1800 | Acquired By: | Olita |
| Sample Type: | Unknown | Sample Set Name: | 26042024 |
| Vial: | 7 | Acq. Method Set: | MeCN_0_5min_100% |
| Injection #: | 1 | Processing Method: | Processing |
| Injection Volume: | 30.00 ul | Channel Name: | W2489 ChB |
| Run Time: | 20.0 Minutes | Proc. Chnl. Descr.: | W2489 ChB 254nm |
| Date Acquired: | 2024.04.26. 12:53:45 EET | | |
| Date Processed: | 2024.04.26. 13:34:37 EET | | |

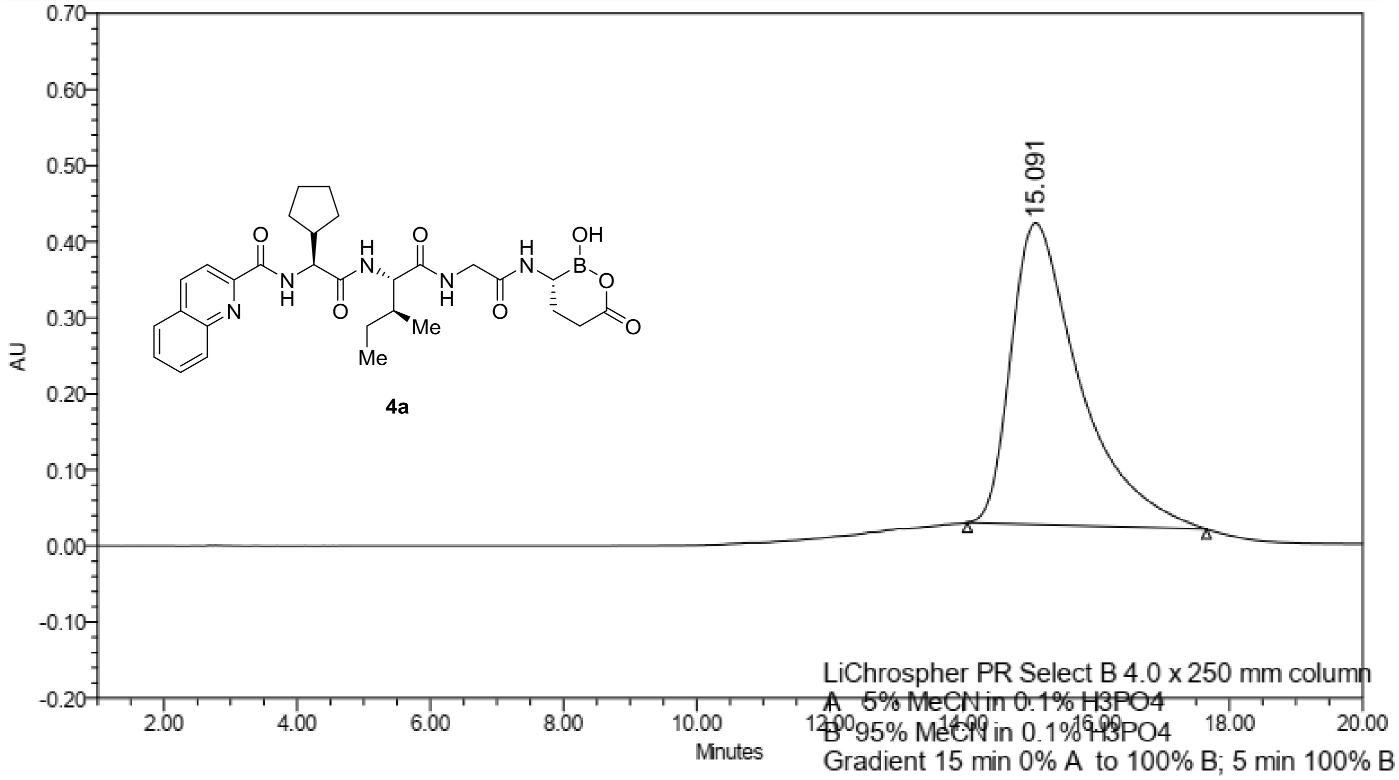


LiChrospher PR Select B 4.0 x 250 mm column
A 5% MeCN in 0.1% H3PO4
B 95% MeCN in 0.1% H3PO4
Gradient 15 min 0% A to 100% B; 5 min 100% B
Column Temperature 40oC
Flow Rate: 1.0 ml/min

| | RT | Area | % Area | Height |
|---|---|---|---|---|
| 1 | 15.091 | 28469907 | 100.00 | 395971 |

Reported by User: Olita
Report Method: Default Individual Report
Report Method ID 12125
Page: 1 of 1

Project Name: Martins
Date Printed:
2024.04.26.
13:36:48 Europe/Riga

# SAMPLE INFORMATION

| | | | |
|---|---|---|---|
| Sample Name: | osm-EP_1913 | Acquired By: | Olita |
| Sample Type: | Unknown | Sample Set Name: | 26042024 |
| Vial: | 2 | Acq. Method Set: | MeCN_0_5min_100% |
| Injection #: | 1 | Processing Method: | Processing |
| Injection Volume: | 30.00 ul | Channel Name: | W2489 ChA |
| Run Time: | 20.0 Minutes | Proc. Chnl. Descr.: | W2489 ChA 210nm |
| Date Acquired: | 2024.04.26. 10:44:42 EET | | |
| Date Processed: | 2024.04.26. 13:32:01 EET | | |

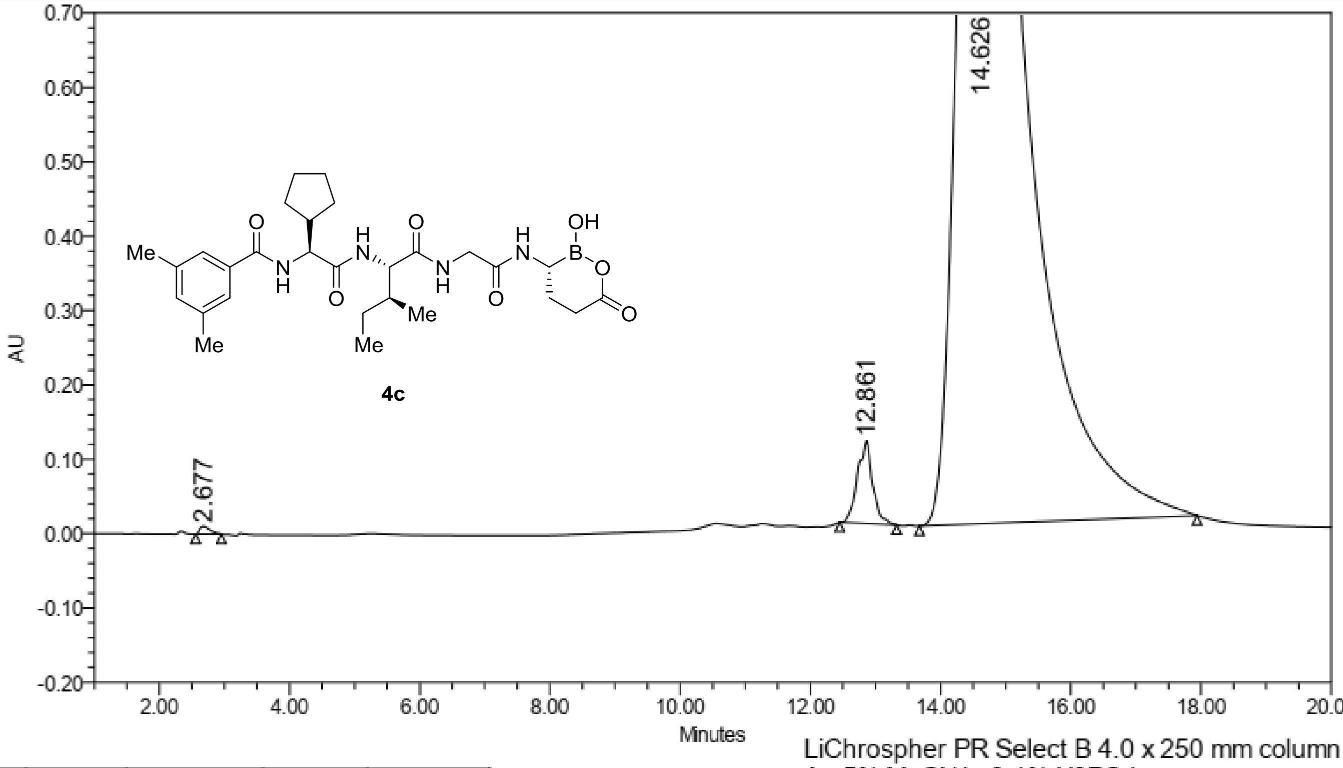


| | RT | Area | % Area | Height |
|---|---|---|---|---|
| 1 | 2.677 | 112442 | 0.11 | 10184 |
| 2 | 12.861 | 1778266 | 1.76 | 110924 |
| 3 | 14.626 | 99338490 | 98.13 | 1527402 |

LiChrospher PR Select B 4.0 x 250 mm column
A 5% MeCN in 0.1% H3PO4
B 95% MeCN in 0.1% H3PO4
Gradient 15 min 0% A to 100% B; 5 min 100% B
Column Temperature 40oC
Flow Rate: 1.0 ml/min

Reported by User: Olita
Report Method: Default Individual Report
Report Method ID 12125
Page: 1 of 1

Project Name: Martins
Date Printed:
2024.04.26.
13:36:11 Europe/Riga

## SAMPLE INFORMATION

| | | | |
|---|---|---|---|
| Sample Name: | osm-EP_1913 | Acquired By: | Olita |
| Sample Type: | Unknown | Sample Set Name: | 26042024 |
| Vial: | 2 | Acq. Method Set: | MeCN_0_5min_100% |
| Injection #: | 1 | Processing Method: | Processing |
| Injection Volume: | 30.00 ul | Channel Name: | W2489 ChB |
| Run Time: | 20.0 Minutes | Proc. Chnl. Descr.: | W2489 ChB 254nm |
| Date Acquired: | 2024.04.26. 10:44:42 EET | | |
| Date Processed: | 2024.04.26. 13:27:48 EET | | |

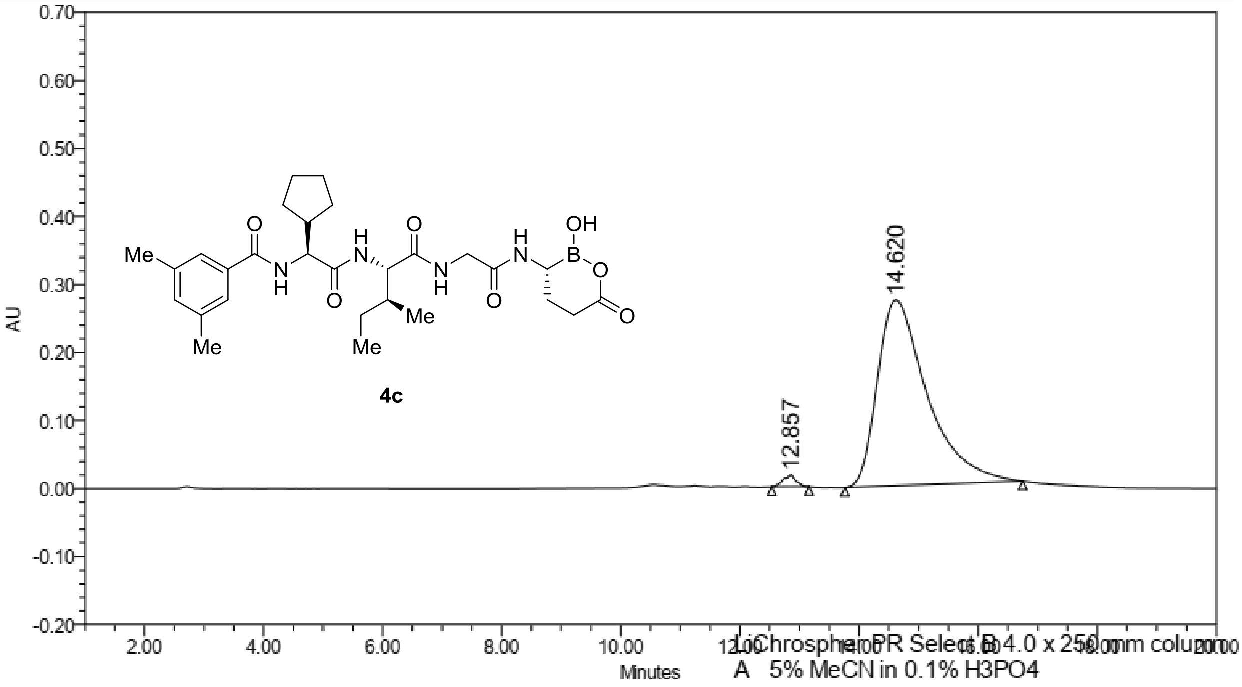


LiChrospher PR Select B 4.0 x 250 mm column
A 5% MeCN in 0.1% H3PO4
B 95% MeCN in 0.1% H3PO4
Gradient 15 min 0% A to 100% B; 5 min 100% B
Column Temperature 40oC
Flow Rate: 1.0 ml/min

| | RT | Area | % Area | Height |
|---|---|---|---|---|
| 1 | 12.857 | 253479 | 1.57 | 17289 |
| 2 | 14.620 | 15915867 | 98.43 | 273005 |

Reported by User: Olita
Report Method: Default Individual Report
Report Method ID 12125
Page: 1 of 1

Project Name: Martins
Date Printed:
2024.04.26.
13:28:39 Europe/Riga
